# Selective outcome reporting in randomised controlled trials including participants with stroke or transient ischaemic attack: A systematic review

**DOI:** 10.1177/23969873231194811

**Published:** 2023-08-22

**Authors:** Mohshin Syed, Helena Martin, Emily S Sena, Paula R Williamson, Rustam Al-Shahi Salman

**Affiliations:** 1 Medical School, University of Edinburgh, Edinburgh, UK; 2 Centre for Clinical Brain Sciences, University of Edinburgh, Edinburgh, UK; 3 MRC-NIHR Trials Methodology Research Partnership, Department of Health Data Science, University of Liverpool, Liverpool, UK

**Keywords:** Stroke, transient ischaemic attack, outcome reporting bias, selective outcome reporting, randomised controlled trial

## Abstract

**Introduction:**

The prevalence of outcome reporting bias (ORB, i.e. selective reporting according to the results observed) across primary outcomes in randomised controlled trials (RCTs) including participants with stroke or transient ischaemic attack (TIA) is unknown.

**Materials and Methods:**

We searched the Cochrane Database of Systematic Reviews on 3 February 2021 for reviews published 2008–2020 with at least one RCT of a therapeutic intervention, for participants with stroke or TIA, and a safety or efficacy outcome. We took a random sample of these RCTs and included those with a trial registry record or protocol published before reporting results. Two reviewers assessed discrepancies in outcome reporting across the trial registry record, protocol, statistical analysis plan, and publication for each RCT, using the classification system designed by the Outcome Reporting Bias in Trials group.

**Results:**

Of 600 RCTs, we identified a trial registry record in 120 (20%), a protocol in 28 (5%), and a statistical analysis plan in 5 (1%) with 123 (21%) distinct RCTs being eligible for assessment: 110 (89%, 95% CI 83–94) were at no risk, 7 (6%, 95% CI 3–11) RCTs were at low risk, and 6 (5%, 95% CI 2–10) were at high risk of ORB.

**Discussion:**

The prevalence of ORB in primary outcomes was low in stroke/TIA RCTs that were included in Cochrane reviews and had an identifiable trial registry record or protocol. Concerningly, we were unable to identify a trial registry record or protocol in most of our sample.

**Conclusion:**

Work is needed to further reduce ORB in stroke/TIA RCTs and explore the generalisability of these findings to RCTs outside of Cochrane reviews or without a registry record or protocol, as well as to secondary outcomes.

## Introduction

Randomised controlled trials (RCTs) are the most reliable assessment of the effectiveness of diagnostic tests or therapeutic interventions,^1–3^ but they are at risk of bias. The main sources of bias are selection bias, performance bias, detection bias, attrition bias, and outcome reporting bias (ORB). An RCT is at risk of ORB where the selection of outcomes reported differs from the outcomes pre-specified in the trial registry record, RCT protocol, or statistical analysis plan.^4,5^ ORB occurs when the statistical significance or the effect size of the results drive what outcomes are reported.^6^

Selective outcome reporting can occur in varying forms: outcomes may be modified or omitted in comparison to the original set of outcomes; particular data for an outcome may be selectively reported (e.g. reporting measurements for select timepoints); or non-significance of an outcome may be reported with absence of any recorded data – incomplete reporting.

ORB is common. In a study conducted by the Outcome Reporting Bias in Trials (ORBIT) group, 14% of RCTs contained within a random sample of Cochrane systematic reviews were suspected of being at high risk of ORB.^7^ Furthermore, 33% of the reviews contained one or more of the high risk RCTs. The presence of ORB within RCTs biases treatment effect; a sensitivity analysis for ORB in the primary outcome of reviews demonstrated a reduction in treatment effect by at least 20% in 23% of reviews.^7^

Selective outcome reporting in RCTs including participants with stroke or transient ischaemic attack (TIA) was identified as a knowledge gap in 2016 and remains so now.^8^ We conducted a comprehensive literature search of MEDLINE via PubMed on 22 January 2021 (see Supplemental Materials) for studies of ORB in RCTs including participants with stroke or TIA. None of the 4776 articles that we identified addressed ORB in RCTs including participants with stroke or TIA. A search of the PROSPERO database and ORBIT publications did not reveal such studies either.^9^ Therefore, we aimed to assess the prevalence of ORB in RCTs including participants with stroke or TIA.

## Methods

### Protocol and registration

The study protocol is publicly available on the Open Science Framework (https://doi.org/10.17605/OSF.IO/QTNFY).

### Eligibility criteria

We sought RCTs identified by Cochrane systematic reviews published between 1 March 2008 to 1 March 2020, when risk of bias assessment would have been completed by review authors after implementation of the Cochrane risk-of-bias tool in February 2008.^10^ We required reviews to include at least one completed, published RCT, including human participants with stroke (ischaemic or haemorrhagic (i.e. intracerebral haemorrhage or subarachnoid haemorrhage)) or TIA. We included the final results of RCTs, not the results of any interim analyses.

We required individual RCTs to have a trial registry record and/or a protocol that investigators had recorded before the reporting of the RCT results. We included RCTs of therapeutic interventions, but not RCTs of diagnostic tests. We required RCTs to have reported at least one safety or efficacy outcome. We included RCTs excluded from reviews due to having no relevant outcome data, as unreported outcomes may still have been measured and excluded on the basis of their results, which would indicate a risk of ORB.

### Information sources

We searched the Cochrane Database of Systematic Reviews via The Cochrane Library Interface (https://www.cochranelibrary.com/advanced-search) on 3 February 2021 for systematic reviews including RCTs that met our eligibility criteria. We sought prospective records of trial registry information, protocol and statistical analysis plan, using a four-stage approach. Firstly, we examined the RCT publication as well as the systematic review that identified it. Secondly, we searched the following trial registries: ClinicalTrials.gov (www.clinicaltrials.gov/); ISRCTN (https://www.isrctn.com/); EU Clinical Trials Register (www.clinicaltrialsregister.eu/) and, where appropriate, the country-specific trial registry. Thirdly, we searched the associated journal website, Google and PubMed, using keyword searches; for example [Title of RCT Publication] AND ‘protocol’. Where these approaches were unsuccessful, we contacted the corresponding authors of the RCT to ascertain the whereabouts of a prospective record of a trial registry entry or protocol.

We had initially planned to use the WHO International Clinical Trials Registry Platform (WHO ICTRP), which is comprised of a global network of databases of 17 primary clinical trial registries and would have allowed for a more expansive pool to search from.^11^ However, due to increased traffic brought about by the COVID-19 pandemic, the WHO ICTRP was non-functional during the data collection period.

### Selection and data collection process

Two independent reviewers (MS and HM) screened the titles and abstracts of each record and classified them as ‘eligible’ (as per the eligibility criteria), ‘might be eligible’ or ‘not eligible’.^12^ We read the full text of records deemed ‘might be eligible’ to determine if the eligibility criteria were satisfied.^13^ We documented our reasons where we deemed a record ‘not eligible’ and excluded. We resolved disagreements through discussion, with a third reviewer (RASS) having final say where an agreement could not be reached. We used SyRF (Systematic Review & Meta-analysis Facility) – an online platform developed for data management and analysis of large reference groups for systematic reviews and meta-analyses (https://app.syrf.org.uk/) – for data collection.

### Data items

#### Primary outcome

Our primary outcome was the prevalence of ORB in stroke/TIA RCTs. We expressed ORB prevalence as the proportion of all RCTs identified that we deemed at high risk of ORB according to categories A, D, E and G of the ORBIT classification system for missing or incomplete outcome reporting (Table 1).

**Table 1.** ORBIT classifications for risk of outcome reporting bias (ORB) in benefit outcomes.^7^

Classification	Description	Level of reporting	Level of suspicion of ORB	
Clear that the outcome was measured and analysed				
A	Trial report states that outcome was analysed but only reports that result was not significant (typically stating *p*-value >0.05)	Partial	High risk	
B	Trial report states that outcome was analysed but only reports that result was significant (typically stating *p*-value <0.05)	Partial	No risk	
C	Trial report states that outcome was analysed but insufficient data were presented for the trial to be included in meta-analysis or to be considered to be fully tabulated	Partial	Low risk	
D	Trial report states that outcome was analysed but no results reported	None	High risk	
Clear that the outcome was measured				
E	Clear that outcome was measured but not necessarily analysed. Judgement says likely to have been analysed but not reported because of non-significant results	None	High risk	
F	Clear that outcome was measured but not necessarily analysed. Judgement says unlikely to have been analysed	None	Low risk	
Unclear that the outcome was measured				
G	Not mentioned but clinical judgement says likely to have been measured and analysed but not reported on the basis of non-significant results	None	High risk	
H	Not mentioned but clinical judgement says unlikely to have been measured at all	None	Low risk	
Clear that the outcome was NOT measured				
I	Clear that outcome was not measured	N/A	No risk	

ORBIT classifications, for benefit outcomes, as an indicator for the level of reporting of outcomes within published trials, and the level of suspicion of outcome reporting bias based on the nature of reporting.

#### Secondary outcomes

Our secondary outcomes included the difference in prevalence of ORB between trial registry record, protocol, and statistical analysis plan, and the prevalence of a change in outcome reporting (i.e. upgraded, downgraded, modified, added, omitted, partially reported or fully reported). We had intended to investigate the association of RCT-level characteristics (year of trial publication; sample size; meeting trial recruitment target; statistically significant result for the primary outcome(s) and funding sources) with the primary outcome, but ultimately the number of primary outcomes was insufficient to allow univariate or multivariable analyses.

### Data synthesis

We extracted the primary outcomes described in the trial registry record, protocol, and statistical analysis plan (if available and recorded before RCT results were reported) for each eligible RCT. We compared the extracted outcomes with those primary outcomes reported at final publication – that is, trial registry record versus final publication, protocol versus final publication and statistical analysis plan versus final publication. For each primary outcome, we designated it ‘fully reported’; ‘partially reported’ (e.g. reporting of non-significance with absence of actual estimates); ‘not reported’ or ‘not measured’. In addition, we also recorded where the primary outcome(s) had been ‘upgraded’ (where a secondary outcome is shifted up to a primary outcome); ‘downgraded’ (where a primary outcome is shifted down to a secondary outcome); ‘added’ (where a new primary outcome had been included in the final publication) or ‘modified’ (where there has been alteration to an outcome’s definition but not severe enough to consider it a new outcome – e.g. a change in timeframe).

We used the ORBIT classification tool (Table 1) to assign risk classifications of ORB for each primary outcome per RCT.^14^ The ORBIT classification tool is reasonably accurate for assessing ORB with a sensitivity of 88% (95% CI 65–100) and a specificity of 80% (95% CI 69–100).^7^ We deemed an RCT to be at high risk of ORB where there was at least one high-risk classification (A, D, E or G) for any primary outcome within that trial. We classified RCTs as low risk of ORB where there was one or more low-risk classifications (C, F or H) but no high-risk classifications for any primary outcome. We deemed RCTs at no risk of ORB where every primary outcome had been determined to be ‘fully reported’ or had been assigned a no-risk classification (B or I).

## Results

### Study selection

**Figure 1.**
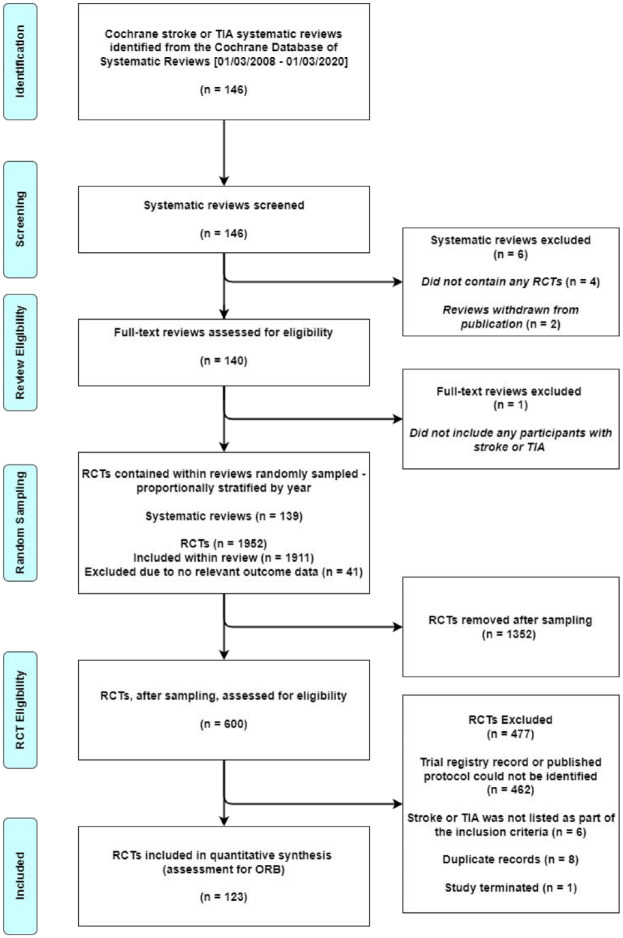
Study flow diagram.

Of the 146 Cochrane reviews, 139 were eligible. The 139 reviews contained 1952 RCTs. Due to time and resource constraints, we took a random sample, proportionally stratified by year, of 600 RCTs using a Python script. We identified a trial registry record for 120 of these 600 RCTs, a protocol for 28 RCTs, and a statistical analysis plan for five RCTs – all of which were prospectively recorded. Overall, 123 (21%) of the 600 sampled RCTs were eligible and assessed for ORB (Figure 1).

Characteristics of the included RCTs are shown in Table 2. We grouped the breakdown of characteristics into two periods (2001–2009 and 2010–2021). Each period corresponds to the publication of a revision to the CONSORT statement: a set of standardised guidelines on the reporting of RCTs.^15,16^

**Table 2.** Characteristics of 123 included RCTs.

Characteristics	*n* (%) of RCTs		
	Total RCTs (*n* = 123)	2001–2009 (*n* = 41)	2010–2021 (*n* = 82)
Type of stroke/TIA included			
Ischaemic or haemorrhagic	29 (24%)	9 (22%)	20 (24%)
Ischaemic stroke	30 (24%)	14 (34%)	16 (20%)
Haemorrhagic stroke	4 (3%)	4 (10%)	0
TIA or stroke^a^	17 (14%)	4 (10%)	13 (16%)
Subtype not specified	43 (35%)	10 (24%)	33 (40%)
Type of RCT			
Acute treatment	35 (28%)	20 (49%)	15 (18%)
Rehabilitation	56 (46%)	10 (24%)	46 (56%)
Secondary prevention	27 (22%)	10 (24%)	17 (21%)
Other	5 (4%)	1 (3%)	4 (5%)
Scale of trial			
Single centre	46 (37%)	9 (22%)	37 (45%)
Multi-centre	77 (63%)	32 (78%)	45 (55%)
Sample size			
Median (IQR)	101 (48–439)	321 (78– 1001)	71 (38–252)
Trial recruitment target met?			
Yes	59 (48%)	18 (44%)	41 (50%)
No	48 (39%)	18 (44%)	30 (37%)
Target unknown	16 (13%)	5 (12%)	11 (13%)
RCT outcome			
Positive for all primary outcomes	55 (45%)	16 (39%)	39 (48%)
Neutral for all primary outcomes	67 (54%)	25 (61%)	42 (51%)
Negative for all primary outcomes	1 (<1%)	0	1 (<1%)
Funding source			
Commercial	21 (17%)	12 (29%)	9 (11%)
Government or charity	59 (48%)	15 (37%)	44 (54%)
Both	12 (10%)	4 (10%)	8 (10%)
Neither	31 (25%)	10 (24%)	21 (25%)

Type of stroke/TIA included: haemorrhagic stroke is defined as either intracerebral haemorrhage or subarachnoid haemorrhage.

RCT outcome: positive = statistically significant benefit in intervention; neutral = not statistically significant; negative = statistically significant harm in intervention.

a Ischaemic or haemorrhagic stroke.

### Assessment of outcome reporting bias

The breakdown of ORBIT classifications for the 123 RCTs is shown in Table 3 (for further information, see Supplemental Materials). The majority of RCTs were at no risk of ORB (89%, *n* = 110). These RCTs either fully reported all primary outcomes (*n* = 109) or fell into the ‘I, clearly not measured’ category (*n* = 1). This RCT did not report one of their primary outcomes that was pre-specified in their trial registry record, but the trialists provided justification that due to a substantial proportion of participants being unable to participate in the test, they were unable to suitably measure their outcome.^17^

**Table 3.** Risk of ORB in 123 RCTs of stroke or TIA.

ORBIT classifications	Descriptions	Frequency	*n* (%, 95% CI)
High risk			
A	Trial report states that outcome was analysed but only reports that result was not significant (typically stating *p*-value >0.05)	2	
D	Trial report states that outcome was analysed but no results reported	0	
E	Clear that outcome was measured but not necessarily analysed. Judgement says likely to have been analysed but not reported because of non-significant results	0	**6** (5%, 95% CI 2–10)
G	Not mentioned but clinical judgement says likely to have been measured and analysed but not reported on the basis of non-significant results	4	
Low risk			
C	Trial report states that outcome was analysed but insufficient data were presented for the trial to be included in meta-analysis or to be considered to be fully tabulated	0	
F	Clear that outcome was measured but not necessarily analysed. Judgement says unlikely to have been analysed	0	**7** (6%, 95% CI 3–11)
H	Not mentioned but clinical judgement says unlikely to have been measured at all	7	
No risk			
I	Clear that outcome was not measured	1	
Fully reported	Outcome was fully reported	109	**110** (89%, 95% CI 83–94)

Values in brackets represent *95% confidence intervals*, calculated using the Wilson score.^18^

A small proportion of RCTs were at low risk of ORB (*n* = 7, 6%). We assigned all low-risk RCTs ‘H’ classifications, where it was unclear whether the outcome had been measured. This took two forms: a primary outcome would not be reported, and there would be a lack of evidence to conclude the outcome had been measured (*n* = 3), or the RCTs would report select timepoints from a longer list pre-specified in their trial registry record or protocol without any reasoning (*n* = 4). In either scenario, the primary outcome(s) of the RCTs had not been fully reported, but it was unclear whether the outcome was measured and the trialists had subsequently chosen to not fully report the results. Hence, a low risk of ORB.

There was also a small proportion of RCTs deemed to be at high risk of ORB (5%, *n* = 6) in two classification groups A and G. The A-classified RCTs (*n* = 2) had partially reported their primary outcomes. They did not present sufficient data; instead, they only reported that the results were non-significant. The G-classified RCTs (*n* = 4) had not reported one or more of their primary outcomes; however, there was evidence to conclude that the outcome had likely, but not clearly, been measured. Overall, the RCTs at high risk of ORB were likely to have not reported their primary outcomes as originally specified in their trial registry record or protocol due to knowledge of the outcome’s results.

The changes in level of reporting of primary outcomes between trial registry record, protocol, statistical analysis plan and final publication are shown in Table 4. The most prevalent form of discrepancy in outcome level was modification (25% of trial registry records). We identified four main categories of modification: change in measurement methods (37% of modifications), change in timepoints (33%), change in outcome definition (27%) and selective reporting of a subset of data (3%). Of those RCTs we identified to have some form of discrepancy, we assessed 24% to be at, high or low, risk of ORB. Although the remaining RCTs had changed the level of reporting of a primary outcome in some respect, we found these deviations were not major enough to conclude that the RCT was at risk of ORB (see Supplemental Materials).

**Table 4.** Change in primary outcomes between trial registry record, trial protocol, trial statistical analysis plan, and final publication.

Category of change	*n* (%, 95% CI) of RCTs		
	Trial registry (*n* = 120)	Protocol (*n* = 28)	Statistical analysis plan (*n* = 5)
No discrepancy^a^	66 (55%, 95% CI 46–64)	22 (79%, 95% CI 60–90)	4 (80%, 95% CI 38–96)
Discrepancy^b^	54 (45%, 95% CI 36–54)	6 (21%, 95% CI 10–40)	1 (20%, 95% CI 4–62)
Upgraded	10 (8%, 95% CI 5–15)	0	0
Downgraded	11 (9%, 95% CI 5–16)	1 (4%, 95% CI 1–18)	1 (20%, 95% CI 4–62)
Added	10 (8%, 95% CI 5–15)	2 (7%, 95% CI 2–23)	0
Omitted^c^	8 (7%, 95% CI 3–13)	0	0
Partially reported	7 (6%, 95% CI 3–12)	1 (4%, 95% CI 1–18)	0
Modified	30 (25%, 95% CI 18–33)	2 (7%, 95% CI 2–23)	0

Values in brackets represent *95% confidence intervals*, calculated using the Wilson score.^18^

a ‘No discrepancy’: fully reported all outcomes with no upgrading, downgrading, modification, addition or omission.

b The cumulative frequency of sub-categories of ‘Discrepancy’ may exceed the frequency of ‘Discrepancy’. This is because a single RCT may display multiple different changes in outcome reporting. For example, an RCT may have both upgraded and partially reported an outcome; this would, therefore, be counted twice.

c Omitted: outcomes ‘not reported’ or ‘not measured’.

## Discussion

### Summary of principal findings

Overall, there was a slightly lower prevalence of ORB in stroke/TIA RCTs (5% high risk of ORB) in comparison to the overall prevalence of ORB in RCTs (14% high risk of ORB),^7^ and a small number of estimates of ORB in RCTs of other diseases (e.g. 29% of cystic fibrosis RCTs were at high risk of ORB^19^).

Despite our study finding a relatively low prevalence of ORB in stroke/TIA RCTs, there were still areas of concern. Whilst we classified the majority of RCTs as ‘No risk – Fully reported’ (89%), only 55% were fully reported with no upgrading, downgrading, modification, addition, omission or partial reporting of any outcomes (Table 4). Inconsistencies in outcome reporting such as the upgrading or downgrading of outcomes would not lead to ORB, but rather to ‘spin bias’, which affected 17% of RCTs in comparison to their trial registry entries and 4% in comparison to their published protocol.^20^

### Strengths and limitations of the study

This study is subject to several limitations. The assessment and classification of risk of ORB require a degree of subjective judgement. This, therefore, introduces susceptibility to interpretation bias.^21^ We were able to reduce subjectivity to some extent using the ORBIT classification system, which provided a more structured approach to assessing ORB, coupled with two reviewers independently assessing the RCTs. We found disagreements most commonly occurred with RCTs that we deemed to have ‘modified’ an outcome. In many of these cases, there was clearly some discrepancy in the reporting of an outcome; however, there was often a lack of evidence to conclude the RCT was at risk of ORB. For example, in the case of changing timepoints, the assessment of ORB was unclear unless the trialists provided an explanation of the change in an updated trial registry record or protocol (no risk of ORB), or it was clearly demonstrated that they selected particular timepoint(s) from a longer pre-specified list with no explanation (potentially at risk of ORB). To overcome this limitation, we contacted trialists to provide clarification where discrepancies exist.

We were unable to identify a trial registry record or protocol in the majority of our random sample (*n* = 462, 77%). Our results may, therefore, underestimate the prevalence of ORB in stroke/TIA RCTs, as we assume that trialists who have not clearly provided trial registration or protocol information may be more prone to incomplete outcome reporting. Prospective trial registration is critical in maintaining transparency in research. Inadequate provision of trial registry or protocol information hinders the reader’s ability to compare outcome reporting at final publication, thereby making the assessment of risk of ORB unfeasible. Trial registration has improved over time following the International Committee of Medical Journal Editors’ mandate for prospective trial registration.^22,23^ However, our study’s results, similar to a number of others,^24–26^ indicate the need for improvement.

### Comparison with other studies

To our knowledge, our study is the first to assess the prevalence of ORB in stroke/TIA RCTs. Our results revealed a comparatively smaller prevalence of ORB in comparison to previous studies assessing the risk of ORB in other diseases, using similar methods.^7,19,27^ However, this comparison may be affected by the differing publication date ranges of each study’s sample: RCTs identified by Cochrane reviews published prior to 2007,^7^ 2010^19^ and 2012,^27^ respectively. The CONSORT statement was last updated in 2010 and was changed from the previous statement to include an item identifying any changes between outcomes following trial commencement.^16^ A previous study assessing the reporting of stroke RCTs with regards to the CONSORT reporting guidelines found that there was a statistically significant improvement in the reporting of outcomes, following the revision to the CONSORT statement in 2010, when compared using items from the CONSORT checklist.^28^ Hence, we can expect stroke RCTs published after 2010 to be less likely to selectively report outcomes and, thereby, less likely to be at risk of ORB.

### Implications for research and practice

Selective outcome reporting may lead to ORB which can potentially jeopardise the validity of research and has far-reaching implications. At the RCT-level, ORB may distort trial results, which can subsequently impact the findings of systematic reviews and guidelines. Previous studies have demonstrated the capability for ORB to lead to overestimation of a treatment effect.^7^ This might lead to interventions being recommended in clinical practice when in reality they are ineffective or harmful.

Whilst our study’s results demonstrate the prevalence of ORB in stroke/TIA RCTs to be relatively low, we have highlighted areas that need improvement. Trialists should provide trial registration numbers and/or protocol information at publication. This is necessary in enabling readers to be able to identify any discrepancies as well as to discourage selective outcome reporting. Preferably, trialists would follow trial registration with publication of a protocol and statistical analysis plan prior to data collection or analysis.^29^ Alterations of outcome level may be acceptable after trial registration but are more concerning after protocol publication. Where there are any deviations, trialists should publicly document any adjustments and provide an explanation at publication so that readers and peer-reviewers can more easily assess for any risks of bias.

### Future research

Further work is needed to explore the extent to which our findings can be applied to the wider stroke/TIA literature through assessing the risk of ORB in RCTs not included in Cochrane reviews, and ORB in secondary outcomes.

## Conclusions

The prevalence of ORB in stroke/TIA RCTs is low. However, as we were unable to identify a trial registry record or protocol in most of our sample, our results may underestimate ORB prevalence. Trialists should be clear at all stages of reporting, from trial registry record to protocol to statistical analysis plan to publication. Where there are changes to planned outcome reporting, a justification should be provided.

## Supplemental Material

sj-pdf-2-eso-10.1177_23969873231194811 – Supplemental material for Selective outcome reporting in randomised controlled trials including participants with stroke or transient ischaemic attack: A systematic review Click here for additional data file. Supplemental material, sj-pdf-2-eso-10.1177_23969873231194811 for Selective outcome reporting in randomised controlled trials including participants with stroke or transient ischaemic attack: A systematic review by Mohshin Syed, Helena Martin, Emily S Sena, Paula R Williamson and Rustam Al-Shahi Salman in European Stroke Journal

sj-pdf-3-eso-10.1177_23969873231194811 – Supplemental material for Selective outcome reporting in randomised controlled trials including participants with stroke or transient ischaemic attack: A systematic review Click here for additional data file. Supplemental material, sj-pdf-3-eso-10.1177_23969873231194811 for Selective outcome reporting in randomised controlled trials including participants with stroke or transient ischaemic attack: A systematic review by Mohshin Syed, Helena Martin, Emily S Sena, Paula R Williamson and Rustam Al-Shahi Salman in European Stroke Journal

sj-pdf-4-eso-10.1177_23969873231194811 – Supplemental material for Selective outcome reporting in randomised controlled trials including participants with stroke or transient ischaemic attack: A systematic review Click here for additional data file. Supplemental material, sj-pdf-4-eso-10.1177_23969873231194811 for Selective outcome reporting in randomised controlled trials including participants with stroke or transient ischaemic attack: A systematic review by Mohshin Syed, Helena Martin, Emily S Sena, Paula R Williamson and Rustam Al-Shahi Salman in European Stroke Journal

sj-pdf-5-eso-10.1177_23969873231194811 – Supplemental material for Selective outcome reporting in randomised controlled trials including participants with stroke or transient ischaemic attack: A systematic review Click here for additional data file. Supplemental material, sj-pdf-5-eso-10.1177_23969873231194811 for Selective outcome reporting in randomised controlled trials including participants with stroke or transient ischaemic attack: A systematic review by Mohshin Syed, Helena Martin, Emily S Sena, Paula R Williamson and Rustam Al-Shahi Salman in European Stroke Journal

sj-xlsx-1-eso-10.1177_23969873231194811 – Supplemental material for Selective outcome reporting in randomised controlled trials including participants with stroke or transient ischaemic attack: A systematic review Click here for additional data file. Supplemental material, sj-xlsx-1-eso-10.1177_23969873231194811 for Selective outcome reporting in randomised controlled trials including participants with stroke or transient ischaemic attack: A systematic review by Mohshin Syed, Helena Martin, Emily S Sena, Paula R Williamson and Rustam Al-Shahi Salman in European Stroke Journal
