# Supplementary material for: Selective outcome reporting in randomised controlled trials including participants with stroke or transient ischaemic attack: A systematic review
Source: Eur Stroke J. 2023 Aug 22;8(4):923–31. doi: 10.1177/23969873231194811 (PMC10683727; doi:10.1177/23969873231194811)
Supplement: sj-pdf-2-eso-10.1177_23969873231194811 – Supplemental material for Selective outcome reporting in randomised controlled trials including participants with stroke or transient ischaemic attack: A systematic review [file sj-pdf-2-eso-10.1177_23969873231194811.pdf]

LITERATURE SEARCH STRATEGY

Table 1: Literature Search Strategy for Existing Research

Search Set	MEDLINE Search Terms	Results
**1**	report* bias	60,082
**2**	outcome report* bias	21,720
**3**	select* outcome report* bias	9,725
**4**	select* outcome report*	78,209
**5**	1-4/OR	128,566
**6**	cerebrovascular disorders	383,961
**7**	basal ganglia cerebrovascular disease	1,274
**8**	brain ischemia	128,960
**9**	brain infarction	54,262
**10**	ischemic attack, transient	26,249
**11**	vertebrobasilar insufficiency	5,523
**12**	carotid artery diseases	56,420
**13**	cerebral small vessel diseases	8,679
**14**	cerebral amyloid angiopathy, familial	299
**15**	stroke, lacunar	3,222
**16**	cerebrovascular trauma	10,423
**17**	vertebral artery dissection	2,749
**18**	intracranial arterial diseases	66,227
**19**	cerebral arterial diseases	24,759
**20**	cerebral amyloid angiopathy	3,396
**21**	infarction, anterior cerebral artery	2,227
**22**	infarction, middle cerebral artery	18,495
**23**	infarction, posterior cerebral artery	2,121
**24**	moyamoya disease	4,439
**25**	intracranial aneurysm	33,707
**26**	intracranial arteriosclerosis	11,804
**27**	intracranial arteriovenous malformations	10,547
**28**	intracranial embolism and thrombosis	21,912
**29**	intracranial hemorrhages	74,016
**30**	cerebral hemorrhage	62,110
**31**	intracranial hemorrhage, hypertensive	1,065
**32**	intracranial hemorrhage, traumatic	14,866
**33**	subarachnoid hemorrhage	32,386
**34**	stroke	360,602
**35**	vasospasm, intracranial	5,441
**36**	6-35/OR	602,198
**37**	5 AND 36	5,737
**38**	LIMIT 37 to English language AND humans	**4,776**

** = truncated term; AND/OR = Boolean operations; LIMIT = filter criteria*
We searched MEDLINE on 22 January 2021 to identify any existing research that may have addressed stroke or transient ischaemic attack (TIA) and selective outcome reporting. Literature search strategy for associated terms of stroke or TIA was adapted from the Cochrane Stroke Group.[1]

Table 2: Literature Search Strategy – Cochrane Database of Systematic Reviews

Search Set	CDSR Search Terms	Results
**1**	cerebrovascular disorder	3383
**2**	basal ganglia cerebrovascular disease	54
**3**	brain isch*mia	6769
**4**	brain infarct*	4526
**5**	transient near/5 isch*mic attack	3239
**6**	transient isch*	3992
**7**	vertebrobasilar insufficienc*	110
**8**	carotid artery disease	3082
**9**	cerebral small vessel disease	256
**10**	lacunar near/3 stroke	315
**11**	cerebrovascular trauma	205
**12**	vertebral artery dissection	43
**13**	intracranial artery disease	714
**14**	cerebral artery disease	103
**15**	cerebral amyloid angiopathy	0
**16**	familial near/5 (cerebral amyloid angiopathy)	1
**17**	(anterior cerebral artery) near/8 infarct*	51
**18**	(middle cerebral artery) near/8 infarct	51
**19**	(posterior cerebral artery) near/8 infarct	51
**20**	moyamoya disease	0
**21**	intracranial aneurysm	9
**22**	intracranial arteriosclerosis	1
**23**	intracranial arteriovenous malformation	2
**24**	intracranial embol*	12
**25**	intracranial thromb*	1635
**26**	intracranial h*morrhage	55
**27**	*cerebral h*morrhage	138
**28**	(intracranial h*morrhage) near/6 hyperten*	17
**29**	(intracranial h*morrhage) near/5 trauma	12
**30**	subarachnoid h*morrhage	17
**31**	intracranial near/3 vasospasm	3
**32**	Stroke	565
**33**	MeSH descriptor: [Stroke] explode all trees	9992
**34**	{OR #1-#33}	27014
**35**	LIMIT #34 to Cochrane Reviews AND Cochrane Group = Stroke	209
**36**	LIMIT #35 to published between: 01/03/2008 – 01/03/2020	**146**

** at the beginning of a term = inclusion of all prefixes; * within a term = acts as a wildcard to match multiple letter variations of spelling; * at the end of a term = inclusion of all suffixes; near/X = acts as a proximity operator to find terms within X words of each other; LIMIT = filter criteria.*

We searched the Cochrane Database for Systematic Reviews (CDSR) on 3 February 2021 for systematic reviews including randomised controlled trials (RCTs) that met our eligibility criteria.

Literature search strategy for associated terms of stroke or TIA was adapted from the Cochrane Stroke Group.[1]

References

1. Search Methods for Cochrane Stroke Specialised Register (updated 24/06/2020), https://apps.ccbs.ed.ac.uk/csrg/entity/searchmethods.pdf (accessed 25 January 2021).
