# Supplementary material for: Selective outcome reporting in randomised controlled trials including participants with stroke or transient ischaemic attack: A systematic review
Source: Eur Stroke J. 2023 Aug 22;8(4):923–31. doi: 10.1177/23969873231194811 (PMC10683727; doi:10.1177/23969873231194811)
Supplement: sj-pdf-4-eso-10.1177_23969873231194811 – Supplemental material for Selective outcome reporting in randomised controlled trials including participants with stroke or transient ischaemic attack: A systematic review [file sj-pdf-4-eso-10.1177_23969873231194811.pdf]

RATIONALE BEHIND RCT ORBIT CLASSIFICATIONS

Table 1: Rationale Behind RCTs given ORBIT Classifications

Study ID	Trial Registration ID	ORBIT Risk Classification	Rationale
Allison (2007)	ISRCTN66554171	H (Low Risk)	In the trial registry record, the authors outlined the following primary outcome:<br><br>1. "A battery of outcome measures will be recorded throughout the study. These are measures when the participant is asked to perform a number of tasks such as rolling over in bed, standing up, etc. The outcome measurement typically takes about 30-45 minutes to complete. Outcome measures will be taken on a weekly basis during the inpatient stay, and then at 12 weeks after the initial assessment."<br><br>At publication, more specific outcomes were used:<br><br>a. "The Gross Functional Tool Section of the Rivermead Motor Assessment<br>b. "Trunk Control Test"<br>c. "Berg Balance Scale"<br><br>The authors stated in the trial registry record that measurements would be taken on a weekly basis; however, only weeks 1, 2 and 12 were reported.<br><br>Given the generic outline of the primary outcome in the trial registry record and the far more specific definitions provided at publication, coupled with the change in timepoint selection, we believe there is a risk of ORB present. The final primary

			outcomes should be settled upon at the trial registration or protocol stage, or, at the very least, justifications should be provided for any later changes.<br><br>As it is unclear at which point the final primary outcome was decided, a H classification was attributed.
Ang (2014)	NCT01287975	G (High Risk)	In the trial registry record, the authors outlined two primary outcomes:<br><br>1. “Action Research Arm Test”<br>2. “Fugl Myer Upper Limb Assessment<br><br>At publication, outcome [2] was fully reported. Outcome [1] was not reported.<br><br>Given that the “Action Research Arm Test” is a faster test to administer in comparison to the “Fugl Myer Upper Limb Assessment” and the absence of any justification on why outcome [1] was not mentioned at publication, a “G” classification was attributed.
Brunner (2012)	NCT00851123	H (Low Risk)	In the trial registry record, the authors outlined the following primary outcome:<br><br>1. Action Research Arm Test [Time Frame: 3 years]<br><br>At publication, the time frame for outcome [1] was changed to 3 months.<br><br>As there is not sufficient evidence to conclude whether results were measured at the 3-year timepoint, a H classification was attributed.
Chen (2014)	NCT00950521	H (Low Risk)	In the trial registry record, the authors outlined the following primary outcome:

			1. "NIH-stroke scale (NIHSS) [Time Frame: 1, 2, 4, 12 weeks, and confirmed at 6 and 12 months]<br><br>At publication, data was fully reported for outcome [1] at baseline, 6 months, and 12 months. The remaining timepoints were not reported.<br><br>As there is not sufficient evidence to conclude whether measurements were taken during the remaining timepoints, a H classification was attributed.
Cowles (2012)	ISRCTN51553998	G (High Risk)	In the trial registry record, the authors outlined four primary outcomes:<br><br>1. "Ability to produce voluntary contraction of paretic muscle, as measured by the Motricity Index arm section";<br>2. "Ability to produce force in paretic muscle, as measured by torque about the elbow joint during isometric flexion concentric contraction using a digital myometer and maximum pinch and grip force during isometric concentric contraction using a digital pinch/grip analyser";<br>3. "Ability to use the paretic upper limb in functional activity as measured by the Action Research Arm Test (ARAT)";<br>4. "Adverse event monitoring and recording"<br><br>Outcome [1] was fully reported; outcome [3] was fully reported and downgraded to a secondary outcome; outcome [4] was fully reported but no clarification was

			given as to whether it was a primary or secondary outcome.<br><br>Outcome [2] was not reported. There was no mention of torque in the final paper. However, under the Methods section, the authors stated participant entry criteria to include the following: "*able to produce some movement in a substantially paretic upper limb as assessed by a score of at least 18 on the Motricity Index (MI) but unable to produce a grip force of more than 65% of that of the ipsilesional side";*<br><br>As grip force was clearly measured and movement of the upper limb was assessed, we assume it was likely that torque about the elbow joint was measured. Hence, a G classification was attributed.
da Cunha (2002)	NCT00037895	H (Low Risk)	In the trial registry record, the authors outlined four objectives:<br><br>1. Gait performance<br>2. Functional outcomes, as defined by the Functional Independence Measure (FIM)<br>3. Oxygen consumption during a seated exercise task<br>4. Explore whether neurophysiological characteristics using the Brain Motor Control Assessment (BMCA) predict rehabilitation outcomes<br><br>Outcome [1] was fully reported. Outcome [2] was fully reported; however, the metric used to assess functional outcomes was modified from the Function Independence Measure to the Functional Ambulation Category Scale.

			Outcome [3] was modified. Oxygen consumption was assessed during mobile exercise tasks, such as walking but not measured during a seated exercise task as specified in the trial registry record.<br><br>Outcome [4] was not reported.<br><br>Given the modifications to the assessment measures of outcomes [1-2], and the absence of evidence of whether measurements for the BMCA for outcome [4] were taken and not recorded, a H classification was attributed.
Harwood (2012)	ACTRN12606000017527	A (High Risk)	In the trial registry record, the authors outlined the following primary outcome:<br><br>1. "Short form 36 physical component"<br><br>Outcome [1] was to be measured at 6 months and 12 months after stroke. At publication, only measurements for the 12-month timepoint were reported.<br><br>In addition, actual data points were not provided. Instead, it was only reported that the result was non-significant. Hence, an A classification was attributed.
Holmgren (2010)	NCT00377689	G (High Risk)	In the trial registry record, the authors outlined the following primary outcome:<br><br>1. Improvement in balance<br><br>Outcome [1] was not reported at publication. Instead, two new outcomes were added:<br><br>a. Health-related quality of life, measured using the Short Form-36<br>b. Geriatric Depression Scale-15

			As outcome [1] was the sole primary outcome defined in the trial registry record and two new primary outcomes were added at publication, we believe it is likely that there is a risk of ORB present. However, as there is not sufficient evidence to clearly determine whether outcome [1] was measured, a G classification was attributed.
Hornnes (2011)	NCT00253097	H (Low Risk)	In the trial registry record, the authors outlined the following primary outcome:<br><br>1. "blood pressure lowering at 12 and 24 months"<br><br>At publication, data for outcome [1] was fully reported at the 12-month timepoint; however, no data was presented for the 24-month timepoint.<br><br>As there is not sufficient evidence to suggest measurements at a 24-month timepoint were taken, a H classification was attributed.
Immink (2014)	ACTRN12609000666224	I (No Risk)	In the trial registry record, the authors outlined four primary outcomes:<br><br>1. Fine motor coordination assessed by the 9 hole peg test<br>2. Functional capacity assessed with the 2 minute walk test<br>3. State and dynamic postural balance assessed with the Berg Balance Scale<br>4. Everyday motor function assessed with the Motor Assessment Scale<br><br>Outcomes [2], [3], and 4 were fully reported. A new outcome, "The Comfortable Gait Speed Test" was added at publication.

			Outcome [1] was not reported; however, justification was given as a substantial proportion of participants from the control (27%) and intervention group (55%) were unable to participate in the 9 hole peg test.<br><br>Hence, an I classification was attributed.
Lund (2011)	NCT00495248	H (Low Risk)	In the trial registry record, the authors outlined the following primary outcome:<br><br>1. "SF-36 [Time Frame: Approximately 3 months after stroke-BASELINE, after 6 and 9 months]"<br><br>At publication, data for outcome [1] was fully reported at baseline and at 9-months follow-up. Data for the remaining timepoints were not reported.<br><br>As there is not sufficient evidence to conclude whether data was measured for the earlier timepoints, a H classification was attributed.
Palmer (2012)	ISRCTN91534629	G (High Risk)	In the trial registry record, the authors outline the following primary outcome:<br><br>1. Recruitment rate, measured at baseline, 5 months and 8 months<br><br>At publication, outcome [1] was partially reported. Results at baseline and 5 months were fully reported; however, results for the 8-month timepoint were not reported.<br><br>Results for other secondary outcomes was reported at 8-months. Therefore, we believe there is a risk that ORB may be present. Hence, a G classification was attributed.

Spielmann (2016)	NTR4364	A<br>(High Risk)	In the trial registry record, the authors outlined the following primary outcome:<br><br>1. “language functioning i.e. word finding (Boston naming test)<br><br>Outcome [1] was partially reported at publication. Actual data points were not provided. Instead, it was only reported that the results were not significant. Hence, an A classification was attributed.
Wu (2012)	NCT01525979	H<br>(Low Risk)	In the trial registry record, four primary outcomes were outlined:<br><br>1. Movement<br>2. Motor units<br>3. Elbow extension angle<br>4. Lateral index<br><br>The outcome measures, at publication, are outlined as:<br><br>a) Kinematic analyses – appears to encompass outcomes [1]-[3].<br>b) Fugl Myer Assessment – appears to be upgraded<br>c) Motor Activity Log – is newly added<br>d) Stroke Impact Scale – appears to be upgraded<br><br>It is unclear in the final publication which outcomes are strictly classified as primary outcomes.<br><br>Data for outcomes [1], [2], and [3] were fully reported. It should be noted that outcome [2] seems to be presented as “Normalised Movement Units” rather than “Motor Units”; however, the data is still fully presented.<br><br>Outcome [4] was not reported. The “Lateral Index” was described as a calculation to provide an estimation of

	hemispheric activation and would be determined from fMRI scans.<br><br>As there is no clear evidence as to whether this data outcome was measured, a H classification was attributed.

References to RCTs given ORBIT Classifications

Allison (2007)

Allison R, Dennett R. Pilot randomized controlled trial to assess the impact of additional supported standing practice on functional ability post stroke. *Clinical Rehabilitation* 2007; 21: 614–619.

Ang (2014)

Ang KK, Guan C, Phua KS, et al. Brain-computer interface-based robotic end effector system for wrist and hand rehabilitation: results of a three-armed randomized controlled trial for chronic stroke. *Frontiers in Neuroengineering* 2014; 7: 30.

Brunner (2012)
Brunner IC, Skouen JS, Strand LI. Is modified constraint-induced movement therapy more effective than bimanual training in improving arm motor function in the subacute phase post stroke? A randomized controlled trial. *Clinical rehabilitation* 2012; 26: 1078–1086.

Chen (2014)
Chen D-C, Lin S-Z, Fan J-R, et al. Intracerebral Implantation of Autologous Peripheral Blood Stem Cells in Stroke Patients: A Randomized Phase II Study: *http://dx.doi.org/103727/096368914X678562* 2014; 23: 1599–1612.

Cowles (2012)
Cowles T, Clark A, Mares K, et al. Observation-to-Imitate Plus Practice Could Add Little to Physical Therapy Benefits Within 31 Days of Stroke: Translational Randomized Controlled Trial. *http://dx.doi.org/101177/1545968312452470* 2012; 27: 173–182.

da Cunha (2002)
da Cunha IT, Lim PA, Qureshy H, et al. Gait outcomes after acute stroke rehabilitation with supported treadmill ambulation training: A randomized controlled pilot study. *Archives of Physical Medicine and Rehabilitation* 2002; 83: 1258–1265.

Harwood (2012)
Harwood M, Weatherall M, Talemaitoga A, et al. Taking charge after stroke: Promoting self-directed rehabilitation to improve quality of life-a randomized controlled trial. *Clinical Rehabilitation* 2012; 26: 493–501.

Holmgren (2010)
Holmgren E, Gosman-Hedström G, Lindström B, et al. What is the benefit of a high-intensive exercise program on health-related quality of life and depression after stroke? A randomized controlled trial. *Advances in Physiotherapy* 2010; 12: 125–133.

Hornnes (2011)
Hornnes N, Larsen K, Boysen G. Blood Pressure 1 Year after Stroke: The Need to Optimize Secondary Prevention. *Journal of Stroke and Cerebrovascular Diseases* 2011; 20: 16–23.

Immink (2014)
Immink MA, Hillier S, Petkov J. Randomized Controlled Trial of Yoga for Chronic Poststroke Hemiparesis: Motor Function, Mental Health, and Quality of Life Outcomes. *https://doi.org/101310/tsr2103-256* 2014; 21: 256–271.

Lund (2011)

Lund A, Michelet M, Sandvik L, et al. A lifestyle intervention as supplement to a physical activity programme in rehabilitation after stroke: a randomized controlled trial: *http://dx.doi.org/101177/0269215511429473* 2011; 26: 502–512.

Palmer (2012)
Palmer R, Enderby P, Cooper C, et al. Computer therapy compared with usual care for people with long-standing aphasia poststroke: A pilot randomized controlled trial. *Stroke* 2012; 43: 1904–1911.

Spielmann (2016)
Spielmann K, van de Sandt-Koenderman WME, Heijenbrok-Kal MH, et al. Transcranial direct current stimulation in post-stroke sub-acute aphasia: Study protocol for a randomized controlled trial. *Trials* 2016; 17: 380.

Wu (2012)
Wu C, Yang C, Chuang L, et al. Effect of Therapist-Based Versus Robot-Assisted Bilateral Arm Training on Motor Control, Functional Performance, and Quality of Life After Chronic Stroke: A Clinical Trial. *Physical Therapy* 2012; 92: 1006–1016.
