# Supplementary material for: Selective outcome reporting in randomised controlled trials including participants with stroke or transient ischaemic attack: A systematic review
Source: Eur Stroke J. 2023 Aug 22;8(4):923–31. doi: 10.1177/23969873231194811 (PMC10683727; doi:10.1177/23969873231194811)
Supplement: sj-pdf-5-eso-10.1177_23969873231194811 – Supplemental material for Selective outcome reporting in randomised controlled trials including participants with stroke or transient ischaemic attack: A systematic review [file sj-pdf-5-eso-10.1177_23969873231194811.pdf]

CHARACTERISTICS & RISK OF BIAS OF INCLUDED STUDIES

Aben (2013)

Characteristics[1]	
Methods	Multi-site RCT<br><br>Participants block randomised (10 per site) by randomisation programme to intervention group (I) or control group (C; attention control)<br><br>Outcomes assessed at 4 points: 3 weeks prior to intervention (T0), 10 days after last intervention session (T1), 6 months post baseline (T2) and 12 months post baseline (T3)
Participants	The Netherlands<br><br>Community<br><br>Memory impairment assessed using subjective interviews to identify memory complaints<br><br>Method of stroke diagnosis not stated, but study recruited from 2 rehabilitation centres<br><br>At randomisation n = 153<br><br>I: n = 77<br><br>Mean age: 58.3 (10.36), 33/44 (female/male)<br><br>Mean time (months) since onset 52.41 (39.38)<br><br>42 LH, 35 RH; 54 ischaemic, 23 not stated<br><br>C: n = 76<br><br>Mean age 57.86 (9.00), 36/40 (female/male)<br><br>Mean time (months) since onset 55.34 (35.08)<br><br>45 LH, 31 RH; 50 ischaemic, 26 not stated<br><br>Lost to follow-up: at T1 n = 10 (5 I, 5 C; sample n = 72 I, 71 C) with ITT analysis, at T2 n = 1 (1 I, 1 C participants previously lost returned with measures; sample n = 71 I, 72 C), T3 n = 4 (4 I, 0 C, sample n = 67 I, 72 C)
Interventions	Compensatory approach, plus psycho-education and CBT aspects<br><br>I: 9 x 1-hour sessions/twice weekly (9 hours total). Groups of 4 to 6. Memory Self Efficacy training programme covering: 1) theoretical introduction on memory and stroke, 2) training on internal and external memory strategies to improve compensation, 3) psycho-education on effect of mood, anxiety and memory-related worries on memory complaints. Training booklet provided plus half-hour homework per session

	C: 9 x 1-hour sessions/twice weekly (9 hours total). Groups of 4 to 6. Peer support groups, no therapeutic intervention. Education on causes and consequences of stroke; sharing of problems. Hand-outs provided, but no homework assigned
Outcomes	Subjective memory measure: Metamemory In Adulthood questionnaire<br><br>Objective memory measure: RBMT story recall delayed<br><br>Mood measure: CES-D<br><br>Quality of life measure: EQ-5D Utility<br><br>Other outcomes assessed:<br><br>Health-related quality of life (WHOQoL-Bref; EQ-5D - VAS), Memory (AVLT), Social Support List, problem experience (Impact on Participation and Autonomy
Notes	Inclusion criteria: > 18 month post-onset of first and only stroke, 18 to 80 years old, living independently, reported subjective memory complaints (via interview)<br><br>Exclusion criteria: progressive neurological disorder (e.g. dementia or MS), insufficient knowledge and comprehension of Dutch, alcohol/drug abuse, subdural haematoma or subarachnoid haemorrhage<br><br>Adequate matching: demographics and baseline characteristics non-significant on most measures<br><br>Method of diagnosing stroke: not stated, but recruited from 2 rehabilitation centres<br><br>Coping, personality traits, aphasia and executive functioning measures were taken at baseline only<br><br>Statistical tests used: independent t-tests for continuous variables and $\chi 2$ tests for categorical variables using baseline data for MSE and z-scores for AVLT and RBMT<br><br>Short-term follow-up data also presented in Aben 2013 paper, which used an ITT analysis on T1 results

Risk of Bias[1]

Bias	Author's Judgment	Support for Judgement
Random sequence generation	Low risk	Randomisation conducted by someone independent to the trial, using a randomisation program
Allocation concealment	Low risk	"Allocation of the group to either the experimental or control condition was performed by an independent investigator

		who was not involved in the study"
Blinding of participants and personnel	High risk	"Patients were not informed on the type of intervention they had been allocated to". Those delivering intervention were aware
Blinding of outcome assessment	Low risk	"All patients were assessed […] by trained research psychologists who were blinded to group allocation. The data were entered […] by the blinded research psychologists."
Incomplete outcome data	Low risk	Missing outcome data explained and equal in both groups
Selective reporting	High risk	No protocol available to cross-check. However, 2 additional published papers show same outcome measures with equal participants in each. From the EQ-5D only the VAS score was reported, but not the Utility score
Other bias	Low risk	

Algra (2007)

Characteristics[2]	
Methods	Randomised trial<br>Concealment by telephone, computer randomised<br>Blinding: outcome assessment only<br>Results available for 96.9%<br>Intention-to-treat analysis
Participants	Europe, Australia, Asia<br>1089 patients<br>Mean age 61 years<br>67% male<br>Cerebral ischaemia of non-cardiac origin or transient monocular blindness<br>CT in all but 72 (93.3%)<br>Time since stroke: less than 6 months<br>Comparability of groups: no major differences for prognostic variables
Interventions	Rx: phenprocoumon or acenocoumarol or warfarin (INR 2.0 to 3.0)<br>Monitoring: on regular basis check of INR level<br>Compliance: specified in table, stopped in 198 patients (37%), 70% of time within target range<br>Control: aspirin 30 mg (57%), 75 mg (15%), 80 mg (6%), 100 mg (12%), 150 mg (3%), 200 mg (6%)

	Monitoring: no monitoring<br>Compliance: stopped in 84 (15%)
Outcomes	Vascular death, non-fatal stroke, non-fatal myocardial infarction, major bleeding complication
Notes	Exclusion criteria: Rankin grade > 3, (contra) indication for study medication<br>Follow-up: mean 4.6 years<br>Trial stopped early (after 55% of planned patients years) because of new standard therapy of aspirin plus dipyridamole<br>34 patients were lost, 7 had incomplete follow-up

Risk of Bias[2]

Bias	Author's Judgment	Support for Judgement
Random sequence generation	Low risk	Computer generated
Allocation concealment	Low risk	Adequate
Blinding of participants and personnel	High risk	Open label study
Blinding of outcome assessment	Low risk	Blinded outcome assessment
Incomplete outcome data		
Selective reporting		
Other bias		

Allison (2007)

Characteristics[3]

Methods	Study design: RCT<br>Method of randomisation: "Randomization was achieved via the use of 20 sealed envelopes, 10 of each containing a specific group allocation. A staff member who was blind to the study selected a sealed envelope for each participant, indicating the group allocation"
Participants	Number of participants: n = 17<br>Inclusion criteria: "Patients with a confirmed diagnosis of recent stroke were eligible for inclusion"<br>Exclusion criteria: terminally ill, suffering from an unstable co-morbidity and unable to participate safely (physically or mentally) in additional sessions of standing
Interventions	(1) Intervention group (n = 7)<br>"The second treatment group (intervention group) received the conventional treatment session, and in addition had a further session of 45 minutes standing practice on each working day. This was provided by a physiotherapy assistant and typically involved the use of either standing frames, tilt tables or standing at tables to provide support while enabling standing to occur"<br><br>"Participants progressed to standing by a table for support or free standing during rehabilitation as able. Participants were

encouraged to be active whilst standing, practising reaching tasks, sit-to-stand movements and so on, and were given rest periods as necessary throughout the 45-minute session. It was not possible for the physiotherapists providing the conventional treatment to be blind to the extra intervention occurring, due to the organization of therapy on the ward. This treatment regime was continued throughout the participants stay in the rehabilitation unit. After discharge from the unit the participant was referred for outpatient or community-based physiotherapy. Intensity of follow-up offered was based on community assessment and was typically one or two sessions of treatment per week"

The individual components delivered are listed in Table 3. Based on the individual components, this intervention is categorised as comprising functional task training and musculoskeletal intervention (active)

Length of intervention period: ranged from 14 to 28 days dependent upon length of stay on the unit

Number of sessions and length of individual sessions: "received the conventional treatment session, and in addition had a further session of 45 minutes standing practice on each working day"

Intervention provider: "conventional physiotherapy sessions were provided [by] one of three physiotherapists on the ward; the additional session of standing practice was provided by a physiotherapy assistant"

(2) Control group (n = 10)

"Control group received conventional physiotherapy treatment from one of the three physiotherapists working on the ward. This was typically a session of 45 minutes treatment on each working day, including work on strengthening, improving movement, mobility, and upper limb function"

The individual components delivered are listed in Table 3. Based on the individual components, this intervention is categorised as comprising functional task training and musculoskeletal intervention (active)
Length of intervention period: ranged from 14 to 28 days dependent upon length of stay on the unit | Number of sessions and length of individual sessions: "session of 45 minutes treatment on each working day"

Intervention provider: "conventional physiotherapy sessions were provided [by] one of 3 physiotherapists on the ward"

This study is classified as active Intervention one (functional task training, musculoskeletal (active)) versus active intervention two (functional task training, musculoskeletal (active))

Outcomes	Measures of motor function: gross functional tool section of Rivermead Motor Assessment<br>Measures of postural control and balance: Berg Balance Scale<br>Other secondary outcome measures: trunk control test<br>Time points when outcomes were assessed: week one, week two and week 12
Notes	No outcomes included in analysis (data reported as median and IQR)

Risk of Bias[3]

Bias	Author's Judgment	Support for Judgement
Random sequence generation	Low risk	"Randomization was achieved via the use of 20 sealed envelopes, 10 of each containing a specific group allocation"
Allocation concealment	Low risk	"A staff member who was blind to the study selected a sealed envelope for each participant, indicating the group allocation"
Blinding of participants and personnel		
Blinding of outcome assessment	Low risk	"The measurements were conducted by a staff member who did not work on the unit, and who was blind to the treatment allocation"
Incomplete outcome data	High risk	"Three of the participants allocated to the additional practice group withdrew from the additional treatment within the first week of the study, all of them citing fatigue as the reason they would not continue. One of these consented to further measures being taken but two withdrew totally from the study. Results were analysed on an intention to treat basis, but the two participants who only completed the first week of measures were excluded"
Selective reporting		
Other bias	High risk	"It was not possible for the physiotherapists providing the conventional treatment to be blind to the extra intervention occurring, due to the organization of therapy on the ward."

		"Fatigue may be a significant barrier to participate in more intensive programmes"

Anderson (2008)

Characteristics[4]

Methods	Open, blinded outcome, randomised trial<br><br>Randomisation done with minimisation through a password protected Internet-based system<br><br>Intention-to-treat analysis
Participants	International, multicentre<br>404 participants, T: 203, C: 201<br>Age 63 years<br>Male 65%<br>Inclusion: spontaneous ICH confirmed by CT and elevated SBP (≥ 2 measurements of 150 to 220<br>mmHg, recorded ≥ 2 minutes apart)<br>100% CT<br>Enrolment: within 6 hours of ICH onset<br><br>FU: no losses
Interventions	T: early intensive lowering of BP (target SBP 140 mmHg)<br>C: standard guideline based management of BP (target SBP 180 mmHg)<br>Both groups received oral as well as intravenous agents for lowering BP<br>Rx: for 7 days
Outcomes	Proportional change in haematoma volume at 24 hours<br>BP methodology not stated
Notes	Exclusion: indication for intensive lowering of BP, CI to intensive lowering of BP, ICH secondary to structural cerebral abnormality or use of thrombolytic agent, IS within 30 days, deep coma (3 to 5 on the GCS), pre-stroke disability or medical illness, and early planned decompressive neurosurgical intervention

Risk of Bias[4]

Bias	Author's Judgment	Support for Judgement
Random sequence generation		
Allocation concealment	Low risk	Adequate
Blinding of participants and personnel		
Blinding of outcome assessment		
Incomplete outcome data		
Selective reporting		
Other bias		

Andrade (2017)

Characteristics[5]

Methods	Sham-controlled, double-blinded, parallel RCT
Participants	A total of 60 unilateral, non-recurring, acute ischaemic stroke patients with a high risk of falling
Interventions	In addition to the same physical rehabilitation programme (1 hour a day, 3 days a week), all participants received an additional 10 sessions (5 consecutive days for 2 weeks) of:<br>* anodal tDCS for group A (n = 15) * bilateral tDCS for group B (n = 15) * cathodal tDCS for group C (n = 15) * sham tDCS for group D (n = 15)
Outcomes	Number of fallers and adverse events
Notes	Fall registration during both intervention and follow-up period.<br>Registration time: 3 months

Risk of Bias[5]

Bias	Author's Judgment	Support for Judgement
Random sequence generation	Low risk	Quote: "Randomization was conducted with randomly permuted blocks, through an online program (www. random.org)." (page 3)
Allocation concealment	Low risk	Quote: "... blind allocation in the ratio of 1:1:1:1 was carried out with sequentially numbered and sealed opaque envelopes." (page 3)
Blinding of participants and personnel		
Blinding of outcome assessment	Low risk	Although participants recorded falls themselves through the use of a falls calendar, participants were blinded to group allocation.
Incomplete outcome data	Low risk	Incomplete outcome data adequately addressed and unlikely to seriously alter the results. | Quote: "All participants were inserted into the analysis, even if they had attended only one session, following the intention-to-treat principle. Sensitivity analysis was applied to choose the treatment of missing data....Missing data were treated by simple imputation." (page 4)
Selective reporting	Low risk	All of the studies' prespecified outcomes have

		been reported in the prespecified way
Other bias		

Ang (2014)

Characteristics[6]	
Methods	RCT
Participants	Country: Singapore<br><br>Sample size: 21 participants (7 in treatment group: brain computer interface with haptic knob device (BCI-HK); 8 in treatment group: HK; 7 in control group)<br>Inclusion criteria: first-ever stroke, confirmed by neuroimaging; age 21 to 80 years; time since stroke > 4 months; FMA-score 10 to 50 points (moderate to severe arm impairment); motor power grade 2 to 5 MRC shoulder abduction, grade 2 to 5 MRC elbow flexion, and grade 1 to 3 MRC in wrist dorsiflexion and finger flexion<br><br>Exclusion criteria: medical instability; postural hypotension; terminal illness; severe aphasia; inattention; hemispatial neglect; severe visual impairment; epilepsy; severe depression; psychiatric disorders; recurrent stroke; skull defect; severe spasticity; fixed joint contractures; skin lesions
Interventions	3 groups:<br><br>* robot-mediated therapy with the haptic knob robot and a brain computer interface for 60 minutes + therapist-assisted arm mobilisation for 30 minutes<br><br>* robot-mediated therapy with the haptic knob robot alone for 60 minutes + therapist-assisted arm mobilisation for 30 minutes<br><br>* standard arm therapy for 60 minutes + therapist-assisted arm mobilisation for 30 minutes
Outcomes	Outcomes were measured at baseline (week 0), at mid-intervention (week 3), at the end of the intervention period (week 6), 6 weeks' follow-up (week 12), and 18 weeks' follow-up (week 24)<br><br>Primary outcome: total FMA score
Notes	We combined the results of both HK groups in 1 (collapsed) group and compared this collapsed group with the results of the standard arm therapy group

Risk of Bias[6]

**Bias**	**Author's Judgment**	**Support for Judgement**
Random sequence generation	Low risk	Quote: "The randomization block size was 3 and the allocation sequence was 1:1:1 generated using software"

Allocation concealment	Unclear risk	Not described
Blinding of participants and personnel		
Blinding of outcome assessment	Low risk	Quote: "As subject blinding was not feasible, all outcome assessments for this study were performed by occupational therapist DXD who was blinded to allocation."
Incomplete outcome data		
Selective reporting	Unclear risk	Insufficient information to permit judgement
Other bias		

Barker-collo (2009)

Characteristics[7]	
Methods	RCT, parallel group design<br><br>Concealed online Internet randomisation service with stratified minimisation<br><br>Implementation of randomisation sequence by the treating clinician who had no access to assessment data. Randomisation information was not accessible by any other study staff during the study.<br><br>Approach: restoration of attentional functions with the means of APT
Participants	New Zealand, recruited from 2 hospitals<br><br>Total participant sample 78; 10 lost at 5 weeks, 12 were not assessed at 6 months<br><br>Treatment group: n = 38; mean (± SD) age 70.2 ± 15.6 years; 60.5% males; mean (± SD) 18.5 ± 12.0 days since onset; hemisphere of lesion: 14 left (44%), 15 right (47%), 3 bilateral or unclear (9%)<br><br>Control group: n = 40; mean (± SD) age 67.7 ± 15.6 years; 60.0% males; mean (± SD) 18.6 ± 7.6 days since onset; hemisphere of lesion: 25 left (58%), 17 right (40%), 1 bilateral or unclear (2%)<br><br>Inclusion criteria: attention deficit defined as performance > 1 SD below norm on any attentional tests; stroke using WHO criteria; admitted to 1 of 2 hospitals; within 2 weeks of stroke<br><br>Exclusion criteria: unable to give consent; MMSE < 20; medically unstable; unable to speak English; other relevant conditions, such as dementia

Interventions	Treatment: up to 30 hours' individual APT for 1 hour on weekdays for 4 weeks, mean (± SD) 13.5 ± 9.4 hours<br><br>Control: no treatment
Outcomes	Measured immediately after treatment (5 weeks) and at follow-up (6 months)<br><br>Primary outcome:<br>* IVA-CPT Full-Scale Attention Quotient (z-scores)<br><br>Secondary outcomes:<br>* IVA-CPT Auditory attention (z-scores)<br>* IVA-CPT Visual attention (z-scores)<br>* Bells test (omissions of left-, central, and right-sided targets)<br>* Trail Making A & B (z-scores)<br>* PASAT (z-scores for 2 and 2.4 seconds)<br>* SF-36 (Mental Component Score and Physical Component Score)<br>* Modified Rankin (raw score)*<br>* Cognitive Failures Questionnaire (raw score)*<br>* GHQ-28 (raw score)*<br>*Only measured at 6 months' follow-up
Notes	Provided numbers of side of hemisphere lesions did not add up to total participant sample. Additional data for analysis provided by authors.

Risk of Bias[7]

Bias	Author's Judgment	Support for Judgement
Random sequence generation	Low risk	Internet based and independent
Allocation concealment	Low risk	Concealed
Blinding of participants and personnel	Unclear risk	Participants and therapist not blinded as aware of intervention being given.
Blinding of outcome assessment	Low risk	Trained assessor blind to randomisation
Incomplete outcome data	Low risk	Intention-to-treat analysis Last observation carried forward
Selective reporting	Low risk	No indication in article
Other bias		

Barzel (2015)

Characteristics[8]

Methods	Design: cluster-randomised trial of home CIMT vs standard therapy<br>Study duration: 4 weeks<br><br>Randomisation: practices were stratified by region and randomly allocated by an external biometrician (1:1, block size of 4) using a computer-generated sequence.

	Allocation concealment: yes, by the computer-generated sequence. Randomisation was per practice and further allocation concealment was not necessary. Furthermore, patients were included in the study before randomisation of practices to minimise differential self-selection.<br><br>Blinding: assessors blind for group allocation; statistician was also masked.<br><br>ITT: yes
Participants	Randomised: 156 participants<br><br>Withdrawals: 5 withdrawals in the intervention group because of death, poor health, and not wanting to continue; 4 withdrawals in the control group because of moving, death, and poor health<br><br>Intervention: Home CIMT: 85 participants; 51 men and 34 women; mean age 62.55 years (SD 13.73); mean time since stroke 56.57 months (SD 47.36)<br><br>Standard therapy: 71 participants; 43 men and 28 women; mean age 65.30 years (SD 12.63); mean time since stroke 45.65 months (SD 57.69)<br><br>Inclusion criteria: physical and occupational therapy practices: treating adults with upper limb dysfunction after stroke unless they already offered CIMT, with 1 therapist with a professional qualification or at least 2 years of experience in treatment of chronic impairment caused by stroke; patients: > 6 months after stroke, mild-to-moderate impairment of arm function and minimal residual hand function (minimum 10° active wrist extension, 10° active thumb abduction or extension, and 10° extension of 2 additional fingers), had a referral for physical or occupational therapy, > 18 years, had a caregiver who was prepared to be a non-professional coach (e.g. family member).<br><br>Exclusion criteria: severely impaired verbal communication, inability to give consent, severe neurocognitive deficits (score < 23 in the Mini-Mental State Examination), terminal illness, or life-threatening comorbidities, or previously received CIMT.
Interventions	Intervention: home CIMT: patients were instructed to train in their home environment for 2 hours each day, accompanied by a coach. Additionally, patients were asked to wear a resting glove during exercises and ADL to immobilise their non-affected hand. The therapists guided the coach on how to document the time or repetitions per time for each exercise and to assist the patient in keeping a training diary. Therapists used the first of 5 home visits to instruct the patient and the coach in the principles of home CIMT, set individually tailored goals, and work through the first 2 to 3 exercises, focusing on everyday practice. During subsequent weekly home visits, therapists supervised the training, set up new exercises, and applied behavioural techniques. Professional therapy time was not used to practise exercises.<br><br>Control: conventional physical or occupational therapy, but additional home training was not obligatory. Standard therapy could consist of various therapeutic techniques typical of stroke therapy. The standard

	therapy group therapists reported details of professional treatment delivery and any agreements (e.g. homework) made with patients via a standardised documentation sheet.<br><br>Setting: intervention group - home; control group - therapy practice
Outcomes	Included outcomes: Motor Activity Log - quality of movement, Wolf Motor Function test - performance time, Motor Activity Log - amount of use, Wolf Motor Function test - functional ability, Nine Hole Peg Test, SIS hand function, Barthel index, IADL<br><br>Measurements: baseline assessment, post intervention assessment after 4 weeks, follow-up assessment at 6 months. Interim interview (Motor Activity Log) at 3-month follow-up
Notes	For mean changes of outcomes means and 95% confidence intervals were given. To calculate SDs, we used the Z-score (1.96).

Risk of Bias[8]

Bias	Author's Judgment	Support for Judgement
Random sequence generation	Low risk	Practices were stratified and randomly allocated by an external biometrician using a computer-generated sequence.
Allocation concealment	Low risk	By computer-generated sequence. Furthermore, patients were included in the study before randomisation of practices to minimise differential self-selection.
Blinding of participants and personnel	High risk	Participants and personnel cannot be blind for the intervention.
Blinding of outcome assessment	Low risk	Outcome assessors and the statistician were masked.
Incomplete outcome data	Low risk	5 withdrawals in intervention group and 4 in the control group; well described and for similar same reasons.<br><br>Missing data were imputed using correct methods; analyses were by ITT and in case of missing values, a last observation carried forward imputation was performed.
Selective reporting	Low risk	The study protocol is available and preselected outcomes are in the review. There are some minor differences: EQ-5D, costs

		and SIS are not described in this paper.
Other bias	Low risk	

Bath (2015)

Characteristics[9]	
Methods	Single-blind, parallel-group, partial factorial study<br><br>Randomisation via password-protected, data-encrypted website (minimisation by age, sex, stroke severity, time to treatment and total anterior circulation syndrome)
Participants	International (23 countries), multicentre (173 sites)<br><br>4011 participants: treatment 2000; control 2011<br><br>Age: treatment 70 years; control 70 years<br><br>Male: treatment 1147 (57%); control 1150 (57%)<br><br>Inclusion: IS or ICH; motor deficit in arm or leg, or both; systolic BP 140 to 220 mmHg<br><br>Time to randomisation: within 48 hours of onset
Interventions	• Treatment: transdermal GTN patch 5 mg once daily<br>• Control: no GTN (blinding with gauze dressing over patch/equivalent area of skin)<br><br>In appropriate participants:<br>• Treatment: continue pre-stroke antihypertensive medication<br>• Control: stop pre-stroke antihypertensive medication<br><br>Duration: 7 days
Outcomes	Primary: mRS at day 90<br><br>Secondary:<br>• days 1 to 7: BP and HR<br>• day 7: recurrent stroke<br>• discharge: length of hospital stay; disposition<br>• day 90: death or dependency (mRS > 2); BI; EQ-5D, EQ-VAS; MMSE, TICS-M; animal naming; Zung Depression Scale
Notes	Exclusions: GCS < 8; pure sensory stroke; preceding dependency (mRS 3 to 5); confounding neurological or psychiatric illness; stroke mimic; severe liver or renal dysfunction; severe comorbidity; pregnant or breastfeeding; planned surgical intervention; previous participation in ENOS 2015; contraindication to or definite need for nitrates and/or prestroke antihypertensive medication

Risk of Bias[9]

**Bias**	**Author's Judgment**	**Support for Judgement**

Random sequence generation	Low risk	Central computer randomisation with minimisation
Allocation concealment	Low risk	Central website-based
Blinding of participants and personnel	Unclear risk	GTN was given single-blinded as no placebo patches were available. A gauze dressing was placed over the GTN patch or equivalent area of skin out of sight. As a result, participants were blinded whilst the treating clinician was unblinded
Blinding of outcome assessment	Low risk	Outcomes assessed centrally, blinded to treatment group
Incomplete outcome data	Low risk	No difference between trial groups
Selective reporting	Low risk	All prespecified outcomes reported
Other bias	Low risk	None found

Benavente (2011)

Characteristics[10]	
Methods	RCT<br><br>Unit of randomisation: participant
Participants	Place of recruitment: not documented<br><br>Numbers randomised: total: 3020 (I: 1501; C: 1519)<br><br>% Completing final follow-up: 98%<br><br>Inclusion criteria: lacunar stroke syndrome confirmed by MRI, > 30 years old, normotensive and hypertensive patients<br><br>Exclusion criteria: no surgical amenable ipsilateral carotid artery disease, no major risk cardio-embolic sources<br><br>Type of stroke (%): small subcortical stroke (100%)<br><br>Mean age: 63 +/- 11 years<br><br>Gender (men): 63%<br><br>Ethnicity: white (51%), Hispanic (30%), black (19%)<br><br>Socio-economic or socio-demographic status: USA (56%), Latin America (23%), Spain (12%) Canada (9%)

Interventions	Intervention details (components, length, frequency): participants were randomised to 1 or 2 levels of BP control either 'intensive' (< 130 mmHg) or 'usual' (130–149 mmHg). Also participants were randomly assigned to take clopidogrel 75 mg daily or the matching placebo<br><br>Location: outpatient clinic<br><br>Mode of delivery: outpatient clinic face to face, free prescriptions were given<br><br>Personnel responsible for delivery: physicians<br><br>Timing post-stroke: 6 months or less<br><br>Control: usual care - including standard (< 140 mmHg) blood pressure control
Outcomes	3 years: time to first stroke relapse; stroke relapse rate; proportion of participants meeting targets for blood pressure, blood fats, blood sugar and BMI
Notes	Funding: this research was funded by the National Institute of Neurological Disorders and Stroke (NINDS # 2 U01 NS38529-04A1)<br><br>Country of origin: USA<br><br>Publication language: English<br><br>Analysis method: analysis of variance<br><br>Risk of bias: unclear

Risk of Bias[10]

Bias	Author's Judgment	Support for Judgement
Random sequence generation	Low risk	Randomised using a 2 x 2 factorial design stratified by clinical centre and baseline hypertensive stats. Data was inputted and a computer generated unique number was given to assign participants
Allocation concealment	Unclear risk	Insufficient information
Blinding of participants and personnel		
Blinding of outcome assessment		
Incomplete outcome data	Unclear risk	Not addressed
Selective reporting	Low risk	Study protocol is available in a previous publication
Other bias	Low risk	The study appears to be free of other sources of bias

Bethoux (2014)

Characteristics[11]	
Methods	Study design: RCT<br><br>Instruments used: MMSE, BDI, 10MWT, SIS, device-related SAE rate, 6MWT, GaitRite FAP, mEFAP, BBS, TUG, SSQoL<br><br>Study design as described in the article: Quote: "This study was an unblinded, parallel-group RCT"<br><br>Study duration: 24 months<br><br>Year of study: trial ran between April 2010 and April 2012
Participants	Inclusion criteria: ≥ 6 months poststroke; inadequate dorsiflexion with inadequate limb clearance during swing phase of gait; positive response to peroneal nerve stimulation testing; adequate cognitive function (MMSE score > 17); not currently using FES for the treatment of foot drop; ≥ 30 days post-inpatient or outpatient stroke, cardiac, pulmonary, or any other lower extremity physical rehabilitation; able to walk at least 10 meters with or without an assist device; initial gait speed of > 0.0 m/s and < 0.8 m/s; eligible for Medicare or Medicare Choice/Advantage benefits at time of consent; ≥ 90 days post-MI; ≥ 90 days post-stenting procedure (i.e. peripheral, cardiac, carotid, and/or renal); ≥ 90 days post-major orthopedic surgery (i.e. hip, knee, and/or ankle joint replacement); ≥ 6 months post-CABG or cardiac valve procedure; able and willing to give written consent and comply with study procedures, including follow-up visits<br><br>Exclusion criteria: ankle joint instability other than foot drop; needs AFO for stance control of the foot, ankle, and/or knee; unable to safely clear toes in swing phase on the involved lower extremity, defined as > −5 degrees plantar flexion with the WalkAide device (determined at fitting); diagnosed with peripheral neuropathy, and symptoms obstruct or limit ambulation or participation in study; diagnosed with significant peripheral vascular disease accompanied by lower extremity ulceration and/or disabling claudication; underlying condition(s) that would limit study participation; severe hypertonicity resulting in the need for more involved orthotic strategies; excessive dysesthetic pain secondary to neurological involvement; moderate to very severe chronic obstructive pulmonary disease, as defined by the Global Initiative for Chronic Obstructive Lung Disease (GOLD); New York Heart Association (NYHA) Class III-IV; malignant skin lesion below the knee on the affected lower extremity; history of seizure disorder and is currently on seizure control medication for this disorder; aphasia, defined as inability to verbalize commands; BDI score of > 29 indicating severe depression; life expectancy less than 12 months; received botulinum toxin injections in the lower extremity within the past 6 months; baclofen pump with unstable dosing in the last 3 months; participating in another clinical trial that, according to the principal investigator, is likely to affect study outcome or confound results; patient has existing electrical stimulation devices (implantable cardioverter defibrillator, pacemaker, spinal stimulation, TENS)

	Age: MN group mean age (± SD): 63.87 years (± 11.33); control group mean age (± SD): 64.30 years (± 12.01)<br><br>Country: USA<br><br>Sample size: 495 participants<br><br>Sex: MN group: 147 (60.74%) men, 95 (39.26%) women; control group: 157 (62.06%) men, 96 (37.94%) women<br><br>Time poststroke: ≥ 6 months poststroke. MN group mean time poststroke (± SD): 6.90 years (± 6.43); control group mean time poststroke (± SD): 6.86 years (± 6.64)<br><br>Type of stroke: not stated
Interventions	Motor neuroprosthesis<br><br>• Intervention: MN group used WalkAide device for all walking activities on a full-time basis throughout the day. In the first 2 weeks, participants adhered to a progressive wearing schedule, after that they were instructed to wear MN on a full-time basis (i.e. for all walking activities throughout the day).<br>• Number of participants: 242<br>• Device: a single-channel electrical stimulator composed of a cuK worn around the proximal part ofthe lower leg, control module, and surface electrodes. This device uses a tilt sensor and accelerometer to trigger ankle dorsiflexion during the swing phase of gait.<br>• Duration of exposure: the length of treatment with MN was 12 months<br>• Place of application of intervention: lower limb<br><br>Another assistive technology device<br><br>• Intervention: control group used AFO for all walking activities on a full-time basis throughout the day. In the first 2 weeks, participants adhered to a progressive wearing schedule, after that they were instructed to wear AFO on a full-time basis (i.e. for all walking activities throughout the day).<br>• Number of participants: 253<br>• Device: AFO could be either articulated or fixed at the ankle based on the professional opinion of the orthotist and clinical needs of the participant<br>• Duration of exposure: the length of treatment with AFO was 12 months<br>• Place of application of intervention: lower limb
Outcomes	Activities involving limbs: walking speed measured with the 10MWT (m/s)<br><br>• Outcome type: continuous<br>• Assessment time point: baseline, 6 months, and 12 months<br>• Device at assessments: baseline, 6-month, and 12-month assessments performed with MN<br><br>Activities involving limbs: mEFAP (s)<br><br>• Outcome type: continuous • Assessment time point: baseline, 6 months, and 12 months

	• Device at assessments: baseline, 6-month, and 12-month assessments performed with MN Activities involving limbs: TUG (s)<br>• Outcome type: continuous<br>• Assessment time point: baseline and 6 months<br>• Device at assessments: baseline, 6-month, and 12-month assessments performed with MN<br><br>Balance: BBS<br>• Outcome type: continuous<br>• Assessment time point: baseline and 6 months<br>• Device at assessments: baseline, 6-month, and 12-month assessments performed with MN<br><br>Exercise capacity: 6MWT (m)<br>• Outcome type: continuous<br>• Assessment time point: baseline, 6 months, and 12 months<br>• Device at assessments: baseline, 6-month, and 12-month assessments performed with MN<br><br>Participation scale of HRQoL: SSQoL<br>• Outcome type: continuous<br>• Assessment time point: baseline and 6 months<br><br>Participation scale of HRQoL: SIS Social participation domain<br>• Outcome type: continuous<br>• Assessment time point: baseline and 6 months<br><br>Adverse events: dropouts during the intervention period<br>• Outcome type: binary<br><br>Adverse events: serious adverse events related to the intervention<br>• Outcome type: binary<br><br>Adverse events: falls<br>• Outcome type: binary
Notes	This study consisted of 2 articles (Bethoux 2014; Bethoux 2015).

Risk of Bias[11]

Bias	Author's Judgment	Support for Judgement
Random sequence generation	Low risk	Quote: "Using a centralized computer-generated randomization scheme built into the electronic data capture system for this study"
Allocation concealment	Low risk	Quote: "Centralized computer-generated randomization scheme"
Blinding of participants and personnel	High risk	There was no blinding of participants and personnel.
Blinding of outcome assessment	High risk	Unblinded outcome assessment

Incomplete outcome data	Low risk	Quote: "We conducted an ITT analysis using multiple imputations to account for missing data"
Selective reporting	Unclear risk	Although all of the study's prespecified primary outcomes were reported, a secondary variable was included in the study that was not prespecified in the protocol.
Other bias	High risk	This study was sponsored by Innovative Neurotronics.

Bowen (2012)

Characteristics[12]	
Methods	Multicentre RCT stratified by severity of communication impairment and recruiting site, UK
Participants	Inclusion criteria: communication impairment as a result of aphasia, therapist considers able to engage in therapy and likely to benefit, consent<br><br>Exclusion criteria: subarachnoid haemorrhage, dementia, learning disabilities, non-English speaker, serious comorbidity, unable to complete screening procedure within 3 attempts or 2 weeks, family or caregiver objection, therapist assessment required prior to trial screening<br><br>Group 1: 76 participants<br><br>Group 2: 77 participants<br><br>Details of participants are shown in Table 1
Interventions	1. Conventional SLT<br><br>Intervention: speech and language therapy.<br><br>Materials: communication charts, personalised advice booklet, session record, patient life book, AAC devices.<br><br>Procedures: manualised (assessment, information provision, provision of communication materials, caregiver contact, indirect contact (with MDT), direct contact). Direct remediation of speech and language: impairment (hypothesis-driven approach to rehabilitation of language skills), activity (compensatory strategies and conversational skills training), and participation (specific exercises) approaches. Promotion of alternative means of communication, support adjustment to communication impairment, improving communication environment.<br><br>Provided by: 4 therapists. Led by highly experienced speech and language therapists plus delivery by other therapists.

	Delivery: 1-to-1, face-to-face, clinic or home.<br><br>Regimen: Per protocol. 3 sessions (varied length) weekly up to 16 weeks. Delivered average of 22 sessions (18 h) over 13 weeks.<br><br>Tailoring: individualised.<br><br>Modification: therapy amount.<br><br>Adherence: monitored.<br><br>2. Social support and stimulation<br><br>Intervention: 9 part-time paid trained visitors. Attention control.<br><br>Materials: approved board games and activities.<br><br>Procedures: manualised. Participant-led. Everyday activities building rapport including general conversation and activities (reading to the participant, watching television, playing board games (e.g. chess), creative activities, gardening) TV, music. Plus sessions to prepare participants for cessation of visits.<br><br>Provided by: trained paid visitors.<br><br>Delivery: 1-to-1, face-to-face, hospital and at home.<br><br>Regimen: Per protocol up to 3 sessions (varied length up to 60 mins) weekly for 16 weeks. Delivered max 45 sessions (average 15 h; 1-45 contacts, max 41 h) up to 16 weeks.<br><br>Tailoring: yes. Individualised.<br><br>Modification: amount of visits (above).<br><br>Adherence: monitored
Outcomes	Primary outcomes: functional communication; expert blinded therapist rating of semi-structured conversation using TOMs<br><br>Secondary outcomes: participant and caregivers' own perception of functional communication and quality of life, costs of communication therapy compared with that of attention control<br><br>Data collection: baseline and 6 months postrandomisation
Notes	Additional participants with dysarthria (no aphasia) were also randomised to the 2 interventions, but data from these individuals have not been included within this review<br><br>Dropouts are detailed in Table 2<br><br>Statistical data included within the review meta-analyses

Risk of Bias[12]

**Bias**	**Author's Judgment**	**Support for Judgement**
Random sequence generation	Low risk	External, independent, web-based, stratified by severity of communication impairment (TOM) and recruiting site
Allocation concealment	Low risk	External, independent, web-based
Blinding of participants and personnel		
Blinding of outcome assessment	Low risk	Primary outcome rated by expert therapists blinded to allocation<br>Other measures collected by research staff where all attempts to maintain blinding were taken
Incomplete outcome data	Low risk	Dropouts accounted for<br><br>ITT employed
Selective reporting	Low risk	All prespecified outcomes reported
Other bias	Low risk	Groups comparable at baseline<br><br>Sample size calculation reported

Brokaw (2013)

Characteristics[6]

Methods	Randomised cross-over trial
Participants	Country: USA<br><br>Sample size: 12 participants<br><br>Inclusion criteria: adult with ischaemic/haemorrhagic stroke at least 6 months before; persistent hemiparesis (score 1 to 2 on the National Institutes of Health Stroke Scale); voluntary wrist and finger extension; shoulder elevation<br><br>Exclusion criteria: a score of less than 24 on the MMSE; hemispatial neglect; severe sensory loss; excessive pain in any joint of the affected hemisphere or upper extremity injury
Interventions	2 groups:

	1. group AB: 12 hours of robotic training within a month (A) and 12 hours of conventional therapy within a month (B), separated by a month of wash-out period<br><br>2. group BA: 12 hours of conventional therapy within a month (B) and 12 hours of robotic training within a month (A), separated by a month of wash-out period
Outcomes	FMA<br><br>ARAT<br><br>BBT
Notes	

Risk of Bias[6]

Bias	Author's Judgment	Support for Judgement
Random sequence generation	Low risk	Quote: "Randomization was done using a random number generator function in Matlab (MathWorks Inc, Natick, MA) that generated a list of numbers (1-10) randomly ordered
Allocation concealment	Unclear risk	Quote: "The first 5 listed subject numbers received conventional therapy first and the second set received robot therapy first."
Blinding of participants and personnel		
Blinding of outcome assessment	Low risk	Quote: "The OT performing recruitment and clinical evaluations was not aware of the randomization order, so was blinded to group assignment."
Incomplete outcome data		
Selective reporting	Unclear risk	Insufficient information to permit judgement
Other bias		

Brotons (2011)

Characteristics[10]

Methods	RCT<br><br>Unit of randomisation: general practice
Participants	Place of recruitment: 42 primary care centres in 8 regions of Spain

	Numbers randomised: total: 1224 (414 stroke/TIA); I: 624 (203 stroke/TIA); C: 600 (211 stroke/TIA)<br><br>% Completing final follow-up: 70%<br><br>Inclusion criteria: cardiovascular disease (ischaemic heart disease, stroke /TIA and peripheral arterial disease); ≤ 80 years<br><br>Exclusion criteria: cardio-embolic stroke or subarachnoid haemorrhage as a result of valvulopathy; serious disease or terminal illness; bed bound<br><br>Type of stroke (%): not stated<br><br>Mean age (SE): I: 68 (11); C: 69 (11)<br><br>Gender (% men): I: 64%; C: 64%<br><br>Ethnicity: not reported<br><br>Socio-economic or socio-demographic status:<br><br>• Employment status: employed - 11%, unemployed - 2%, sick leave/invalidity - 10%, retired 61%, Other - 16%<br><br>• Education level: illiterate - 4%, uneducated, literate - 36%, primary education - 39%, secondary education - 13%, higher education - 6%, university 3%
Interventions	Intervention details (components, length, frequency): comprehensive secondary prevention program including tailored patient education, promotion of medication adherence and review of secondary prevention medication; participants attended appointment every 4 months for 2.75 years; health professionals delivering the intervention followed protocols for patient care and attended training sessions on secondary prevention of cardiovascular disease<br><br>Location: primary care<br><br>Mode of delivery: outpatient appointment<br><br>Personnel responsible for delivery: nurses with specific training in the secondary prevention of cardiovascular disease<br><br>Timing post-stroke: < 1 year<br><br>Control: usual care
Outcomes	3 years: SBP; DBP; total cholesterol; LDL; HDL; triglycerides; BMI; BP < 140/90 in non-diabetics or BP < 130/80 in diabetics/ patients with chronic renal failure; cardiovascular readmissions; cardiovascular fatal events
Notes	Funding: project co-ordinated and funded by the FIS (PI031421), Instituto de Salud Carlos III, Ministry of Health and Consumer Affairs<br><br>Country of origin: Spain

	Publication language: English<br><br>Analysis method: intention-to-treat<br><br>Risk of bias: low

Risk of Bias[10]

Bias	Author's Judgment	Support for Judgement
Random sequence generation	Low risk	Random numbers generated using a validated computer program
Allocation concealment	Low risk	Central allocation service, stratified by region ("the randomization sequence was not revealed until the intervention was assigned")
Blinding of participants and personnel		
Blinding of outcome assessment		
Incomplete outcome data	Low risk	Missing data reported by group<br><br>Attrition: I: 11 died; 51 lost to follow-up (reasons provided); 6 unknown; C: 13 died; 69 lost to follow-up (reasons provided); 41 unknown*<br><br>*study authors explain that it was difficult to recover reasons for losses in control group because they were visited only at baseline and at end of follow-up<br><br>Judgement: reasons for missing data reported and review authors judge that they are unlikely to be related to study outcomes
Selective reporting	Low risk	Study protocol available and outcomes are reported in pre-specified way
Other bias	Low risk	The study appears to be free of other sources of bias

Brunner (2012)

Characteristics[13]

Methods	Randomisation by computer<br>Blinded outcome assessor<br>Post-treatment withdrawals 6%<br>Multicentre, inpatients and outpatients
Participants	Norway<br>Recruited from 2 hospitals in the City of Bergen<br>30 participants: 14 intervention, 16 control<br><br>Inclusion criteria: cerebrovascular accident between 2 and 16 weeks; ability to extend the affected wrist and fingers at least 10°<br><br>Exclusion criteria: additional neurological diseases, unstable medical conditions, musculoskeletal disorders affecting arm mobility and severe cognitive impairment<br><br>Mean age (SD): intervention group: 61 (10) years, control group: 64.8 (12.8) years<br>% women: intervention group 21%, control group: 50%<br><br>Stroke details: ischaemic or haemorrhagic; 43% with right hemiparesis in treatment group, 37% with right hemiparesis in control group<br><br>Time since stroke, mean (SD): intervention group 1.6 (1.3) months, control group 1.23 (0.8) months
Interventions	mCIMT versus control<br>mCIMT: task-related arm training, strength training, mobility training with shaping approach and self training focusing on unilateral activities<br><br>Amount of restraint: 4 hours per day<br>Anatomical region restraint: hand<br><br>Control: task-related arm training, strength training, mobility training with shaping approach and self training focusing on bilateral activities<br><br>Session duration: 4 hours a week with physiotherapist plus 2-3 hours everyday of self-training for 4 weeks for both groups
Outcomes	Measures pre/post treatment<br>* Arm motor function: ARAT * Dexterity: 9HPT
Notes	

Risk of Bias[13]

**Bias**	**Author's Judgment**	**Support for Judgement**
Random sequence generation	Low risk	Quote: "A randomized controlled trial was applied. A computerized random numbers generator was used for randomising the patients in blocks of four patients into a modified constraint-induced movement therapy or a bimanual training group"

Allocation concealment	Low risk	Quote: "Opaque, sealed envelopes were prepared by a person not involved in the study, classifying the patients into one of the two groups"
Blinding of participants and personnel	Low risk	Quote: "The randomizations led to a balanced allocation, and blinded raters secured unbiased assessments"
Blinding of outcome assessment	Low risk	Quote: "The randomizations led to a balanced allocation, and blinded raters secured unbiased assessments"
Incomplete outcome data		
Selective reporting		
Other bias		

Burgar (2011)

Characteristics[6]	
Methods	Prospective, single-blinded RCT<br>Method of randomisation: stratified random number table
Participants	Country: USA<br><br>Sample size: 54 participants (19 in the first treatment group, 17 in the second treatment group, and 18 in the control group)<br><br>Inclusion criteria: primary diagnosis of stroke<br><br>Exclusion criteria: people were excluded if they exhibited upper limb joint pain that restricted normal movement, had absent proprioception at the elbow or shoulder joints, or scored less than 22 on the MMSE. People with cardiovascular, orthopaedic, or neurological conditions that would have precluded exercise in short-duration, moderate-workload trials were also excluded
Interventions	3 groups:<br><br>1. Robot-Lo: received up to 15 1-hour therapy sessions over a 3-week period with the MIME system<br><br>2. Robot-Hi: received up to 30 1-hour therapy sessions over a 3-week period with the MIME system<br><br>3. control group: received up to 15 1-hour therapy sessions over a 3-week period
Outcomes	Outcomes were recorded at baseline, just after completion of training (after 3 weeks), and 6 months later (follow-up)<br><br>1. FMA (maximum 66 points)<br>2. FIM (upper limb, maximum 63 points)<br>3. Motor Power (maximum 70*)

	4. Ashworth (MAS maximum 5 points)<br>5. WMFT Functional Ability Scale (maximum 5 and time in seconds)<br><br>*The strength of 14 shoulder and elbow muscle groups was assessed by performing manual muscle testing of isolated joint actions and applying the MRC Motor Power grading scale (0 to 5) with a maximum possible score of 70 (scapular abduction/upward rotation, scapular elevation, adduction, adduction/depression, adduction/downward rotation, flexion, extension, abduction, horizontal adduction, horizontal abduction, external rotation, internal rotation, elbow flexion, elbow extension)
Notes	

Risk of Bias[6]

Bias	Author's Judgment	Support for Judgement
Random sequence generation	Low risk	Random number table
Allocation concealment	Unclear risk	Unclear, not reported
Blinding of participants and personnel		
Blinding of outcome assessment	Low risk	A second therapist at each site, blinded to group assignment, performed a clinical assessment battery just before study initiation, just after completion of training, and again at the 6-month follow-up
Incomplete outcome data		
Selective reporting	Unclear risk	Insufficient information to permit judgement
Other bias		

Carnaby (2006)

Characteristics[14]

Methods	Computerised randomisation<br><br>Blinded outcome assessments by SLT ITT (High-intensity vs low-intensity data set)<br><br>Baseline prognostic factors balanced between treatment groups
Participants	1 centre in Australia<br><br>306 participants; baseline characteristics similar<br><br>Enrolment within 2 weeks of stroke onset: mean/median 2 days, range 0 to 12 days<br><br>Clinical and videofluoroscopic evidence of dysphagia
Interventions	Rx 1: standardised high-intensity swallowing therapy (n = 102)

	Rx 2: standardised low-intensity swallowing therapy (n = 102); split into (n = 51) for each data set<br><br>C: usual care (n = 102)<br><br>Treatment for up to 1 month
Outcomes	Outcomes: time to return to normal diet; aspiration pneumonia; dysphagia (PHAD score < 85)
Notes	Trial completed and published 2006<br><br>Exclusions: previous swallowing therapy, head and neck surgery, inability to consent<br><br>Follow-up: 6 months

Risk of Bias[14]

Bias	Author's Judgment	Support for Judgement
Random sequence generation	Low risk	Treatment allocation based on a computer-generated random numbers list generated via the SPSS statistical package
Allocation concealment	Low risk	Randomisation schedule held at the trial office, remote from the study environment; assignment to 1 of 3 treatment options by a telephone call to the trial office made by the study speech pathologist
Blinding of participants and personnel	High risk	Participants and speech pathologist aware of treatment allocation
Blinding of outcome assessment	Low risk	Outcome assessed by an independent speech pathologist, who was unaware of treatment allocation, every month for 6 months after randomisation
Incomplete outcome data	Low risk	3 participants lost to follow-up before 6-month analysis
Selective reporting	Low risk	All outcomes reported
Other bias	Low risk	None identified

Chen (2014)

Characteristics[15]

Methods	Single-blind, randomized, controlled clinical trial

Participants	30 participants with chronic MCA infarction and neurological deficits of intermediate severity<br><br>Treatment 15, control 15<br><br>Treated between 6 months and 5 years from stroke onset
Interventions	Subcutaneous granulocyte-colony stimulating factor injections (15 μg/kg/day) for 5 consecutive days, followed by stereotaxic implantation of autologous 3 to 8 million CD34+ immunosorted peripheral blood stem cells
Outcomes	Improvements in stroke scales (NIHSS, European Stroke Scale, and European Stroke Scale Motor Subscale) and functional outcomes measure (mRS) from baseline to the end of the 12-month follow-up
Notes	None

Risk of Bias[15]

Bias	Author's Judgment	Support for Judgement
Random sequence generation	Low risk	Participants were assigned randomly (1:1) via SAS software to either the peripheral blood stem cells or the control groups
Allocation concealment	Low risk	Participants were correctly allocated
Blinding of participants and personnel	High risk	Experimental procedures (such as stereotaxic implantation) were not blinded
Blinding of outcome assessment	Low risk	All clinical information was assessed by clinician raters in a single-blinded fashion (at clinical evaluation, each participant wore a hat to mask the surgical scar on their skull)
Incomplete outcome data	Low risk	Outcome data were complete
Selective reporting	Low risk	Primary outcomes listed in published protocols were adequately reported in the results
Other bias	Low risk	Granulocyte-colony stimulating factor may be neuroprotective in the acute phase, but this trial included participants treated between 6 months and 5 years from stroke onset

Chen (2017)

Characteristics[16]

Methods	RCT
Participants	Recruited from a Hospital in Shanghai, China<br><br>Inclusion criteria: aged 35 to 85 years old; first diagnosis was ischaemic or haemorrhagic stroke or recurrent stroke but without hemiplegia symptoms before; have a symptom of hemiplegia, left or right; 14 to 90 days from stroke onset; National Institute of Health Stroke Scale scores from 2 to 20 and mRS scores from 1 to 5; have not previously received any rehabilitation intervention since this stroke onset<br><br>Exclusion criteria: Glasgow Coma Scale scores under 15, have been confirmed as having dementia based on Mini Mental State Examination assessment, with mental disorders and unable to cooperate with examination, treatment or follow-up; disability not induced by stroke or disability induced by historical stroke; associated severe primary disease of heart, liver, kidney, or haematological system; cognitive disorder, history of psychosis, substance abuse, or alcoholism; skin infections in the areas of surface electrodes attached; metal implants in the body, including cardiac pacemaker, metal stent, or steel plate; in the gestation or lactation period or have a fertility plan; associated malignant tumour or severe progressive disease in any other system; have been recruited by any other clinical trial in the preceding 90 days; unable to complete the basic course of treatment, with poor treatment adherence or inability to follow-up<br><br>Age, years: intervention group mean 66.5 SD (12.1), control group mean 66.2 SD (12.3)<br>Gender: 67% men<br><br>Time post-stroke: intervention group mean (SD) 25.0 (5.6) days, control group 26.9 (4.7) days
Interventions	After discharge, participants in both groups were given physical exercises and electromyography-triggered neuromuscular stimulation (ETNS). Exercises were conducted for 1 hour, twice in a working day for 12 weeks (total = 60 sessions). ETNS was conducted by using a portable muscle electricity biofeedback instrument for 20 minutes, twice in a working day for 12 weeks, a total of 60 sessions.<br><br>Telerehabilitation intervention: Individualised telerehabilitation physical exercise plan selected by treating therapists and provided as prescription within the telerehabilitation apparatus. Therapists explained and demonstrated exercises. After discharge, participants received rehabilitation via the telerehabilitation system; therapists supervised via live video and collected data remotely. Therapists were available for advice if needed. Carers kept training logs of training.<br><br>Control intervention: received rehabilitation in the outpatient therapy department. Exercises and ETNS were the same but the therapy was provided face-to-face with therapists.
Outcomes	Timing of outcome assessment: baseline, 12, and 24 weeks after randomisation

	Measures: Modified Barthel Index; Berg Balance Scale; mRS; Caregiver Strain Index; Root Mean Square
Notes	

Risk of Bias[16]

Bias	Author's Judgment	Support for Judgement
Random sequence generation	Low risk	Computer-generated sequence
Allocation concealment	Low risk	Held in opaque sealed envelopes
Blinding of participants and personnel		
Blinding of outcome assessment	Low risk	Blinded outcome assessors
Incomplete outcome data	Low risk	Few withdrawals, even across groups, and reasons reported
Selective reporting	Low risk	Trial registered and all outcomes reported
Other bias	Low risk	No other sources of bias noted

Ciccone (2010)

Characteristics[17]

Methods	Randomised, controlled, multi-centre trial
Participants	* Age 18-80 years<br>* Sudden focal neurological deficit attributable to a stroke<br>* Clearly defined time of onset, allowing initiation of IV treatment within 3 h of symptom onset and intra-arterial treatment as soon as possible and, in any case, within 6 h from symptom onset (having verified the availability of the interventional neuroradiologist and taking into account all possible impediments)
Interventions	Intervention group: decided by interventional neuroradiologist. Either mechanical thrombus disruption using microcatheter, suction thrombectomy, clot extraction by retrieval device. Intra-arterial rtPA at a dose of 0.9 mg/kg (maximum: 90 mg) could be given either alone or in addition to mechanical clot disruption. Concomitant heparin flush with 2000 U bolus, followed by 500 U per hour<br><br>Control group: IV t-PA at a dose of 0.9 mg/kg (maximum: 90 mg), with 10% given as an initial bolus and the remaining 90% as a constant infusion over a period of 1 h
Outcomes	Primary outcomes: mRS score 0-2 at 90 days' follow-up<br><br>Secondary outcomes: decrease in NIHSS, baseline to day 7; neurological deterioration (≥ 4-point NIHSS) on day 7; any neurological deterioration during the first 7 days; all-cause death
Notes	Pilot study

Risk of Bias[17]

Bias	Author's Judgment	Support for Judgement

Random sequence generation	Low risk	Random assignment to treatment was stratified per centre and prepared in a ratio 1:1 with casual numbers by a person not involved in the recruitment and not operating in any of the recruiting centres. This person prepared opaque, sealed envelopes that were sequentially opened by the randomising investigators
Allocation concealment	Low risk	Open-label
Blinding of participants and personnel	High risk	Participants and personnel were not blinded to treatment allocation
Blinding of outcome assessment	Low risk	Outcome assessors were blinded to treatment allocation
Incomplete outcome data	Low risk	No loss to follow-up
Selective reporting	Low risk	ITT analyses
Other bias	Unclear risk	No

Combs-Miller (2014)

Characteristics[18]	
Methods	RCT<br>Method of randomisation: drawing sealed envelopes<br>Blinding of outcome assessors: stated as 'yes' by the investigator<br>Adverse events: not stated<br>Deaths: not stated<br>Dropouts: 2 (0 in group BWSTT, 2 in group OWT at 3-month follow-up)<br>ITT: yes
Participants	Country: USA<br>20 participants (10 in group BWSTT, 10 in group OWT)<br>Ambulatory at study onset<br>Mean age: 61 years (56 years BWSTT, 66 years OWT)<br>Inclusion criteria: minimum of six months post ischaemic or haemorrhagic stroke; age between 21 and 80 years; community dwelling; able to walk with or without an assistive device or orthosis at a self-selected gait speed of < 1.0 m/s over 10 m; medically stable with physician release; able to follow two-step verbal instructions Exclusion criteria: currently in physical therapy; health conditions prohibiting exercise or influencing walking ability
Interventions	2 arms:<br><br>BWSTT group undertook 30 minutes of treadmill training with systematically less body weight support (start at 30%), 5 times per week for 2 weeks

	OWT group B undertook overground walking training at fast speed, 5 times per week for 2 weeks
Outcomes	Outcomes were recorded at baseline and immediately after, and three months following the intervention<br>* distance in the 6-Minute Walk Test<br>* 10 m walking speed<br>* spatio temporal symmetry<br>* ICF Measure of Participation and ACTivity
Notes	

Risk of Bias[18]

**Bias**	**Author's Judgment**	**Support for Judgement**
Random sequence generation	Unclear risk	Method for randomisation not clearly described
Allocation concealment	Low risk	Sealed envelopes as method for allocation concealment after baseline assessment
Blinding of participants and personnel		
Blinding of outcome assessment	Low risk	Blinding of assessor was provided
Incomplete outcome data		
Selective reporting		
Other bias		

Connolly (2009)

Characteristics[19]

Methods	Study design: RCT, safety/efficacy study<br>Power calculation: 90%<br><br>Number of participants randomised: 18,113 (dabigatran: 12,091; VKA: 6022)<br>Number of participants analysed: 18,113<br>Number of exclusions post-randomisation: 0<br>Number of withdrawals and reasons: 3274 (18.1%); 2372 (19.6%) from the dabigatran (all doses) group and 902 (15.0%) from the warfarin group. The principal reason was participant decision: 914 (7.6%) and 375 (6.2%) from the dabigatran and warfarin group respectively. Other reasons were: outcome event: 356 (2.9%) versus 130 (2.2%), serious adverse event: 329 (2.7%) versus 105 (1.7%), gastrointestinal symptoms: 264 (2.2%) versus 38 (0.6%) and gastrointestinal bleeding 138 (1.1%) versus 54 (0.9%). Only 20 participants were lost during the study. It is not specified whether they were in the VKA or DTI group<br><br>ITT analysis: yes<br>Treatment within target INR: 64%<br>Source of funding: pharmaceutical: Boehringer Ingelheim

Participants	Country: 44 (Argentina, Australia, Austria, Belgium, Brazil, Bulgaria, Canada, China, Colombia, Czech Republic, Denmark, Finland, France, Germany, Greece, Hong Kong, Hungary, India, Italy, Japan, Republic of Korea, Malaysia, Mexico, Netherlands, Norway, Peru, Philippines, Poland, Portugal, Romania, Russian Federation, Singapore, Slovakia, South Africa, Spain, Sweden, Switzerland, Taiwan, Thailand, Turkey, Ukraine, UK, USA)<br><br>Setting/location: hospitals<br>Number of centres: 951<br>Age: 71<br>Sex: 63% male<br><br>Inclusion criteria<br>* AF (paroxysmal, persistent, or permanent), documented by ECG on the day of screening and within the past 6 months<br>* One or more of the following conditions:<br>* history of previous stroke, TIA, or systemic embolism<br>* ejection fraction < 40% documented by echocardiogram, radionuclide or contrast angiogram in the last 6 months<br>* age ≥ 75<br>* age ≥ 65 and diabetes mellitus on treatment<br>* coronary artery disease<br>* age ≥ 65 and documented coronary artery disease<br>* age ≥ 65 and hypertension requiring treatment<br>* Age ≥ 18 years at entry<br>* Written informed consent<br><br>Exclusion criteria<br>* Prosthetic heart valves requiring anticoagulation per se, or haemodynamically relevant valve disease that is expected to require surgical intervention during the course of the study<br>* Severe, disabling stroke within the previous 6 months, or any stroke within the previous 14 days<br>* Conditions associated with an increased risk of bleeding<br>* Contraindication to warfarin treatment<br>* Reversible causes of AF (e.g. cardiac surgery, pulmonary embolism, untreated hyperthyroidism).<br>* Plan to perform a pulmonary vein ablation or surgery for cure of the AF<br>* Severe renal impairment (estimated creatinine clearance ≤ 30 mL/min)<br>* Active infective endocarditis<br>* Active liver disease<br>* Women who are pregnant, lactating, or of childbearing potential who refuse to use a medically acceptable form of contraception throughout the study<br>* Anaemia (haemoglobin < 100 g/L) or thrombocytopenia (platelet count < 100 x 10^9/L)<br>* Participants who have developed transaminase elevations upon exposure to ximelagatran<br>* Participants who have received an investigational drug in the past 30 days or are participating in another drug study<br>* Participants considered unreliable by the investigator concerning the requirements for follow-up during the study and/or compliance with study drug administration, has a life expectancy less than the expected duration of the trial due to concomitant disease, or has any condition which in the

	opinion of the investigator, would not allow safe participation in the study (e.g. drug addiction, alcohol abuse)<br>* Any known hypersensitivity to galactose if the warfarin used contains galactose
Interventions	Treatments: dabigatran etexilate 110 mg and 150 mg bid<br>Control: VKA (warfarin) with target INR: 2.0 to 3.0<br>Duration: 2 years
Outcomes	Primary outcomes<br>* Yearly event rate for composite endpoint of stroke or systemic embolism event: time to first occurrence of stroke or systemic embolic eventSecondary outcomes<br>* Yearly event rate for composite endpoint of stroke, SEE and all-cause death: time to first occurrence of stroke, systemic embolism or all-cause death<br>* Yearly event rate: composite of stroke, systemic embolism event, pulmonary embolism, MI and vascular death: time to first occurrence of stroke, systemic embolic event, pulmonary embolism, MI or vascular death
Notes	

Risk of Bias[19]

**Bias**	**Author's Judgment**	**Support for Judgement**
Random sequence generation	Low risk	Computer-generated scheme
Allocation concealment	Low risk	Central randomisation through an interactive voice response system
Blinding of participants and personnel	Unclear risk	"Dabigatran was administered in a blinded fashion [...] warfarin was administered in an unblinded fashion"
Blinding of outcome assessment	Low risk	The primary data analyses and evaluation of outcome events were carried by an independent research centre
Incomplete outcome data	Unclear risk	A major proportion of participants in the dabigatran group (19.6% versus 15.0%) discontinued treatment; not all the causes of discontinuation are specified in the study. It is mentioned that only 20 participants were lost during the study, it is uncertain whether these were from the DTI or VKA groups. They used an ITT analysis and all participants randomised were analysed in their original groups

Selective reporting	Unclear risk	The study is registered in clinicaltrials.gov and their outcomes are reported according to that guideline. Protocol could not be found
Other bias		

Connolly (2013)

Characteristics[20]

Methods	Randomised, partially-blinded, active controlled trial
Participants	508 people with documented non-valvular AF and an indication for anticoagulation with VKA
Interventions	Betrixaban (40 mg, 60 mg or 80 mg daily; n = 381) or dose-adjusted warfarin (target INR 2.0 to 3.0) for at least 3 months (n = 127)
Outcomes	Primary safety outcome: composite of major or clinically relevant non-major bleeding (ISTH criteria)<br><br>Secondary safety and efficacy outcomes: stroke (fatal and non-fatal); myocardial infarction; systemic embolic events; pulmonary embolism; all-cause death; other adverse events
Notes	Study sponsored by Portola Pharmaceuticals

Risk of Bias[20]

**Bias**	**Author's Judgment**	**Support for Judgement**
Random sequence generation	Low risk	Participants were randomly assigned to treatment groups
Allocation concealment	Low risk	Participants were randomly assigned to treatment groups with a computerised interactive voice response system
Blinding of participants and personnel	High risk	Open-label administration of both betrixaban and warfarin. Separate dosages of betrixaban administered in double-blind fashion
Blinding of outcome assessment	Low risk	Adjudication of safety and efficacy outcomes by an independent clinical endpoint committee that was blinded to treatment assignment
Incomplete outcome data	Low risk	Outcomes reported in ITT population. Number of participants that discontinued during study stated with reason
Selective reporting	Low risk	All predefined outcomes reported for ITT population
Other bias	Low risk	

Cowles (2012)

Characteristics[21]	
Methods	Randomized, controlled, observer-blind efficacy trial
Participants	All participants were recruited from an inpatient stroke unit<br><br>29 participants: 15 experimental group, 14 control group<br><br>Inclusion criteria: adults who had suffered a stroke between 3 and 31 days before recruitment but with an intact premotor area (ability to encode observed human actions); able to produce some movement in a substantially paretic upper limb as assessed by a score of at least 18 on the MI but unable to produce a grip force of more than 65% of that of the ipsilesional side; free from observable contralesional upper limb movement deficits attributable to pathology other than stroke<br><br>Exclusion criteria: patients who had no ability to imitate action with their ipsilesional limb. This ability was assessed by the research therapist who performed an upper limb activity 3 times, and potential participants were asked to observe and then perform the activity. This assessment was digitally filmed and saved onto a computer. An independent assessor viewed the recordings and judged the accuracy of imitation.<br><br>Mean (SD) age: 77.2 (SD 10.4) years<br><br>Stroke details: not reported by authors<br><br>Stroke phase: acute
Interventions	Experimental group additionally watched functional tasks with intent to imitate for 1 to 2 minutes; and then for 4 to 6 minutes performed the activity simultaneously with the therapist<br><br>Control group received conventional physical therapy<br><br>Sessions were 2 × 30-minute sessions, with 10-minute rest, for 15 working days
Outcomes	Outcomes recorded at baseline and within 3 working days of the end of intervention<br><br>Ability to voluntarily contract paretic muscle: MI<br><br>Upper limb motor function: ARAT
Notes	

Risk of Bias[21]

**Bias**	**Author's Judgment**	**Support for Judgement**
Random sequence generation	Low risk	Group allocation was computer generated
Allocation concealment	Low risk	Details of group allocation were placed into sequentially-

		numbered, opaque, sealed envelopes
Blinding of participants and personnel	Low risk	No blinding, but the outcome is not likely to be influenced by lack of blinding
Blinding of outcome assessment	Low risk	All measures were made by an assessor blinded to treatment allocation
Incomplete outcome data	Low risk	29 participants were randomized. Outcome measures were completed on 13 participants in the CPT group and 9 in the OTI + PP group (22 participants). Reasons for attrition were provided: moved out of area (one in CG and 4 in EG); withdrew from trial (1 in EG); medically unwell (1 in EG); 1 of the participants in the control group failed to fully comprehend the instructions for the MI at baseline so this score was missing. Reasons for missing outcome data unlikely to be related to true outcome.<br><br>Where participants withdrew before outcome they were not included in the analysis, and imputation was not used
Selective reporting	High risk	The study protocol is available but not all of the study’s pre-specified primary outcomes have been reported. Registered on the Current Controlled Trials Database — ISCRTN 51553998: www.controlled-trials.com/ISR CTN51553998/51553998.
Other bias	Low risk	

Cramer (2019)

Characteristics[16]	
Methods	RCT
Participants	Recruited from: 11 sites in the USA

	Inclusion criteria: age ≥ 18 years, stroke onset 4 to 36 weeks prior, arm motor Fugl-Meyer score 22-56 (out of 66)<br><br>Exclusion criteria: major active coexistent neurological or psychiatric disease; severe depression, cognitive impairment (MoCA < 22), communication deficits interfering with participation, life expectancy < 6 months, non-English speaking, unable to perform the 3 rehabilitation exercise test examples<br>Age, years: intervention group mean age 62 (14), control group 60 (13)<br><br>Gender: 73% men<br>Time post-stroke: intervention group mean 132 (65) days, control group 129 (59) days
Interventions	Participants in both groups were offered 36 sessions (18 supervised, 18 unsupervised) lasting for 70 minutes each over 6 to 8 weeks. All participants signed a behavioural contract that included a treatment goal and treatment was based on an upper extremity task-specific training manual and accelerated skill acquisition programme.<br><br>Telerehabilitation intervention: rehabilitation treatment sessions via an in-home internet-connected computer. The participant performed daily assigned home-based telerehabilitation exercises and functional training (including use of games and input devices such as PlayStation Move Controller) and 5 minutes of stroke education, all guided by the telerehabilitation system. During half of the sessions, therapists initiated a video conference with the participant's telerehabilitation system to discuss progress, issues, and revise treatment plans as needed.<br><br>Control intervention: same intensity, duration, and frequency of therapy and stroke education content but provided in clinic with therapist feedback based on observations on supervised days
Outcomes	Timing of outcome assessment: baseline, 30 days after randomisation<br><br>Measures: Fugl-Meyer Arm, Box and Block test, Stroke Impact Scale-Hand Domain
Notes	NCT02360488

Risk of Bias[16]

Bias	Author's Judgment	Support for Judgement
Random sequence generation	Low risk	Randomisation schedule developed at the StrokeNet National Data Management Centre
Allocation concealment	Low risk	Web-based central randomisation system
Blinding of participants and personnel		
Blinding of outcome assessment	Low risk	Blinded outcome assessment
Incomplete outcome data	Low risk	Low number of withdrawals and balanced across groups
Selective reporting	Low risk	Trial registered

Other bias	Low risk	None noted

da Cunha (2002)

Characteristics[18]	
Methods	Parallel-group design<br>Participants randomised to groups using a random number table<br>Allocation to groups was not concealed<br>13% dropouts at the end of the treatment phase<br>Outcome assessors were not blinded to group allocation
Participants	7 participants in the EXP group and 8 participants in the CTL group<br><br>Inclusion criteria: less than 6 weeks post-stroke; hemiparetic stroke based on clinical examination or MRI, or both; significant gait deficit - speed of no more than 36 m/min or FAC 0 to 2 (that is, needs assistance); sufficient cognition to participate in training (at least 21 on the MMSE); ability to stand and take at least 1 step with or without assistance; informed consent<br><br>Exclusion criteria: any comorbidity or disability other than hemiparesis that would preclude gait-training; recent myocardial infarction; any uncontrolled health condition for which exercise is contraindicated (e.g. diabetes); severe lower extremity joint disease or rheumatoid arthritis that would interfere with gait-training; obesity (mass more than 110 kg)
Interventions	Treated as inpatients for 5 x 20-minute sessions per week for 2 to 3 weeks<br>BWSTT (EXP): participants walked on a treadmill with up to 30% of their body weight supported using a harness<br><br>Regular gait-training (CTL): strengthening, functional and mobility activities
Outcomes	Assessed at baseline and after treatment phase:<br><br>• FAC<br>• FIM - locomotion score<br>• fast walking speed over 5 metres using a gait aid and personal assistance, if required<br>• walking endurance - maximum distance walked in 5 minutes, using parallel bars if necessary<br>• energy expenditure during gait<br>• bike ergometer exercise test
Notes	The rating of dropouts and the allocation concealment classification were changed based on correspondence from the trialist

Risk of Bias[18]

**Bias**	**Author's Judgment**	**Support for Judgement**
Random sequence generation	Low risk	Random number table
Allocation concealment	High risk	Inadequate (based on correspondence from the investigator)

Blinding of participants and personnel		
Blinding of outcome assessment	High risk	Not blinded (based on correspondence from the investigator)
Incomplete outcome data		
Selective reporting		
Other bias		

Davis (2008)

Characteristics[22]	
Methods	Double-blind, randomised, prospective, phase II placebo-controlled study<br><br>Location: 15 centres in Australia, New Zealand, Belgium and the UK during 2001 to 2007<br><br>Method of randomisation: computerised randomisation giving out the number of a treatment pack<br><br>The packs were centrally prepared in blocks for each centre (4 treatment packs per block)<br><br>Analysis: intention-to-treat, and of primary outcome measure: per-protocol
Participants	Acute hemispheric ischaemic stroke<br><br>The participants were randomised on the basis of plain CT<br><br>Age: ≥ 18 years<br><br>NIHSS: ≥ 4; premorbid mRS ≤ 2<br><br>Time window: 3 to 6 hours<br><br>Baseline CT (in one centre MRI) to exclude: haemorrhage and early ischaemic changes ≥ ⅓ of the MCA territory; CT was repeated in clinical deterioration possibly due to a haemorrhagic transformation<br><br>MRI was performed before start of treatment, at day 3 to 5 (DWI, PWI/concentration-time curves after gadolinium, and MRA (time of flight or phase contrast))<br><br>At day 90 (T2-weighted images to measure final infarct volume)<br><br>To standardise image analysis, all MRI were centrally read<br><br>Number randomised: 101 (treatment group: 52; control group: 49)
Interventions	Alteplase (rt-PA) 0.9 mg/kg i.v. up to a maximum of 90 mg, 10% given as a bolus, the remainder as infusion over 1 hour, or placebo
Outcomes	MRI definitions and outcome measures: mismatch, PWI-DWI volume > 1 to 2, and PWI-DWI volume ≥ 10ml; infarct growth, 4 measurements, i.e.

	expansion between baseline and day 90 T2-weighted lesion; reperfusion > 90% reduction between baseline and day 3 PWI volumes; recanalisation: improvement of TIMI from baseline to day 3 to 5 by ≥ 2 points; SICH according to SITS-MOST criteria; target mismatch, mismatch excluding 'malignant profile'; 'malignant profile': DWI volume ≥100 ml, PWI volume ≥ 100 ml, or both with PWI defined as Tmax delay ≥ 8 seconds<br><br>Clinical: NIHSS at day 3 to 5 and day 90; mRS day 90<br><br>Good neurological outcome: NIHSS 0 to 1 or improvement ≥ 8 from baseline<br><br>Good functional outcome: mRS 0 to 2<br><br>Primary outcome measure: infarct growth<br><br>Secondary outcome measures included: difference in mismatch participants in reperfusion; good neurological and functional outcome between the treatment and control group; difference in DWI lesion volumes in the treatment group between participants with and without SICH; difference in infarct growth in non-mismatch participants between the treatment arms; difference in infarct growth, good neurological and functional outcomes in the treatment group between participants with and without mismatch; difference in infarct growth, good neurological and functional outcomes in the treatment group between participants with target mismatch and those with 'malignant profile'
Notes	The paucity of participants without mismatch precluded comparisons of the effect of rt-PA in the presence versus absence of mismatch

Risk of Bias[22]

Bias	Author's Judgment	Support for Judgement
Random sequence generation		
Allocation concealment	Low risk	
Blinding of participants and personnel		
Blinding of outcome assessment		
Incomplete outcome data		
Selective reporting		
Other bias		

Dennis (2006a)

Characteristics[23]

Methods	Computerised randomisation by minimisation<br><br>Blinded outcome assessment by post or telephone<br><br>Cross-overs: 13 in NGT group received early PEG, 23 allocated to PEG received NGT

	Baseline prognostic factors balanced between treatment groups
Participants	47 centres in 11 countries<br><br>321 dysphagic patients: 144 male<br><br>Mean age 76 (SD 10) years<br><br>Stroke 100%<br><br>Enrolment within 30 days of stroke onset
Interventions	Rx: PEG feeding (within 3 days of enrolment) (n = 162)<br><br>C: NGT (n = 159)
Outcomes	Primary outcomes: dead or dependent (mRS 4 to 6); death at 6 months<br><br>Secondary outcomes: place of residence, EURO-QoL, treatment compliance, length of hospital stay, discharge destination, treatment complications
Notes	Exclusions: SAH<br>Follow-up: 6 months

Risk of Bias[23]

Bias	Author's Judgment	Support for Judgement
Random sequence generation	Low risk	Used a computer-generated minimisation algorithm
Allocation concealment	Low risk	The randomisation systems were housed on a secure server with access permitted, via a password, only to those members of the coordinating team who had been fully trained how to use the systems<br><br>Participating centres were issued with codes in order for them to access the randomisation services
Blinding of participants and personnel	High risk	FOOD was an open trial, with both the randomising person and the patient being aware of the treatment allocation The only blinded assessment was the 6-month follow-up
Blinding of outcome assessment	Low risk	As above
Incomplete outcome data	Low risk	None lost to follow-up
Selective reporting	Low risk	
Other bias		

Dennis (2006b)

Characteristics[23]

Methods	Computerised randomisation by minimisation<br>Blinded outcome assessment by post or telephone<br>Cross-overs: 3 normal diet to supplement, 48 supplement to normal diet, 79 did not receive allocated supplements<br>Baseline prognostic factors balanced between treatment groups
Participants	125 centres in 15 countries<br>4023 non-dysphagic patients: 2149 male<br>Mean age 71 (SD 13) years<br>Stroke 99%<br>Enrolment within 30 days of stroke onset
Interventions	Rx: protein (22.5 g per day) energy (540 kcal) supplements + normal hospital diet (n = 2011)<br>C: normal hospital diet (n = 2001)
Outcomes	Primary outcomes: dead or dependent (mRS 3 to 6); death at 6 months<br>Secondary outcomes: place of residence, EURO-QoL, treatment compliance, length of hospital stay, discharge destination
Notes	Exclusions: dysphagia, SAH<br>Follow-up: 6 months

Risk of Bias[23]

**Bias**	**Author's Judgment**	**Support for Judgement**
Random sequence generation	Low risk	Used a computer-generated minimisation algorithm |
Allocation concealment	Low risk	The randomisation systems were housed on a secure server with access permitted, via a password, only to those members of the co-ordinating team who had been fully trained how to use the systems<br><br>Participating centres were issued with codes in order for them to access the randomisation services
Blinding of participants and personnel	High risk	All outcomes: FOOD was an open trial, with both the randomising person and the patient being aware of the treatment allocation<br><br>The only blinded assessment was the 6-month follow-up<br><br>All outcomes: As above
Blinding of outcome assessment	All outcomes: High risk<br>All outcomes: Low risk	All outcomes: FOOD was an open trial, with both the

		randomising person and the patient being aware of the treatment allocation<br><br>The only blinded assessment was the 6-month follow-up<br><br>All outcomes: As above
Incomplete outcome data	Low risk	11 lost to follow-up
Selective reporting	Low risk	
Other bias	Unclear risk	

Dennis (2009)

Characteristics[24]	
Methods	Study: RCT<br>Exclusion to post-randomisation: 0<br>Losses to follow up: 69 missing data (41 in treatment group and 28 in control group) - no CDU prior to death or 30 days<br>DVT diagnosis: CDU (minimum of the popliteal and femoral veins) between day 7 and 10 and between day 25 and 30<br>Statistical analysis: odds ratio and NNT<br>Scheduled treatment and follow-up period: 30 days; clinical follow up at 6 months
Participants	Country: UK, Italy and Australia<br>Total number of participants: 2518<br>Total available for analysis: 2518<br>Age: 76 years (68 to 83) for both groups<br>Sex: males 49.4% (620/1256) in the treatment group and 49.3% in the control group (622/1262)<br>Immobilisation: yes<br>Inclusion criteria: patients admitted with an acute stroke up to day 3 post-admission<br>Exclusion criteria: peripheral vascular disease, or with diabetic/sensory neuropathy, if clinicians judged GCS could cause skin damage<br>Full intention-to-treat analysis: performed
Interventions	Type: thigh-length Tyco Healthcare TED GCS<br>Control: 1262<br>Treatment: 1256<br>Duration applied: night and day until death/discharge/mobile/refused<br><br>Use of anticoagulants post randomisation: group allocated GCS<br>• 117 post-randomisation prophylactic dose heparin/LMWH prescribed<br>• 78 post-randomisation treatment dose heparin/LMWH prescribed<br>• 186 post-randomisation warfarin prescribed<br><br>Use of anticoagulants post randomisation: group allocated 'avoid GCS'<br>• 129 post-randomisation prophylactic dose heparin/LMWH prescribed<br>• 97 post-randomisation treatment dose heparin/LMWH prescribed<br>• 208 post-randomisation warfarin prescribed

Outcomes	Any DVT<br>Control: 224<br>Treatment: 205<br>P value: ns
Notes	The primary outcome focused on proximal DVTs (popliteal or femoral) rather than any DVT. Randomising clinicians were allowed to elect prior to randomisation whether patients would have a second CDU at 25 to 30 days. The 6-month outcomes have not yet been reported. The median delay from stroke onset to enrolment was 2 days but there was no trend towards more effect with earlier recruitment

Risk of Bias[24]

Bias	Author's Judgment	Support for Judgement
Random sequence generation	Low risk	A - Adequate. Computer-generated allocation based on within centre minimisation for prognostic factors (stroke onset; stroke severity; leg paresis; use of anticoagulants) plus random allocation
Allocation concealment	Low risk	A - Adequate. Central allocation (web and telephone)
Blinding of participants and personnel		
Blinding of outcome assessment	Low risk	A - Adequate. GCS removed before CDU was performed Hard copies of positive scans were recorded for independent assessment
Incomplete outcome data	Low risk	A - Adequate. 69 missing data
Selective reporting		
Other bias		

DePaul (2014)

Characteristics[18]

Methods	RCT<br>Method of randomisation: permuted block randomisation schedule<br>Blinding of outcome assessors: stated as 'yes' by the investigator<br>Adverse events: one or more falls (11 participants in the MLWP and 10 participants in the BWSTT group had 1 fall;<br>2 in the MLWP and 4 in the BWSTT group reported multiple falls); new stroke: 3 in MLWP, 1 in BWSTT; cardiac event: 2 in the BWSTT group<br>Deaths: 4 (2 in the MLWP, 2 in the BWSTT)<br>Dropouts: 4 (0 in EXP group A, 1 in EXP group B, 3 in CTL group)<br>ITT: yes
Participants	Country: Canada

	70 participants (35 in MLWP, 35 in BWSTT)<br>Ambulatory at study onset<br>Mean age: 68 years; (MLWP 66 years, BWSTT 69 years)<br><br>Inclusion criteria: ≥ 40 years old, living in the community, < 12 months since onset of Ischaemic or haemorrhagic stroke, able to walk 10 m without assistance (gait aid allowed), able to follow a 2-step verbal command, and independent community ambulation prior to stroke<br><br>Exclusion criteria: cognitive impairment (i.e. MMSE score less than age and education norms); severe visual impairment; lower-extremity amputation; unstable cardiac, medical, or musculoskeletal conditions that would limit treatment participation (determined by physician screening and baseline interview); comfortable gait speed > 1.0 m/s without a gait aid
Interventions	2 arms:<br><br>1. MLWP group undertook a Motor LearningWalking Program and practiced various overground walking tasks for 40 minutes, 15 sessions over 5 weeks<br><br>2. BWSTT group undertook a Body-Weight-Supported Treadmill Training for 30 minutes, 15 sessions over 5 weeks
Outcomes	Outcomes were recorded at 1 week prior to initiating training, within 1 week following completion of training and 2 months after training<br>• 5-m Walk Test (maximum pace)<br>• 6-Minute Walk Test<br>• the Functional Balance Test (FBT)<br>• Activities-specific Balance Confidence Scale<br>• modified Functional Ambulation Categories<br>• Stroke Impact Scale<br>• Life Space Assessment
Notes	

Risk of Bias[18]

Bias	Author's Judgment	Support for Judgement
Random sequence generation	Low risk	Central randomisation
Allocation concealment	Low risk	Central assignment
Blinding of participants and personnel		
Blinding of outcome assessment	Low risk	Blinded physical therapist
Incomplete outcome data		
Selective reporting		
Other bias		

Diener (2008)

Characteristics[4]

Methods	Double-blind, 2 x 2 factorial trial

	Randomisation done by central telephone system
Participants	International (35 countries), multicentre (695 centres)<br><br>1360 participants. T: 647 , C: 713<br><br>Mean age T: 66.8 years, C: 67.1 years<br><br>Male T: 64.9%, C: 65.1%<br><br>Inclusion: IS<br><br>Enrolment within 72 hours<br><br>FU: no losses
Interventions	T: telmisartan 80 mg once daily<br><br>C: placebo<br><br>Rx: 2.5 years
Outcomes	Primary: BP, HR at days 7, 30 and 90 (BP and HR recorded using validated semiautomatic monitor - Omron 705CP)<br><br>Secondary: mRS day 30, haemorrhagic transformation of the infarct, cerebral oedema, recurrent stroke, MI, composite vascular events (vascular death, non-fatal stroke, or MI), death at days 7, 30 and 90
Notes	Exclusion: mRS > 3, using or needing ARA at time of randomisation, known severe renal insufficiency or renal artery stenosis, hyperkalaemia, uncorrected volume or sodium depletion, known severe coronary artery disease or recent MI, patients scheduled for carotid endarterectomy.

Risk of Bias[4]

**Bias**	**Author's Judgment**	**Support for Judgement**
Random sequence generation	Low risk	Adequate
Allocation concealment	Low risk	Central allocation ensured allocation concealment
Blinding of participants and personnel	Low risk	Double-blind
Blinding of outcome assessment	Low risk	Double-blind
Incomplete outcome data	Low risk	Endpoint adjudication committee blinded; independent safety committee blinded
Selective reporting	Low risk	All pre-specified outcomes were reported
Other bias	Low risk	None

Dippel (2001)

Characteristics[25]

Methods	Randomised, computer-generated random numbers<br>Blinding: double blind<br>Placebo controlled<br>Losses to follow up: 1<br>Intention to treat: yes
Participants	Patients with acute ischaemic anterior circulation stroke < 24 hours of symptom onset 49 patients (medium dose 25, placebo 24)<br>Mean age (SD) medium dose paracetamol 74 years (14 years), placebo 68 years (15 years)<br>30 male (61%)<br>Stroke severity: (NIHSS) mean (SD), medium dose paracetamol 10.0 (8.2), placebo 8.8 (5.4)<br>Body temperature was measured by both tympanic and rectal thermometers
Interventions	Paracetamol 500 mg suppository 6 times daily<br>Control: placebo<br>Duration: 5 days
Outcomes	Body temperature<br>Death or dependency (mRS ≥ 3) at 1 month<br>Death at 1 month<br>Intracranial/extracranial haemorrhage/haemorrhagic transformation of infarction<br>Infections<br>Other side effects: deep venous thrombosis and cardiac arrhythmias
Notes	Two intervention groups: we divided the number of participants with poor outcome and the total number of participants in the control group by 2 in order to avoid multiple comparisons using the same subset of participants

Risk of Bias[25]

**Bias**	**Author's Judgment**	**Support for Judgement**
Random sequence generation		
Allocation concealment	Low risk	A - Adequate
Blinding of participants and personnel		
Blinding of outcome assessment		
Incomplete outcome data		
Selective reporting		
Other bias		

Dohle (2009)

Characteristics[26]

Methods	RCT
Participants	Country: Germany<br><br>Setting: inpatient rehabiltation centre

	Age: aduts (mean age: 56.5 years)<br><br>Sample size: 48 participants (24 in each group, 12 dropped out)<br><br>Sex: 10 women, 26 men<br><br>Inclusion criteria: first-ever ischaemic stroke in the territory of the middle cerebral artery; not more than 8 weeks post-stroke; between 25 and 80 years old; able to follow therapy instructions; capable of participating in 30-minute daily therapy sessions<br><br>Exclusion criteria: experienced previous stroke; major haemorrhagic changes; increased intracranial pressure; hemicraniectomy or orthopaedic, rheumatologic, or other diseases interfering with their ability to sit or to move either upper limb
Interventions	2 arms<br><br>1. MT: participants were instructed to move both arms "as well as possible" while looking in the mirror<br>2. Bilateral arm training: participants performed the same treatment protocol as in group 1 but without a mirror<br><br>1 and 2: 5 days a week; 30 minutes of therapy for 6 weeks<br><br>Date of intervention: October 2004 to April 2006
Outcomes	Outcomes were recorded at baseline and after the intervention<br><br>1. FM-UE motor, ROM, pain and sensory section (FM-UE 0 to 126)<br>2. ARAT 0 to 57<br>3. FIM self-care and mobility items (7 to 77)<br>4. self-defined Neglect score (0 to 4)
Notes	Published and unpublished data; we extracted the motor section of the FM-UE (without reflex activity, 0 to 60)<br><br>Funding source: rehabilitation research network (refonet) of the German Pension Scheme Rhineland<br><br>Declarations of trialists’ interests: not stated

Risk of Bias[26]

Bias	Author’s Judgment	Support for Judgement
Random sequence generation	Low risk	Sealed, numbered envelopes were created
Allocation concealment	Low risk	Sealed envelopes were broken after study inclusion
Blinding of participants and personnel		
Blinding of outcome assessment	Low risk	Assessors of primary outcome were blinded to group allocation

Incomplete outcome data	High risk	Dropouts were not included in analysis
Selective reporting		
Other bias		

Dormandy (2005)

Characteristics[27]

Methods	A multicentre, randomised, double-blind, placebo-controlled trial
Participants	People aged 35 to 75 years with type 2 diabetes and a previous stroke (6 months before randomisation) were included.<br>984 eligible people were randomised into the trial, with 486 in the pioglitazone group and 498 in the placebo group.
Interventions	Pioglitazone titration from 15 mg to 45 mg per day depending on tolerability or placebo was given until the first occurrence of any of the events described in the primary outcomes. The mean duration was 34.5 months.
Outcomes	Primary outcomes: all-cause mortality, non-fatal myocardial infarction (including silent myocardial infarction), stroke, acute coronary syndrome, cardiac intervention (including coronary artery bypass graft surgery or percutaneous coronary intervention), leg revascularisation, and amputation above the ankle<br><br>Secondary outcomes: all-cause death, non-fatal myocardial infarction or non-fatal stroke; adverse events
Notes	We did not include data for participants without previous stroke.

Risk of Bias[27]

**Bias**	**Author's Judgment**	**Support for Judgement**
Random sequence generation	Unclear risk	Method of random sequence generation was not described.
Allocation concealment	Unclear risk	Allocation concealment was not reported.
Blinding of participants and personnel	Unclear risk	Insufficient information to judge
Blinding of outcome assessment	Unclear risk	Insufficient information to judge
Incomplete outcome data	High risk	882/984 (90%) participants completed the final visit: 439/486 (90%) in the pioglitazone group and 443/498 (89%) in the placebo group. Intention-to-treat analysis was used.
Selective reporting	Low risk	All prespecified outcomes were reported
Other bias	Unclear risk	This study was funded by Takeda Pharmaceuticals and Eli Lilly, and designed by the

		international steering committee, who also approved the protocol and amendments. The sponsors had 2 representatives on the international steering committee; the same 2 were also members of the executive committee. Data analysis, data interpretation, and writing of the report was done by the executive committee, with contributions from the international steering committee, the data and safety monitoring committee, and the endpoint adjudication committee. All the authors had full access to all the data in the study and had final responsibility for the decision to submit for publication.

Dregan (2014)

Characteristics[10]	
Methods	RCT<br><br>Unit of randomisation: family practices
Participants	Place of recruitment: primary care<br><br>Numbers randomised: total: 11,391 (I: 5875; C: 5516)<br><br>Completing final follow-up: 90%<br><br>Inclusion criteria: ≥ 18 years, included on the practice stroke register<br><br>Exclusion criteria: none stated<br><br>Type of stroke (%): haemorrhagic (I: 18 C: 16), ischaemic (I: 26 C: 21), undefined (I: 56 C: 63)<br><br>Mean age (SD): I: 72.9 (14.1); C: 72.2 (13.9)<br><br>Gender (% women) I: 49 C: 47<br><br>Ethnicity: not stated<br><br>Socio-economic or socio-demographic: not stated
Interventions	Intervention details (components, length, frequency): educational and decision support tools for primary care healthcare providers, taken from

	evidence summarised from guidelines, including clinical trials, meta-analysis and observational analysis - prompts for BP/cholesterol level/statins/anticoagulant assessment.<br><br>Mode of delivery: delivered remotely via point of care software for use in the community<br><br>Personnel responsible for delivery: software system<br><br>Timing post-stroke: unlimited<br><br>Control: usual care
Outcomes	12 months: BP and total cholesterol levels
Notes	Analysis method: marginal methods estimated using the method of generalised estimating equations<br><br>Risk of bias: Low risk

Risk of Bias[10]

Bias	Author's Judgment	Support for Judgement
Random sequence generation	Low risk	"The allocation is by minimization controlling for region in England (North (North-East and North-West), Midlands (East and West Midlands), South-East (South-East and East of England), South-West, and London) and country in the UK (Scotland, Wales, England) and list size (number of registered patients). This list size was dichotomized for the minimization using 7,500 as the cut-point. The allocation is performed at King's College London using anonymised practice identifiers supplied by the recruitment team at GPRD/MHRA"
Allocation concealment	Low risk	Allocation was performed using anonymised practice identifiers
Blinding of participants and personnel		
Blinding of outcome assessment		
Incomplete outcome data	Low risk	Risk of bias is acceptable - no added value is obvious

		from the results. Sensitivity analysis was also undertaken
Selective reporting	Low risk	The study protocol was clear and had been published prior to the study
Other bias	Low risk	The study appeared to be free of other sources of bias

Drummond (2013)

Characteristics[5]

Methods	RCT
Participants	93 patients transferred from an acute stroke unit with a confirmed diagnosis of stroke.
Interventions	The experimental group received a predischarge home assessment visit with an occupational therapist to identify and address any potential problems in the home environment<br>The control group also received a predischarge home assessment with an occupational therapist, but this was conducted in the hospital. Potential problems were discussed in general terms
Outcomes	Number of falls, economic evaluation, and quality of life
Notes	Fall registration during both intervention and follow-up period. Registration time: 1 month

Risk of Bias[5]

**Bias**	**Author's Judgment**	**Support for Judgement**
Random sequence generation	Low risk	Quote: "Patients recruited to the randomized controlled trial were registered using a web-based randomization program. This was managed by Nottingham Clinical Trials Unit who held a pre-prepared list in random varying block sizes." (Page 3)
Allocation concealment	High risk	No report about concealment was found
Blinding of participants and personnel		
Blinding of outcome assessment	Unclear risk	Insufficient explanation regarding falls evaluation.
Incomplete outcome data	Low risk	Quote: "Analyses were carried out on the basis of intention to treat. For baseline and outcome measures where less than 10% of the total data were missing, mean values were

		imputed for individual missing items. Where 10% or more data were missing, the entire measure was coded as 'missing'" (Page 3)
Selective reporting	Low risk	All of the studies' prespecified outcomes have been reported in the prespecified way
Other bias		

Du (2016)

Characteristics[14]

Methods	Randomisation by sequentially numbered sealed envelopes<br>Blinded outcome assessments by trained neurologist<br>(Sham vs low-frequency (1 Hz) data set)<br>Baseline prognostic factors balanced between treatment groups
Participants	1 centre in China<br>40 participants; baseline characteristics similar<br>Enrolment within 2 months of stroke onset confirmed by CT or MRI scan<br>Clinical evidence of dysphagia
Interventions	Rx 1: 1 Hz rTMS to unaffected hemisphere (n = 13)<br>Rx 2: 3 Hz rTMS to affected hemisphere (n = 13)<br>C: sham rTMS (n = 12), split into n = 6 for each data set<br>Treatment for up to 5 days
Outcomes	Outcomes: swallow score using Standardised Swallow Assessment (SSA), BI, mRS, and measures of mylohyoid MEPs
Notes	Exclusions: other concomitant neurological diseases, fever, infection, prior administration of tranquilliser, severe aphasia or cognitive impairment, inability to complete the follow-up, and other contraindications for rTMS<br>Follow-up: up to 3 months

Risk of Bias[14]

Bias	Author's Judgment	Support for Judgement
Random sequence generation	Low risk	Randomisation by sequentially numbered sealed envelopes
Allocation concealment	Low risk	Allocation concealed by sealed envelopes
Blinding of participants and personnel	Low risk	All outcomes: Participant blinded; outcome assessor blinded<br>All outcomes: Participant blinded
Blinding of outcome assessment	Low risk	All outcomes: Participant blinded; outcome assessor blinded

		All outcomes: Outcome assessor blinded - measures evaluated by a trained neurologist who was blinded to participants' group allocation throughout
Incomplete outcome data	Low risk	2 participants lost to follow-up
Selective reporting	Low risk	Only NIHSS not recorded at the end; all other measures reported on for all 3 time points
Other bias	Low risk	None identified

Eames (2013)

Characteristics[10]	
Methods	RCT<br><br>Unit of randomisation: participant
Participants	Place of recruitment: 2 acute stroke units in metropolitan hospitals<br><br>Numbers randomised: total: 77 (I:37; C: 40)<br><br>% Completing final follow-up: 86%<br><br>Inclusion criteria: ischaemic stroke, haemorrhagic stroke or TIA; admitted to hospital for stroke or TIA; living in a residential care facility prior to admission and it was not a planned discharge destination; adequate spoken English, cognition, communication and corrected vision and hearing to complete the outcome measures<br><br>Exclusion criteria: poor medical prognosis (i.e. medically unstable patients or those undergoing palliative treatment)<br><br>Type of stroke: ischaemic (I: 73%; C: 84%); haemorrhagic (I: 25%; C: 14%), TIA (I:3%, C: 0%)<br><br>Mean age (SD): I: 57.0 (16.6); C: 64.1 (14.3)<br><br>Gender (% men) I: 55%; C: 51%<br><br>Ethnicity: not reported<br><br>Socio-economic or socio-demographic status: not reported
Interventions	Intervention details (components, length, frequency): tailored written stroke information (stroke booklet) and verbal reinforcement of this information by a health professional (verbal reinforcement was offered face-to-face up to 3 times prior to discharge and over the telephone up to 3 times following

	discharge). Participants could tailor the content of the information booklet and the verbal sessions<br><br>Location: acute stroke unit (prior to discharge) and community/inpatient rehabilitation ward (post-discharge)<br><br>Mode of delivery: outpatient appointment<br><br>Personnel responsible for delivery: occupational therapist<br><br>Timing post-stroke: approximately 1 week prior to acute stroke unit discharge<br><br>Control: usual care (stroke unit care included usual medical, nursing, and allied health management)
Outcomes	3 months: adherence to secondary prevention medications
Notes	Analysis method: unknown<br><br>Risk of bias: low

Risk of Bias[10]

Bias	Author's Judgment	Support for Judgement
Random sequence generation	Low risk	"Concealed, random allocation was achieved via sequentially numbered envelopes containing computer-generated random numbers prepared by a person not involved in the study"
Allocation concealment	Low risk	"Baselines outcome measures were obtained prior to randomization and therefore by a blinded assessor. Administration of outcome measures at the follow-up interview was undertaken by a blinded assessor. Once completed, the assessor opened a sealed section of the form to determine group allocation and asked intervention group participants additional questions regarding the intervention." (Unpublished information provided by trialists)
Blinding of participants and personnel		
Blinding of outcome assessment		

Incomplete outcome data	Low risk	Missing data reported by group<br><br>Attrition: I: 5/40 (4 unable to be contacted; 1 cognition impairment too severe for interview follow-up; C:6/37 (2 withdrew; 3 unable to be contacted; 1 admitted to residential care<br><br>Judgement: reasons for missing data reported and review authors judge that they are unlikely to be related to study outcomes
Selective reporting	Low risk	Protocol is available and outcomes are reported in the pre-specified way
Other bias	Low risk	The study appears to be free of other sources of bias

England (2012)

Characteristics[28]

Methods	Single-centre, double-blind, randomised, placebo-controlled, phase IIb trial
Participants	60 participants (1 UK centre), 3 to 30 days post ischaemic or haemorrhagic stroke
Interventions	G-CSF (Filgrastim) 10 µg/kg versus normal saline sc for 5 days
Outcomes	(1) Serious adverse events<br>(2) Efficacy (SSS, mRS, BI, EADL), lesion volume (MRI)
Notes	Started: July 2007

Risk of Bias[28]

**Bias**	**Author's Judgment**	**Support for Judgement**
Random sequence generation	Low risk	Computerised randomisation with minimisation for baseline prognostic values
Allocation concealment	Low risk	Adequate
Blinding of participants and personnel	Low risk	Investigator, participants and analysis all blinded to treatment
Blinding of outcome assessment	Low risk	Investigator, participants and analysis all blinded to treatment
Incomplete outcome data	Low risk	No losses to follow-up
Selective reporting	Low risk	All outcomes reported
Other bias	Low risk	No conflicts of interest

England (2017)

Characteristics[29]	
Methods	Study design: RCT<br><br>Generation of the allocation sequence: minimised on age, sex, NIHSS score, and systolic blood pressure<br><br>Allocation concealment: web-based, 1:1 allocation<br><br>Study grouping: parallel group<br><br>Masking: triple-blinded (participants, investigator, outcome assessor)<br><br>Analysis method: ITT analysis<br><br>Total duration of study: 27 months, from March 2013 to July 2015
Participants	26 participants (intervention = 13; placebo control = 13)<br><br>UK<br><br>Single centre: Derby Teaching Hospitals NHS Foundation Trust<br><br>Setting: inpatient<br><br>Sex: both (65.4% men, 34.6% women)<br><br>Mean age: 76.2 years old<br><br>Inclusion criteria: adults with an ischaemic stroke in the past 24 hours causing arm or leg weakness<br><br>Exclusion criteria: mRS score > 3, thrombolysis for index event, and significant comorbidity
Interventions	Participants were randomly assigned to either of 2 groups:<br><br>Group 1: RIC group (n = 13), standard medical management plus RIC. RIC consisted of 4 cycles of intermittent limb ischaemia; alternating 5 minutes' inflation (20 mmHg above systolic blood pressure) and 5 minutes' deflation performed manually using a standard upper arm blood pressure cuff in the nonparetic arm.<br><br>Group 2: placebo control group (n = 13), standard medical management plus sham RIC. Sham RIC consisted 4 cycles of intermittent limb ischaemia; alternating 5 minutes' inflation (30 mmHg) and 5 minutes' deflation performed manually using a standard upper arm blood pressure cuff in the nonparetic arm.<br><br>Concomitant medications: single antiplatelets, blood pressure and cholesterol reduction treatment
Outcomes	Primary outcome: tolerability and feasibility of RIC after acute ischaemic stroke

	Secondary outcomes: surrogate markers of efficacy (plasma S100-Î², matrix metalloproteinase-9, troponin T), inflammation (C-reactive protein), and other putative biomarker (heat shock proteins and endocannabinoids). Transcranial Doppler was performed as a continuous beat-to-beat recording during the intervention; central pressures measured with Sphygmocor. Serious adverse events, mRS score, impairment (NIHSS score and motricity index), BI, extended activities of daily living, Zung Depression Scale, and cognition (MMSE) were measured at day 90.
Notes	External blinded review of outcomes<br><br>Follow-up: 100%<br><br>Demographic: adequately reported and similar<br><br>Principle investigator: Timothy J England, PhD, FRCP<br><br>Funding: British Medical Association Vera Down Grant<br><br>Notable authors' disclosures: no

Risk of Bias[29]

Bias	Author's Judgment	Support for Judgement
Random sequence generation	Low risk	The study minimised on age, sex, NIHSS score, and systolic blood pressure.
Allocation concealment	Low risk	Web-based, and 1:1 allocation
Blinding of participants and personnel	Low risk	The intervention group received RIC procedure and the control group received sham procedure.
Blinding of outcome assessment	Low risk	This was a single-centre, randomised, outcome-blinded, placebo-controlled trial.
Incomplete outcome data	Low risk	No missing outcome data
Selective reporting	Low risk	The study protocol was available and all of the study's prespecified outcomes had been reported in the pre-specified way.
Other bias	Low risk	No other bias identified

Ginsberg (2013)

Characteristics[30]

Methods	RCT

	Centralised step-forward, web-based 1 : 1 randomisation<br><br>All study personnel and participants were blinded
Participants	People aged 18-83 years with acute ischaemic stroke within 5 hours of stroke onset and within 60 minutes of intravenous tPA<br><br>Diagnosis of ischaemic stroke by CT or MRI scan
Interventions	25% albumin therapy (2 g/kg intravenously administered over 120 minutes) versus isovolaemic 0.9% normal saline
Outcomes	Death<br>Neurological score<br>Functional score
Notes	Neurological death reported at 7 days<br><br>Death from any cause reported at 30 and 90 days

Risk of Bias[30]

Bias	Author's Judgment	Support for Judgement
Random sequence generation	Low risk	Quote: "Centralized step-forward, web-based 1:1 randomisation process with a biased coin minimisation approach that accounted for the status of treatment group balance within and across sites"
Allocation concealment	Low risk	Quote: "Centralized step-forward, web-based 1:1 randomization process"
Blinding of participants and personnel	Low risk	Quote: "All study personnel and patients were masked to the identity of the study drug. Each sealed kit contained two bottles (initially of 500 mL and 250 mL, then, for manufacturing reasons, both of 500 mL) of the same substance (either albumin or saline) encased in cardboard blinding boxes similar to those used in the Saline versus Albumin Fluid Evaluation (SAFE) Trial, in addition to filters and opaque sheathing to conceal the intravenous tubing. ... A bedside nurse or other personnel not associated with the trial administered the study drug (8 mL/kg estimated bodyweight) by constant intravenous infusion

		over 2 h (plus or minus 15 min)"
Blinding of outcome assessment	Low risk	Quote: "All study personnel and patients were masked to the identity of the study drug"
Incomplete outcome data	Low risk	Albumin: 3 participants withdrew, 11 participants lost to follow-up<br><br>Control: 3 participants withdrew, 13 participants lost to follow-up, 10 other participants with 90-day assessment missing<br>ITT analysis
Selective reporting	Low risk	All outcomes reported
Other bias		

Glass (2004)

Characteristics[31]	
Methods	Randomised controlled trial<br>Centralised randomisation by computer-generated random numbers and remote telephone allocation
Participants	Stroke patients recruited from inpatient stroke unit care within 30 days of event (291 participants)<br><br>Exclusion criteria: age less than 45, resident out with area, terminally ill, severe communication problems, cognitive impairment, poor English language, institutional care, social isolation Inclusion criteria: confirmed stroke of mild or moderate severity, competent to consent
Interventions	Intervention: intervention was provided by a clinical psychologist or a social worker who were formally trained; the emphasis of the intervention was on recruiting families and naturally occurring social networks rather than formal or community-care based services; 15 intervention visits were made according to protocol (approximately 90 minutes in duration)<br><br>Control: the control group received usual care (not defined)
Outcomes	Outcome assessment was conducted at 3 and 6 months by a blinded outcome assessor<br><br>Patient outcome measures: included activities of daily living (Barthel, instrumental activities of daily living), dependency (physical performance test), mood (Centre for Epidemiological Studies Depression Scale CESD), cognition (a cognitive summary score), perceived health status (self-rated health and quality of life)
Notes	Involvement of a carer was not compulsory
Risk of Bias[31]	

Bias	Author's Judgment	Support for Judgement
Random sequence generation		
Allocation concealment	Low risk	A - Adequate
Blinding of participants and personnel		
Blinding of outcome assessment		
Incomplete outcome data		
Selective reporting		
Other bias		

Globas (2011)

Characteristics[18]	
Methods	RCT<br>Method of randomisation: computer-based<br>Blinding of outcome assessors: not blinded<br>Adverse events: 1 recurrent stroke (EXP group)<br><br>Dropouts: 2 (2 in EXP group, 0 in CTL group)<br>ITT: stated by the trialists
Participants	Country: Switzerland and Germany<br>38 participants (20 in EXP group, 18 in CTL group)<br>Ambulatory at study onset<br>Mean age: 69 years (both CTL and EXP groups)<br><br>Inclusion criteria: hemiparetic gait as evaluated by a neurologist with at least 1 clinical sign for paresis, spasticity or circumduction of the affected leg while walking, and the ability to walk on the treadmill at ≥ 0.3 km/hour for 3 minutes with handrail support<br><br>Exclusion criteria: unstable angina pectoris, heart failure (New York Health Association > II°), haemodynamically significant valvular dysfunction, peripheral arterial occlusive disease, dementia (MMSE < 20), aphasia (unable to follow 2 commands), major depression (CES-D > 16), and other medical conditions precluding participation in aerobic exercise, as well as participants already performing aerobic exercise training for > 20 minutes per day and > 1 day per week
Interventions	3 months (3 times per week) progressive graded, high-intensity aerobic treadmill exercise (TAEX) or conventional care physiotherapy
Outcomes	• peak VO2 during maximum effort treadmill walking<br>• walking ability measured in 6-minute walks<br>• 10-Metre Walk Test at comfortable (self-selected) and maximum walking speeds<br>• functional leg strength, the 5-Chair-Rise (5CR)<br>• Berg Balance Scale<br>• self rated mobility and activities for daily living function assessed by the Rivermead Mobility Index (RMI)<br>• physical and mental health measured by the Medical Outcomes Study Short-Form 12 (SF-12)

Notes	

Risk of Bias[18]

Bias	Author's Judgment	Support for Judgement
Random sequence generation	Low risk	A computer-based pseudo random number generator and the Moses–Oakford assignment algorithm were used to develop the randomisation schedule
Allocation concealment	Low risk	The procedure was performed by independent study staJ at the Department of Biostatistics, University of Ulm, Germany
Blinding of participants and personnel		
Blinding of outcome assessment	High risk	No blinding of outcomes was done
Incomplete outcome data		
Selective reporting		
Other bias		

Granger (2011)

Characteristics[20]

Methods	Randomised, double-blind, active controlled trial
Participants	18,201 people with documented AF or atrial flutter and at least 1 additional risk factor for stroke: at least 75 years old; previous stroke, TIA or systemic embolic event; symptomatic heart failure within previous 3 months or left ventricular ejection fraction of no more than 40%; diabetes mellitus; hypertension requiring pharmacologic treatment
Interventions	Apixaban (5 mg twice daily, or 2.5 mg twice daily in participants with at least 2 or more of the following criteria: age at least 80 years, body weight of no more than 60 kg, or serum creatinine level of 1.5 mg/dl or more; n = 9120) versus dose-adjusted warfarin (target INR 2.0 to 3.0; n = 9081)
Outcomes	Primary efficacy outcome: composite of stroke or systemic embolic events<br><br>Secondary efficacy outcomes: death from any cause, myocardial infarction<br><br>Primary safety outcome: major bleeding (ISTH criteria)<br><br>Secondary safety outcomes: composite of major bleeding and clinically relevant non-major bleeding; any bleeding; other adverse events; liver function abnormalities
Notes	Study co-sponsored by Bristol-Myers Squibb and Pfizer

Risk of Bias[20]

Bias	Author's Judgment	Support for Judgement

Random sequence generation	Low risk	Participants were randomly assigned to treatment groups
Allocation concealment	Low risk	Participants were randomly assigned to treatment groups. Stratification by clinical site and prior VKA use
Blinding of participants and personnel	Low risk	Double-blind, double-dummy design
Blinding of outcome assessment	Low risk	Efficacy and safety outcomes were adjudicated by a clinical events committee whose members were not aware of study group assignments
Incomplete outcome data	Unclear risk	Efficacy and safety outcomes analysed in ITT population. Number of participants that discontinued during study and reasons are reported. Number of participants with missing data on vital status rather high (n = 380; 2.1%) and could have had an impact on the robustness of the mortality data from this study
Selective reporting	Low risk	All predefined efficacy and safety outcomes reported for ITT population
Other bias	Low risk	

Gray (2007)

Characteristics[32]	
Methods	Multicentre, randomised controlled trial<br>Blinded outcome assessments<br>Randomisation: first 571 patients sealed envelopes, the rest by central randomisation service<br>ITT analysis
Participants	UK, 933 patients<br>T: 464, C: 469<br>Mean age: 75 years<br>Male: 45%<br>Inclusion: acute ischaemic stroke or primary intracerebral haemorrhage with admission venous plasma glucose 6 to 17 mmol/L<br>Enrolment within 24 hours of stroke onset
Interventions	T: 500 ml GKI (of 10% dextrose, 20 mmol potassium chloride and 16U soluble recombinant human insulin) continuous iv infusion<br>C: 0.9% normal saline<br>Rx: 24 hours

Outcomes	Death at 90 days, European stroke scale score, OCSP subtype, Glasgow Coma Scale at baseline Barthel index, mRS at 30 and 90 days
Notes	Ex: SAH, isolated posterior circulation syndromes no physical disability, pure language disorders, renal failure, anaemia, coma, established history of insulin treated diabetes, previous disabling stroke, dementia or symptomatic cardiac failure

Risk of Bias[32]

Bias	Author's Judgment	Support for Judgement
Random sequence generation	Low risk	Probably done
Allocation concealment	Low risk	Treatment allocation was concealed
Blinding of participants and personnel	High risk	Probably not done
Blinding of outcome assessment	High risk	Probably not done
Incomplete outcome data	High risk	Probably not done No loss of follow up for death at 90 days Day 90 mRS missing for 5 patients Day 90 Bartel Index missing for 30 patients
Selective reporting		
Other bias		

Gurm (2008)

Characteristics[33]

Methods	• Multicentre randomised non-inferiority trial conducted in the USA (29 centres)<br>• No predefined sample size; recruitment was planned to stop if non-inferiority of stenting was demonstrated at repeated interim analyses. 334 patients randomly assigned to endovascular treatment or endarterectomy (n = 167 in each arm) between August 2000 and July 2002
Participants	• Inclusion criteria: age ≥ 18 years, symptomatic stenosis of ≥ 50% or asymptomatic stenosis of ≥ 80% according to NASCET criteria documented by duplex ultrasound; high surgical risk defined by at least one of the following criteria: significant cardiopulmonary disease, contralateral carotid occlusion, contralateral laryngeal nerve palsy, previous radical neck surgery or radiation therapy to the neck, recurrent stenosis after endarterectomy, age > 80 years<br><br>• Exclusion criteria: ischaemic stroke within the previous 48 hours, intraluminal thrombus, vascular disease precluding endovascular treatment, intracranial aneurysm, need for more than 2 stents, history of bleeding disorder, planned percutaneous or surgical intervention, life expectancy <1 year, ostial lesion in the common carotid or brachiocephalic artery<br><br>• Mean age 72.5 years, 67% male. 71% had asymptomatic carotid stenosis

Interventions	• Endovascular treatment consisted of insertion of a stent (Smart or Precise, Cordis, Johnson & Johnson, USA) with routine use of a distal protection device (Angioguard or Angioguard XP, Cordis, Johnson & Johnson, USA). Patients received 81 or 325 mg aspirin daily starting at least 72 hours before stenting and indefinitely thereafter, as well as 75 mg clopidogrel daily starting 24 hours before stenting until 2 to 4 weeks after treatment. Interventionalists had performed a median of 64 carotid stent procedures before joining the trial (range 20 to 700)<br><br>• Endarterectomy was performed according to customary techniques. Patients received 81 or 325 mg aspirin daily starting at least 72 hours before endarterectomy and indefinitely thereafter. Surgeons had median annual volumes of 30 carotid endarterectomies (range 15 to 100) and had to demonstrate peri-procedural stroke or death rates of < 6% prior to joining the trial<br><br>• All patients were given heparin during the procedure
Outcomes	• Method of comparison: intention-to-treat and per-protocol<br><br>• Non-inferiority margin (primary safety outcome or its 30-day components): 95% confidence interval of risk difference below 3.0% (type 1 error probability one sided 2.5%)<br><br>• Primary outcome: death, any stroke, or MI within 30 days aftertreatment or death or ipsilateral stroke between 31 days and 1 year after treatment<br><br>• Secondary outcomes relevant for this review included components of the primary outcome, major and minor stroke, cranial nerve palsy, and death or any stroke within 30 days after treatment or ipsilateral stroke or death from neurologic causes up to 3 years after treatment
Notes	• Terminated early due to a drop in the randomisation rate

Risk of Bias[33]

**Bias**	**Author's Judgment**	**Support for Judgement**
Random sequence generation	Low risk	* Quote: "Randomization was performed with the use of a pseudo random-number generator, and the numbers were distributed by an automated, centralized telephone response system"<br><br>* Comment: adequate
Allocation concealment	Low risk	* Quote: see above<br>* Comment: adequate
Blinding of participants and personnel	Low risk	* Comment: blinding not possible, but the outcome is not likely to have been influenced
Blinding of outcome assessment	Low risk	* Outcome assessment was not blinded but done by neurologists who were not

		involved in the procedures. Outcome events were centrally adjudicated blinded to treatment<br><br>* Comment: influence on outcome judged unlikely
Incomplete outcome data	Low risk	* 143 of 167 patients (86%) in the endovascular arm and 117 of 167 patients (70%) in the surgical arm had available follow-up at 3 years<br><br>* Numbers of patients at risk during follow-up provided<br><br>* Comment: censoring assumed to be non-informative
Selective reporting	Unclear risk	* Protocol was published only a few months before the initial results of the trial<br><br>* Expected major outcome events were reported, but not for symptomatic and asymptomatic patients separately<br><br>* Concern has been expressed about the fact that the Chief Investigator of SAPPHIRE received undeclared royalties from sales of the protection device used in the trial<br><br>* Comment: bias possible
Other bias		

Ha (2010)

Characteristics[23]	
Methods	Computer-based randomisation<br>Blinding unknown<br>Baseline prognostic factors were balanced between treatment groups
Participants	1 centre in Norway<br>170 patients < 3 days of acute stroke<br>5 excluded after randomisation, 41 lost to follow-up (22 died, 19 refused to participate in follow-up)<br>Mean age 79 years

Interventions	Rx: individualised nutritional treatment (n = 58)<br>C: routine care (n = 66)
Outcomes	Primary: percentage of patients with weight loss > 5%<br>Secondary: quality of life, hand grip strength, length of hospital stay
Notes	Exclusions: stroke diagnosis unclear, critically ill, severe dementia, could not be weighed, planned discharge < 24 hours after the first visit by trial assessor

Risk of Bias[23]

Bias	Author's Judgment	Support for Judgement
Random sequence generation	Low risk	The sequence of treatment allocation was prepared from a computer-generated randomisation list by a person not involved in patient assessments
Allocation concealment	Low risk	Patients randomised to individualised, nutritional treatment or to routine care in blocks of 20 patients using sequentially numbered, non-transparent envelopes containing the treatment allocation information
Blinding of participants and personnel	Unclear risk	All outcomes: Blinding unknown
Blinding of outcome assessment	Unclear risk	All outcomes: Blinding unknown
Incomplete outcome data	High risk	41 lost to follow-up (22 died, 19 refused to participate in follow-up)
Selective reporting	Low risk	
Other bias	Unclear risk	

Hacke (2006)

Characteristics[33]

Methods	• Multicentre, randomised non-inferiority trial (35 centres in Germany, Austria and Switzerland)<br><br>• Planned sample size 1900 patients, revised upwards to 2500 after interim analysis; 1214 patients randomly assigned to endovascular treatment (n = 613) or endarterectomy (n = 601) between March 2001 and February 2006
Participants	• Inclusion criteria: symptomatic carotid stenosis of ≥ 50% according to NASCET criteria or ≥ 70% according to ECST criteria, documented by duplex ultrasound (most recent event in the last 180 days before randomisation), age > 50 years, modified Rankin score ≤ 3<br><br>• Exclusion criteria: intracranial bleeding in the previous 90 days, uncontrolled hypertension, intracranial arteriovenous malformation or

	aneurysm, life expectancy < 2 years, coagulation abnormality, contraindications for heparin, aspirin, or clopidogrel, planned surgery, stenosis due to dissection, irradiation, or occurring after previous revascularisation, floating thrombus, intracranial tandem stenosis of higher degree<br><br>• Mean age 69 years, 72% male<br><br>• 62% of patients had ≥ 70% degree of stenosis
Interventions	• Endovascular treatment consisted of stent insertion. Choice of balloon size, pre-dilation, and use of protection devices were at the discretion of the interventionalist. Used stents and protection devices had to be CE-certified and approved for use in the study by a separate committee. Patients had to be given 100 mg aspirin plus 75 mg clopidogrel daily for 3 days before and 30 days after treatment. Interventionalists had to show proof of at least 25 consecutive successful angioplasty or stent procedures in the carotid artery<br><br>• Carotid endarterectomies were performed by vascular surgeons who needed to have 25 consecutive procedures completed. Surgeons used their routine techniques without specifications on type of anaesthesia or shunt use. Patients had to be given at least 100 mg aspirin before, during, and after surgery
Outcomes	• Primary method of comparison: intention-to-treat<br><br>• Non-inferiority margin (primary safety outcome): 90% confidence interval of risk difference below 2.5% (type 1 error probability one-sided 5%)<br><br>• Primary safety outcome: death or ipsilateral stroke between randomisation and 30 days after treatment<br><br>• Secondary safety outcomes relevant for this review included death or any stroke, death or disabling stroke, death, and any stroke between randomisation and 30 days after treatment<br><br>• Primary efficacy outcome: death or any stroke between randomisation and 30 days after treatment, or ipsilateral stroke up to 2 years after randomisation<br><br>• Secondary efficacy outcomes relevant for this review included death or any stroke, and carotid restenosis within 2 years after randomisation
Notes	• Terminated early by the steering committee due to futility concerns and lack of funding

Risk of Bias[33]

**Bias**	**Author's Judgment**	**Support for Judgement**
Random sequence generation	Low risk	• Quote: "The random allocation schedule was generated using a computer program. This was done by members of the data and statistic centre, who also obtained and analysed the data."

		• Comment: adequate
Allocation concealment	Low risk	• Quote: see above<br>• Comment: adequate
Blinding of participants and personnel	Low risk	• Comment: blinding not possible, but the outcome is not likely to have been influenced
Blinding of outcome assessment	Low risk	• Outcome assessment was not blinded but done by neurologists who were not involved in the procedures. Outcome events were centrally adjudicated blinded to treatment<br><br>• Comment: influence on outcome judged unlikely
Incomplete outcome data	Low risk	• 6 and 12 patients in the endovascular and surgical arms, respectively, immediately withdrew consent after randomisation<br><br>• 541 (89%) patients in the endovascular arm and 522 (89%) patients in the surgical arm had follow-up data available at 2 years. Reasons for attrition provided. Numbers of patients at risk during follow-up provided<br><br>• Comment: No information on clinical outcome is available from patients who withdrew consent and were excluded from the analysis in the trial; however, the numbers are small and the risk of bias is considered low. Censoring assumed to be non-informative
Selective reporting	Low risk	• Comment: the primary analysis prespecified in the protocol was reported
Other bias		

Hacke (2009)

Characteristics[34]

Methods	Randomised, placebo-controlled, double-blind, dose ranging study phase III study<br><br>Testing the effect of 2 doses of desmoteplase at 3 to 9 hours post stroke<br><br>Computer-generated randomisation codes, stratified by centre, double-blinded
Participants	Multicentre trial (Europe, North America, Australia, China) June 2005 to March 2007<br><br>3 to 9 hour post onset with > 20% potentially salvageable tissue as defined by perfusion study (CT or MR)<br><br>18 to 85 years old, NIHSS 4 to 24
Interventions	Participants randomised to 90 µg/kg (N = 57) or 125 µg/kg (N = 66) of desmoteplase or placebo (N = 63) 1 intravenous bolus over 1 to 2 minutes
Outcomes	Good clinical outcome at 90 days = improvement in NIHSS > 8 points and BI 75 to 100 and mRS 0 to 2<br><br>Intracranial haemorrhage (symptomatic or asymptomatic), major haemorrhagic event, death<br><br>Change in infarct volume between baseline and 30 days
Notes	Baseline study and follow-up by same modality at 30 days. CT at 24 to 72 hours to assess for haemorrhage<br><br>Less severe strokes than DIAS 2005/DEDAS studies

Risk of Bias[34]

Bias	Author's Judgment	Support for Judgement
Random sequence generation	Low risk	Central randomisation
Allocation concealment	Low risk	Central randomisation
Blinding of participants and personnel	Low risk	Double-blind
Blinding of outcome assessment	Low risk	Double-blind
Incomplete outcome data	Low risk	
Selective reporting		
Other bias		

Harold (2008a)

Characteristics[35]

Methods	C = central randomisation, stratified<br>Double blind<br>Exclusions during trial: 2 (Rx)<br>Losses to follow up: 3 (2 Rx, 1 control)
Participants	112 sites in Australia, Europe, South Africa, South and North America

	Companion cohort<br>Ischaemic stroke within 5 to 6 hours after onset<br><br>319 participants (160 assigned Abciximab), between December 2003 and September 2005<br>173 (54%) male<br>Mean age: 69 years<br>100% CT before entry<br>NIHSS score: 4 to 22
Interventions	Rx: Abciximab 0.25 mg/kg intravenous bolus followed by a 0.125 mg/kg/min infusion for 12 hours Control: intravenous placebo<br><br>Duration: 12 hours
Outcomes	Death at 3 months<br>ICH (symptomatic and systemic) at 5 days and 3 months<br>Extracranial haemorrhage at 5 days and 3 months<br>Thrombocytopenia at 5 days<br>Functional outcome (mRS and BI) at 3 months<br>Neurological recovery (NIHSS) at 5 days and 3 months
Notes	Ex: specified by protocol - similar to other acute stroke treatment trials<br>Participants that might be treated with intravenous rt-PA were excluded<br>FU: 3 months<br>The trial was terminated prematurely, after 808 participants of the 1800 planned had been included, due to an unfavourable benefit-risk profile<br><br>Funding source: supported by Eli Lilly and Company, and Centocor Research and Development

Risk of Bias[35]

Bias	Author's Judgment	Support for Judgement
Random sequence generation		
Allocation concealment	Low risk	Assignment of treatments was performed via an interactive voice response system
Blinding of participants and personnel	Low risk	Participants and personnel were given identically appearing 5-mL vials of Abciximab or placebo, the 2 study treatments were administered with the same modality
Blinding of outcome assessment	Low risk	Assessors did not know the allocated interventions
Incomplete outcome data	Low risk	All analyses for efficacy were performed on the intention-to-treat population regardless of whether study medication was administered

	Unclear risk	Safety analyses were not performed on the intention-to-treat population but on participants who received at least some study treatment, even if not randomised
Selective reporting	Low risk	No suggestion of selective reporting
Other bias		

Harold (2008b)

Characteristics[36]	
Methods	C = central randomisation, at a ratio of 1:1 (abciximab or placebo), stratified according to NIHSS (4 to 7, 8 to 14, and 15 to 22), study population (primary or companion), clinical site, and time since onset<br>Double blind<br>Exclusions during trial: 2 (Rx)<br>Losses to follow up: 3 (2 Rx, 1 control)
Participants	Companion cohort<br>International<br>319 participants<br>173 (54%) male<br>Mean age: 69 years<br>100% CT before entry<br>Ischaemic stroke within 5 to 6 hours after onset<br>NHISS 4 to 22
Interventions	Rx: abciximab bolus 0.25 mg/kg plus infusion of 0.125 mcg/kg/min (maximum 10 mcg/min) for 12 hours<br>Control: placebo<br>Duration: 12 hours
Outcomes	Death at 3 months<br>Intracranial haemorrhage (symptomatic and systematic) at 5 days and 3 months<br>Extracranial haemorrhage at 5 days and 3 months<br>Thrombocytopenia at 5 days<br>Functional outcome (modified Rankin scale and Barthel Index) at 3 months<br>Neurological recovery (NIHSS) at 5 days and 3 months
Notes	Ex: specified by protocol - similar to other acute stroke treatment trials<br>Patients that might be treated with intravenous recombinant tissue plasminogen activator were excluded<br>FU: 3 months<br>The trial was terminated prematurely due to an unfavourable benefit-risk profile

Risk of Bias[36]

**Bias**	**Author's Judgment**	**Support for Judgement**
Random sequence generation		
Allocation concealment	Low risk	A - Adequate

Blinding of participants and personnel	
Blinding of outcome assessment	
Incomplete outcome data	
Selective reporting	
Other bias	

Harwood (2012)

Characteristics[37]	
Methods	RCT
Participants	Adults with stroke, living in community (Maori and Pacific Islander)
Interventions	Overall:<br><br>• intended audience: stroke survivors and their family<br>• theoretical rationale: need for development of self support after discharge for these ethnic group/s<br>• mode: 1-to-1<br>• personnel: trained RA from same ethnic group<br>• delivery method: face-to-face; completing booklet<br>• language: not stated but stories relevant to ethnic groups<br>• content/topics covered: individualised assessment (risk factor and ADL), process of recovery, self<br>identify progress, goal-setting<br>• duration: ongoing/as needed<br><br>Intervention 1: DVD - inspirational stories and advice from same ethnic group: n = 48<br>• 80-minute DVD with encouragement to listen as often as participant wished<br><br>Intervention 2: TCS: n = 46<br>• 80 minutes, individual assessment and goal setting with booklet<br><br>Intervention 3: DVD and TCS: n = 39<br>• combination of intervention 1 and 2<br><br>Control: usual care<br>• single 30-minute education session with standard written information about stroke (not Maori/Islander specific)
Outcomes	Primary: SF-36<br><br>Secondary: BI, FAI, Carer Strain Index, mRS, use of rehabilitation services<br><br>Assessed at 6/12 and 12/12
Notes	Included unpublished data from author: mean scores SF-36, BI, FAI

Risk of Bias[37]

**Bias**	**Author's Judgment**	**Support for Judgement**

Random sequence generation	Low risk	Random numbers table with stratification by ethnic group
Allocation concealment	Low risk	Opaque sealed envelopes to conceal allocation
Blinding of participants and personnel	High risk	Neither able to be blinded to group
Blinding of outcome assessment	Low risk	Assessments by RAs masked to allocation
Incomplete outcome data	High risk	Missing data were not missing at random
Selective reporting	Low risk	Only 12/12-month data in journal article, authors supplied 6/12 data
Other bias	Low risk	n/a

Hedegaard (2014)

Characteristics[10]	
Methods	RCT<br><br>Unit of randomisation: participant
Participants	Place of recruitment: emergency ward or from 5 locations - 2 inpatient wards,1 patient hotel, 1 rehabilitation centre and 1 TIA outpatient clinic<br><br>Numbers randomised: total: 211 (I: 104; C: 107)<br><br>% Completing final follow-up: 96%<br><br>Inclusion criteria: ischaemic stroke or TIA within the previous 30 days, acute first stroke, > 18 years of age, prescribed at least 1 antiplatelet or anticoagulant medication, participant or co-habiting relatives dispensed the participant's medications<br><br>Exclusion criteria: lives in a care home or institution, dose dispersed medications from a pharmacy, if medication was dispensed by a home nurse, terminal illness or cognitive/physical impairment<br><br>Type of stroke: TIA (I: 47%; C: 49%); ischaemic stroke (I: 52%; C: 50%)<br><br>Mean age (range): I: 64 (56-73), C: 68 (61-73)<br><br>Gender (% men): I: 59.8; C: 62.4<br><br>Ethnicity: not reported<br><br>Socio-economic or socio-demographic status: not reported
Interventions	Intervention details (components, length, frequency): clinical pharmacists were trained in providing<br>1) a focused medication review followed by dialogue based on motivational interviewing to support adherence and lifestyle changes;

	2) a patient interview followed by a list of their own goals and agreed actions;<br>3) 3 follow-up telephone calls to the participant (1 week, 2 months and 6 months) where participants were given a written summary of their goals and plans after the second and third calls<br><br>Location: outpatient clinic<br><br>Mode of delivery: outpatient appointment and telephone follow-up<br><br>Personnel responsible for delivery: pharmacists<br><br>Timing post-stroke: within 30 days<br><br>Usual care (I and C): usual care without the clinical pharmacist. 2 months after the start of the study, a secondary prevention clinic was initiated for all participants with follow-up from a stroke specialist nurse, including baseline risk factor assessment, medication adherence and lifestyle behaviour at day 14 and 3 months
Outcomes	Overall adherence to thrombo-preventative regimen in the year after hospitalisation based on the medication adherence ratio
Notes	Analysis method: exploratory per-protocol analysis<br><br>Risk of bias: unclear

Risk of Bias[10]

**Bias**	**Author's Judgment**	**Support for Judgement**
Random sequence generation	Low risk	Performed by clinical trial group at hospital pharmacy. 1:1 allocation, randomised in blocks of 4 and 6 by computer prior to enrolment and concealed in opaque envelopes
Allocation concealment	Low risk	Central allocation with opaque envelopes
Blinding of participants and personnel		
Blinding of outcome assessment		
Incomplete outcome data	Unclear risk	The study did not address this outcome
Selective reporting	Unclear risk	Insufficient information to permit judgement. Protocol available
Other bias	Low risk	The study appears to be free of other sources of bias

Herisson (2016)

Characteristics[38]

Methods	Prospective multicenter RCT testing 2 sitting procedures at the acute phase of ischaemic stroke
Participants	Acute ischaemic stroke patients recruited from 11 centres in the North West region of France<br><br>Participants were: over 18 years age, had neurological deficits, haemorrhage excluded on imaging, enrolled in a healthcare plan (French social security)<br><br>Exclusions included; very mild (NIHSS < 3) or very severe stroke (NIHSS > 22), reduced consciousness Glasgow Coma Score < 13), fluctuating neurological signs (history of worsening linked to an upright positioning), known symptomatic intra-cranial stenosis > 50%, vomiting or difficulty in breathing, contraindication for sitting, e.g. deep vein thrombosis or lower limb fracture, prior dependency Rankin score 3 to 6, anticipated difficulty in follow-up<br><br>167 participants: 82 intervention, 85 control<br><br>Mean age: 70 years<br>89/138 (64%) men<br><br>0/138 (0%) haemorrhagic<br><br>Mean NIHSS 7.5<br><br>76 (56%) had mild stroke (Rankin score 0 to 3)<br><br>45 (34%) moderate stroke (Rankin score 4)<br><br>13 (10%) severe stroke (Rankin score 5)
Interventions	**What**<br><br>This study aimed to test 2 different protocols for sitting in acute ischaemic stroke patients.<br><br>Early protocol: participants seated out of bed at the earliest time possible, ideally the day of stroke onset (day 0) and no later than the calendar day after stroke onset<br><br>Progressive protocol: the participant would be positioned in bed at 30°, 45° the day after (day 1), and 60° at day 2, and sitting out of bed at day 3 (which corresponds to the first sitting in this group). Those angles reflect the position of the upper body relative to the bed (and floor)<br><br>**Who provided**<br><br>The physiotherapist or the nurses were in charge of collecting the data (e.g. blood pressure, tolerance) related to the protocol<br><br>**How**

For both protocols, minimal duration of the first sitting was 15 minutes. The procedure could be continued, depending on participant fatigue and tolerance (60 minutes maximum). The physiotherapist or the nurses were in charge of collecting the data (blood pressure, tolerance. . .) related to it. Sitting posture (legs dangling or feet positioned on a foot rest), was done as usual, in keeping with each unit's protocol. The use of a lifter, when necessary, was allowed

Where

Stroke centres of 11 hospitals in North West France

When and how much

For both protocols, minimal duration of the first sitting was 15 minutes. The procedure could be continued, depending on patient fatigue and tolerance (60 minutes maximum). Sitting was repeated on a daily basis according to initial tolerance of the procedure, as approved by the physician in charge

Tailoring

Close monitoring of blood pressure and heart rate was performed: before the sitting procedure, immediately after, and 5 minutes after. While sitting, participants showing any sign of low tolerance, defined by neurological worsening (of current or new neurological deficits), vagal reaction (bradycardia or nausea), a greater than 40 mmHg increase of blood pressure topping 180/100 mmHg, or a symptomatic decrease in blood pressure, would be put back in bed. Sitting was repeated on a daily basis according to initial tolerance of the procedure, as approved by the physician in charge

Modifications

While sitting, participants showing any sign of low tolerance, defined by neurological worsening (of current or new neurological deficits), vagal reaction (bradycardia or nausea), a greater than 40 mmHg increase of blood pressure topping 180/100 mmHg, or a symptomatic decrease in blood pressure, would be put back in bed

How well

Planned: the early protocol intended that participants would be seated, out of bed, at the earliest time possible, no later than the calendar day after stroke onset. In the progressive protocol, the participant would be positioned in bed at 30°, 45° the day after (day 1), and 60° at day 2, and sitting out of bed at day 3 (which corresponds to the first sitting in this group)

Actual: time from stroke to the first sitting time was 1.1 ± 0.2 days in the early sitting group versus 3 ± 0.2 days in the progressive group

First sitting lasted significantly longer in the progressive group compared to the early group: 83.7 ± 94.7 minutes versus 56.6 ± 41.7 minutes respectively ($P < 0.05$). Tolerance of the sitting procedure was the same in the early and progressive sitting groups, with a prevalence of side effects of 14.5% and

	13.7%, respectively. Sitting was continued daily for both groups during hospitalisation in 96% of cases.<br><br>NB: a small amount of out-of-bed activity was incorporated in transferring between bed and chair, which started 2 days earlier in the intervention group than the standard care group<br><br>Physiotherapy and deep vein thrombosis prevention by low molecular weight heparin were performed as usual in each unit
Outcomes	The primary outcome measure was the proportion of mRS 0 to 2 at 3 months visit after stroke onset<br><br>Secondary outcomes were assessed at 7 days (or the day of discharge, if before 7 days), and the 3-month follow-up and included:<br><br>• NIHSS score, Rankin score, Barthel score;<br>• data about the tolerance of the sitting positioning (including prevalence of side effects that forced termination of the procedure);<br>• length of hospitalisation;<br>• complications that occurred during hospital stay were reviewed at 3 months using a multiple-choice list, and based on both participant interview and medical records;<br>• duration of sitting out of bed was calculated from the recorded time at which the participant was positioned seated out of bed to the time at which the participant would be put back in bed
Notes	SEVEL (Stroke and Early VErticaL positioning) study<br><br>clinicaltrials.org registration number NCT01573299<br><br>The enrolment period covered November 2011 to April 2014. The study ended prematurely (after 167 of a target of 400) as it became unviable, due to the slow recruitment rate

Risk of Bias[38]

Bias	Author's Judgment	Support for Judgement
Random sequence generation	Low risk	Quote: "The random sequence was generated by our statistician (CV) using the SAS software"
Allocation concealment	Low risk	Randomisation between the early and progressive sitting groups was performed via "numbered sealed envelopes that the investigator would draw from, in consecutive fashion (with blocks of 4 in 1:1 ratio, stratified by center) each time a patient was enrolled in the study"<br><br>Quote: "Data were reported online using a server dedicated to the study"

Blinding of participants and personnel	Unclear risk	Probably not blinded
Blinding of outcome assessment	High risk	Quote: "Evaluations were made during the intermediate time point at 7 days (or the day of discharge, if before 7 days) and at 3 months after stroke, by a neurologist from the same stroke unit, aware of the study and unblinded to the patient group assignment"
Incomplete outcome data	High risk	Substantial drop out (29/167; 17%) after randomization<br><br>Participants with major deviation to the protocol or serious adverse event that were enrolled but could not continue the study were assigned a Rankin score in the category 3 to 6
Selective reporting	Unclear risk	Primary outcome largely complete
Other bias		

Hill (2011)

Characteristics[30]	
Methods	RCT<br><br>Centralised step-forward, web-based 1 : 1 randomisation process<br><br>All study personnel and participants were blinded
Participants	People aged ≥ 18 years with acute ischaemic stroke within 5 hours of stroke onset and within 60 minutes of intravenous tPA<br><br>Diagnosis of ischaemic stroke by CT or MRI scan
Interventions	25% albumin therapy (2 g/kg intravenously administered over 120 minutes) versus isovolaemic 0.9% normal saline
Outcomes	Death<br>Neurological score<br>Functional score
Notes	Study suspended in 2007 after interim analysis of differences in overall death between groups<br><br>Neurological death reported at 7 days<br><br>Death from any cause reported at 30 and 90 days

Risk of Bias[30]

Bias	Author's Judgment	Support for Judgement
Random sequence generation	Low risk	Quote: "Centralized step-forward, web-based 1:1 randomisation process was used"
Allocation concealment	Low risk	Quote: "Centralized step-forward, web-based"
Blinding of participants and personnel	Low risk	Quote: "All study personnel and patients were blinded"
Blinding of outcome assessment	Low risk	Quote: "All study personnel and patients were blinded"
Incomplete outcome data	Low risk	Albumin: 8 participants did not receive allocated intervention, 9 participants lost to follow-up<br><br>Control: 2 participants did not receive allocated intervention, 2 participants lost to follow-up<br><br>ITT analysis
Selective reporting	Low risk	All outcomes reported
Other bias		

Hofmeijer (2009)

Characteristics[39]

Methods	Prospective, randomised, controlled open trial
Participants	64 patients < 60 years of age, all had an infarct involving > 2/3 of MCA territory with evidence of mass effect and midline shift as well as changes in the level of consciousness as demonstrated by a GCS < 13<br><br>32 patients were randomised to each group<br><br>Patients were excluded if they had an ischaemic stroke of the whole cerebral hemisphere, decrease in consciousness partially because of causes other than the formation of oedema, such as metabolic, disturbances or medication, pupils fixed and dilated, thrombolysis in the 12 hours before randomisation, coagulopathy, pre-stroke score on the mRS > 1 or < 95 on the BI, life expectancy less than 3 years or other serious illness that might confound treatment assessment
Interventions	Medical treatment alone versus medical treatment plus surgical decompression performed < 96 hours from symptom onset and < 3 hours from randomisation

	Medical treatment included the use of osmotic agents on schedule to a target osmolality 315 to 320 mOsm, mechanical ventilation, blood pressure control, temperature and glucose control maintaining euvolemia<br><br>Surgical decompression consisted of removal of a flap of bone of at least 12 cm in diameter including frontal, parietal, temporal and occipital bones<br><br>The dura was opened and a dural patch was placed<br><br>Infarcted brain tissue was not resected
Outcomes	Primary effect measure: favourable outcome mRS ≤ 3 at 1 year<br><br>Secondary effect variable: favourable outcome defined as mRS score of 0 to 4, case fatality, functional dependence expressed as BI, symptoms of depression measured by the Montgomery and Asberg depression rating scale (MADRS), and quality of life measured with the Medical Outcomes Study 36-item short-form health survey (SF-36) and a visual analogue scale (VAS)15 at 1 year
Notes	The study was stopped prematurely as the Data Monitoring Committee established that no statistically significant difference would be found between both arms with the pre-established sample size

Risk of Bias[39]

Bias	Author's Judgment	Support for Judgement
Random sequence generation		
Allocation concealment	High risk	
Blinding of participants and personnel	Low risk	An unblinded investigator provided narrative description that was submitted to three blinded investigators to ascertain mRS The final score was obtained by consensus from these blinded observers
Blinding of outcome assessment	Low risk	An unblinded investigator provided narrative description that was submitted to three blinded investigators to ascertain mRS The final score was obtained by consensus from these blinded observers
Incomplete outcome data		
Selective reporting		
Other bias		

Hofstad (2013)

Characteristics[40]

Methods	Subgroup of Bergen 2014 who received their treatment in a community day unit<br><br>RCT comparing two different ESD models with treatment as usual
Participants	Patients admitted to the stroke unit (Department of Neurology, Haukeland University Hospital, Bergen) who were living at home in the Municipality of Bergen prior to having a stroke, had a stroke within the previous 7 days, and were admitted to the stroke unit within the previous 5 days. NIHSS score of 2 to 26 of a 13 items Norwegian version (range 0 to 34). Patients with NIHSS score < 2 were included if the mRS score was > 1. The patients had to be awake and able to agree to participation in the study by signing an informed consent, either themselves or by their relatives<br><br>At recruitment (2008 to 2011) characteristics were:<br><br>Average 72 years (range 27 to 98), 169 (55%) men, baseline BI 95 (SD 40), baseline NIHSS 3 (SD 4)<br><br>On average 306/1736 (18%) of screened patients were included
Interventions	Patients in 2 of the 3 study arms were treated according to the ESD concept. They were followed-up by a designated multi-disciplinary ambulatory team consisting of a nurse, a physiotherapist, and an occupational therapist from soon after admission to the stroke unit until shortly after discharge to home. This team originated from the rehabilitation department and served as a co-ordinating link between the patient, relatives, hospital personnel, and the personnel in primary health care. The team was particularly important in the discharge process and co-operated closely with the municipal health care in the planning and implementation of further treatment after discharge.<br><br>The two ESD arms differed by the location of treatment:<br><br>ESD 1 group received their treatment in a community day unit; whereas ESD 2 group patients stayed in their homes with home visits from the community health team<br><br>Patients in the third study arm constituted a control group and were treated as usual without any intervention from the study, except outpatient appointments for testing. Treatment 'as usual' mainly comprised institutional stay if necessary and/or physiotherapy as needed in the municipality (0 to 2 hours per week). Patients in all 3 study arms received language therapy as needed, regardless of allocated arm<br><br>The patients in the two ESD arms were discharged to their homes as soon as possible. Patients in need of a longer in-patient treatment period than offered by the stroke unit were discharged to a municipal institution or rehabilitation department for a period before going home. All patients in the ESD arms were offered rehabilitative treatment by a multi-disciplinary community health team, consisting of a nurse, a physiotherapist, and an occupational therapist<br><br>The scheduled treatment period was 5 weeks and maximally 4 hours per day 5 days a week, but many patients did not comply with this

Outcomes	The primary study outcome was mRS at 6 months<br><br>Secondary outcomes included mRS at 3 months, NIHSS, Barthel ADL Index, and patient satisfaction (5-point Likert scale with 1 best) at 3 and 6 months
Notes	

Risk of Bias[40]

Bias	Author's Judgment	Support for Judgement
Random sequence generation	Unclear risk	"Participants were randomised according to a computer generated block randomisation list (six patients in each block; two for each study arm) and consecutively assigned to their groups in the same order as they were included into the study"
Allocation concealment	Unclear risk	"The randomisation list was kept by a study coordinator and was not known to any persons in the stroke unit"
Blinding of participants and personnel	Unclear risk	Not explicitly stated but probably not possible to blind participants and personnel
Blinding of outcome assessment	Unclear risk	"The testers were blinded for study arm and the patients were instructed not to reveal this information"
Incomplete outcome data	Unclear risk	77/306 (25%) were not retested at 6 months (22 Day Unit; 22 Home group; 33 control)
Selective reporting	Unclear risk	All pre-specified outcomes appear to have been reported
Other bias		

Holmgren (2010)

Characteristics[41]

Methods	Single-blind RCT
Participants	Sweden<br><br>34 participants: 15 RTT, 19 control<br><br>Participants were recruited from Umeå Stroke Unit. There were 3-monthly recruitment periods between February 2005 and June 2007

	Inclusion criteria: first-ever or recurrent ischaemic or haemorrhagic stroke 3 to 6 months before enrolment and randomisation, age ≥ 55, the ability to walk 10 metres with or without a walking device, the ability to understand and comply with instructions in Swedish, risk of fall at the time of enrolment according to subjective clinical observations in the assessment situation performed by the experienced physiotherapists in the study<br><br>Exclusion criteria: the ability to walk outdoors independently, i.e. without personal assistance or walking device, severe aphasia or severe vision or hearing impairment, a medical condition that a physician determined was inconsistent with study participation, e.g. cancer or severe congestive heart failure with expected short remaining life expectancy, recurrent stroke within 3 months before study start, living more than 100 km away from the training facilities<br><br>Mean age: RTT 77.7 years (SD 7.6), control 79.2 years (SD 7.5)<br><br>62% male<br><br>Stroke details: first or recurrent stroke, 97% ischaemic, 3% haemorrhagic<br><br>Time since stroke: RTT 139.7 days (SD 37.3), control 126.8 days (SD 28.2)<br><br>Pre-intervention functional activity level: Barthel Index: RTT 44.3 (CI 40.0 to 48.7), control 44.2 (CI 39.3 to 49.2)
Interventions	RTT intervention: the intervention was based on the HIFE (High Intensity Functional Exercise) program, to improve the participants lower-limb strength, balance and gait ability<br><br>The program includes lower-limb strength (e.g. chair stand) and balance exercises (e.g. weight shifting outside support surface), standing (e.g. knee bend) and walking (e.g. obstacle crossing course)<br><br>A home visit was conducted by a physiotherapist and an occupational therapist to determine each participant's ability to perform ADLs and lifestyle activities and to experience the participants daily difficulties in their own environment<br><br>Sessions were 45 minutes, 6 times per week (twice daily) for 5 weeks = 22.5 hours<br><br>Comparison group: participants met once per week for a 1 hour of educational session during the 5-week period<br><br>The session was led by an occupational therapist, group discussions were about communication difficulties, fatigue, depressive symptoms, mood swings, personality changes and dysphagia, all more or less hidden dysfunctions after stroke and how to cope with these difficulties<br><br>There was no special focus on the risks of falling in these discussions
Outcomes	Outcomes were recorded at baseline, 5 weeks (post treatment), 3 months and 6 months

	Balance/sit-to-stand outcome measures: Berg Balance Scale, Falls Efficacy Scale, number of falls<br><br>ADL outcome measures: Barthel Index, Frenchay Activities Index
Notes	No significant differences between groups at baseline<br><br>11 participants in total fell during study (32%), RTT (n = 5), control (n = 6)

Risk of Bias[41]

**Bias**	**Author's Judgment**	**Support for Judgement**
Random sequence generation	Low risk	Minimisation software program
Allocation concealment	Low risk	The randomisation procedure was conducted by the 2 principal investigators who were involved neither in the assessments, nor in the RTT or control group<br><br>Both investigators were blinded to allocation at the time of randomisation, which was made possible by using code numbers for each participant
Blinding of participants and personnel		
Blinding of outcome assessment	Low risk	All assessments were done by blinded staff, who were instructed that if they had any reason to believe that they had revealed a participant's group they should make an adverse event report. The staff in the intervention did not take part in any of the assessments
Incomplete outcome data	Low risk	Small number of dropouts, reasons provided
Selective reporting	Unclear risk	No study protocol
Other bias		

Hornnes (2011)

Characteristics[10]

Methods	RCT<br><br>Unit of randomisation: participant
Participants	Place of recruitment: hospital

	Numbers randomised: total: 349 (I: 172; C: 177)<br><br>% Completing final follow-up: 87%<br><br>Inclusion criteria: ischaemic stroke, intracerebral haemorrhage or TIA<br><br>Exclusion criteria: discharged to a nursing home; cognitive deficits prohibiting informed consent; life expectancy < 2 years<br><br>Type of stroke (%): ischaemic (I: 71%; C: 73%); intracerebral haemorrhage (I: 3%; C: 5%); TIA: (I: 26%; C: 22%)<br><br>Mean age (SD): I: 70.2 (13.7); C: 68.5 (12.2)<br><br>Gender (% women): I: 48%; C: 50%<br><br>Ethnicity: not reported<br><br>Socio-economic or socio-demographic status:<br><br>• Living alone (%): I: 52%; C: 52%<br>• Educational level (%): I: low – 31%, medium – 26%, high – 43%; C: low – 32%, medium – 26%, high – 42%
Interventions	Intervention details (components, length, frequency): 4 home visits from a nurse at 1, 4, 7 and 10 months; each visit included blood pressure monitoring, tailored lifestyle counselling and promotion of medication compliance; hypertensive participants encouraged to visit their GP<br><br>Location: community<br><br>Mode of delivery: home visits<br><br>Personnel responsible for delivery: nurse<br><br>Timing post-stroke: randomised at time of discharge<br><br>Control: usual care (neurologist outpatient visit 3 months post-stroke)
Outcomes	12 months: SBP; DBP; proportion of participants meeting BP targets; proportion of participants adhering antihypertensive therapy
Notes	Analysis method: not reported<br><br>Risk of bias: low

Risk of Bias[10]

Bias	Author’s Judgment	Support for Judgement
Random sequence generation	Low risk	"Used a computer-generated, block randomization procedure"
Allocation concealment	Low risk	"The allocation sequence was concealed … the study nurses who administered the

		intervention had access to a computer program … entering the patient's Central Person Registry number, BP value, and hospital yielded a printout of the patient's randomization number and allocation"
Blinding of participants and personnel		
Blinding of outcome assessment		
Incomplete outcome data	Low risk	Missing data reported by group<br><br>Attrition: I: 27/172 (13 dropped out; 3 diagnosis revised; 10 died; 1 too ill); C: 19/177 (9 dropped out; 5 died; 2 too ill; 2 diagnosis revised; 1 other reason)<br><br>Judgement: reasons for missing data reported and review authors judge that they are unlikely to be related to study outcomes
Selective reporting	Low risk	Outcomes pre-specified (trial registry: www.clinicaltrials.gov/ct2/show/NCT00253097)
Other bias	Low risk	The study appears to be free of other sources of bias

Hougaard (2014)

Characteristics[29]	
Methods	Study design: RCT<br><br>Randomisation: drawing from a large number of sealed opaque envelopes<br><br>Allocation concealment: sealed opaque envelopes<br><br>Study grouping: parallel group<br><br>Masking: single-blinded (outcome assessor)<br><br>Analysis method: PP analysis<br><br>Total duration of study: 19 months, from June 2009 to January 2011
Participants	285 participants (intervention = 160; control = 125)<br><br>Denmark

	Single centre: Aarhus University Hospital<br><br>Setting: emergency<br><br>Sex: both (57% men, 59% women)<br><br>Mean age: 66.9 years old<br><br>Inclusion criteria: eligible patients were ≥ 18 years of age with symptoms of acute stroke according to the prehospital services assessment, as per standardised national guidelines (modified Face, Arm, Speech Test (FAST) criteria).<br><br>Exclusion criteria: contraindications to rtPA or MRI, known hypersensitivity to Gadovist or any of its ingredients, acute or chronic severe renal impairment, and people with a non-stroke diagnosis
Interventions	Participants were randomly assigned to either of 2 groups:<br><br>Group 1: RIC group (n = 81), intravenous thrombolysis plus RIC. RIC was induced by 4 inflations of a standard upper limb blood pressure cuff to either 200 mmHg or 25 mmHg above the patient's systolic blood pressure, each lasting 5 minutes and separated by 5 minutes of cuff deflation<br><br>Group 2: control group (n = 68), intravenous thrombolysis alone<br><br>Concomitant medications: standard care according to national guidelines for the treatment of acute ischaemic stroke by intravenous thrombolysis
Outcomes	Primary outcome: the penumbra salvage<br><br>Secondary outcomes: final infarct size, infarct growth, and clinical outcome at 3 months<br><br>Other outcomes: risk of infarction in treated and untreated tissue according to the severity of the level of hypoperfusion at admission
Notes	External blinded review of outcomes<br><br>Follow-up: 52.3%<br><br>Demographic: inadequately reported<br><br>Principle investigator: Kristina Dupont Hougaard, MD<br><br>Funding: the study was funded by grants from the Danish National Research Foundation, the Aase and Ejnar Danielsen Foundation, and the Tryg Foundation. Bayer AG supplied the contrast media for perfusion scans.<br><br>Notable disclosures: 2 authors are shareholders in CellAegis Devices Inc, who made the RIC device in the study.

Risk of Bias[29]

**Bias**	**Author's Judgment**	**Support for Judgement**

Random sequence generation	Low risk	Randomisation was performed by drawing from a large number of sealed opaque envelopes.
Allocation concealment	Unclear risk	Sealed opaque envelopes were used, but whether envelopes were sequentially numbered is unclear.
Blinding of participants and personnel	High risk	The participants were not blinded to the treatment protocol.
Blinding of outcome assessment	Low risk	All outcomes assessors were blinded to treatment allocation.
Incomplete outcome data	High risk	"As-treated" analysis was done with substantial departure of the intervention received from that assigned at randomisation.
Selective reporting	Low risk	The study protocol was available and all of the study's prespecified outcomes had been reported in the prespecified way.
Other bias	High risk	2 authors were shareholders in CellAegis Devices Inc where the devices used in this study were made.

Immink (2014)

Characteristics[42]	
Methods	Design: RCT<br><br>Study duration: 10 weeks<br><br>Randomisation: a random allocation table was generated using Microsoft Excel (Microsoft Corporation, Redmond, WA) to allocate consenting participants to either of the 2 groups<br><br>Allocation concealment: randomisation, using concealed allocation procedures, was conducted by a research associate who was external to the study<br><br>Blinding: not possible due to the nature of the intervention. Participant assessment was conducted by author 2 who was blinded to participant allocation<br><br>ITT: yes
Participants	Randomised: 25

	Withdrawals: intervention group: n = 1, no reason given; waiting-list control group: n = 2, 1 due to an unrelated medical condition, no reason was provided for the other<br><br>Intervention group: 11 participants; 5 women, 6 men; mean age 56.1 (SD 13.6) years; mean time since stroke: 81.6 (SD 77.5) months<br><br>Waiting-list control group: 11 participants; 8 women, 3 men; mean age 63.2 (SD 17.4) years; mean time since stroke: 23.3 (SD 12.5) months<br><br>Inclusion criteria: ≥ 18 years of age, diagnosis of stroke ≥ 9 months prior to baseline assessment, hemiparesis, completion of post-stroke rehabilitation, ability to follow 2-step commands, able to ambulate independently or with supervision, with or without an assistive device<br><br>Exclusion criteria: other neurological or neuromuscular conditions, current or previous participation in yoga or meditation practice, currently participating in structured exercise programmes
Interventions	Intervention: a standardised 10-week yoga intervention, involving:<br><br>Weekly 90-minute group classes<br><br>• 10 minutes of education component (lecture on concepts in yoga and the focus theme for that week's class)<br>• 30 minutes of yoga asana<br>• 10-12 minutes of pranayama<br>• 20-30 minutes of Satyananda Yoga Nidra<br>• 8-10 minutes discussion<br><br>Daily 40-minute (35-45 minutes) individual home practice<br><br>• 10-20 minutes for yoga asana and pranayama<br>• 25 minutes for Satyananda Yoga Nidra<br><br>Weekly group classes were facilitated by 2 accredited yoga instructors; an illustrated guide book and compact disc containing audio recordings was provided for home practice<br><br>Intervention design: the intervention was specifically developed for a chronic post-stroke population. It appears to be well divided between asanas, breathing/relaxation exercises and discussion. There is no indication of which type of yoga was used to design the course<br><br>Control: participants were advised to maintain their usual treatment and lifestyle behavior where possible during the period of their participation, and to advise the investigators of any change to these<br>conditions.<br><br>Setting: a recreation room at the University of South Australia campus
Outcomes	Included outcomes<br><br>• Motor Function: 9-hole peg test of manual dexterity; Motor Assessment Scale; Berg Balance Scale; 2-minute walk distance; Comfortable Gait Speed

	• Anxiety and Depression: Geriatric Depression Scale-short form; State Trait Anxiety Inventory<br>• Quality of Life: Stroke Impact Scale version 3<br><br>Measurement time points: baseline assessment; upon completion of the intervention
Notes	

Risk of Bias[42]

Bias	Author's Judgment	Support for Judgement
Random sequence generation	Low risk	A random allocation table was generated using Microsoft Excel (Microsoft Corporation, Redmond, WA) to allocate consenting participants to either of the 2 groups
Allocation concealment	Low risk	Randomisation, using concealed allocation procedures, was conducted by a research associate who was external to this study
Blinding of participants and personnel	High risk	Not possible due to nature of the intervention
Blinding of outcome assessment	Low risk	Participant assessment was conducted by Author 2 who was blinded to participant allocation
Incomplete outcome data	Unclear risk	9-Hole Peg Test was not included in the analysis
Selective reporting	High risk	9-Hole Peg Test was not included in the analysis
Other bias	Unclear risk	None identified

Jayasekeran (2010)

Characteristics[23]

Methods	Computerised randomisation by minimisation<br><br>Blinded outcome measures<br><br>Balancing of prognostic baseline factors between treatment groups unclear
Participants	2 centres in UK<br><br>28 patients with acute anterior circulation cerebral infarct or haemorrhage (< 3 weeks)<br><br>Mean age 75 years
Interventions	Rx: bedside pharyngeal electrical stimulation<br>C: sham stimulation

	Duration: once daily for 3 consecutive days
Outcomes	Airway aspiration at 2 weeks' post intervention
Notes	Exclusion: dementia, pacemaker or implantable cardiac defibrillator, severe receptive aphasia, unstable cardiopulmonary status, distorted oropharyngeal anatomy (e.g. pharyngeal pouch), brain-stem stroke, and dysphagia resulting from conditions other than hemispheric stroke

Risk of Bias[23]

**Bias**	**Author's Judgment**	**Support for Judgement**
Random sequence generation	Low risk	Computerised randomisation by minimisation
Allocation concealment	Low risk	As above
Blinding of participants and personnel	Unclear risk	Unclear
Blinding of outcome assessment	Low risk	Blinded outcome measures
Incomplete outcome data	High risk	3 lost to follow-up
Selective reporting	Low risk	
Other bias		

Jüttler (2007)

Characteristics[39]

Methods	Prospective, randomised, controlled, open trial<br>Blocked randomisation, stratified for each centre
Participants	32 patients < 60 years of age<br><br>Patients included had an infarct involving > 2/3 of the MCA territory with score > 1 in item 1a of NIHSS<br><br>15 received medical therapy and 17 received medical therapy and surgical decompression<br><br>Patients were excluded if they had a mRS ≥ 2, a BI < 95, a GCS < 6, dilated pupils, any other brain lesion that might affect outcome, haemorrhagic transformation, life expectancy < 3 years, coagulopathy or any other serious illness that might affect outcome
Interventions	Medical treatment alone versus medical treatment and surgical decompression<br><br>Medical treatment included the use of osmotic agents on schedule to a target osmolality 315 to 320 mOsm, mechanical ventilation, blood pressure control, temperature and glucose control maintaining euvolemia<br><br>Surgical decompression included a bone flap > 12 cm in diameter including frontal, temporal parietal and occipital bones and durotomy<br><br>Surgery had to be performed > 12 hours but < 30 hours from symptom onset and no more than 6 hours from randomisation

Outcomes	Primary effect measure: favourable functional outcome defined as mRS ≤ 3 at 6 months and 12 months<br><br>Secondary effect measures: mRS ≤ 4 and BI at 6 and 12 months<br><br>Mortality at 30 days was also assessed but not as primary outcome
Notes	The study was stopped prematurely given the results of the pooled analysis of the three European trials

Risk of Bias[39]

Bias	Author's Judgment	Support for Judgement
Random sequence generation	Low risk	
Allocation concealment	High risk	
Blinding of participants and personnel	High risk	
Blinding of outcome assessment	High risk	
Incomplete outcome data		
Selective reporting		
Other bias		

Kennedy (2007)

Characteristics[43]

Methods	Parallel group, placebo-controlled trial<br>2 x 2 factorial design with clopidogrel
Participants	392 patients<br>Country: Canada<br>Study years: 2003 to 2006<br>Age: 40 years or older<br>Male: 53%<br>Inclusion: TIA or minor stroke (NIHSS < 4) within 24 hours of onset
Interventions	Simvastatin (40 mg/d)<br>Matching placebo
Outcomes	Stroke within 90 days<br>Major vascular event
Notes	Trial stopped early because of low recruitment

Risk of Bias[43]

Bias	Author's Judgment	Support for Judgement
Random sequence generation		
Allocation concealment	Low risk	A - Adequate
Blinding of participants and personnel		
Blinding of outcome assessment		

Incomplete outcome data
Selective reporting
Other bias

Kernan (2016)

Characteristics[27]	
Methods	A multicentre, double-blind, placebo-controlled clinical trial to test the effectiveness of pioglitazone for insulin resistant, non-diabetic patients with a recent ischaemic stroke or TIA
Participants	People aged at least 40 years with qualifying ischaemic stroke or TIA during the 6 months before randomisation, as well as insulin resistance, defined as a value of more than 3.0 on the homeostasis model assessment of insulin resistance index. 3876 participants were randomised.
Interventions	Pioglitazone or matching placebo. The initial dose was 15 mg of pioglitazone daily or placebo. The dose was increased to 2 pills daily (30 mg of pioglitazone or placebo) at 4 weeks and to 3 pills daily (45 mg of pioglitazone or placebo) at 8 weeks. At 12 weeks, participants were started on 1 x 45 mg pioglitazone tablet or placebo tablet daily.
Outcomes	Fatal or non-fatal stroke; fatal or non-fatal myocardial infarction; heart failure resulting in hospitalisation or death; death from any cause; diabetes; and cognitive decline
Notes	Participants were contacted every 4 months with a median follow-up of 4.8 years.

Risk of Bias[27]

**Bias**	**Author's Judgment**	**Support for Judgement**
Random sequence generation	Low risk	Randomisation was performed using a random permuted block design with variable block sizes stratified by site.
Allocation concealment	Low risk	To conceal the allocation sequence, randomisation lists were kept only at the central pharmacy and the statistical centre.
Blinding of participants and personnel	Low risk	The Investigational Drug Service prepared medication bottles, including starter supplies, which were stored at the research sites. At the baseline visit, a structured interview was administered and the starter bottle with the participant's assigned randomisation number was dispensed.
Blinding of outcome assessment	Low risk	Reviewers were blinded to treatment allocation.

Incomplete outcome data	Low risk	During a median follow-up of 4.8 years, a total of 227 participants (5.9%) withdrew consent and 99 participants (2.6%) were lost to follow-up.
Selective reporting	Low risk	All prespecified outcomes were reported.
Other bias	Unclear risk	This study was supported by the National Institute of Neurological Disorders and Stroke (NINDS) and monitored by an independent data and safety monitoring board appointed by NINDS. Pioglitazone and placebo were provided by Takeda Pharmaceuticals International.

Kerry (2013)

Characteristics[10]	
Methods	RCT<br><br>Unit of randomisation: participant
Participants	Place of recruitment: outpatient and inpatient stroke clinics<br><br>Numbers randomised: total: 381 (I: 187; C: 194)<br><br>% Completing final follow-up: 88%<br><br>Inclusion criteria: ≤ 9 months since stroke or TIA and hypertension (BP > 140/85 mmHg or treatment with antihypertensive medications)<br><br>Exclusion criteria: enrolled in another trial; severely ill or too frail; already using a blood pressure monitor; severe cognitive impairment; non-English speaking<br><br>Type of stroke (%): ischaemic (I: 58%; C: 64%); haemorrhagic (I: 7%; C: 5%); TIA (I: 34%; C: 30%); both types of stroke or unknown (I: 1%; C: 2%)<br><br>Mean age (SD): I: 71.1 (12.6); C: 72.6 (11.4)<br><br>Gender (% men): I: 59%; C: 56%<br><br>Ethnicity: White (I: 80%; C: 73%); Black (I: 11%; C: 15%); Asian (I: 4%; C: 7%); other (I: 5%; C: 5%)<br><br>Socio-economic or socio-demographic status:<br><br>• Index of Multiple Deprivation score* (mean ± SD): I: 17.5 ± 10.7; C: 19.3 ± 10.1
Interventions	Intervention details (components, length, frequency): participants provided with a home blood pressure monitor, brief training and ongoing nurse-led telephone

	support targeting BP reduction (average of 3.8 telephone calls over 12 months); participants with consistent blood pressure readings ≥ 130/80 mmHg advised to consult their GP and received intensified nurse-led telephone follow-up until the target was reached (i.e. implementation of protocols for BP reduction)<br><br>Location: community<br><br>Mode of delivery: home visits and telephone follow-up<br><br>Personnel responsible for delivery: nurse<br><br>Timing post-stroke: ≤ 9 months<br><br>Control: baseline assessment conducted during home visit and all participants with BP > 150/90 mmHg were advised to see their GP; usual care provided by GP (all GPs sent information about the study and a recommended target for home blood pressure of < 130/80 mmHg); participants in the control group received telephone calls after 3 and 9 months to check on their well-being
Outcomes	12 months: SBP; DBP, proportion of participants with recurrent stroke
Notes	Analysis method: available case analysis<br><br>Risk of bias: low<br><br>*Trialists state that "the Index of Multiple Deprivation 2007 scale is a measure of poverty and is based on postal codes and ranges from 0.37 to 85.46. A higher score indicates higher deprivation. Further information can be found at www.communities.gov.uk/communities/research/indicesdeprivation/deprivation10/"

Risk of Bias[10]

Bias	Author's Judgment	Support for Judgement
Random sequence generation	Low risk	"A computer-generated randomization sequence to implement stratified randomization … with a 1:1 allocation using random block sizes of 4 and 6"
Allocation concealment	Low risk	"Allocation to the intervention or control group was contained within a sealed, numbered envelope and assigned to the participant by the trial administrator before the baseline visit. The research nurse opened the envelope after she completed the home baseline assessment."
Blinding of participants and personnel		
Blinding of outcome assessment		
Incomplete outcome data	Low risk	Missing data reported by group

		Attrition: I: 18/187 (9 died, 5 lost contact, 1 moved away, 3 declined); C: 25/194<br>(10 died, 6 lost contact, 5 withdrew because of illness, 2 moved away, 2 declined)<br><br>Excluded from analysis: I: 1/187 (reason not provided); C: 0<br><br>Judgement: reasons for missing data reported and review authors judged that they were unlikely to be related to study outcomes
Selective reporting	Low risk	Protocol is available and outcomes are reported in the pre-specified way
Other bias	Low risk	The study appears to be free of other sources of bias

Khedr (2013)

Characteristics[44]	
Methods	Study design: randomised controlled trial (parallel assignment)<br><br>Dropouts: none<br><br>Adverse effects: none<br><br>Deaths: none<br><br>ITT: yes, all participants completed the study<br><br>Country: Egypt
Participants	Number of participants: 40 outpatients<br><br>Age: (mean ± SD) years<br><br>Gender: 14 females (35%)<br><br>Type of stroke: acute single thromboembolic non-haemorrhagic infarction, documented by MRI<br><br>Time poststroke: (mean ± SD) 17.1 ± 3.6 days<br><br>Severity: (range) 7 to 13 on NIHSS<br><br>Exclusion criteria: extensive infarction (all territories of MCA), severe flaccid hemiplegia, head injury, neurological disease other than stroke, renal or hepatic impairment, previous administration of tranquillizer, inability to give informed consent, no MEP recorded from FDI muscle of the affected hand
Interventions	3 arms:

	1. A-tDCS, 25 minutes at 2 mA daily for 6 consecutive days on M1 of the lesioned hemisphere, delivered by saline-soaked pads (5 × 7 cm)<br><br>2. C-tDCS, 25 minutes at 2 mA daily for 6 consecutive days on M1 of the non-lesioned hemisphere, delivered by saline-soaked pads (5 × 7 cm)<br><br>3. S-tDCS, 25 minutes daily (with a short ramp-up and ramp-down of the current at the beginning and at the end of each session) for 6 consecutive days on M1 of the lesioned hemisphere
Outcomes	NIHSS at baseline, at the end of the intervention phase and at 1, 2 and 3-month follow-up (0 to 42, with higher scores indicating a more severe stroke)<br><br>OMCASS at baseline, at the end of the intervention phase and at 1, 2 and 3-month follow-up (0 to 100, with higher scores indicating no clinical impairment due to stroke)<br><br>BI at baseline, at the end of the intervention phase and at 1, 2 and 3-month follow-up (0 to 100, with higher scores indicating better global function)<br><br>Muscle strength according to MRC at the end of the intervention phase, at 1, 2 and 3-month follow-up (0 to 5, with higher scores indicating higher muscle strength)<br><br>Cortical excitability (as measured by RMT and AMT) at the end of the intervention phase, at 1, 2 and 3-month follow-up (with greater intensity indicating a higher threshold)
Notes	

Risk of Bias[44]

Bias	Author's Judgment	Support for Judgement
Random sequence generation	Low risk	Quote: "Each patient was given a serial number from a computer-generated randomisation table [...]"
Allocation concealment	Low risk	Quote: "Group allocations (Anodal, Cathodal, or Sham) were placed in serially numbered, opaque closed envelopes [...] and [each patient] was placed in the appropriate group after opening the corresponding sealed envelope"
Blinding of participants and personnel	Low risk	Participants were blinded
Blinding of outcome assessment	Low risk	Outcome assessor was blinded
Incomplete outcome data	Low risk	No loss to follow-up
Selective reporting	Low risk	All outcomes stated in the study protocol and listed in the methods section of the publication have been reported
Other bias		

Kronish (2014)

Characteristics[10]	
Methods	RCT<br><br>Unit of randomisation: participant
Participants	Place of recruitment: senior centres, churches, health fairs, from hospital registries of an academic centre, a federally funded health centre, a home care nursing program, community organisations, through advertising in clinics newspaper adverts<br><br>Numbers randomised: total: 600 (I: 301; C: 299)<br><br>% Completing final follow-up: I: 80% C: 89%<br><br>Inclusion criteria: stroke or mini stroke within the past 5 years, ≥ 40 years<br><br>Exclusion criteria: lacked capacity to consent, lacked physical or mental capacity to participate meaningfully in workshops, non-English/non-Spanish speaking, institutionalised resident<br><br>Type of stroke (%): ischaemic (100%)<br><br>Mean age (SD): I: 63 (11); C: 64 (11)<br><br>Gender (% women): I: 60%; C: 59%<br><br>Ethnicity: Black (I: 40% C: 43%), Latino (I: 42% C: 37%), White (I: 13% C: 14%), other (I: 4% C: 6%).<br><br>Socio-economic or socio-demographic status<br>• Annual income ≤ 15,000 dollars/year (%) (I: 56 C: 58)<br>• Less than high school education (%) (I: 31 C: 30)
Interventions	Intervention details (components, length, frequency): Weekly peer-led workshops models on chronic disease self-management program. Also received culturally sensitive educational material at randomisation and encouraged to discuss results with a health care provider<br><br>Location: community<br><br>Mode of delivery: peer-based education<br><br>Personnel responsible for delivery: peers<br><br>Timing post-stroke: up to 5 years post event<br><br>Control: usual care plus the same educational materials at randomisation, a list of local health providers and advice to seek GP. Informed would become involved in intervention after waiting for 1 year
Outcomes	6 months: BP (< 140/90 mmHg) LDL cholesterol < 100mg/dl and antithrombotic use

Notes	Analysis method: intention-to-treat<br><br>Risk of bias: unclear risk

Risk of Bias[10]

Bias	Author's Judgment	Support for Judgement
Random sequence generation	Low risk	Randomisation generated by a computerised random number sequence in blocks of 2, 4, or 6
Allocation concealment	Low risk	Central allocation
Blinding of participants and personnel		
Blinding of outcome assessment		
Incomplete outcome data	Low risk	Missing values were imputed using multiple imputations under the assumption that values were missing at random
Selective reporting	Unclear risk	Insufficient information to permit judgement
Other bias	Low risk	The study appears to be free of other sources of bias

Langhorne (2010)

Characteristics[38]

Methods	A pilot randomized trial (using a 2 x 2 factorial design) to compare conventional stroke unit procedures with more progressive (nurse-led) protocols of early mobilisation (EM), automated physiological monitoring, or both<br><br>As there was no statistical interaction between the 2 protocols, the EM arms were compared directly with the normal mobilisation arms
Participants	1 stroke unit in Glasgow, Scotland<br><br>Recruited 32 acute stroke patients admitted to hospital within 24 hours (and recruited within 36 hours of symptom onset) with no premorbid severe disability<br><br>Of the 16 EM patients, 8 were allocated to the EM protocol alone and 8 to the EM plus automated monitoring protocols<br><br>Of the 16 controls, 8 were allocated to normal mobilisation alone and 8 to normal mobilisation plus the automated monitoring protocol<br><br>Mean age: 67.5 years<br>16/32 (50%) men

	1/32 (3%) haemorrhagic<br>Mean NIHSS 5<br><br>23 (72%) had mild stroke (NIHSS 1 to 7)<br><br>7 (22%) moderate stroke (NIHSS 8 to 16)<br><br>2 (6%) severe stroke (NIHSS > 16)
Interventions	**What**<br><br>Materials: protocols for early mobilisation (VEM) or intensive physiological monitoring, or both, or standard care alone (control). VEM was planned to be similar to AVERT protocol but did not have access to the same written protocol.<br><br>Procedures: the VEM protocol aimed to get participants up to sit, stand, and walk within 24 hours of the stroke and continue this at least 4 times per day<br><br>Controls: the stroke unit had a philosophy of getting patients up to sit, stand, and walk early but did not have staff specifically allocated to this role. Mobilisation was normally provided by physiotherapists and nurses (30 to 60 minutes per day). Normal monitoring involved intermittent (4-hourly) checking of pulse, temperature, oxygen saturation, and blood pressure<br><br>**Who provided**<br><br>The research nurse had a role ensuring the VEM protocol was implemented in conjunction with physiotherapy and nursing staff<br><br>**How**<br><br>The research nurse facilitated the VEM protocol that was largely delivered face-to-face by physiotherapy and nursing staff<br><br>**Where**<br><br>Provided in the stroke unit<br><br>**When and how much**<br><br>For 1 week after recruitment or until discharge<br><br>**Tailoring**<br>EM could be adapted according to the abilities of the participant<br><br>**Modifications**<br><br>Mobilisation could be halted if monitoring of pulse, temperature, oxygen saturation, or blood pressure suggested abnormal changes<br><br>**How well**<br><br>Planned: the EM protocol aimed to get participants up to sit, stand, and walk within 24 hours of the stroke and continue this at least 4 times per day. This

	was monitored as: 1) time-to-first mobilisation (attempt to get the participant out of bed, to sit, stand, or walk); 2) best level of mobilisation activity achieved (lying, sitting, standing, walking); and 3) participant activity (using automated activity monitor recordings)<br><br>Actual: median time from stroke to first mobilisation in the VEM group was 27.3 hours (IQR 26.0 to 29.0) vs 32.0 hours (IQR 22.5 to 47.3) in controls (P = 0.31), however a significantly greater number of EM participants (P = 0.03) were mobilised within 1 hour of randomization. A significantly great number of EM participants (P = 0.02) achieved standing or walking when mobilisation was recorded using activity monitors
Outcomes	mRS at 3 months, adverse events, patient activity, neurological deterioration, Barthel Index at 1 week and 3 months, walking speed (1 week and discharge), patient satisfaction, resource allocation
Notes	Recruitment took place in February 2007 to January 2008

Risk of Bias[38]

Bias	Author's Judgment	Support for Judgement
Random sequence generation	Low risk	The randomization sequence was to 1 of 4 nurse-led treatment protocols (2 of which delivered EM). The sequence was computer-generated in blocks of 4
Allocation concealment	Low risk	Quote: "patients were then randomly allocated by telephoning a secretary in an independent office who logged the patient and opened the next in a series of sequentially numbered opaque sealed envelopes"
Blinding of participants and personnel	Unclear risk	Participants were aware that they were being randomized to different care protocols. No specific measures were in place to segregate participants but recruitment did not result in participants receiving different protocols in the same ward space
Blinding of outcome assessment	Low risk	Key outcome observations were blinded to treatment allocation by using an independent assessor at day 5 and at 3 months
Incomplete outcome data	Low risk	No losses after randomization
Selective reporting	Low risk	Primary outcome complete
Other bias		

Lennon (2010)

Characteristics[45]	
Methods	RCT<br>Sample = 48<br>CR group: 24 participants<br>Control group: 24 participants<br>Allocation concealment: opaque envelope<br>Blinding: single blind<br>Withdrawal/dropout: 1<br>Follow-up: 1<br>Treatment duration: 10 weeks (twice weekly)
Participants	Age (mean ± SD): intervention = 59.0 ± 10.3 years; control = 60.5 ± 10.0 years<br><br>Males: intervention = 14; control = 14<br>Females: intervention = 10; control = 10<br><br>Inclusion criteria: > 1-year post ischemic stroke (confirmed by CT or MRI scan)<br><br>Over 18 years of age
Interventions	All participants who were receiving physiotherapy or occupational therapy at the time of the study continued with these therapies<br><br>Intervention: 10-week CR program consisting of 30-minute cycle ergometry exercise using either upper or lower limbs twice weekly. Additionally, participants attended 2 life-skills classes addressing stress management, relaxation and life balance<br><br>Control: no additional intervention
Outcomes	Cardiac risk score; VO2 (mL O2 /kg per minute) and RPE were assessed during a standardized ergometry test; HADS; Frenchay Activity Index; fasting lipid profiles and resting blood pressure
Notes	

Risk of Bias[45]

**Bias**	**Author's Judgment**	**Support for Judgement**
Random sequence generation	Low risk	Participants were "randomized assigned using a sequence generator (SPSS version 12.0), to either intervention or control groups, by an independent party" (127/1/1)
Allocation concealment	Low risk	"Participants were handed an opaque envelope which contained details of their group assignment, by clerical staff unrelated to the trial" (127/1/2)

Blinding of participants and personnel	Low risk	Participants were randomly allocated to either intervention or control group by an independent party
Blinding of outcome assessment	Low risk	"Measurements were made by an independent assessor blinded to allocation" (126/4/2)
Incomplete outcome data	Low risk	Of the 66 interested participants, 18 were withdrawn at initial screening (5 on holidays during trial, 12 on beta-blockers and 1 had unstable diabetes). Of the 48 participants enrolled in the trial (24 in control group, 24 in intervention group), 1 participant in the intervention group was withdrawn prior to intervention and 1 participant in the control group was lost to follow-up making a total of 23 people analyzed in each group. Data were presented for all participants assigned to intervention and control groups
Selective reporting	Low risk	All outcomes pre-specified in the methods section were reported in the results section. Data for primary outcomes were presented in Table 2
Other bias	Unclear risk	The influence of unequal volumes of intervention between the experimental and control groups on outcomes could not be ascertained

Levin (2012)

Characteristics[46]	
Methods	RCT |
Participants	Recruited from an outpatient rehabilitation centre in Israel<br><br>12 participants: 6 intervention, 6 control<br><br>Inclusion criteria: unilateral left- or right-sided stroke > 3 months previously. No hemispatial neglect or uncorrected visual field deficits including

	hemianopia and could understand and follow instructions (no receptive aphasia, MMSE evaluation)<br><br>Exclusion criteria: shoulder or arm pain, lack of endurance as judged by their treating physician<br><br>Mean (SD) age: intervention group 58.1 (14.6) years, control group 59.8 (15.1) years<br><br>50% men<br><br>Stroke details: 58% right hemiplegia<br><br>Timing post stroke: intervention group mean 2.6 (1.2) years, control group mean 3.8 (0.9) years
Interventions	VR intervention: goal-directed reaching tasks using the affected arm in a virtual environment (virtual supermarket, birds and balls, soccer, volleyball, VMall). Practice involved reaching but not grasp or manipulation. Task difficulty was matched to capabilities<br><br>Control intervention: OT including exercises reaching for and holding cones, cups and other objects with and without external loading<br><br>Sessions were 45 min for 9 sessions over a 3-week period
Outcomes	Assessed post intervention and 4 weeks after the end of intervention<br><br>Fugl Meyer Arm Scale<br><br>Composite Spasticity Index<br><br>Reach Performance Scale for Stroke<br><br>Upper limb activity: box and blocks test<br><br>Upper limb activity: Wolf Motor Function Test<br><br>Motor Activity Log<br><br>Adverse events
Notes	NCT01388400

Risk of Bias[46]

Bias	Author's Judgment	Support for Judgement
Random sequence generation	High risk	Coin toss
Allocation concealment	Low risk	As above – coin toss
Blinding of participants and personnel		
Blinding of outcome assessment	Low risk	Blinded assessor

Incomplete outcome data	Low risk	Small number of withdrawals
Selective reporting	Low risk	Reported on clinical trial registry
Other bias		

Levy (2009)

Characteristics[47]	
Methods	C: identical-appearing vials were assigned by using an interactive voice response system<br><br>Patients and assessors blinded<br><br>Ex during trial: none<br><br>Losses to FU: 0 in Rx, 3 in control at 90 days
Participants	500 patients<br><br>Age: mean 70.2 years in Rx group, mean 69.6 years in placebo group<br><br>Sex: 49.2% male<br><br>Ischaemic stroke with pretreatment NIHSS ≧ 5 (pretreatment NIHSS 1a > 2 were excluded)<br><br>100% CT or MRI before entry<br><br>< 6 hours from stroke onset<br><br>Comparability: similar
Interventions	Rx: 0.5 IU/kg ancrod for 3 hours in patients with pretreatment fibrinogen levels ≧ 200 mg/dL or 0.33 IU/kg ancrod for 2 hours in patients with pretreatment fibrinogen levels 100 to 199 mg/dL<br><br>Control: placebo<br><br>Duration: 2 or 3 hours
Outcomes	Death at 90 days<br><br>Disability at 90 days (mRS)<br><br>Symptomatic intracerebral haemorrhage<br><br>Asymptomatic intracerebral haemorrhage
Notes	FU: 90 days for death and disability

	Ex: neuroimaging evidence of haemorrhage; coma; NIHSS < 5; use or intended use of thrombolytic agent; previous stroke; blood pressure > 185/105 mmHg; fibrinogen < 100 mg/dL; intrinsic or extrinsic coagulation disorders; patients with medical condition not likely to survival through 90 days; previous exposure to ancrod or snake bites

Risk of Bias[47]

Bias	Author's Judgment	Support for Judgement
Random sequence generation	Low risk	Computer-generated codes
Allocation concealment	Low risk	Identical-appearing vials
Blinding of participants and personnel	Low risk	Patients and assessors blinded
Blinding of outcome assessment	Low risk	Patients and assessors blinded
Incomplete outcome data	Low risk	ITT analysis
Selective reporting	Low risk	No obvious selective reporting was found
Other bias	High risk	Ended early for futility reasons

Lowe (2006)

Characteristics[48]

Methods	Randomised using sealed opaque envelopes in blocks of 10 and 1 to 1 ratio.<br>Envelopes prepared by independent researcher<br>Blinded outcome assessment<br>Stated intention-to-treat analysis<br>16 patients (6 treatment, 10 control) lost to follow-up<br>3 and 6-month follow-up
Participants	Liverpool, UK<br><br>100 stroke patients: treatment N = 50; control N = 50. Completed final follow-up: N = 84<br><br>Median age of patient: treatment 68 years; control 73 years<br>Sex of patient female: treatment 42%; control 38%<br><br>Inclusion criteria: Confirmed stroke, all ages, either sex, patients who are discharged home and who can complete a questionnaire, or who have a named carer who can do so<br><br>Exclusion criteria: pre-existing cognitive impairment, discharge to institutionalised care, discharge home but unable to self-complete questionnaire and no named carer
Interventions	Treatment: CareFile (A5 size laminated 29 page booklet). Includes general information about stroke as well as information personal to the patient, secondary prevention measures, and personal goals aimed at reducing risk of further stroke. Also contains useful telephone numbers for all stroke-

	related services and local support agencies. Design allows for removal of pages not relevant to the individual. Sections included for members of the multi-disciplinary team to complete summaries of patient's achievements and future rehabilitation goals. Also provided with advice from therapists and offered leaflets from Chest, Heart and Stroke Association<br><br>Focus: patient<br><br>Setting: hospital ward<br><br>Administration: interview arranged between researcher and patient when patient discharge date in place. Carer also invited to attend. The CareFile and its contents explained by the research registrar and any additional concerns or issues addressed in discussion lasting approximately 15 to 20 minutes. Patients advised to take the CareFile with them to all General Practitioner and clinic appointments<br><br>Control: received the usual stroke information leaflets provided by the stroke unit and follow-up in stroke review clinic
Outcomes	Primary<br>(1) Knowledge of stroke (3 and 6 months)<br><br>Secondary<br>(1) Utilisation of CareFile (3 and 6 months)<br>(2) Satisfaction with information given (3 and 6 months)<br>(3) Blood pressure (3 and 6 months)<br>(4) Participation (3 and 6 months)<br>(5) Screening question for depression (3 and 6 months)
Notes	

Risk of Bias[48]

Bias	Author's Judgment	Support for Judgement
Random sequence generation	Unclear risk	Reported that eligible patients were randomised but method not reported
Allocation concealment	Low risk	Reported to have used sealed opaque envelopes
Blinding of participants and personnel	High risk	No report of blinding of participants or personnel
Blinding of outcome assessment	High risk	Outcome assessors do not appear to have been blinded - "those in the intervention group were asked if they had brought the CareFile to the Review Clinic and if they found it useful"
Incomplete outcome data	High risk	Almost twice as many lost to follow-up in the control group (10/50) compared with the intervention group (6/50)

Selective reporting	Unclear risk	Study protocol not available so cannot assess reporting bias
Other bias	Low risk	No other obvious sources of bias

Lund (2011)

Characteristics[37]	
Methods	RCT
Participants	Adults with stroke, living in community
Interventions	Intervention: lifestyle course and PA: n = 48<br><br>* intended audience: stroke survivors<br>* theoretical rationale: need for long-term intervention and support, lifestyle-oriented, occupation-based rationale<br>* mode: group, senior centres<br>* personnel: trained stroke health professional (OT) for lifestyle sessions and volunteers for PA<br>* delivery method: face-to-face<br>* language: not stated<br>* content/topics covered: COPM interviews (goal setting) then topics on lifestyle, choices, healthy living, habit change, oral and written evaluations ongoing<br>* duration: over 9/12, weekly session (2 hours each - total 36 sessions)<br><br>Control: PA only: n = 51<br><br>Completed over 9/12, 1 x 30- to 60-minute group session per week (36 sessions) - non-specific physical activity<br><br>Open to all seniors regardless of diagnosis
Outcomes	Primary: SF-36<br><br>Secondary: COPM, HADS, Timed Up and Go; Trail making A and B<br><br>Assessed at baseline and 9/12
Notes	

Risk of Bias[37]

**Bias**	**Author's Judgment**	**Support for Judgement**
Random sequence generation	Low risk	Computerised randomisation list in blocks of 10, stratified to centres
Allocation concealment	Low risk	Sealed envelopes opened by researcher
Blinding of participants and personnel	High risk	Neither able to be blinded to group

Blinding of outcome assessment	Low risk	Assessor blinded
Incomplete outcome data	High risk	Imbalance in lost to follow-up
Selective reporting	Unclear risk	Protocol not available
Other bias	Low risk	n/a

MacMahon (2001)

Characteristics[49]

Methods	RCT
Participants	Total number of participants randomised: 6105<br><br>Setting: hospital<br><br>Participants: patients with TIA or stroke in the previous 5 years<br><br>Mean age: 64 years<br><br>Sex: 70% men<br><br>Country: Australia, Belgium, China, France, Japan, Italy, New Zealand, Sweden and UK
Interventions	Perindopril (4 mg once a day) ± indapamide (2.5 mg once a day, Japan: 2 mg once a day)
Outcomes	Recurrent stroke of any type<br><br>Time to recurrent stroke<br><br>Major vascular event<br><br>Ischaemic stroke<br>Haemorrhagic stroke<br><br>Myocardial infarction (composite of non-fatal myocardial infarction and fatal coronary)<br><br>Vascular death<br><br>Death by any cause<br><br>Dementia
Notes	Index event was prior history of stroke or TIA

Risk of Bias[49]

Bias	Author's Judgment	Support for Judgement

Random sequence generation	Low risk	Quote: "A minimization algorithm stratifies treatment allocation..."
Allocation concealment	Low risk	Quote: "Allocation to active treatment or to placebo was performed by fax from the collaborating clinical centres to the study randomization centre in Auckland."
Blinding of participants and personnel	Low risk	Quote: "Patients assigned placebo received placebo tablets identical in appearance to perindopril."
Blinding of outcome assessment	Low risk	Quote: "An endpoint adjudication committee reviewed source documentation for all individuals (...)"<br><br>Comment: probably done.
Incomplete outcome data	Low risk	Comment: missing outcome data balanced, and the proportion not enough to have a clinically relevant impact on the intervention effect estimate
Selective reporting	Low risk	Comment: study protocol published; all prespecified outcomes reported
Other bias	Low risk	Comment: the study appears to be free of other sources of bias

Mansfield (2018)

Characteristics[5]

Methods	Multi-site single-blind RCT
Participants	A total of 88 community-dwelling individuals with chronic stroke (> 6 months post stroke)
Interventions	2 training sessions per week, lasting 1 hour per session, for 6 weeks. These sessions include:<br><br>* Experimental group (n = 44): external perturbation training with (apart from 5 to 10 minute warming-up and cooling-down) up to 60 pushes or pulls from the supervising physiotherapist<br><br>* Control group (n = 44): Keep Moving with Stroke programme – an exercise programme to enhance balance and mobility. Quote: "'traditional' balance training program that focuses on maintaining stability during voluntary movement, rather than responding to instability." (Page 5)

	Additionally, participants attended a 1 hour "booster" training 3 and 9 months following the initial training period |
Outcomes	Rate of falls, number of fallers, number of people sustaining fall-related fractures, number of people with fall-related hospital admissions, quality of life, and adverse events
Notes	Fall registration during follow-up period. Registration time: 12 months

Risk of Bias[5]

Bias	Author's Judgment	Support for Judgement
Random sequence generation	Low risk	Quote: "Participants will be assigned using blocked stratified randomization with allocation concealment to one of two training group ... To maintain allocation concealment, a variable block size ranging from 4–8 will be used. There will be four strata based on two stratification factors: site (two levels), and frequency of 'failures' during baseline reactive balance control assessment (two levels)." (Page 4)
Allocation concealment	Low risk	Quote: "Participants will be assigned using blocked stratified randomization with allocation concealment to one of two training groups ..."<br><br>Quote: "To maintain allocation concealment, a variable block size ranging from 4–8 will be used." (Page 4) |
Blinding of participants and personnel		
Blinding of outcome assessment	High risk	Participants recorded falls themselves through the use of a falls calendar
Incomplete outcome data	Low risk	Incomplete outcome data adequately addressed and unlikely to seriously alter the results<br><br>Quote: "Intent-to-treat analysis will be used; all individuals with some falls-monitoring data will be

		included in the analysis." (Page 8)
Selective reporting	Low risk	All of the studies' prespecified outcomes have been reported in the prespecified way
Other bias		

Mayer (2009a)

Characteristics[50]	
Methods	Parallel group, randomised, placebo-controlled, phase III trial
Participants	Inclusion criteria<br><br>* Spontaneous ICH (including bleeding in brainstem and cerebellum) diagnosed by a CT scan within 3 h of symptom onset<br>* Men or women, aged 18 years or over (20 or over in Taiwan)<br>* Informed consent<br><br>Exclusion criteria<br><br>* Time of ICH onset is unknown, or more than 3 h<br>* People with secondary ICH<br>* Surgical haematoma evacuation planned within 24 h of symptom onset<br>* GCS 3 to 5<br>* Known oral anticoagulant use (unless the INR is documented below 1.4)<br>* Known thrombocytopenia (unless current platelets documented above 50,000/mL)<br>* Pre-existing disability (mRS 3 to 5)<br>* Any known history of haemophilia or other coagulopathy<br>* Known acute myocardial ischaemia, unresolved unstable angina, acute septicaemia, acute crush injury, acute disseminated intravascular coagulation, or acute thrombotic stroke<br>* Pregnancy<br>* Known or suspected allergy to trial product or related products<br>* Previous participation in this trial<br>* Known participation in any investigational drug or device trial within 30 days of entry into this trial<br>* People known or suspected of not being able to comply with this trial protocol (e.g. due to alcoholism, drug dependency, or psychological disorder)
Interventions	Intervention:<br><br>Recombinant activated factor VII (NovoSeven) at doses of 20 mcg/kg or 80 mcg/kg, within 1 h of baseline CT and no later than 4 h after ICH onset<br><br>Comparator:<br><br>Placebo

Outcomes	Primary efficacy endpoint was poor outcome, defined as death or severe disability (scores of 5 to 6 on the mRS) at day 90. Analysis claimed to be intention-to-treat, but it did not appear to be.<br><br>Secondary efficacy endpoints: BI, GOS-E, NIHSS, the EuroQol scale, and the Revised Hamilton Rating Scale for Depression at day 90; absolute and percent change in ICH volume as measured by CT from prior to dosing to 24 h after the baseline scan; good outcome (mRS 0 to 1) at day 90; absolute and percent change in total lesion volumes (ICH + IVH + oedema) from baseline to 72 h; BI at day 90; case fatality.<br><br>Safety endpoints: the occurrence of adverse events until hospital discharge, or until day 90, whichever came first, and serious adverse events until the 'End of trial' form was completed
Notes	This trial was funded by Novo Nordisk, which did not respond to repeated requests to provide further data from this trial

Risk of Bias[50]

Bias	Author's Judgment	Support for Judgement
Random sequence generation	Unclear risk	Quote: "Block randomization according to site..."
Allocation concealment	High risk	It became apparent during questioning after the presentation of this trial's data at the European Stroke Conference (Glasgow 2007), that the imbalance in allocation between the 3 groups in this trial (there were approximately 30 more participants analysed in the 80 mcg/kg dose group than the other 2 groups) was due to the fact that the 80 mcg/kg dose of rFVIIa tended to be packed in the first of the 3 boxes of study drug for part of the trial (which might have unblinded investigators, in view of the preponderance of thromboembolic adverse events with the higher dose)
Blinding of participants and personnel	Unclear risk	Classified as 'double-blind', but not described
Blinding of outcome assessment	Unclear risk	Classified as 'double-blind', but not described
Incomplete outcome data	Low risk	Quote: "Outcome scores at day 15 were used according to the principle of the last observation carried forward for 9 patients receiving

		placebo, 9 patients receiving 20 µg of rFVIIa per kilogram, and 13 patients receiving 80 µg of rFVIIa per kilogram (3.7% of patients overall), for whom scores at day 90 were missing. Modified Rankin scale scores were not available for one patient receiving placebo and one patient receiving 20 µg of rFVIIa per kilogram."
Selective reporting	High risk	EuroQuol and Hamilton depression score not reported
Other bias		

Mayer (2009b)

Characteristics[51]	
Methods	Parallel group, randomised (4/block, sequentially numbered, identical-appearing containers), placebo-controlled, phase IIB, dose-ranging, 'proof-of-concept' study.
Participants	Inclusion criteria: age 18 years or older; spontaneous ICH within three hours of onset.<br><br>Exclusion criteria: GCS 3 to 5; surgical ICH evacuation planned within 24 hours; known underlying cause of ICH; on oral anticoagulants; known thrombocytopaenia; coagulopathy, disseminated intravascular coagulation, sepsis, or crush injury; pregnant; mRS > 2 pre-ICH; symptomatic thrombotic or vaso-occlusive disease within 30 days before ICH (mid-way through the trial this was amended to exclude patients with any history of thrombotic or vaso-occlusive disease).
Interventions	Recombinant activated factor VII (NovoSeven) at doses of 40 mcg/kg, 80 mcg/kg, or 160 mcg/kg) versus placebo, within one hour of baseline CT and no later than four hours after ICH onset.
Outcomes	Percentage change in ICH volume on CT from baseline to 24 hours; mRS 4 to 6 or GOS-E 1 to 4 at 90 days; adverse events in hospital and serious adverse events until day 90.
Notes	One important exclusion criterion was changed mid-way through the RCT.<br><br>This trial was funded by Novo Nordisk.

Risk of Bias[51]

Bias	Author's Judgment	Support for Judgement
Random sequence generation		
Allocation concealment	Low risk	A- Adequate

Blinding of participants and personnel	
Blinding of outcome assessment	
Incomplete outcome data	
Selective reporting	
Other bias	

McAlister (2014)

Characteristics[10]	
Methods	RCT<br>Unit of randomisation: participant
Participants	Place of recruitment: outpatient clinic<br><br>Numbers randomised: total: 279 (I: 143; C: 136)<br><br>% Completing final follow-up: 86%<br><br>Inclusion criteria: ischaemic stroke or TIA confirmed by a stroke specialist at one of 3 clinics in Edmonton Canada, > 18 years age, systolic BP or LDL cholesterol above guideline-recommended targets (average systolic BP over 2 visits > 140 mmHg, fasting LDL cholesterol > 2.0mmol/L or total: HDL cholesterol > 4.0)<br><br>Type of stroke (%): stroke (I: 45.4% C: 40.4%), TIA (I: 51.1% C: 55.9%), ocular (I: 3.5% C: 3.7%)<br><br>Mean age (SD): I: 68.8 (11.1); C: 66.6 (11.3) | Gender (% men): I: 60.8%; C: 55.2%<br><br>Ethnicity: not reported<br><br>Socio-economic or socio-demographic status: not reported
Interventions	Intervention details (components, length, frequency): the intervention group was managed by prescribing pharmacists who gave advice on lifestyle (exercise/low salt diet/smoking cessation/medication adherence), checked BP and LDL and initiated or titrated antihypertensive medication and/or lipid lowering therapy<br><br>Location: community<br><br>Mode of delivery: community<br><br>Personnel responsible for delivery: nurse and a prescribing pharmacist<br><br>Timing post-stroke: not stated<br><br>Control: the intervention group was compared to a group managed by a nurse who gave advice on lifestyle (exercise/low salt diet/smoking

	cessation/medication adherence), checked BP and LDL and then sent a list of the findings to the patients GP after each visit
Outcomes	Proportion of participants at 6 months who attained optimal blood pressure (≤ 140 mmHg systolic BP) and fasting LDL cholesterol ≤ 2.0 mmol/L
Notes	Analysis method: intention-to -treat<br>Risk of bias: low risk

Risk of Bias[10]

Bias	Author's Judgment	Support for Judgement
Random sequence generation	Low risk	Computer generated random numbers with variable sized blocked randomisation stratified by stroke prevention clinic to preserve allocation concealment
Allocation concealment	Low risk	Central allocation
Blinding of participants and personnel		
Blinding of outcome assessment		
Incomplete outcome data	Low risk	Missing data were imputed with a last observation carried forward strategy - assumed no change in BP or lipid level. Missing data has been imputed using appropriate methods
Selective reporting	Low risk	The protocol has been published previously
Other bias	Unclear risk	Unclear if recurrent events were presented as number of events rather than number of people with one or more event

McManus (2014)

Characteristics[10]

Methods	RCT<br><br>Unit of randomisation: participant
Participants	Place of recruitment: general practice patient records<br><br>Numbers randomised: total: 555 (I: 277; C: 278)<br><br>% Completing final follow-up: 81%<br><br>Inclusion criteria: > 35 years of age, at least 1 high risk conditions (including previous stroke/diabetes/stage 3 chronic kidney disease/cardiovascular disease), BP ≥ 130/80

	Type of stroke (%): not reported<br><br>Mean age (SD): I: 75.8 (12.4); C: 70.6 (14.5)<br><br>Gender (% men): I: 49%; C: 62%<br><br>Ethnicity: I: white 96% C: white 96%<br><br>Socio-economic or socio-demographic status: not given
Interventions	Intervention details: participants were trained how to take their own BP. They were also given a protocol of how to titrate antihypertensive medication. Participants were asked to take their BP twice daily and followed a protocol if not in range<br><br>Location: community<br><br>Mode of delivery: community<br><br>Personnel responsible for delivery: not reported<br><br>Timing post-stroke: not reported<br><br>Control: usual care without any specific BP targets
Outcomes	BP differences at 1 year for stroke subgroup analysis
Notes	Analysis method: mixed model adjusted for baseline BP, practise, sex and high risk group<br><br>Risk of bias: low

Risk of Bias[10]

Bias	Author's Judgment	Support for Judgement
Random sequence generation	Low risk	Minimisation used - adaptive stratified sampling that balances different groups or clinical trials simultaneously
Allocation concealment	Low risk	Central allocation
Blinding of participants and personnel		
Blinding of outcome assessment		
Incomplete outcome data	Low risk	Multiple imputations for missing values showed a marginally lower mean difference in systolic BP. Sensitivity analysis did not show any effect on the primary outcome
Selective reporting	Low risk	Based on a previously peer reviewed publication

Other bias	Low risk	The study appears to be free of other sources of bias

Meier (2013)

Characteristics[52]	
Methods	Prospective, multicenter, randomized, superiority clinical trial
Participants	Multicenter: 29 sites (Europe, UK, Australia, Brazil, and Canada)<br><br>414 participants < 60 years of age: 204 randomly assigned to closure and 210 to medical therapy<br><br>All participants had a PFO with a right-to-left shunt documented by TEE through the bubble study or color Doppler flow imaging, and had a history of no other identifiable cause of stroke, TIA, or peripheral thromboembolism proven both clinically and radiologically<br><br>Exclusion criteria: any identifiable cause for the thromboembolic event other than PFO; on chronic anticoagulant therapy for another disease other than paradoxical embolism; previous surgical or percutaneous PFO closure; severe central nervous system disease (i.e. Barthel-index < 50, mRS > 3); contraindication to medical or device therapy; follow-up over the next 5 years not possible (e.g. severe comorbid diseases with limited life expectancy, unreliable patients, etc)
Interventions	Closure of the PFO with the Amplatzer PFO Occluder (St Jude Medical) plus antithrombotic therapy versus medical therapy<br><br>Prophylactic antibiotic therapy was recommended during the periprocedural period. After the closure, all participants were given a standard antithrombotic regimen (acetylsalicylic acid 100 to 325 mg daily for at least 5 to 6 months, ticlopidine 250 to 500 mg daily, or clopidogrel at a dose of 75 to 150 mg daily 1 to 6 months). Ticlopidine or clopidogrel alone was recommended if intolerant to acetylsalicylic acid<br><br>In the medical therapy group, antithrombotic treatment was left to the discretion of the treating physician and could have included antiplatelet therapy or oral anticoagulation, provided that participants received at least 1 antithrombotic drug<br><br>If participants in the medical therapy group crossed over to the closure group, data were censored at the time of crossover<br><br>Mean follow up: 4.1 years in the closure group and 4.0 years in the medical therapy group
Outcomes	Participants were followed up in the hospital and in office visits at 6 months and annually for up to 5 years<br><br>Primary endpoint: composite of death, non-fatal stroke, TIA, or peripheral embolism

	Secondary endpoint: cardiovascular death, new arrhythmias (particularly new-onset atrial fibrillation), myocardial infarction, hospitalizations related to the PFO or its treatment, device problems, and bleeding
Notes	The trial was sponsored by St Jude Medical, who was not involved in the conduct of the trial, the writing of the manuscript, or the decision to submit the manuscript for publication but did provide organizational support for the adjudication of clinical events and the meetings of the data and safety monitoring board

Risk of Bias[52]

Bias	Author's Judgment	Support for Judgement
Random sequence generation	Low risk	The randomization schedule was computer generated with block sizes of 4 and stratification according to age of the participant and presence or absence of atrial septal aneurysm. The block size was unknown to investigators
Allocation concealment	Low risk	Participants were centrally randomized using a web-based randomization system (InterCorNet, Zurich, Switzerland)
Blinding of participants and personnel	High risk	Not blinded
Blinding of outcome assessment	Low risk	The assessors of endpoints were unaware of the study group assignments
Incomplete outcome data	High risk	Although the study clearly reported the number of participants who withdrew from the study and were lost to follow-up (31 (15%) participants in the closure group and 42 (20%) participants in the medical therapy group) and performed the ITT analysis, the high dropout rate compared with event rate and the difference in the dropout rate between the closure and medical therapy groups could still lead to high risk of attrition bias
Selective reporting	High risk	Selective reporting bias was introduced because the Clinical Events Committee

		discounted potential primary endpoint events more often in the medical therapy group than in closure group
Other bias	Unclear risk	Not reported

Meng (2012)

Characteristics[29]	
Methods	Study design: RCT<br><br>Generation of the allocation sequence: random number<br><br>Allocation concealment: unclear<br><br>Study grouping: parallel group<br><br>Masking: single-blinded (outcome assessor)<br><br>Analysis method: PP analysis<br><br>Total duration of study: unclear
Participants	103 participants (intervention = 51; control = 52)<br><br>China<br>2 centres<br>Setting: clinic and inpatient<br><br>Sex: both (58.8% men, 41.2% women)<br>Mean age: 60.6 years old<br><br>Inclusion criteria:<br>1) intracranial artery stenosis (measured as 50% by angiography or 70% by ultrasound, CTA or MRA) patient with corresponding stroke and TIA within 30 days from the start of the study;<br>2) age range 18 to 80 years;<br>3) NIHSS score 0-15, and mRS score 2-4 (mRS 0-1 were excluded); 4) ABCD2 score 6-7; 5) stable vital signs, normal hepatic and renal functions; 6) no haemorrhagic tendency; 7) Trial of Org 10172 in Acute Stroke Treatment (TOAST) 1 subtype
Interventions	Participants were randomly assigned to one of 2 groups:<br><br>Group 1: RIC group (n = 38), standard medical management plus RIC. RIC consisted of 5 cycles of bilateral upper limbs ischaemia (induced by inflating tourniquets to 200 mmHg) for 5 minutes followed by reperfusion for another 5 minutes, performed twice daily for a total of 300 consecutive days.<br><br>Group 2: control group (n = 30), standard medical management alone<br><br>Concomitant medications: antiplatelet agents, lipid control agents, and antidiabetic agents (if necessary)
Outcomes	Primary outcome: time point of the first stroke recurrence event

	Secondary outcome: time to which mRS recovers to 0-1<br><br>Other outcomes: RIC treatment intolerance
Notes	External blinded review of outcomes<br><br>Follow-up: 66%<br><br>Demographic: adequately reported and similar<br><br>Principle investigator: Xunming Ji, MD, PhD<br><br>Funding: this study was sponsored by the National Natural Science Foundation (30870854 and 30770741, China) and the Foundation of the Beijing High Level Health Systems Talented Technical Personnel Program (2009-03-02, China).<br><br>Conflicts of interest of authors: the corresponding author is one of the inventors of the electric auto-control device used in the study.

Risk of Bias[29]

Bias	Author's Judgment	Support for Judgement
Random sequence generation	Low risk	All participants were randomly divided into 2 groups according to a random number.
Allocation concealment	Unclear risk	The method of concealment was not described.
Blinding of participants and personnel	High risk	Doctors and participants were unmasked to the treatment protocol and allocation.
Blinding of outcome assessment	Low risk	Investigators responsible for evaluating the results of NIHSS, mRS scores, TCD, MRI, and SPECT, and the examiners who were responsible for testing plasma samples of the participants were masked to the treatment protocol and allocation.
Incomplete outcome data	High risk	35 (33.98%) participants lost to follow-up
Selective reporting	Low risk	Study authors published findings on all the predefined study outcomes.
Other bias	High risk	The corresponding author is one of the inventors of the electric autocontrol RIC device used in the study.

Mohr (2009)

Characteristics[53]	
Methods	Randomised trial.<br>Concealment by prearranged assignment and labelled boxes.<br>Blinding: double.<br>Results available for 98.5%.<br>Intention-to-treat analysis.
Participants	USA.<br>2206 patients.<br>Mean age 63 yrs; 59% male.<br>Ischaemic stroke of non-cardiac origin.<br>Time since stroke: < 30 days.<br>Comparability of groups: no significant differences in any characteristic.
Interventions	Rx: warfarin (INR 1.4 - 2.8).<br>Monitoring: first month 7 times, thereafter monthly INR measurements.<br>Compliance: not described, 16.3% below range.<br>Control: aspirin 325 mg daily.<br>Monitoring: idem.<br>Compliance: not described.
Outcomes	Death from any cause or recurrent ischaemic stroke.
Notes	Ex Crit: GOS < 3, TIA, contraindication for study medication.<br>Follow up: 2 years. 12 patients lost in treated group, 21 in control group.<br>Outcomes measured at end of planned follow up.

Risk of Bias[53]

**Bias**	**Author's Judgment**	**Support for Judgement**
Random sequence generation		
Allocation concealment	Low risk	A - Adequate
Blinding of participants and personnel		
Blinding of outcome assessment		
Incomplete outcome data		
Selective reporting		
Other bias		

Molina (2009)

Characteristics[54]	
Methods	Prospective RCT (2:1 ratio), placebo-controlled, single-blinded, multicentre, international safety dose-escalation clinical trial
Participants	35 patients within 3 hours and a proximal intracranial arterial occlusion diagnosed by transcranial doppler sonography with TIBI scale

	3-month follow-up of 33 patients<br><br>2 cases lost (1 from cohort 2 and 1 from control)<br><br>3 groups comparable in terms of pre-treatment characteristics
Interventions	(1) Target groups: standard iv tPA therapy + 90 minutes 2 MHz TCD + a dose escalation MRX-801 (Cohort 1: 1.4 ml; Cohort 2: 2.8 ml)<br><br>(2) Control group: standard iv tPA therapy + brief assessment by TCD + placebo
Outcomes	Primary endpoint: symptomatic intracerebral haemorrhage within 36 hours after tPA<br><br>Secondary endpoint: complete recanalisation at 120 minutes and time to complete recanalisation
Notes	

Risk of Bias[54]

**Bias**	**Author's Judgment**	**Support for Judgement**
Random sequence generation		
Allocation concealment	Low risk	Clearly described in the protocol
Blinding of participants and personnel	Low risk	Single blind study
Blinding of outcome assessment	Low risk	Single blind study
Incomplete outcome data	Unclear risk	2 patients unavailable for follow-up, reasons not clear
Selective reporting	Low risk	No problem
Other bias	Low risk	No problem

Nagapattinam (2015)

Characteristics[26]

Methods	RCT
Participants	Country: India<br><br>Setting: hospital<br><br>Age: adults (mean age: 44.9 years)<br><br>Sample size: 60 participants (40 in 2 experimental groups, 20 in control group, 1 dropout)

	Sex: 20 women, 40 men<br><br>Inclusion criteria: unilateral hemiplegic stroke, between 6 weeks and 6 months post-stroke, ischaemic stroke, age 18 to 60 years, both men and women, BRS 2 - 5, modified AS ≥ 1, voluntary extension of wrist and fingers of at least 10° from the resting position<br><br>Exclusion criteria: > 60 years of age, BRS 1 or 6, wrist and/or finger contracture, cardiac pacemaker or other metal implants, significant visual, auditory and cognitive impairment
Interventions	3 arms<br><br>1, 2 and 3: conventional therapy<br><br>1. Task-oriented MT: bilateral active wrist extension and fingers extension in mid-prone and pronated forearm, task-specific grasping and releasing of a bottle while looking to the image of the unaffected hand in the mirror<br><br>2. FES: electrodes placed on wrist extensors of the affected upper limb, participants were instructed to look into the opaque side of the mirror while the stimulation was given and was asked to perform the following exercises synchronously with the duty cycle of the stimulation, parameters of stimulation: frequency 35 Hz, pulse width 250 μs, symmetrical biphasic waveform, duty cycle of 5 secs on and 5 secs off, amplitude adjusted to maximal tolerance of the participant up to 90 mA<br><br>3. Task-oriented MT plus FES: participants were instructed to observe the mirror reflection and asked to perform simultaneous bilateral movements with the affected limb performing synchronously with the duty cycle of electrical stimulation<br><br>1, 2 and 3: 2 weeks, 6 days a week, 30 minutes daily MT, MT + FES, or FES<br><br>Date of intervention: not stated
Outcomes	Outcomes were recorded at baseline and after 2 weeks of therapy<br><br>1. ARAT
Notes	Based on published information<br><br>Funding source: not stated<br><br>Declarations of trialists' interests: none

Risk of Bias[26]

**Bias**	**Author's Judgment**	**Support for Judgement**
Random sequence generation	Low risk	Participants were randomly assigned by cards composed of odd and even numbers
Allocation concealment	Low risk	Concealed allocation by sealed envelopes
Blinding of participants and personnel		

Blinding of outcome assessment	High risk	Assessors were not blinded to group allocation
Incomplete outcome data	Low risk	All data were collected and analysed as allocated
Selective reporting		
Other bias		

O'Carroll (2013)

Characteristics[10]	
Methods	RCT<br><br>Unit of randomisation: participant
Participants	Place of recruitment: hospital stroke clinic and stroke unit<br><br>Numbers randomised: total: 62; I: 31; C :31<br><br>% Completing final follow-up: 87%<br><br>Inclusion criteria: first stroke or TIA; discharged home; prescribed secondary prevention antihypertensive medication; suboptimal medication adherence score<br><br>Exclusion criteria: requirement for help with taking medications; using a Dosette box; cognitive difficulties that precluded participation in the study<br><br>Type of stroke (%): not reported<br><br>Mean age (SD): I: 68 (11); C: 71 (11)<br><br>Gender (% male): 65%<br><br>Ethnicity: not reported<br><br>Socioeconomic or sociodemographic status (Scottish Index of Multiple Deprivation Quintile): 1 (highest deprivation) – 2%, 2 – 10%, 3 – 19%, 4 – 19%, 5 (lowest deprivation) – 51%
Interventions	Intervention details (components, length, frequency): 2 intervention sessions (approximately 30 minutes each) conducted 2 weeks apart: session 1 helped participants to establish a better medication-taking routine through completing individualised worksheets; session 2 reviewed participants' plans and addressed barriers to implementation; electronic recording of pill-taking for a duration of 3 months (researcher made monthly home visits to refill the electronic pill bottle)<br><br>Location: participants' homes or a research facility<br><br>Mode of delivery: home visits<br><br>Personnel responsible for delivery: researcher

	Timing post-stroke: < 3 months post-discharge<br><br>Control: participants attended 2 sessions with a researcher who "engaged the patient in non-medication related conversation in an attempt to provide some control for non-specific effects of attention/social contact"; electronic recording of pill-taking for 3 months
Outcomes	3 months: medication adherence; SBP; DBP
Notes	Analysis method: stated intention-to-treat<br><br>Risk of bias: low

Risk of Bias[10]

Bias	Author's Judgment	Support for Judgement
Random sequence generation	Low risk	"Participants were randomised to either the Intervention or Control group using web-based software set up by the Edinburgh Clinical Trials Unit."
Allocation concealment	Low risk	Web-based randomisation
Blinding of participants and personnel		
Blinding of outcome assessment		
Incomplete outcome data	Low risk	Missing data reported by group<br><br>Attrition I: 2/31 (2 hospitalised for non-stroke reasons); C: 2/31 (1 hospitalised for non-stroke reasons; 1 relocated)<br><br>Excluded from the analysis: (did not receive intervention): I: 2/31 (1 declined to use electronic pill bottle; 1 hospitalised for non-stroke reasons); C: 2/31 (2 hospitalised for non-stroke reasons)<br><br>Judgement: reasons for missing data reported and review authors judge that they are unlikely to be related to study outcomes

Selective reporting	Low risk	Protocol available and outcomes reported in the pre-specified way
Other bias	Low risk	The study appears to be free of other sources of bias

Palmer (2012)

Characteristics[12]	
Methods	Multicentre RCT stratified by severity of aphasia (mild/moderate/severe) and time poststroke (< 2 years/≥ 2 years), UK
Participants	Inclusion criteria: diagnosis of stroke and aphasia with word-finding difficulties as 1 of the predominant features as assessed by the Object and Action Naming Battery and the Comprehensive Aphasia Test (Druks 2000; Swinburn 2004, respectively). Participants were included only if they had the ability to repeat spoken words presented by the recruiting speech and language therapist. Eligible participants no longer received impairment-focused speech and language therapy enabling the computer treatment to be better isolated and evaluated. Participants with motor deficits poststroke were not excluded from the study. Where upper limb impairments made physical manipulation of the computer hardware difficult, assistive devices such as tracker balls or touchscreen computers were offered to enable access to the computer treatment<br><br>Exclusion criteria: 3 people with severe visual or cognitive difficulties reducing ability to use the computer programme were excluded from the study, tested by the ability to see and perform a simple, nonlanguage-based computer game<br>Group 1: 16 participants<br>Group 2: 17 participants<br><br>Details of participants are shown in Table 1
Interventions	1. Computer-mediated word finding therapy<br>Intervention: StepbyStep. Computer programmes developed for the treatment of aphasia provide exercises that can be carried out on a regular basis, targeting personal vocabulary and focusing on the patient's conversational needs. Such software has been reported to be useful in the provision of intensive independent language practice, giving rise to new opportunities to provide self management of continued aphasia treatment. There is growing evidence to suggest that the use of aphasia software can help to improve outcomes in language domains including reading, spelling, and expressive language.<br><br>Materials: usual language activities (as described in no SLT arm). In addition, they received speech and language therapy intervention delivered through independent use of a computer therapy programme (StepbyStep) configured by a speech and language therapist and supported by a volunteer. A library of more than 13,000 language exercises. Photographic images can be added to enable practice of personally relevant words such as names of people and pets. The intervention group practiced Object and Action Naming

battery words during the treatment (Druks 2000). In addition, participants in the intervention group practiced 48 words of personal relevance.

Procedures: each exercise follows steps progressing from listening to target words, producing words with visual, semantic, phonemic, or written letter/word cues through to saying the words in sentences. Speech and language therapist also provided initial instruction to the participant and caregiver on how to use the computer exercises and progress through the therapy steps. Volunteers provided assistance in using the software and hardware, encouragement to practice, and activities to promote use of the new words in daily life.

Provided by: speech and language therapist tailored the steps in the therapy process. Volunteers provided assistance in using the software and hardware, encouragement to practice, and activities to promote use of the new words in daily life. Volunteers contacted the participants once a week in the first month and at least once a month thereafter by telephone or home visit. Speech and language therapists trained. Volunteers included SLT students and existing volunteers from communication support groups. Volunteers were given a 3 h training session on how to use the StepbyStep programme and their role in supporting the intervention.

Delivery: 1-to-1, computer facilitated. Speech and language therapist supported face-to-face, at home. Regimen: per protocol: 20 minutes 3 d a week for 5 months (approximately 1500 minutes of practice time in total). Volunteers contacted the participants once a week in the first month and at least once a month thereafter by telephone or home visit. Total dose = 25 h therapy.

Tailoring: yes. Speech and language therapist tailored the steps in the therapy process as appropriate to the abilities and needs of the individual participant and provided initial instruction to the participant and caregiver on how to use the computer exercises and progress through the therapy steps.as appropriate to the abilities and needs of the individual.

Modification: tailored choice of words and level of difficulty.
Adherence: collected data via computer programme

2. No SLT
   Intervention: No formal SLT. Participation in everyday communication tasks and for some participants this may include attendance at communication support groups and conversation, reading, and writing activities that are part of everyday life.

   Materials: none.

   Procedures: none.

   Provided by: none (volunteers if attending local group)

   Delivery: not reported. Regimen: none.

	Tailoring: none. Modification: none.<br><br>Adherence: not applicable
Outcomes	Primary outcomes: feasibility of carrying out the study design and using self managed computer treatment supported by volunteers as a long-term intervention. Primary measures of feasibility were the recruitment rate, completion rates, and statistical variability. Outcomes indicating feasibility of the intervention included the percentage of the eligible population interested in receiving the intervention, the ability to offer the intervention per protocol (provision of computer software and volunteer support), and the ability of the participants to carry out the intervention per protocol (using the computer for at least 20 min 3 times a week for 5 months). Amount of practice time was stored by the StepbyStep computer software automatically and reviewed by a speech and language therapist at the end of treatment<br><br>Secondary outcome: measures of clinical and cost-effectiveness. Naming words that had been practiced in treatment at 5 and 8 months from baseline from the Object and Action Naming Battery (Druks 2000). Cost-effectiveness was investigated by estimating total costs (including intervention costs and other healthcare resource use costs collected using patient and caregiver diaries) and total quality adjusted life-years (QALYs) calculated using a pictorial version of the EQ5D26 questionnaire for an incremental cost-effectiveness ratio to be calculated<br><br>Data collection: baseline, at 1 month and 3 month. Follow-up at 5 and 8 months following treatment
Notes	Dropouts are detailed in Table 2<br>Statistical data included within the review meta-analyses

Risk of Bias[12]

Bias	Author's Judgment	Support for Judgement
Random sequence generation	Low risk	Web-based randomisation system. Stratified randomisation based on severity of aphasia (mild/moderate/severe) and time poststroke (< 2 years/> 2 years)
Allocation concealment	Low risk	Adequate
Blinding of participants and personnel	Low risk	Baseline assessments were conducted before randomisation, and assessment of outcomes undertaken blind to baseline and treatment allocation by blinded speech and language therapists
Blinding of outcome assessment	Low risk	Baseline assessments were conducted before randomisation, and

		assessment of outcomes undertaken blind to baseline and treatment allocation by blinded speech and language therapists
Incomplete outcome data	Low risk	Dropouts accounted for; ITT analysis employed
Selective reporting	Low risk	All prespecified outcomes reported
Other bias	Low risk	Groups were comparable at baseline in terms of severity, sex, age, time postonset<br><br>Pilot study so not possible to perform power calculation in advance but used data to calculate future sample size<br><br>No other obvious bias

Park (2013)

Characteristics[14]

Methods	Computer-generated randomisation sequence<br>Outcomes and participants blinded
Participants	Study in Korea<br>18 participants with stroke > 1 month<br>Baseline characteristics similar<br>Dysphagia confirmed by videofluoroscopy
Interventions	Rx: active high-frequency rTMS (5 Hz) at the contralesional intact cortex<br>C: sham rTMS
Outcomes	VDS, PAS
Notes	Exclusions: metal implants or a pacemaker in the body, history of seizures

Risk of Bias[14]

**Bias**	**Author's Judgment**	**Support for Judgement**
Random sequence generation	Low risk	Computer-generated randomisation sequence
Allocation concealment	Low risk	Automated assignment system
Blinding of participants and personnel	Low risk	Participants and outcome assessors blinded
Blinding of outcome assessment	Low risk	Participants and outcome assessors blinded
Incomplete outcome data	Low risk	None lost to follow-up
Selective reporting	Low risk	All outcomes reported
Other bias	Low risk	None identified

Poletto (2015)

Characteristics[38]	
Methods	Quote: "randomized, controlled, single-blind clinical trial compared early mobilization (within 48 h of symptom onset) with routine physical therapy"
Participants	Quote: "adult patients with CT- or MRI-confirmed ischemic stroke within 48 h of symptom onset who were admitted on weekdays from March to November 2012 to the acute vascular unit or general emergency unit of a large urban emergency department"<br><br>39 participants: 19 intervention, 20 control<br>Mean age: 65 years<br>13/37 (35%) men<br>0/37 (0%) haemorrhagic<br>Mean NIHSS score of 10.5<br>11 (30%) had mild stroke (NIHSS 0 to 5)<br>12 (32%) moderate stroke (NIHSS 6 to 11)<br>14 (38%) severe stroke (NIHSS > 11)
Interventions	What<br>Materials: unclear for staff but "patients and their families received a manual developed for the study, with guidance on positioning in bed and posture shifting to use at home after discharge"<br><br>Procedures: these "focused on getting out of bed, sitting in a chair, or standing (whenever and as soon as possible), and conducting functional training and motor relearning, pursuant to the Bobath concept. Exercises were performed bilaterally with at least 5 repetitions for each joint and each exercise, with emphasis on deficits on the impaired side. In addition, patients and their families received a manual developed for the study, with guidance on positioning in bed and posture shifting to use at home after discharge. Intervention patients were mobilized 5 times a week, once a day, for approximately 30 min per session, in addition to sitting out of bed for at least 30 min whenever possible"<br><br>Controls: they received "conventional physical therapy performed when requested by the staff. Therapy varied according to the patients' needs and the availability of physical therapists but generally included global motor exercises and respiratory therapy (ordinarily in bed). The duration of standard-care therapy sessions was approximately 15 min"<br><br>Who provided<br>Quote: "The program was carried out by trained physical therapists"<br><br>How<br>Quote: "The program was carried out by trained physical therapists"<br><br>Where<br>The acute vascular unit or general emergency unit of a large urban hospital in Brazil.<br><br>When and how much<br>Quote: "Exercises were performed bilaterally with at least 5 repetitions for each joint and each exercise, and emphasis on deficits on the impaired side.

	Intervention patients were mobilized 5 times a week, once a day, for approximately 30 min per session, in addition to sitting out of bed for at least 30 min whenever possible". "Sessions were held until hospital discharge or the 14th treatment day, whichever occurred first, regardless of where the patient was located"<br><br>Tailoring<br>Not stated<br><br>Modifications<br>Not stated<br><br>How well<br>Planned: "The session duration (in minutes) and number of sessions were recorded. Sessions were held until hospital discharge or the 14th treatment day, whichever occurred first, regardless of where the patient was located"<br>Actual: "Intervention patients received mobilization earlier and more frequently than controls (table 2). The median time from stroke to first mobilization was 43 h (vs. 72 h in the CG), and the total duration of mobilization during the hospitalization period was 135 min (IQR 85 to 213; vs. 0 min in the CG (IQR 0 to 50)). Only 2 patients did not initiate early mobilization (within 48 h) in the IG. Moreover, only 5 patients in the CG (26%) received physical therapy during hospitalization, with an average duration of 15 min per session. After hospital discharge, 57% of the patients in the IG and 37% in the CG underwent physical therapy sessions (P = 0.28). IG patients had more out-of-bed activities compared with controls (4.3 vs 0.3), initiating activities while still in the ED. Only the 5 patients who received physical therapy in the CG left their beds; all other controls remained bedbound during hospitalization"
Outcomes	The trialists "sought only to evaluate the feasibility and safety of the intervention. The primary outcome measures were functional capacity (mRS score 0 to 2) and mortality at 3 months.<br><br>Feasibility endpoints were<br>(a) time-to-first mobilisation, and<br>(b) total duration of motor physical therapy.<br><br>Safety endpoints were:<br>(a) complications during early mobilisation (first 48 h), i.e. symptomatic hypotension (syncope or presyncope) or neurological deterioration (defined as any worsening in NIHSS score);<br>(b) falls during hospitalisation and within 3 months of stroke;<br>(c) complications related to immobility (pneumonia, pulmonary embolism, and deep vein thrombosis) at 3 months, and<br>(d) death within 3 months.<br><br>Secondary outcomes were measured at 3 months (mRS score 0 to 1, mRS score 0 to 2, mean NIHSS score, and mBI ≥ 85)".
Notes	ClinicalTrials.gov NCT01694992<br>Reported as a pilot RCT that recruited from March to November 2012. Planned recruitment was for 174 participants (82 per group) but was limited by slow recruitment

Risk of Bias[38]

Bias	Author's Judgment	Support for Judgement
Random sequence generation	Low risk	Quote: "Randomization was performed online (randomization.com) by a study investigator, using a randomization plan stratified by blocks of varying sizes (blocks of 2, 4, or 6)"
Allocation concealment	Low risk	Quote: "randomly allocated to an intervention group (IG) or a control group (CG), with the allocation records stored in opaque, sealed, and sequentially numbered envelopes"
Blinding of participants and personnel	High risk	Quote: "Patients were informed that they would begin physical therapy on the first day of assessment (IG) or follow the hospital routine (CG)"
Blinding of outcome assessment	Low risk	Quote: "Baseline and postintervention measures were performed by a study investigator who remained blinded to group allocation. To ensure blinding of any monitoring neurologists, no notes of group allocation were made in the hospital's electronic medical record".
Incomplete outcome data	High risk	6/39 (15%) missing at 3 month follow-up
Selective reporting	Low risk	Primary outcome largely complete
Other bias		

Poppitt (2009)

Characteristics[55]	
Methods	RCT; parallel, placebo-controlled<br><br>Number of study sites: 1<br><br>Dates: randomisation from 29 July 2004 to 23 December 2005. Last follow-up in March 2006

Participants	Inclusion criteria: participants were aged > 45 years, clinically stable, and with a history of CT-confirmed first-ever or recurrent ischaemic stroke of probable noncardioembolic aetiology > 3 months before registration<br><br>Exclusion criteria: intolerance/hypersensitivity to fish/fish oils; current use of fish oil supplements; malabsorptive bowel diseases; or participation in a concurrent clinical trial<br><br>Sample size: 102 participants (total population, all with history of ischaemic stroke)<br><br>Participants randomised to intervention group: 51<br><br>Participants randomised to control group: 51<br><br>Sex: fish oil = 41 men and 10 women, placebo oil = 31 men and 20 women<br><br>Age, years (mean ± SD): fish oil group = 64 ± 10, placebo oil group = 65 ± 12<br><br>Time from last event (ischaemic stroke) to start of intervention (median and IQR): fish oil group = 1.18 (0.5, 2.1) years, placebo oil group = 0.90 (0.5, 2.3) years<br><br>Type of baseline stroke: ischaemic<br><br>Country: New Zealand<br><br>Ethnicity: not stated
Interventions	Comparison groups: fish oil versus placebo oil<br><br>Intervention: 3 g/day (3 × 1 g capsules) of fish oil (Hoki liver oil) supplement containing approximately 1.2 g/day total omega-3 PUFA<br><br>Control: matching placebo (palm and soy oils), 3 g/day<br><br>Compliance: capsule count and analysis of serum phospholipid fatty acid methyl esters<br><br>Duration of intervention: 12 weeks<br><br>Co-interventions: N/A
Outcomes	Primary outcome of study: change in serum triglycerides between baseline and 12 weeks<br><br>Relevant review outcomes measured: vascular-related death (due to MI), quality of life, mood<br><br>Available outcome data for stroke and/or TIA population only: vascular-related death (due to MI), quality of life, mood<br><br>Latest time point of assessment: 12 weeks

	Withdrawals or exclusions, n (stroke and/or TIA population only): fish oil = 4 refused follow-up, placebo oil = 2 refused follow-up, 1 death. Data missing at follow-up were imputed using last value carried forward<br><br>Study authors' contact status: replied, no data provided
Notes	Dietary information: participants advised to abstain from other fish oil supplements, and maintain their habitual diet<br><br>Funding: The Health Research Council of New Zealand. The Maurice and Phyllis Paykel Trust, New Zealand (LDL particle size and fatty acid analyses). Sea Dragon, New Zealand, provided the fish oil and Nutrition Laboratories, New Zealand, provided the placebo treatment and encapsulated the oils

Risk of Bias[55]

Bias	Author's Judgment	Support for Judgement
Random sequence generation	Low risk	Quote: "The randomization was a blocked (varying block sizes), unstratified design sequence."
Allocation concealment	Low risk	Quote: "Sequential allocation of packs to patients after confirmation of inclusion criteria [...] generated using an automated Internet-based system held at the Clinical Trials Research Unit, University of Auckland."
Blinding of participants and personnel	Unclear risk	Quote: "Treatment packs blinded for treatment"
Blinding of outcome assessment	Unclear risk	It does not state who assessed the outcomes
Incomplete outcome data	Low risk	Missing data imputed adequately. Participants lost to follow-up distributed evenly among groups
Selective reporting	Unclear risk	Protocol not available
Other bias	Low risk	None detected

Prange (2014)

Characteristics[46]

Methods	RCT
Participants	Recruited from an inpatient rehabilitation centre in the Netherlands<br><br>70 participants: 37 intervention, 33 control

	Inclusion criteria: first stroke 1-12 weeks ago, medically stable, display limited arm function but have active control of the elbow/shoulder of ≥ 15°, be free from other conditions or pain, be able to follow instructions and understand (and see) the visual game display<br><br>Exclusion criteria: treated with botulinum toxin and/or electrical stimulation to improve arm function before or during participation<br><br>Mean (SD) age: intervention group 60.3 (9.7) years, 58 (11.4) years<br><br>Stroke details: 78% ischaemic, 60% right hemiparesis<br><br>Timing post stroke: intervention group mean 7.3 (3.4) years, control group mean 6.8 (3.1) years
Interventions	VR intervention: training using a customised arm support program. Training consisted of playing games with the affected arm, supported by the device, working toward maximising movement ability with as little arm support as possible. The training involved mostly shoulder and elbow movements with exercises structured according to categorisation of the games for increasing difficulty (1D, 2D and 3D)<br><br>Conventional therapy: standard set of exercises to reflect usual physiotherapy and OT<br><br>Sessions were 30 min, 3 times/week for 6 weeks
Outcomes	Outcomes assessed post intervention<br><br>Fugl-Meyer assessment UE<br><br>Maximal reach distance<br><br>Stroke Upper Limb Capacity Scale (SULCS)<br><br>Visual Analogue Scale for arm pain<br>Intrinsic Motivation Inventory post training
Notes	NTR2539

Risk of Bias[46]

Bias	Author's Judgment	Support for Judgement
Random sequence generation	Unclear risk	Method not described
Allocation concealment	Low risk	Concealed envelopes
Blinding of participants and personnel		
Blinding of outcome assessment	Low risk	Blinded assessor
Incomplete outcome data	Low risk	Only 2 withdrawals and both withdrew due to inadvertent concurrent treatment

Selective reporting	Low risk	Outcomes reported as per trial registration
Other bias		

Rabadi (2008)

Characteristics[23]

Methods	Method of randomisation: sealed opaque envelope block randomisation of 10 patients<br><br>Double blind<br><br>Baseline prognostic factors were balanced between treatment groups
Participants	1 centre in US < 4 weeks of stroke<br>Mean age 74 years
Interventions	Rx: intensive nutritional supplementation (n = 51)<br>C: routine nutritional supplementation (n = 51)
Outcomes	Primary: change in total score on the FIM<br><br>Secondary: FIM motor and cognitive subscores, length of stay, 2-minute and 6-minute timed walk tests measured at admission and on discharge and discharge disposition
Notes	Exclusions: prior history of alcohol abuse, renal and liver disease, malabsorption, medically unstable or demented, terminally ill, participating any other therapeutic trial

Risk of Bias[23]

Bias	Author's Judgment	Support for Judgement
Random sequence generation	Low risk	Identical sealed opaque envelope containing block randomisation of 10 patients
Allocation concealment	Low risk	As above
Blinding of participants and personnel	Low risk	Double blind
Blinding of outcome assessment	Low risk	Double blind
Incomplete outcome data	Low risk	2 lost to follow-up
Selective reporting	Low risk	
Other bias	Unclear risk	

Rasmussen (2016)

Characteristics[40]

Methods	RCT<br>External list generated and managed by external person, blocks of 10<br>Opaque sealed envelopes

Participants	100 patients recruited from stroke unit of 1 university hospital, 1 to 3 days post stroke<br>Inclusion criteria: mRS 0 to 3 pre-stroke, living at home<br>Median age 81 (range 33 to 98) years, median BI 69 (0 to 100), median SSS 45 (11 to 58)
Interventions	Hospital out-reach multidisciplinary team, based within stroke unit. Co-ordinated and delivered low intensity (1 to 3 times per week) home based rehabilitation for a period of 1 month. All staff were skilled in stroke care and co-ordinated via weekly multidisciplinary meetings<br><br>Control: conventional discharge planning from combined acute/rehabilitation stroke unit and conventional after discharge care
Outcomes	At 90 days: dependency (mRS, BI, MAS, COPM), cognition (CT-50), quality of life (EQ-5D)<br>At 150 days: mortality, use of municipal services, hospital contacts, cost, carer satisfaction
Notes	The published report excluded some mild stroke patients that were included in the original unpublished report

Risk of Bias[40]

Bias	Author’s Judgment	Support for Judgement
Random sequence generation	Low risk	"In blocks of each ten patients" "Sealed envelopes containing a card with the word 'intervention' or 'control'" made by a research centre in the Capital Region of Denmark (Research Centre for Prevention and Health, Department of Planning Health and Quality)
Allocation concealment	Low risk	"Consecutively numbered and sealed envelopes containing a card with the word 'intervention' or 'control'"
Blinding of participants and personnel	High risk	Not explicitly stated but probably not possible to blind participants and personnel
Blinding of outcome assessment	High risk	"Blinded investigators were not used in the trial and all tests were performed by members of the multidisciplinary team".
Incomplete outcome data	Low risk	7 patients in the intervention group and 3 control patients 'dropped out' prior to discharge and were not included in the final analysis
Selective reporting	Low risk	All pre-specified outcomes appear to have been reported (unpublished)
Other bias		

Rehani (2015)

Characteristics[26]	
Methods	RCT
Participants	Country: India<br><br>Setting: outpatient<br><br>Age: adults (mean age: 54.8/57.9 years)<br><br>Sample size: 20 participants (6 in experimental group, 6 in control group, 8 dropped out)<br><br>Sex: not stated<br><br>Inclusion criteria: age 45 to 65 years, 1st episode of ischaemic and haemorrhagic stroke, stroke between 1 to 6 months, men and women, MMSE > 23, BRS 4 and 5<br><br>Exclusion criteria: any musculoskeletal disorders, neurological disorder other than stroke, visual impairment, systemic disease, non-cooperative patients, psychological problems
Interventions	2 arms<br><br>1 and 2: conventional therapy<br><br>1. MT: bilateral intransitive exercises such as hand opening, wrist extension and flexion, forearm pronation and supination, hand sliding on a flat surface while looking into the mirror<br><br>2. MRP: Motor relearning programme exercises for training of wrist extensors, extension of wrist and holding objects, training of supination of forearm, opposition of thumb, cupping of hand and training of manipulation of the objects<br><br>1 and 2: 4 weeks, 6 days a week, 30 minutes a day conventional therapy<br><br>1 and 2: additional 30 minutes a day MT or MRP<br><br>Date of intervention: not stated
Outcomes	Outcomes were recorded at baseline and after 4 weeks of therapy<br><br>1. CAHAI
Notes	Information based on unpublished data<br><br>Funding source: not stated<br><br>Declarations of trialists' interests: none

Risk of Bias[26]

Bias	Author's Judgment	Support for Judgement
Random sequence generation	Low risk	Participants were randomly assigned by computer-generated random-number sequence
Allocation concealment	Low risk	Concealed allocation by an independent investigator
Blinding of participants and personnel		
Blinding of outcome assessment	Unclear risk	Not stated
Incomplete outcome data	High risk	Dropouts were not included in analysis
Selective reporting		
Other bias		

Ross (2009)

Characteristics[41]

Methods	RCT
Participants	Australia<br><br>35 participants, 17 RTT, 18 control<br><br>Participants were recruited from a rehabilitation hospital (inpatients and outpatients). Date of recruitment not reported<br><br>Inclusion criteria: acquired brain injury within the past five years, over 18 years of age and notable hand impairment (i.e. a score of less than 80% on the Action Research Arm Test).<br><br>Exclusion criteria: co-existing injury or disease affecting hand function; unable to complete six weeks of training (i.e. for geographical, medical or psycho-social reasons). Patients with cognitive or physical problems precluding cooperation with the programme were also excluded.<br><br>Mean age: RTT 62.2 years (SD 18.2), control 60.8 years (SD 16.7)<br><br>48.6% male<br><br>Stroke details: 85.7% ischaemic, 48.72% right sided hemiplegia<br><br>Timing post stroke: RTT median 2.3 months (IQR 0.7 - 4.4), control median 0.7 months (IQR 0.3 - 3.0).

	Pre-intervention functional ability level: Scandinavian Stroke Scale: RTT 36.2 (SD 11.7), control 39.8 (SD 8.7)
Interventions	RTT intervention: all hand training was based on the principles of task-specific motor training and included repetitive practice of tasks which were individualised to the functional goals of each participant. Training was closely supervised on a 1-to-1 basis by 1 of a small number of experienced therapists The amount of actual practice performed in each session was carefully monitored, for this purpose a stopwatch was used to record the time spent performing hand activities. The aim was to achieve at least 45 minutes of repetitious practice in each session. Sessions were 1-hour with a therapist 5 x per week for six weeks = 30 hours<br><br>Comparison group: both groups continued to receive usual arm care which consisted of half an hour of motor training for the shoulder and elbow 5 x per week. A cup or splint was strapped to participants' hands to standardise inadvertent hand training<br><br>Usual care for both groups also consisted of strategies such as slings, wheelchair arm troughs and positioning programmes. In addition, participants in the control group had similar hand therapy as participants in the experimental group but for only 10 minutes, 3 x per week
Outcomes	Outcomes were recorded at baseline and 6 weeks (post treatment)<br><br>Upper limb functional outcome measures: Disability of Shoulder Arm and Hand Assessment, Action Research Arm Test, Summed Manual Muscle Test, Wolf Motor Function Test, long finger flexor extensibility<br><br>ADL outcome measures: Canadian Occupational Performance Measure
Notes	No significant differences at baseline

Risk of Bias[41]

Bias	Author's Judgment	Support for Judgement
Random sequence generation	Low risk	Computer-generated allocation schedule
Allocation concealment	Low risk	Concealed opaque consecutively numbered envelopes by a person not otherwise involved in the study. The allocation schedule and envelopes were kept off-site
Blinding of participants and personnel		
Blinding of outcome assessment	Low risk	Participants were instructed not to discuss their intervention or group allocation with assessors.

		The success of blinding was verified by asking assessors each time a participant completed the trial whether they had been unblinded. Assessors were then asked for their best guess at which group each participant had been allocated to
Incomplete outcome data	Low risk	2 dropouts in the control group unrelated to the intervention
Selective reporting	Unclear risk	No study protocol
Other bias		

Saal (2015)

Characteristics[16]	
Methods	RCT
Participants	Recruited from 2 acute hospitals in Germany<br><br>Inclusion criteria: age ≥ 18 years, ischaemic or haemorrhagic stroke for the first time (confirmed by imaging), main residency in the Federal States of Saxony-Anhalt, Saxony, or Thuringia, and able to speak German<br><br>Exclusion criteria: previous ischaemic or haemorrhagic stroke, alcoholism, National Institute of Health Stroke Scale (NIHSS) score > 25, and homelessness<br><br>Age, years: intervention group mean 68.1 (SD 12.6), control group 68.4 (12.7)<br><br>Gender: intervention group 34% men, control group 38% men<br><br>Time post-stroke: not reported but participants recruited from an acute hospital
Interventions	Telerehabilitation intervention: in-depth assessment and stroke support service provided by a nurse and physiotherapist. The stroke support service comprised stroke outreach support, educational sessions, and written patient information and was directed to both the patient and the next of kin. The stroke outreach support included home visits and telephone contacts and was individually tailored based on an agreement between the stroke support organiser and the patient and carer. The number of contacts between stroke support organiser and patient/carer was: 12.31% of contacts face-to-face and 61% via telephone; the remaining were written communications per email and normal post, or patient educational sessions.<br><br>Control intervention: usual care

Outcomes	Timing of outcome assessment: pre (prior to discharge from acute care), baseline (4 weeks after discharge prior to randomisation), and post (12 months after randomisation)<br><br>Measures: Stroke Impact Scale (physical function domain), WHOQOL-BREF, Geriatric Depression Scale, Symptom Checklist 90 Revised, health service use
Notes	

Risk of Bias[16]

Bias	Author's Judgment	Support for Judgement
Random sequence generation	Low risk	Computer-generated
Allocation concealment	Low risk	Sequentially numbered, opaque, sealed and stapled envelopes
Blinding of participants and personnel	Low risk	Blinded outcome assessment
Blinding of outcome assessment	High risk	Withdrawals across both groups but more in the usual care group
Incomplete outcome data	Low risk	Registration as clinical trial performed in advance. All outcomes reported
Selective reporting	Low risk	No other sources of bias noted
Other bias		

Sandset (2011)

Characteristics[4]	
Methods	Double-blind, placebo-controlled, blinded-endpoint<br><br>Randomisation (1:1) by secure Internet website with both participants and investigators masked to treatment allocation
Participants	International (9 countries), multicentre (146 sites)<br><br>2029 participants. T: 1017, C: 1012<br><br>Mean age T: 70.8 years, C: 71.0 years<br><br>Male T: 60%, C: 56%<br><br>Inclusion: IS or ICH<br><br>Enrolment within 30 hours of stroke onset and elevated SBP > 140 mm Hg<br><br>FU: 25 losses

Interventions	T: candesartan (Astra Zeneca), doses increasing from 4 mg on day 1 to 16 mg on days 3 to 7<br><br>C: placebo<br><br>Rx: 7 days
Outcomes	BP measured twice with validated automated blood pressure monitor (UA-767 Plus 30, A&D Medical, San Jose, CA, USA).<br><br>Primary: composite of vascular death, nonfatal MI or non-fatal stroke in first 6 months; mRS at 6 months<br><br>Secondary: SSS at day 7 and BI; death from all causes; vascular death; recurrent stroke; MI; stroke progression
Notes	Exclusion: CI to, or current treatment with ARA; markedly reduced consciousness (SSS consciousness score ≤ 2); clear indication for an ARA during treatment period; clear indication for antihypertensive treatment during the acute phase of stroke; premorbid modified mRS ≥ 4; life expectancy of 12 months or less; patient unavailable for follow-up; pregnancy or breastfeeding

Risk of Bias[4]

Bias	Author's Judgment	Support for Judgement
Random sequence generation	Low risk	Randomisation was done by secure Internet system
Allocation concealment	Low risk	Both candesartan and placebo tablets were identical in appearance. Central web-based allocation ensured allocation concealment. If Internet was not available, investigators used the drug pack with the lowest pack number
Blinding of participants and personnel	Low risk	Double-blind
Blinding of outcome assessment	Low risk	Double-blind
Incomplete outcome data	Low risk	No differences between trial groups
Selective reporting	Low risk	All pre-specified outcomes were reported; no differences between trial groups
Other bias	Unclear risk	No other biases found

Saposnik (2010)

Characteristics[46]	
Methods	RCT
Participants	Recruited from a subacute rehabilitation facility in Toronto, Canada<br>22 participants: 11 intervention, 11 control<br><br>Inclusion criteria: 18-85 years with first time ischaemic or haemorrhagic stroke within the last 6 months, Chedoke McMaster scale (UE) score of > 3 in the arm or hand<br><br>Exclusion criteria: unable to follow instructions, pre-stroke Modified Rankin Score of ≥ 2, medically unstable or with uncontrolled hypertension, severe illness with life expectancy of < 3 months, unstable angina, recent MI (within 3 months), history of seizures or epilepsy, participating in another clinical trial involving an investigational drug or physical therapy, any condition that might put the patient at risk (for example, known shoulder subluxation)<br>Mean age: intervention group 55 years, control group 67 years<br>64% men<br>Stroke details: 45% right hemiparesis<br><br>Timing post stroke: intervention group mean (SD) 27 (16) d, control group 23 (9) d
Interventions	VR intervention: participants used the Nintendo Wii gaming console playing 'Wii sports' and 'Cooking Mama'<br><br>Control intervention: leisure activities including cards, bingo and Jenga<br><br>Sessions were 60 min for 8 sessions (8 h total)
Outcomes	Outcomes recorded at baseline, post intervention and at 1 month<br><br>Upper limb function and activity outcomes (arm): abbreviated version of the Wolf Motor Function Test<br><br>Upper limb function and activity outcomes (hand): Box and Block test, Grip strength (kg)<br><br>Participation restriction and quality of life: Stroke Impact Scale (hand function, composite function, perception of recovery)<br><br>Adverse events reported<br><br>Other outcomes: therapy time
Notes	

Risk of Bias[46]

Bias	Author's Judgment	Support for Judgement
Random sequence generation	Low risk	Participants were randomly allocated using a basic computer random number generator

Allocation concealment	Unclear risk	Unclear
Blinding of participants and personnel		
Blinding of outcome assessment	Low risk	Blind
Incomplete outcome data	Low risk	Some attrition was reported. Outcomes were calculated based on the number of participants and there was no reporting of imputation of data. ITT analysis was completed
Selective reporting	Low risk	Reports on all measures reported in the study protocol paper
Other bias		

Saposnik (2016)

Characteristics[46]	
Methods	RCT
Participants	Recruited from rehabilitation units in 4 countries: Canada, Argentina, Peru, Thailand<br><br>141 participants: 71 intervention group, 70 control group<br><br>Inclusion criteria: 18-85 years with first time ischaemic stroke within 3 months of enrolment and with mild to moderate motor disability (Chedoke McMaster Stroke Assessment stage > 3)<br><br>Exclusion criteria: no disability in the UE (arm components of the Chedoke McMaster scale = 7), were unable to follow instructions, pre-stroke Modified Rankin score of ≥ 2, medically unstable or uncontrolled hypertension; severe illness with a life expectancy of < 3 months, unstable angina or MI within 3 months, history of seizures or epilepsy (except for febrile seizures of childhood); participating in another clinical trial involving an investigational drug or physical therapy or had any condition that might put the patient at risk (e.g. known shoulder subluxation)<br><br>Mean (SD) age: intervention group 62 (13) years, control group 62 (12) years<br><br>Stroke details: 100% ischaemic; right hemiparesis 47%<br><br>Timing post stroke: intervention group mean 27 d, control group mean 24.5 d
Interventions	VR intervention: Nintendo Wii Sports and Game Party 3. Progression through the intervention allowed participants to choose some specific activities within those games (last 3 min of the intervention) based on their

	capabilities and interest with the goals of enhancing flexibility, ROM, strength and co-ordination of the affected arm<br><br>Control intervention: recreational therapy with progression through activities such as cards, bingo, Jenga or a ball game<br><br>Administered 1:1 by a rehabilitation therapist<br><br>Sessions were 60 min, 5 times/week for 2 weeks
Outcomes	Outcomes were recorded at 2 weeks (post intervention) and 4 weeks<br><br>Abbreviated Wolf Motor Function Test<br><br>Box and Block Test<br><br>Quality of life after stroke - Stroke Impact Scale<br><br>Functional Independence Measure, Barthel Index, Modified Rankin Scale<br><br>Grip strength (dynamometer)<br><br>Hand function - Stroke Impact Scale<br><br>Adverse events reported
Notes	NCT01406912

Risk of Bias[46]

**Bias**	**Author's Judgment**	**Support for Judgement**
Random sequence generation	Low risk	Computer-generated assignment
Allocation concealment	Low risk	Assignment at the point enrolment
Blinding of participants and personnel		
Blinding of outcome assessment	Low risk	Blinded assessor
Incomplete outcome data	Low risk	ITT analysis conducted. Details of withdrawals reported transparently
Selective reporting	Low risk	All outcomes reported
Other bias		

Schick (2017)

Characteristics[26]

Methods	RCT
Participants	Country: Austria/Germany<br>Setting: 3 inpatient rehabilitation centres<br><br>Age: adults (mean age: 63 years)<br><br>Sample size: 32 participants (15 in experimental group, 17 in control group, 2 dropouts)<br><br>Sex: 13 women, 19 men<br><br>Inclusion criteria: had suffered their 1st ischaemic or haemorrhagic stroke within 6 months prior to entering the study, had severe (FM-UE ≥ 18 ≤ 33 points) or very severe arm paresis (FM-UE ≤ 17 points) as assessed with the Fugl-Meyer Assessment, had arm/hand function that could be electrically stimulated and EMG-triggered pulses that could be elicited, reported to have been independent in their activities of daily living before stroke, reported to have had full functionality of their upper extremities before the stroke, and were able to understand study tasks and test instructions<br><br>Exclusion criteria: were pregnant, had an implanted cardiac pacemaker, defibrillator, brain stimulation, drug pump, or metal implant, had wounds, thrombosis, or phlebitis in the stimulation area; severe forms of Dupuytren's contracture, dementia and concomitant severe neurological diseases; or profound neurocognitive deficits
Interventions	2 arms<br>1 and 2: conventional therapy<br><br>1. Multi-channel EMG-triggered electrostimulation (EMG-MES) + MT: electrostimulation with a device (4 muscle stimulation channels and up to 2 EMG measurement channels), EMG-triggered pulses for the affected and the unaffected sides were measured and elicited exclusively via the unimpaired side to initiate synchronous bilateral forearm and hand movements (grip and release without objects), standard current frequency was between 30 and 35 Hz, participants were asked to observe the grasping movements of their unaffected limb in the mirror and actively imagine that they were movements of their affected limb<br><br>2. EMG-MES: same device and protocol (same pulse intensity, same standard current frequency) participants observed directly their grip and release movements on the affected side1 and 2: 3 weeks, 5 days a week conventional therapy<br><br>1 and 2: 3 weeks, 5 days a week, 30 minutes a day EMG-MES + MT or EMG-MES<br><br>Date of intervention: September 2013 to August 2014
Outcomes	Outcomes were recorded at baseline and after therapy<br><br>1. FM-UE (0 - 66 points)

	2. German language version of the Rivermead Assessment of Somatosensory Performance (RASP-DT) * BBT * GAS * BI
Notes	Abstract published in 2015, full-text publication received in 2017<br><br>Funding source: not stated<br><br>Declarations of trialists' interests: the first author was employed by MED-EL after the end of study (STILLWELL, one of the distributors and developers of the stimulation device which was used in the study) and gives seminars for EMG-triggered multichannel electrostimulation; the senior author delivers seminars and has authored two manuals on MT; the other authors declared no potential conflicts of interest

Risk of Bias[26]

Bias	Author's Judgment	Support for Judgement
Random sequence generation	Low risk	Participants were randomly assigned by computer-generated random-number sequence and block randomisation
Allocation concealment	Low risk	Concealed allocation by sealed envelopes and independent investigator
Blinding of participants and personnel		
Blinding of outcome assessment	Low risk	Assessor were blinded to group allocation
Incomplete outcome data	Low risk	All data included as intended
Selective reporting		
Other bias		

Shaw (2014)

Characteristics[4]

Methods	Double-blind parallel-group external pilot controlled trial<br><br>Both active and control group tablets were blister packed and placed in identical boxes. Each box had a unique study number according to the randomisation code (intervention and control in 1:1 ratio)
Participants	UK, single centre<br><br>14 participants. T: 6 C: 8<br><br>Median age 73 years<br><br>Male T: 7, C: 7

	Inclusion: conscious ("A" on AVPU scale), ≥ 40 years with new unilateral arm weakness thought to be due to be acute stroke and SBP > 160 mm Hg on 2 consecutive seated or lying readings taken 5 to 10 minutes apart<br><br>Enrolment within 3 hours of symptom onset<br><br>FU: not completed for 1 participant
Interventions	T: lisinopril (Modepharma) 5 mg sublingual and second dose of 5 mg given po, sublingual or via nasogastric tube<br><br>C: matched placebo (Haupt Pharma Wuelfing)<br><br>Rx: 7 days
Outcomes	BP measured seated or supine 5 to 10 minutes apart before randomisation<br><br>Primary: feasibility-recruitment rate, compliance with data collection<br><br>Secondary: change in BP for 7 days (BP measurement methodology during study schedule not given); NIHSS at days 3 and 7; BI, mRS, renal function, death at day 7
Notes	Exclusion: age < 40 years; females, pregnant, lactating or at risk of pregnancy; females < 56 years of age consented by a relative; suspected stroke without unilateral arm weakness; unable to establish whether stroke onset time was within the last 3 hours; SBP < 160 mm Hg; reduced level of consciousness below "A" on AVPU scale; patient not being transported to PIL-FAST trial site; absence of participant or next of kin consent; known to be taking ACE inhibitor or angiotensin II receptor blocker medication already; known sensitivity to lisinopril or other ACE inhibitor medication; pulse > 120 bpm; seizure; hypoglycaemia; unable to walk independently prior to stroke; obvious understanding or memory problems when next of kin is absent; significant head trauma or brain surgery in the last 3 months; known renal failure, liver failure (or currently jaundiced); uncontrolled heart failure (breathlessness at rest); receiving palliative care for known malignancy; participating in a clinical trial assessing a study drug

Risk of Bias[4]

**Bias**	**Author's Judgment**	**Support for Judgement**
Random sequence generation	Low risk	Randomisation list created by independent statistician
Allocation concealment	Low risk	Both lisinopril and placebo were supplied in identical boxes and each box was packaged into a secondary trial pack. Each pack carried a unique study number linked to the randomisation code

Blinding of participants and personnel	Low risk	Double-blind
Blinding of outcome assessment	Low risk	Outcomes were assessed by investigators centrally masked to treatment allocation
Incomplete outcome data	Low risk	All participants accounted for, both groups have similar dropout rates; clinical reasoning given
Selective reporting	Low risk	All pre-specified outcomes were reported
Other bias	Low risk	No other biases evident

Sheffler (2013)

Characteristics[11]	
Methods	Study design: RCT<br><br>Instruments used: lower limb portion of the Fugl-Meyer Assessment, mEFAP, SSQoL, gait analysis with Vicon system<br><br>Study design as described in the article: "Single-blinded randomized controlled trial"<br><br>Study duration: not stated<br><br>Year of study: not stated
Participants	Inclusion criteria: age ≥ 18 years, ≥ 12 weeks poststroke with unilateral hemiparesis and ankle dorsiflexion strength of ≤ 4/5 on the Medical Research Council (MRC) scale. Participants were required to ambulate ≥ 30 feet without an AFO, score ≥ 24 on the BBS, and demonstrate correction of foot drop using a PNS without evidence of knee hyperextension during stance.<br><br>Exclusion criteria: lower extremity edema, skin breakdown, or absent sensation; serious cardiac arrhythmias, pacemakers or other implanted electronic systems; pregnancy; uncontrolled seizure disorder; concomitant lower motor neuron dysfunction and non-stroke upper motor neuron dysfunction; uncompensated hemineglect; sensory or motor peripheral neuropathy; fixed ankle plantarflex or contracture; or lower extremity botulinum toxin injection within the 3 months prior to study enrollment<br><br>Age: MN group mean age (± SD): 52.8 years (± 12.2); control group mean age (± SD): 53.2 years (± 10.1)<br><br>Country: USA

	Sample size: 110 participants<br><br>Sex: MN group: 30 men and 24 women; control group: 37 men and 19 women<br><br>Time poststroke: > 12 weeks poststroke. MN group mean time poststroke (± SD): 44.7 months (± 97.5); control group mean time poststroke (± SD): 44.9 months (± 79.2)<br><br>Type of stroke: MN group: 13 embolic, 17 thrombotic, 9 lacunar, and 15 hemorrhagic; control group: 12 embolic, 23 thrombotic, 6 lacunar, and 15 hemorrhagic
Interventions	Motor neuroprosthesis<br><br>• Intervention: MN group used Odstock Dropped-Foot Stimulator (ODFS) device up to 8 hours per day once device safety was demonstrated. In the first 5 weeks the Functional Training phase (2 x 1-hour sessions per week) took place, in which participants were trained to use the MN device for home and community mobility with an assistive device, such as a straight cane, quad cane, or walker, if needed. Activities included passive and active range-of-motion exercises, lower extremity strengthening, standing balance and weight-shifting activities to the affected limb with transition to least-restrictive assistive device, and refinement of a reciprocal gait pattern. Exercises were done with multiple repetitions with an increase in difficulty and decrease in cues, with and without the MN, as appropriate. In the last 7 weeks the Post-Functional Training phase (3 x 1-hour sessions) took place, in which device function, application, and usage guidelines were reviewed with each participant to maximize MN compliance.<br>• Number of participants: 54<br>• Device: a single-channel surface stimulator with surface electrodes. The stimulation was triggered by an insole pressure sensor.<br>• Duration of exposure: 12 weeks of independent use of MN<br>• Follow-up: 12 and 24 weeks' post-treatment<br>• Place of application of intervention: lower limb<br><br>Another assistive technology device<br><br>• Intervention: control group consisted of treatment with AFO or no device up to 8 hours per day. In the first 5 weeks the Functional Training phase (2 x 1-hour sessions per week) took place, in which participants were trained to use the AFO device for home and community mobility with an assistive device, such as a straight cane, quad cane, or walker, if needed. Activities included passive and active range-of-motion exercises, lower extremity strengthening, standing balance and weight-shifting activities to the affected limb with transition to less restrictive assistive device, and refinement of a reciprocal gait pattern. Exercises were done with multiple repetitions with an increase in difficulty and decrease in cues, with and without the AFO, as appropriate. In the last 7 weeks the Post-Functional Training phase (3 x 1-hour sessions) took place, in which device

	function, application, and usage guidelines were reviewed with each participant to maximize AFO compliance.<br>• Number of participants: 56 (48 participants used AFO as usual care, and 6 participants used no device)<br>• Device: a custom-molded hinged AFO with plantarflexion block that was fabricated using conventional techniques<br>• Duration of exposure: 12 weeks of independent use of AFO<br>• Place of application of intervention: lower limb
Outcomes	Activities involving limbs: mEFAP (s)<br><br>• Outcome type: continuous<br>• Assessment time point: baseline, 12 weeks, 12 weeks post-treatment, and 24 weeks post-treatment<br>• Device at assessments: baseline, 12 weeks, 12 weeks post-treatment, and 24 weeks post-treatment assessments performed without MN<br><br>Activities involving limbs: walking speed (m/s)<br>• Outcome type: continuous<br>• Assessment time point: baseline, 12 weeks, 12 weeks post-treatment, and 24 weeks post-treatment<br>• Device at assessments: baseline, 12 weeks, 12 weeks post-treatment, and 24 weeks post-treatment assessments performed without MN<br><br>Participation scale of HRQoL: SSQoL<br>• Outcome type: continuous<br>• Assessment time point: baseline, 12 weeks, 12 weeks post-treatment, and 24 weeks post-treatment<br><br>Adverse events: dropouts during the intervention period<br>• Outcome type: binary<br><br>Adverse events: falls<br>• Outcome type: binary
Notes	This study consisted of 2 articles (Sheffler 2013a; Sheffler 2015).

Risk of Bias[11]

Bias	Author's Judgment	Support for Judgement
Random sequence generation	Low risk	The investigators described that envelopes were used as a random component in the sequence generation process.
Allocation concealment	Unclear risk	Although the investigators stated that the randomization sequence was concealed, there is no mention as to

		whether the envelopes were sealed or not.
Blinding of participants and personnel	High risk	There was no blinding of participants and personnel.
Blinding of outcome assessment	Low risk	Quote: "blinded outcomes assessor".
Incomplete outcome data	Low risk	ITT analysis was performed.
Selective reporting	Low risk	The study protocol is available, and all of the study's prespecified (primary and secondary) outcomes that are of interest in the review have been reported in the prespecified way.
Other bias	Low risk	No other bias detected.

Simpson (2009)

Characteristics[56]	
Methods	Randomised, double-blind, 3-arm, placebo-controlled study<br><br>Due to mandate of review, only the tizanidine and placebo groups are reported - the botulinum toxin arm is not reported
Participants	USA<br><br>40 adults (19 female, 21 male) of which 32 had a diagnosis of stroke were randomly divided into 2 groups (the other 8 had TBI)<br><br>Tizanidine group had mean age of 51.9 years (SD 17.3)<br><br>Placebo group had mean age of 51.3 years (SD 14.7)
Interventions	Tizanidine starting dose of 2 mg/day titrated up by 4 mg every 3 to 4 days to a maximum of 36 mg/day; also received at least 2 placebo injections to forearm muscles<br><br>Placebo group provided with placebo oral medication; also received at least 2 placebo injections to forearm muscles
Outcomes	Modified Ashworth Scale at the wrist and finger flexors<br><br>Modified Ashworth dichotomised

	Disability Assessment Scale: 1 domain was identified by the participant and assessor as the primary therapeutic target<br><br>Modified Frenchay Scale, 10-Metre Walk Test, contralateral grip strength, and Finger Tap Test<br><br>Other measures included Epworth Sleepiness Scale, Geriatric Depression Scale, and Letter-Number Sequencing
Notes	We contacted 2 of the authors by email but received no response

Risk of Bias[56]

Bias	Author's Judgment	Support for Judgement
Random sequence generation	Unclear risk	There is no description of how sequence generation was achieved
Allocation concealment	Low risk	The review authors assume that allocation concealment was maintained
Blinding of participants and personnel	Low risk	Both participants and injectors were blinded to treatment. Clear explanation provided
Blinding of outcome assessment	Low risk	The outcome assessor was blinded to the treatment
Incomplete outcome data	Low risk	Attrition was documented for all groups and explanations provided. At primary endpoint 93% of participants were assessed. We noted that there was a high attrition rate between week 6 and week 22
Selective reporting	High risk	Planned data for report were presented at primary endpoint in the report; however, there has been no follow-up report of Modified Frenchay Scale and 10-Metre Walk Test results from the study
Other bias		

Spielmann (2016)

Characteristics[57]	
Methods	Multi-centre RCT
Participants	Country: the Netherlands<br><br>58 participants (40 men, 18 women); mean age (SD): 58 (10) years in the experimental group and 60 (10) years in the control group; time since stroke (SD): 1.4 (0.5) months in the experimental group and 1.6 (0.7) months in the control group; educational level: 12 (3) years in the experimental group and 13 (3) in the control group; mean aphasia severity according to shortened token test at baseline (SD):18.8 (7.9) in the experimental group and 19.1 (9.0) in the control group<br><br>Inclusion criteria: aphasia after stroke, time post onset < 3 months, age 18 to 80 years, native speaker of Dutch, right-handed<br><br>Exclusion criteria: subarachnoid haemorrhage, prior stroke resulting in aphasia, brain surgery in the past, epileptic activity in the past 12 months (or anti-epileptic medications), excessive use of alcohol/drugs, premorbid (suspected) dementia, premorbid psychiatric disease affecting communication, severe non-linguistic cognitive disturbances impeding language therapy, pace maker, global aphasia, defined as Shortened Token Test < 9 and score 0 on the Aphasia Severity Rating Scale, severe Wernicke's aphasia, defined as Shortened Token Test < 9 and score 0 or 1 on the Aphasia Severity Rating Scale, residual aphasia, defined as Shortened Token Test > 28 and score 4 or 5 on the Aphasia Severity Rating Scale and Boston Naming Test > 150
Interventions	2 arms; each group received word-finding therapy for 45 minutes per day on 5 consecutive sessions; 225 minutes per week:<br><br>- A-tDCS for 1 mA for the first 20 minutes<br><br>- S-tDCS for the first 20 minutes
Outcomes	Outcomes were recorded at baseline, at the end of intervention phase and 6-month follow-up:<br><br>Primary outcome measures:<br><br>- Boston Naming Test (before and after each intervention week and at 6-month follow-up<br><br>Secondary outcome measures:<br><br>- naming performance on trained and untrained items (in per cent, after each intervention week)<br><br>- Aphasia severity rating scale (after the second intervention week and at 6-month follow-up)

	- ANELT (after the second intervention week and at 6-month follow-up)<br><br>- Wong-Baker Faces 5-point pain rating scale for assessing adverse events
Notes	

Risk of Bias[57]

Bias	Author's Judgment	Support for Judgement
Random sequence generation	Low risk	Quote: "One of the authors (MHK, epidemiologist), not involved in selecting, testing, or treating participants, performed the randomization using an online random number generator."
Allocation concealment	Low risk	Quote: "The random numbers were combined with 5-number codes from the tDCS manual for active or sham-tDCS. These codes were concealed in opaque envelopes;[...]"
Blinding of participants and personnel	Low risk	Quote: "[...] a unique code, which did not disclose whether active or sham tDCS would be provided, was used for each individual and was opened at the first therapy session by the speech and language therapists (SLTs)."
Blinding of outcome assessment	Low risk	Outcome assessors were blinded
Incomplete outcome data	Low risk	1 out of 26 participants of the experimental group (4%) and 1 out of 32 (3%) participants of the control group did not receive allocated intervention due to reasons supposed to be unrelated to the intervention
Selective reporting	High risk	In comparison to the published protocol results for the following outcomes were not presented in this publication so far: SAQOL, Euroqol-5D, care consumption, Werk en zorg

		vragenlijst (Health care consumption and labour productivity), laterality index, fMRI. Other outcomes: demographic data (socio-economic status), size and location of the lesion (fMRI), participation: CIQ – overall functioning: Barthel Index
Other bias	Low risk	No other bias identified

Standen (2016)

Characteristics[46]	
Methods	RCT
Participants	Study took place in the UK<br><br>27 participants: 17 intervention, 10 control<br><br>Inclusion criteria: 18 years or over, no longer receiving any other intensive rehabilitation and still had residual upper limb dysfunction<br><br>Exclusion criteria: failure to meet above criteria<br><br>Mean (SD) age: intervention group 59 (12.03), control group 63 (14.6) years<br><br>59% male<br><br>Timing post stroke: intervention group mean (SD) 38 (41.28) weeks, control group 24 (36.26) weeks
Interventions	Virtual reality intervention: virtual glove which translates the position of the hand into gameplay. Participants were instructed to use the program at home<br><br>Control intervention: usual care (no specific intervention)<br><br>Sessions were 20 minutes, 3 times a day for 8 weeks (approximately 52 hours)
Outcomes	Outcomes recorded at baseline, 4 weeks and post-intervention (8 weeks)<br><br>Upper limb function outcome: Wolf Motor Function Test, Nine Hole Peg Test<br><br>Other: Motor Activity Log<br><br>Activity outcomes: Nottingham Extended Activities of Daily Living Scale (NEADL)

Notes

Risk of Bias[46]

Bias	Author's Judgment	Support for Judgement
Random sequence generation	Low risk	Computerised random number generator
Allocation concealment	Low risk	Managed externally
Blinding of participants and personnel		
Blinding of outcome assessment	Low risk	Blinded to allocation
Incomplete outcome data	High risk	Large number of drop outs in the intervention group
Selective reporting	Low risk	Unpublished data obtained via personal communication
Other bias		

Stoykov (2009)

Characteristics[58]

Methods	Randomised controlled trial<br>Stratified into 2 impairment levels based on Fugl-Meyer upper extremity scores (19 to 28 or 29 to 40)<br>Within each group of 12 participants a randomised computer-generated list provided group assignment
Participants	24 participants<br><br>Inclusion criteria: Fugl-Meyer upper extremity score 19 to 40, ≥ 6 months post-stroke, cortical or subcortical lesion, ability to follow 2-step commands, 18 to 80 years of age, no evidence of cerebellum or brainstem involvement, no evidence of field cut, no evidence of neglect, ability to give informed consent, no symptomatic cardiac failure or unstable angina, no uncontrolled hypertension, no significant orthopaedic or pain conditions in affected upper extremity, no severe obstructive pulmonary disease
Interventions	Group 1 (12 participants): unilateral training<br><br>Group 2 (12 participants): bilateral training<br><br>Training consisted of 6 training tasks that incorporated both discrete movements (2 tasks) and rhythmic movements (4 tasks), paced by a metronome<br><br>Initially most tasks completed for 20 repetitions, which was gradually increased to 40 repetitions<br><br>Therapeutic challenge was increased throughout the training period

	3 training sessions of 1 hour duration completed each week for 8 weeks were completed<br><br>Profession of individual administering training not reported
Outcomes	Primary outcome: functional movement - arm functional movement: Motor Assessment Scale (upper arm function and combined upper limb movements; upper arm function scores used for analysis); hand functional movement: Motor Assessment Scale (hand movements and advanced hand movements; hand movement scores used for analysis)<br><br>Secondary outcome: motor impairment - motor impairment scales: Motor Status Score (total scale, shoulder/elbow scale and wrist/hand scale; total scale selected for use in analysis); strength outcomes: muscle strength comparator dynamometer for arm strength and Jamar dynamometer for grip strength (arm strength outcome selected for use in analysis)
Notes	Data presented in paper in graph format - mean and SE for Motor Assessment Scale and Motor Status Score<br><br>Means estimated from graph and standard deviation calculated from estimated standard error to allow for inclusion in statistical pooling<br><br>2 review authors independently estimated the values from the graphs; the average of the 2 estimates was used in the analysis<br><br>Unable to include strength outcome in analysis as separate results for the 2 groups (unilateral and bilateral) not presented<br><br>A non-significant result between the groups reported in the paper on these measures and this indicated in the results section

Risk of Bias[58]

Bias	Author's Judgment	Support for Judgement
Random sequence generation		
Allocation concealment	High risk	Computer-generated list provided group assignment but first author enrolled participants and provided both treatment interventions
Blinding of participants and personnel		
Blinding of outcome assessment	Low risk	Single rater completed outcome assessments blinded to group allocation and study methodology
Incomplete outcome data		
Selective reporting		
Other bias		

Subramanian (2013)

Characteristics[46]	
Methods	RCT
Participants	Study took place in Canada<br><br>32 participants: 16 intervention, 16 control<br><br>Inclusion criteria: between 40 and 80 years, sustained single ischaemic or haemorrhagic stroke 6 to 60 months previously, scored 3 to 6 on the Chedoke McMaster Stroke Assessment arm subscale and had no other neurologic or neuromuscular/orthopaedic problems affecting the upper limb and trunk<br><br>Exclusion criteria: brainstem or cerebellar lesions, comprehension difficulties and marked apraxia, attention or visual field deficits<br><br>Mean (SD) age: intervention group 62 (9.7), control group 60 (11) years<br><br>72% male<br><br>Stroke details: 47% right hemiparesis<br><br>Timing post stroke: intervention group mean (SD) 3.7 (2.2) years, control group 3.0 (1.9) years
Interventions	Virtual reality intervention: a 3D virtual environment (CAREN system) simulated a supermarket scene. Participants had to reach for objects in the virtual environment. Training was high in intensity with 72 trials of reaching in each session<br><br>Control intervention: pointing at targets in a physical environment<br><br>Sessions were 45 minutes for 12 days spaced over 4 weeks
Outcomes	Outcomes were recorded at baseline, post-intervention and 3 months following intervention<br><br>Upper limb outcomes: Fugl Meyer, Reaching Performance Scale for Stroke, Wolf Motor Function Test<br><br>Adverse events reported<br><br>Other outcomes: Motor Activity Log-AS<br><br>Other outcomes: Motivation Task Evaluation Questionnaire<br><br>Other outcomes: kinematic data
Notes	
Risk of Bias[46]	

Bias	Author's Judgment	Support for Judgement
Random sequence generation	Low risk	Computer-generated
Allocation concealment	Low risk	Managed by external personnel
Blinding of participants and personnel		
Blinding of outcome assessment	Low risk	Blinded to allocation
Incomplete outcome data	Low risk	All completed the assessments. Small number of intervention drop outs and balanced across groups
Selective reporting	Low risk	All outcomes reported as per entry on clinical trial registry
Other bias		

Sullivan (2007)

Characteristics[18]	
Methods	RCT, parallel-group design<br><br>Method of randomisation: stratified block randomisation (block size not stated)<br>Blinding of outcome assessors: yes<br><br>Adverse events: 21 cumulative adverse events in 18 participants until follow-up<br><br>Deaths: none<br><br>Dropouts: 9 until follow-up (6 in EXP group, 3 in CTL group)<br><br>ITT: yes, last observation carried forward for primary outcomes
Participants	Country: USA<br><br>80 participants (60 in EXP group, 20 in CTL group)<br><br>Ambulatory at study onset: yes<br><br>Mean age: 63 and 60 years (CTL and EXP group, respectively)<br><br>Inclusion criteria: aged 18 and above, ischaemic or haemorrhagic stroke confirmed by CT, MRI or clinical criteria, 4 to 60 months post-stroke, ambulate at least 10 metres with assistive or orthotic device, FAC 2 or above, walking speed < 1 m/s, informed consent, approval of primary care physician

	Exclusion criteria: serious medical conditions interfering with the study protocol such as high blood pressure, high resting heart rate, lower limb orthopaedic conditions, recent botulinum toxin injections, recent baclofen delivery, MMSE score < 24, co-interventions aiming at gait-training or lower extremity strengthening, prior enrolment to similar studies, plans to move out of the area of study centres during the next year
Interventions	4 arms:<br>1. CTL group received combined resistive leg cycling and upper-extremity ergometry, 4 times per week for 6 weeks (4 hours per week)<br>2. EXP group 1 received combined body weight-supported treadmill training and upper extremity ergometry for the same time and frequency<br>3. EXP group 2 received combined body weight-supported treadmill training and resistive leg cycling for the same time and frequency<br>4. EXP group 3 received combined body weight-supported treadmill training and lower extremity progressive-resistive exercise for the same time and frequency
Outcomes	Primary outcome was recorded at baseline, after 12 and 24 treatment sessions, and at 6-month follow-up<br><br>Secondary outcomes were recorded at baseline, at the end of the intervention phase, and at 6-month follow-up<br><br>Primary outcome: overground self-selected walking speed<br><br>Secondary outcomes: fast walking speed, 6-Minute Walk Test, lower extremity FMA, Berg Balance Scale, 16-item Stroke Impact Scale (SIS-16), Medical Outcomes Study Short Form Health Survey (SF-36), lower extremity isometric peak torque
Notes	The 3 experimental groups (using body weight-supported treadmill training) were collapsed together and compared with the CTL group

Risk of Bias[18]

**Bias**	**Author's Judgment**	**Support for Judgement**
Random sequence generation	Low risk	Random sequence was generated at a central data management centre
Allocation concealment	Low risk	Allocation was performed by a central data management centre
Blinding of participants and personnel		
Blinding of outcome assessment	Low risk	Outcome assessors were blinded
Incomplete outcome data		
Selective reporting		
Other bias		

Sundseth (2012)

Characteristics[38]

Methods	Prospective RCT with blinded assessment at follow-up
Participants	1 stroke unit in Akershus, Norway<br><br>Acute stroke patients (infarct or haemorrhage), defined according to the WHO criteria, admitted to the stroke unit within 24 hours of stroke<br><br>Exclusion criteria included; age < 18 years, prior dependency (mRS score 1 to 5), secondary haemorrhage, acute coronary disease, treated with thrombolysis or thrombectomy<br><br>65 participants recruited (12 lost to follow-up): 32 intervention (7 lost after randomization), 33 control (5 lost after randomization)<br><br>Mean age: 77 years<br>25/56 (45%) men<br><br>5/56 (18%) haemorrhagic<br>Mean NIHSS 8<br><br>37 (66%) had mild stroke (NIHSS 1 to 7)<br><br>11 (20%) moderate stroke (NIHSS 8 to 16)<br><br>8 (14%) severe stroke (NIHSS > 16)<br><br>Significantly lower prevalence of diabetes in the intervention group: 2/27 (7%) vs 8/29 (28%); P = 0.05
Interventions	**What**<br><br>Materials: both groups received standard stroke unit care. No detailed mobilisation protocol was used.<br><br>Procedures: VEM participants were mobilised out of bed as soon as possible after randomization and at least 24 hours from admission to hospital. Mobilisation, meaning all out-of-bed activities, was carried out several times per day. Control participants started mobilisation 24 to 48 hours from admission<br><br>**Who provided**<br><br>VEM was performed by physiotherapists, nursing staff, and occupational therapists<br><br>**How**<br><br>VEM participants were mobilised out of bed as soon as possible after randomization and at least 24 hours from admission to hospital by physiotherapy, nursing, and occupational therapy staff<br><br>**Where**<br><br>In the stroke unit

	**When and how much**<br><br>Until discharge from the stroke unit<br><br>**Tailoring**<br><br>All mobilisation was adjusted to the participant's needs and abilities. A neurologist could be called to postpone mobilisation in participants with deteriorating condition while exercising<br><br>**Modifications**<br><br>The intervention protocol was not changed during the trial. The type and amount of mobilisation was the same for infarct and haemorrhage<br><br>**How well**<br><br>Planned: the type and amount of mobilisation was similar to the VEM group but neither time nor duration was recorded<br><br>Actual: 5/32 VEM participants were not mobilised to protocol (3 were mobilised within 48 hours and 2 within 72 hours)<br><br>1/33 control participant had very delayed mobilisation at 85 hours. Median time from stroke to first mobilisation in the VEM group was 13.1 hours (IQR 8.5 to 25.6) vs 33.3 hours (IQR 26.0 to 39.0) in controls (P = 0.001). Neither frequency nor duration of mobilisation was recorded
Outcomes	Outcomes were recorded at discharge and 3 months and included:<br>• good outcome (mRS of 0 to 2);<br>• survival;<br>• ADL score (Barthel index);<br>• Complications were classified as:<br>o stroke-related (recurrent stroke or intracerebral haemorrhage, transient ischaemic attack, post-apoplectic epilepsy)<br>o immobility-related (deep vein thrombosis, pulmonary embolism, bedsores, pneumonia, urinary tract infection, and fall(s); and comorbidity-related (angina pectoris, myocardial infarction)<br>o Hospital Anxiety and Depression Scale (HADS)
Notes	Study recruited during 2007 and 2009 to 2010. Additional activity was not specified, but the intervention group participants appeared to have received more out-of-bed activity in total, because it commenced earlier. Study was powered to include 246 participants, but stopped early because of slow recruitment<br><br>9 participants were excluded after randomization when the diagnosis of stroke could not be confirmed

***Risk of Bias*[38]**

Bias	Author's Judgment	Support for Judgement

Random sequence generation	Low risk	Quote: "computer-generated, blocked, randomization procedures"
Allocation concealment	Low risk	Quote: "using opaque envelopes"
Blinding of participants and personnel	Unclear risk	No specific comment
Blinding of outcome assessment	Low risk	Quote: "blinded assessment at the end of follow-up". Blinding was not present for earlier outcomes
Incomplete outcome data	Unclear risk	A total of 12/65 (18%) patients randomized did not undergo 3-month follow-up
Selective reporting	Unclear risk	Primary outcome largely complete
Other bias		

Tavazzi (2008)

Characteristics[55]	
Methods	RCT; parallel; placebo-controlled<br><br>Number of study sites: 357<br><br>Dates: randomisation between 6 August 2002 and 28 February 2005. Follow-up concluded on 31 March 2008
Participants	Inclusion criteria: men and women aged 18 years or older, with clinical evidence of heart failure of any cause that was classified according to the European Society of Cardiology guidelines as New York Heart Association (NYHA) class II–IV, provided that they had had their LVEF measured within 3 months before enrolment. When LVEF was greater than 40%, the patient had to have been admitted at least once to hospital for heart failure in the preceding year to meet the inclusion criteria<br><br>Exclusion criteria: specific indication or contraindication to n-3 PUFA; known hypersensitivity to study treatments; presence of any non-cardiac comorbidity (e.g. cancer) that was unlikely to be compatible with a sufficiently long follow-up; treatment with any investigational agent within 1 month before randomisation; acute coronary syndrome or revascularisation procedure within the preceding 1 month; planned cardiac surgery, expected to be done within 3 months after randomisation; significant liver disease; and pregnant or lactating women or women of childbearing potential who were not adequately protected against becoming pregnant; conditions that in the opinion of the investigator would be associated with poor adherence to the

	protocol; background therapy including: (1) for randomisation to n-3 PUFA, an ongoing post-MI treatment with n-3 PUFA; (2) for randomisation to rosuvastatin, lipid-lowering therapy with statins<br><br>Sample size: 6975 participants (total population), 346 participants with history of stroke<br><br>Participants randomised to intervention group (only stroke population): 168<br><br>Participants randomised to control group (only stroke population): 178<br><br>Sex (only stroke population): marine-derived n-3 PUFAs group = 130 men and 38 women; placebo = 145 men and 33 women<br><br>Age, years (only stroke population) mean ± SD: marine-derived n-3 PUFAs group = 71 ± 8; placebo = 70 ± 9<br><br>Time from event (stroke) to start of intervention: not stated<br><br>Type of baseline stroke: not stated<br><br>Country: Italy<br><br>Ethnicity: not stated
Interventions	Comparison groups: marine-derived n-3 PUFAs versus placebo. Participants without specific indications or contraindications to statins were further randomised to rosuvastatin or corresponding placebo<br><br>Intervention: 1 capsule per day of 1 g marine-derived n-3 PUFAs (850 to 882 mg EPA and DHA as ethyl esters in the average ratio of 1:1.2)<br><br>Control: matching placebo (no further details)<br><br>Compliance: a participant was regarded as compliant to the treatment if the study drug was administered for at least 80% of the days of observation<br><br>Duration of intervention: median of 3.9 years (IQR 3.0 to 4.5)<br><br>Co-interventions: participants also randomised to rosuvastatin (10 mg/day) or corresponding placebo
Outcomes	Primary outcome of study: time to death, and time to death or admission to hospital for cardiovascular reasons<br><br>Relevant review outcomes measured (in total population): vascular-related death; recurrent events; adverse events<br><br>Available outcome data for stroke population only: vascular-related death; recurrent events (stroke)<br><br>Latest time point of assessment: median of 3.9 years (IQR 3.0 to 4.5)<br><br>Withdrawals or exclusions, n (stroke population only): not available

	Study authors' contact status: replied, data provided
Notes	Dietary information: treatment with n-3 PUFA was part of exclusion criteria. Further data not provided for overall study; however, in a sub-study (51 centres) authors report the following (quote): "fish consumption was distributed between never consumers (25.6%), those consuming fish about once per week (36.3%), and those consuming fish 2 or more times per week (38.1%). Baseline levels of n-3 PUFA were positively associated with the frequency of fish consumption [...] There was no specific information on the consumption of types of fish such as oily fish, which have highest levels of EPA and DHA"<br><br>Funding: pool of financial grants by the companies which are the owners and the suppliers of the study drugs (Pharmacia-Upjohn, Sigma-Tau, and Societa' Prodotti Antibiotici supply capsules containing 850 to 882 mg EPA/DHA ethyl esters; rosuvastatin is supplied by Astra-Zeneca)

Risk of Bias[55]

Bias	Author's Judgment	Support for Judgement
Random sequence generation	Low risk	Quote: "Computerised telephone randomization system"
Allocation concealment	Low risk	Quote: "Allocation of patients to treatment groups will be accomplished via a telephone call-in system, and centrally approved at the study Coordinating Centre"
Blinding of participants and personnel	Low risk	Quote: "All patients and study personnel were blinded to treatment"
Blinding of outcome assessment	Low risk	Quote: "All the events recorded in the study were adjudicated blindly by an ad-hoc committee on the basis of pre-agreed definitions and procedures"
Incomplete outcome data	Low risk	Participants discontinuing study treatment balanced across intervention and control groups
Selective reporting	Low risk	Rationale published before randomisation period finished, All pre-specified outcomes were reported

Other bias	Low risk	None detected

Thieme (2013)

Characteristics[26]	
Methods	RCT
Participants	Country: Germany<br><br>Setting: inpatient rehabilitation centre<br><br>Age: adults (mean age: 67.2 years)<br><br>Sample size: 60 participants (21 in the mirror therapy group intervention, 18 in the mirror therapy single therapy, 21 in the sham group; 11 dropped out in the intervention period)<br><br>Sex: 25 women, 35 men<br><br>Inclusion criteria: 1st supratentorial stroke within the previous 3 months; aged between 18 and 80 years; clinically diagnosed severe hemiparesis or hemiplegia of the distal upper limb with MRC grading of 0 or 1 of wrist and finger extensors<br><br>Exclusion criteria: visual impairments that may limit participation in mirror therapy; severe cognitive and/or language deficits which preclude participants from following instructions in the group training protocol; other neurological or musculoskeletal impairments of the upper extremity not due to stroke; severe neglect (head is not turned to the affected side due to instruction)
Interventions	3 arms<br><br>1, 2 and 3: standard rehabilitation programme, additional:<br><br>1. MT group intervention: participants perform movements with both arms (the affected arm as best as could be) while watching the mirror image of the unaffected arm, participants exercised in open groups of 2 to 6 participants<br>2. MT single therapy: see group 1, participants exercised in one-to-one therapy<br>3. Sham therapy: group intervention; participants exercise in open groups of 2 to 6 participants with the non-reflecting side of the mirror positioned to the unaffected arm<br><br>1, 2 and 3: 5 weeks, additional 20 sessions, 30 minutes MT, MT group or sham therapy<br><br>Date of intervention: April 2009 to July 2011
Outcomes	Outcomes assessed before and after treatment, and 7 months after treatment

1. FMA-UE (0 to 66)
2. FMA sensory assessment, range of motion and pain arm
3. ARAT (0 to 57)
4. MAS (0 to 5) wrist and finger flexors, elbow flexors
5. BI (0 to 100)
6. SIS
7. SCT

Notes	Published and unpublished data<br><br>Funding source: Klinik Bavaria Kreischa, Germany<br><br>Declarations of trialists' interests: the first author received and will receive honorarium for presentations and seminars on MT; the other authors declared no potential conflicts of interest

Risk of Bias[26]

Bias	Author's Judgment	Support for Judgement
Random sequence generation	Low risk	Participants were randomly assigned by computer-generated random sequence
Allocation concealment	Low risk	Concealed allocation by an independent person
Blinding of participants and personnel		
Blinding of outcome assessment	Low risk	Assessors of primary outcome (motor function) were blinded to group allocation
Incomplete outcome data	Low risk	ITT analysis was performed ('last observation carried forward' method)
Selective reporting		
Other bias		

Todd (2005)

Characteristics[59]

Methods	RCT<br><br>Multicentre study from centres in the USA, UK, New Zealand, Austria, Australia, Canada and Germany
Participants	1001 participants with a preoperative WFNS score of I, II, or III (good grade patients), who had SAH no more than 14 days before planned surgical

	aneurysm clipping: 499 participants were allocated to the hypothermia group, 501 participants were allocated to the normothermia group, and 1 participant was lost to follow-up<br><br>Inclusion criteria: at least 18 years of age, not pregnant, had suffered a subarachnoid haemorrhage from a radiologically demonstrated intracranial aneurysm within 14 days before surgery, had a WFNS score of I, II, or III ("good grade") at the time of enrolment, which was verified on arrival in the operating room. Participants were required to have had a Rankin<br><br>score of 0 (no neurologic disability) or 1 (mild disability) before their haemorrhage<br><br>Exclusion criteria: body-mass index of more than 35, had a cold-related disorder, had an endotracheal tube in place
Interventions	Intervention: participants were assigned to hypothermia, oesophageal temperature was reduced as quickly as possible, without any delay in the progress of surgery. The goal was to achieve a temperature between 32.5 and 33.5° C by the time the first clip was applied<br><br>Control: the temperature of participants assigned to normothermia was kept between 36 and 37°C
Outcomes	Patients' neurological outcome were assessed 90 days after surgery by means of the GOS, the Rankin scale, the Barthel index, the NIHSS, and a battery of neuropsychological examinations. If a participant could not be seen by examiners at the operating centre, arrangements were made for that participant to be seen elsewhere by a certified examiner
Notes	Study was supported by a grant from the National Institute of Neurological Disease and Stroke (RO1 NS38554)

Risk of Bias[59]

**Bias**	**Author's Judgment**	**Support for Judgement**
Random sequence generation	Low risk	A permuted-block scheme was used for randomisation, with stratification according to the centre and the time between SAH and surgery (0 to 7 days or 8 to 14 days)
Allocation concealment	Low risk	Less than 2 hours before the planned start of surgery, patients were evaluated and enrolled by means of a telephone-accessed computer system, which directed the anaesthesiologist to use a numbered opaque envelope containing the participant's treatment assignment. The envelope was to be opened only after the induction of anaesthesia

Blinding of participants and personnel	Low risk	All study personnel, except the anaesthesiologists involved in intraoperative care, were unaware of the participants' treatment assignments
Blinding of outcome assessment	Low risk	All study personnel, except the anaesthesiologists involved in intraoperative care, were unaware of the participants' treatment assignments
Incomplete outcome data	Low risk	Quote "... resulting in a total enrolment of 1001 patients. One patient was lost to follow-up, leaving 1000 patients in our efficacy population."<br><br>Comment: 1 participant lost to follow-up out of 1001 participants had little influence on the effect assessment
Selective reporting	Low risk	Death and neurological outcomes were measured by the GOS, the Rankin scale, the Barthel index, the NIHSS, and the occurrence of possible side effects of this treatment were reported
Other bias	Low risk	No other bias was found

Tomić (2017)

Characteristics[6]	
Methods	RCT
Participants	Country: Serbia<br><br>Sample size: 26 participants (13 in treatment group; 13 in control group)<br><br>Inclusion criteria: unilateral paresis as a result of first ischaemic or haemorrhagic stroke confirmed by CT or MRI that occurred less than 3 months before enrolment, the ability to understand and follow simple instructions, the ability to perform some active movements in the shoulder or elbow joints, or both, in the sitting position, allowing for trunk compensation if needed

	Exclusion criteria: multiple strokes, bilateral impairment, severe sensory deficits in the paretic upper limb, the inability to provide informed consent, and medical conditions that could interfere with treatment (severe cardiovascular disease, severe visual or auditory impairments, and orthopaedic contracture)
Interventions	2 groups:<br>1. additional robot therapy with the ArmAssist (AA) for 30 minutes administered over 15 sessions each lasting 30 minutes, scheduled 5 days per week (Monday to Friday) for 3 weeks,<br>2. additional occupational therapy for 30 minutes that was matched in its structure and amount to the AA training as close as possible and administered over 15 sessions each lasting 30 minutes, scheduled 5 days per week (Monday to Friday) for 3 weeks
Outcomes	Outcomes were measured at baseline (week 0), and at the end of intervention period (week 3)<br><br>Primary outcome:<br>1. total FMA score<br><br>Secondary outcome:<br>1. WMFT<br>2. Barthel Index
Notes	

Risk of Bias[6]

Bias	Author's Judgment	Support for Judgement
Random sequence generation	Low risk	Table of random numbers
Allocation concealment	Unclear risk	Insufficient information to permit judgement of 'low' or 'high' risk
Blinding of participants and personnel		
Blinding of outcome assessment	Low risk	Blinded physiotherapist
Incomplete outcome data		
Selective reporting	Low risk	No missing outcome data
Other bias		

Tyson (2015)

Characteristics[26]

Methods	RCT
Participants	Country: UK<br><br>Setting: 12 inpatient stroke services

	Age: adults (mean age: 64 years) | Sample size: 94 participants from 12 sites: 63 in experimental group (6 dropped out), 31 in control group (3 dropped out)<br><br>Sex: 34 women, 60 men<br><br>Inclusion criteria: stroke at least 1 week previously and inpatient in a stroke rehabilitation unit, no premorbid conditions limiting upper or lower limb function, sufficient cognitive and communication to give consent, medically stable and able to participate in rehabilitation, upper or lower limb weakness which limits activity<br><br>Exclusion criteria: not stated
Interventions	2 arms<br><br>1 and 2: conventional rehabilitation programme<br><br>1. Participant–led MT: participants were taught how to do the mirror therapy and given an (aphasia-friendly) instruction booklet to show them how to position the mirror themselves and also the exercises to do. An allocated member of staff checked on them daily to remind them to do the therapy and complete their diary sheets, help them get set up (if necessary), deal with any problems and progress the exercises<br>2. Attentional control: lower limb exercises (without a mirror)<br><br>1 and 2: 4 weeks, 7 days a week, 30 minutes a day MT or lower-limb exercises<br><br>Date of intervention: not stated
Outcomes	Outcomes were recorded at baseline, after 4 weeks of therapy, and 8 weeks after baseline<br><br>1. Feasibility and acceptability of patient-led mirror therapy from a patient and staff perspective (assessed by questionnaire and interviews/ focus groups)<br>2. Recruitment and retention rate<br>3. Adherence to the therapy<br>4. Adverse events<br>5. SCT<br>6. MI-UL<br>7. BBT<br>8. ARAT<br>9. RASP<br>10. MAS elbow<br>11. Adverse events - participant self-report<br>12. Adherence – practice log sheets completed by the participant and treating clinician
Notes	Published and unpublished data, full-text publication received in 2016

	Funding source: National Institute for Health Research under its Research for Patient Benefit (RfPB) Programme<br><br>Declarations of trialists' interests: there are no conflicts of interest

Risk of Bias[26]

Bias	Author's Judgment	Support for Judgement
Random sequence generation	Low risk	Participants were randomly assigned by computer-generated random-number sequence
Allocation concealment	Low risk	Allocation by an independent web-based randomisation service
Blinding of participants and personnel		
Blinding of outcome assessment	Low risk	Assessor was blinded to group allocation
Incomplete outcome data	High risk	No ITT analysis
Selective reporting		
Other bias		

Vanoglio (2017)

Characteristics[6]

Methods	RCT
Participants	Country: Italy<br>Sample size: 30 participants (15 in treatment group; 15 in control group)<br>Inclusion criteria: age > 18 years, patients affected by stroke from cerebral ischaemia or haemorrhage that had occurred ≤ 30 days before, with Ashworth spasticity index < 3<br><br>Exclusion criteria: orthopaedic limitation (amputations, irreducible articular limitations, advanced osteoarthritis, active rheumatoid arthritis); peripheral nerve injury; uncontrolled inflammation; severe cognitive and behavioural disorders; neurodegenerative and neuromuscular diseases; Ashworth Spasticity index ≥ 3
Interventions	2 groups:<br><br>1. robot therapy with the Gloreha Professional (Idrogenet, Lumezzane, Italy) consisted of a total of 30 sessions, lasting 40 minutes per day, for 5 days per week<br>2. passive arm therapy for the same amount of therapy

Outcomes	Outcomes were measured at baseline (week 0), and at the end of intervention period (after 30 days)<br><br>Outcomes:<br>1. Motricity Index<br>2. Nine Hole Peg Test<br>3. Grip and Pinch test<br>4. Quick DASH
Notes	

Risk of Bias[6]

Bias	Author's Judgment	Support for Judgement
Random sequence generation	Low risk	Computerised random numbers
Allocation concealment	Low risk	Randomisation procedure was conducted independently from the study investigators, and authors used central randomisation
Blinding of participants and personnel		
Blinding of outcome assessment	Low risk	Blinding of outcome assessors was done
Incomplete outcome data		
Selective reporting	Unclear risk	Insufficient reporting of attrition/exclusions to permit judgement of 'low' or 'high' risk
Other bias		

Varley (2016)

Characteristics[12]

Methods	RCT (cross-over), UK
Participants	50 participants randomised a | and not receiving impairment SLT<br><br>Inclusion criteria: unilateral left-hemisphere lesion, adults, at least 6 months poststroke, apraxia of speech<br>Exclusion criteria: not premorbidly competent in English, insufficient auditory and visual acuity to interact with laptop, currently receiving impairment-based SLT or presence of degenerative neurocognitive impairment<br><br>Group 1: 25 participants reported<br><br>Group 2: 25 participants reported<br><br>Details of participants are shown in Table 1

Interventions	**1. Self administered computer programme therapy targeting whole word production and error reduction strategies ("Speech-first")**<br><br>**Intervention**: computer SLT . Errorless learning, therapy delivered at level of sufficient level of difficulty and intensity to facilitate neuronal reorganisation. **Materials**: computer-based programme. **Procedures**: participant practiced automatic, fluent, errorless speech production. Aim for non-fluent speech attempts and struggle and groping reduced. Self administered for 6 weeks. Computer booted up at point where participant had previously left off. **Provided by**: self administered but with access to support if required. **Delivery**: computer-facilitated, 1 to computer, at home. **Regimen**: average of 3.3 h/week delivered over 6 weeks. Total dose = approx 20 h of self administered therapy. **Tailoring**: automatic tailoring of level of difficulty within computer programme. **Modification**: none other than the automated process. **Adherence**: computer programme recorded activity, mean 1187 (SE 135.2; range 254-3029) min<br><br>**2. Visuo-spatial sham computer programme ("Sham-first")**<br><br>**Intervention**: no SLT. Sham programme, minimal speech/language content, visuo-spatial problem solving. **Materials**: delayed matching of complex designs. **Procedures**: automatically booted up to where participants had left off at previous session. **Provided by**: self administered but with access to support if required. Delivery: computer-facilitated, 1 to computer, at home. **Regimen**: self administered over 6 weeks. Total dose = up to approx 18 h of self administered therapy. **Tailoring**: automatic tailoring of level of difficulty within computer programme. **Modification**: none other than the automated process. **Adherence**: computer programme recorded activity, mean 1058 (SE 154.22; range137-3129) min
Outcomes	Primary outcomes: word Repetition<br><br>Secondary outcomes: Comprehensive Aphasia Test (subtest Comprehension of Spoken Sentences); PALPA (written word to picture matching subtest); Picture naming test<br><br>Data collection: baseline (x 2); post-therapy 1; post-therapy 2; follow-up 8 weeks post-therapy (time point 3)
Notes	Dropouts are detailed in Table 2

Risk of Bias[12]

Bias	Author's Judgment	Support for Judgement
Random sequence generation	Low risk	Web-based randomisation system, block randomised
Allocation concealment	Low risk	Adequate (blind envelope system by investigator blind to case)

Blinding of participants and personnel	Low risk	Adequate
Blinding of outcome assessment	Low risk	Adequate
Incomplete outcome data	Low risk	Dropouts accounted for<br><br>ITT analysis employed
Selective reporting	Low risk	All prespecified outcomes reported
Other bias	Unclear risk	Groups were comparable at baseline in relation to measures of aphasia and apraxia severity (Spoken Picture Naming, Spoken Reversible Sentence Comprehension, Auditory Lexical Decision, Auditory Minimal Pairs, non-word repetition accuracy; Repetition of words of increasing syllable length; non-speech oro motor tasks, mean phonation time in seconds, DDK rates), mean time postonset 22 months (range 5-105) months. There was a sex imbalance in the 'speech-first' condition, with more men than women.<br><br>Power calculation a priori

Villafañe (2017)

Characteristics[6]	
Methods	RCT
Participants	Country: Italy<br><br>Sample size: 32 participants (16 in treatment group; 16 in control group)<br><br>Inclusion criteria: history of acute phase of stroke, first stroke episode, no history of peripheral nerve injury or musculoskeletal disease (e.g. arthritis, musculotendinous injury, or bone fracture) in the affected upper extremity, no contracture of the affected wrist or fingers (MAS < 3),and no history of any invasive procedure (botulinum toxin type A) for the treatment of spasticity for at least 6 months prior to the start of this study, and paralysis of the wrist and fingers and absence in voluntarily initiating and controlling finger extension movements

	Exclusion criteria: unstable medical disorders, active complex regional pain syndrome, severe spatial neglect, aphasia, or cognitive problems, > 4 points on the Beck Depression Inventory or more than 30 points in the State Trait Anxiety Inventory
Interventions	2 groups:<br><br>1. robot therapy with the hand Gloreha for 30 minutes for 3 days per week<br>2. physical and occupational arm therapy for the same amount and intensity
Outcomes	Outcomes were measured at baseline (week 0), and at the end of intervention period (week 3)<br><br>Outcomes:<br><br>1. NIH Stroke Scale<br>2. MAS<br>3. Barthel Index<br>4. Motricity Index<br>5. QuickDASH (short version of the Disabilities of the Arm, Shoulder and Hand)<br>6. visual analogue scale
Notes	

Risk of Bias[6]

**Bias**	**Author's Judgment**	**Support for Judgement**
Random sequence generation	Unclear risk	Method of randomisation unclear because described as "simple randomization"
Allocation concealment	Unclear risk	Insufficient information to permit judgement of 'low' or 'high' risk
Blinding of participants and personnel		
Blinding of outcome assessment	Low risk	Blinded outcome assessors
Incomplete outcome data		
Selective reporting	Unclear risk	Insufficient information to permit judgement of 'low' or 'high' risk
Other bias		

Weitz (2010)

Characteristics[20]

Methods	Randomised, partially-blinded, active controlled trial
Participants	1146 people aged between 18 and 65 years with documented non-valvular AF and a CHADS2 score of at least 2
Interventions	Edoxaban (30 mg once daily, 30 mg twice daily, 60 mg once daily, or 60 mg twice daily) versus dose-adjusted warfarin (target INR 2.0 to 3.0) during a predefined 12-week period
Outcomes	Primary safety outcome: major bleeding (defined by modified ISTH criteria)<br><br>Secondary safety outcomes: clinically relevant non-major bleeding; minor bleeding; liver function tests<br><br>Secondary efficacy outcomes: composite of stroke (ischaemic or haemorrhagic), systemic embolic event, myocardial infarction, cardiovascular death and hospitalisation for any cardiac conditions
Notes	Study sponsored by Daiichi Sankyo

Risk of Bias[20]

Bias	Author's Judgment	Support for Judgement
Random sequence generation	Low risk	Participants were randomly assigned to treatment groups
Allocation concealment	Low risk	Participants were randomly assigned to treatment groups
Blinding of participants and personnel	High risk	Open-label administration of both edoxaban and warfarin. Different doses of edoxaban administered in double-blind fashion
Blinding of outcome assessment	Unclear risk	Adjudication of bleeding events by independent central adjudication committee that was blinded to treatment assignment. Unclear whether efficacy outcomes were centrally adjudicated
Incomplete outcome data	Unclear risk	Safety and efficacy outcomes analysed in 'safety population' (participants who received at least 1 dose of study drug and had at least 1 post-dose safety assessment). Number of participants that discontinued

		during study and reasons for discontinuation not stated
Selective reporting	Low risk	All predefined safety and efficacy outcomes reported for safety population
Other bias	Unclear risk	Randomisation into edoxaban 60 mg twice daily treatment arm prematurely terminated after enrolment of 180 patients based on recommendation of independent DSMB due to an excess of bleedings

Wolf (2006)

Characteristics[60]	
Methods	Randomisation automated, balanced with respect to sex, premorbid handedness, side of stroke and level of function<br><br>Blinded outcome assessor<br><br>Post-treatment withdrawals: 8%; follow-up withdrawals: 17%<br><br>Multicentric, outpatients
Participants	USA<br>222 participants<br><br>Intervention: N = 106, mean age 61 ± 13.5 years, 34.9% women<br>Control: N = 116, mean age 63.43 ± 12.6 years, 37.1% women<br><br>Mean time since stroke: intervention: 179.8 ± 66.1 days, control: 187.7 ± 70.8 days Stroke details: ischaemic or haemorrhagic; intervention: 47.2% paresis of dominant side, control: 51.75% paresis of the dominant side<br><br>Focal inclusion criteria: cerebrovascular accident between 3 and 9 months; 10° of active extension to the metacarpophalangeal and interphalangeal joints and 20° at wrist or 10° of active extension to the metacarpophalangeal and interphalangeal joints of two digits, and at wrist, 10° of thumb abduction/extension
Interventions	CIMT versus usual care<br>CIMT: adaptive task practice (shaping) and standard task training of the paretic limb<br><br>Amount of restraint: 90% of waking hours<br>Usual care: usual and customary care ranged from no treatment to the application of mechanical interventions or various occupational and physical therapy approaches in the home<br>Session duration: intervention: 6 hours per day, 7 days per week, 2 weeks

	Anatomical region restraint: hand
Outcomes	Measures pre/post treatment and follow up at 4, 8, and 12 months<br>• Motor function: WMFT<br>• Perceived arm motor function: MAL<br>• Quality of life: SIS
Notes	

Risk of Bias[60]

Bias	Author's Judgment	Support for Judgement
Random sequence generation	Low risk	Sequence generation by random automated generator
Allocation concealment	Low risk	Centralised
Blinding of participants and personnel	Low risk	Blinding of outcome assessor
Blinding of outcome assessment	Low risk	Blinding of outcome assessor
Incomplete outcome data		
Selective reporting		
Other bias		

Wolf (2015)

Characteristics[6]

Methods	RCT
Participants	Country: USA<br><br>Sample size: 99 participants (51 in treatment group; 48 in control group)<br><br>Inclusion criteria: unilateral ischaemic or haemorrhagic stroke within the previous 6 months confirmed by neuroimaging; persistent hemiparesis with some upper limb voluntary movement, as indicated by a score of 11 to 55 on the FMA; ineligibility to receive any further upper-extremity therapy; and preserved cognitive function (Short Portable Mental Status Questionnaire)<br><br>Exclusion criteria: inability to provide informed consent; not independent before the stroke (determined by score > 1 on the Modified Rankin Scale); hemispatial neglect as determined by > 3 errors on the Star Cancellation Test; sensory loss ≥ 2 on the sensory item of the NIH Stroke Scale; hypertonic affected arm as indicated by a score ≥ 3 on the MAS; antispasticity injection in hemiparetic arm since onset of the stroke; presence of upper-extremity pain or uncorrected vision problems; unmanaged psychiatric issues; and terminally ill with an anticipated survival of less than 1 year
Interventions	2 groups:<br><br>1. robot therapy with the Hand Mentor Pro (Kinetic Muscles Incs) for 60 minutes over a 8 (to 12) weeks period

	2. home exercises for the arm therapy for 60 minutes over a 8 (to 12) weeks period
Outcomes	Outcomes were measured at baseline (week 0), and at the end of intervention period (week 8 to 12)<br><br>Primary outcome:<br><br>1. Action Research Arm Test<br><br>Secondary outcomes:<br><br>1. WMFT<br>2. FMA - Arm
Notes	Formerly ongoing study Linder 2013 (NCT01144715)

Risk of Bias[6]

Bias	Author's Judgment	Support for Judgement
Random sequence generation	Low risk	Computer-driven randomisation procedure
Allocation concealment	Unclear risk	Insufficient information to permit judgement of 'low' or 'high' risk
Blinding of participants and personnel		
Blinding of outcome assessment	Low risk	Blinding of outcome assessors was done
Incomplete outcome data		
Selective reporting	Unclear risk	Insufficient information to permit judgement of 'low' or 'high' risk
Other bias		

Wu (2012)

Characteristics[6]

Methods	RCT<br><br>Method of randomisation: not described
Participants	Country: Taiwan<br><br>Sample size: 42

	Inclusion criteria: unilateral stroke at least 6 months previously, mild to moderate motor impairment (total score of 26 to 66 on the upper extremity part of the FMA, no severe spasticity in the paretic arm (MAS score of 2 in any joint), no serious cognitive deficits (MMSE score of 22), no other neurologic, neuromuscular, or orthopaedic disease and no participation within the previous 3 months in any experimental rehabilitation or drug studies<br><br>Exclusion criteria: none described
Interventions	3 groups:<br><br>1. therapist-mediated bilateral arm training group<br>2. robot-assisted (Bi-Manu-Track) arm trainer (RAT Group)<br>3. CT involved weight bearing, stretching, strengthening of the paretic arms, coordination, unilateral and bilateral fine-motor tasks, balance, and compensatory practice on functional tasks<br><br>Each group received treatment for 90 to 105 minutes per session, 5 sessions on weekdays, for 4 weeks
Outcomes	Outcomes were recorded at baseline and post-treatment after 4 weeks<br><br>1. Kinematic analysis<br>2. FMA<br>3. MAL (quality of use and amount of use)<br>4. SIS
Notes	We combined the results of both the first and the third groups (the non-robot groups) in 1 (collapsed) group and compared this collapsed group with the results of the RAT group

Risk of Bias[6]

**Bias**	**Author's Judgment**	**Support for Judgement**
Random sequence generation	Unclear risk	Exact method not described
Allocation concealment	Low risk	Quote: "The allocation to group was concealed from the investigators"
Blinding of participants and personnel		
Blinding of outcome assessment	Low risk	Quote: "The participants were blinded to the study hypotheses." and "Clinical outcome measures were administered ... by ... therapists blinded to the participant group."

Incomplete outcome data		
Selective reporting	Unclear risk	Insufficient information to permit judgement
Other bias		

Zedlitz (2012)

Characteristics[61]	
Methods	Parallel randomised trial<br>Method of randomisation: block randomisation per treatment centre<br>Method of concealment: interventions used in the 2 groups were visibly different, so participants and medical staff would be aware of what was being used<br>Blinding: 'assessor-blind'<br>Analysis: ITT
Participants	Location: Netherlands<br><br>Setting: community-dwelling, recruited from rehabilitation centres<br><br>Number of participants: 83 participants at randomisation, 73 completed treatment, 68 completed follow-up. ITT analysis for 83 participants (52% male, mean age 55 years)<br><br>Treatment 1 group: 38 (55% male, mean age 55 years)<br><br>Treatment 2 group: 45 (49% male, mean age 55 years)<br><br>Stroke criteria: ischaemic stroke, haemorrhagic stroke or SAH, criteria unclear<br><br>Time since stroke onset at randomisation: > 4 months after stroke, mean 3.9 years<br><br>Fatigue criteria: CIS-fatigue ≥ 40 | Other entry criteria: more than 4 months after stroke, 18 to 70 years old, no cardiopulmonary complications or psychiatric disorders<br><br>Comparability of groups: unclear
Interventions	Treatment 1 intervention (CO): group cognitive therapy (emphasising pacing and relaxation), 2 hours each session, once a week, for 12 weeks<br><br>Treatment 2 intervention (COGRAT): CO plus physical training (walking on treadmill, strength training and homework assignment, 2 hours each session, twice a week, for 12 weeks)<br><br>Treatment duration: 12 weeks<br><br>Delivered by: CO was delivered by neuro-psychologists and GRAT was delivered by physiotherapists

Outcomes	Time for fatigue assessment: baseline, by the end of 12-week treatment, and at 3-month follow-up<br><br>Primary outcome: CIS-f<br><br>Secondary outcomes: self observation list (for fatigue, pain and sleep), Hamilton Anxiety and Depression Scale (for depression and anxiety), Stroke-adapted Sickness Impact Profile-30, 6-minute walk test
Notes	Only people with severe fatigue (CIS-f ≥ 40) and > 4 months after stroke were recruited<br><br>Mainly focused on the change of scores before and after treatment within a group rather than comparing between groups<br><br>Funding: a grant from the Dutch Health Research and Development (ZonMw) (Grant No. 14350053)

Risk of Bias[61]

Bias	Author's Judgment	Support for Judgement
Random sequence generation	High risk	Interventions used in the 2 groups were visibly different, so participants and medical staff would be aware of what was being used
Allocation concealment		
Blinding of participants and personnel	Low risk	Quote: "assessor-blind"
Blinding of outcome assessment		
Incomplete outcome data		
Selective reporting		
Other bias		

References to RCTs Included in Review

Aben (2013)
Aben L, Heijenbrok-Kal MH, Ponds RWHM, et al. Long-Lasting Effects of a New Memory Self-efficacy Training for Stroke Patients: A Randomized Controlled Trial. *https://doi.org/101177/1545968313478487* 2013; 28: 199–206.

Algra (2007)
Algra A. Medium intensity oral anticoagulants versus aspirin after cerebral ischaemia of arterial origin (ESPRIT): a randomised controlled trial. *Lancet Neurology* 2007; 6: 115–124.

Allison (2007)
Allison R, Dennett R. Pilot randomized controlled trial to assess the impact of additional supported standing practice on functional ability post stroke. *Clinical Rehabilitation* 2007; 21: 614–619.

Anderson (2008)
CS A, Y H, JG W, et al. Intensive blood pressure reduction in acute cerebral haemorrhage

trial (INTERACT): a randomised pilot trial. *The Lancet Neurology* 2008; 7: 391–399.

Andrade (2017)
Andrade SM, Ferreira JJ de A, Rufino TS, et al. Effects of different montages of transcranial direct current stimulation on the risk of falls and lower limb function after stroke. *Neurological Research* 2017; 39: 1037–1043.

Ang (2014)
Ang KK, Guan C, Phua KS, et al. Brain-computer interface-based robotic end effector system for wrist and hand rehabilitation: results of a three-armed randomized controlled trial for chronic stroke. *Frontiers in Neuroengineering* 2014; 7: 30.

Barker-Collo (2009)
Barker-Collo SL, Feigin VL, Lawes CMM, et al. Reducing attention deficits after stroke using attention process training: A randomized controlled trial. *Stroke* 2009; 40: 3293–3298.

Barzel (2015)
Barzel A, Ketels G, Stark A, et al. Home-based constraint-induced movement therapy for patients with upper limb dysfunction after stroke (HOMECIMT): A cluster-randomised, controlled trial. *The Lancet Neurology* 2015; 14: 893–902.

Bath (2015)
Bath PMW, Woodhouse L, Scutt P, et al. Efficacy of nitric oxide, with or without continuing antihypertensive treatment, for management of high blood pressure in acute stroke (ENOS): a partial-factorial randomised controlled trial. *The Lancet* 2015; 385: 617–628.

Benavente (2011)
Benavente OR, White CL, Pearce L, et al. The Secondary Prevention of Small Subcortical Strokes (SPS3) Study: *https://doi.org/101111/j1747-4949201000573.x* 2011; 6: 164–175.

Bethoux (2014)
Bethoux F, Rogers HL, Nolan KJ, et al. The Effects of Peroneal Nerve Functional Electrical Stimulation Versus Ankle-Foot Orthosis in Patients With Chronic Stroke: A Randomized Controlled Trial. *http://dx.doi.org/101177/1545968314521007* 2014; 28: 688–697.

Bowen (2012)
Bowen A, Hesketh A, Patchick E, et al. Clinical effectiveness, cost-effectiveness and service users’ perceptions of early, well-resourced communication therapy following a stroke: a randomised controlled trial (the ACT NoW Study). *Health Technology Assessment* 2012; 16: 1–159.

Brokaw (2013)
Brokaw EB, Nichols D, Holley RJ, et al. Robotic Therapy Provides a Stimulus for Upper Limb Motor Recovery After Stroke That Is Complementary to and Distinct From Conventional Therapy: *http://dx.doi.org/101177/1545968313510974* 2013; 28: 367–376.

Brotons (2011)
C B, N S, I M, et al. Randomized clinical trial to assess the efficacy of a comprehensive programme of secondary prevention of cardiovascular disease in general practice: the PREseAP study. *Revista espanola de cardiologia* 2011; 64: 13–20.

Brunner (2012)
Brunner IC, Skouen JS, Strand LI. Is modified constraint-induced movement therapy more effective than bimanual training in improving arm motor function in the subacute phase post stroke? A randomized controlled trial. *Clinical rehabilitation* 2012; 26: 1078–1086.

Burgar (2011)
CG B, PS L, AM S, et al. Robot-assisted upper-limb therapy in acute rehabilitation setting following stroke: Department of Veterans Affairs multisite clinical trial. *Journal of rehabilitation research and development* 2011; 48: 445–458.

Carnaby (2006)
Carnaby G, Hankey GJ, Pizzi J. Behavioural intervention for dysphagia in acute stroke: a randomised controlled trial. *The Lancet Neurology* 2006; 5: 31–37.

Chen (2014)
Chen D-C, Lin S-Z, Fan J-R, et al. Intracerebral Implantation of Autologous Peripheral Blood Stem Cells in Stroke Patients: A Randomized Phase II Study: *http://dx.doi.org/103727/096368914X678562* 2014; 23: 1599–1612.

Chen (2017)
Chen J, Jin W, Dong WS, et al. Effects of Home-based Telesupervising Rehabilitation on Physical Function for Stroke Survivors with Hemiplegia. *American Journal of Physical Medicine & Rehabilitation* 2017; 96: 152–160.

Ciccone (2010)
Ciccone A, Valvassori L, Ponzio M, et al. Intra-arterial or intravenous thrombolysis for acute ischemic stroke? The SYNTHESIS pilot trial. *Journal of NeuroInterventional Surgery* 2010; 2: 74–79.

Combs-Miller (2014)
Combs-Miller SA, Kalpathi Parameswaran A, Colburn D, et al. Body weight-supported treadmill training vs. overground walking training for persons with chronic stroke: A pilot randomized controlled trial. *Clinical Rehabilitation* 2014; 28: 873–884.

Connolly (2013)
Connolly SJ, Eikelboom J, Dorian P, et al. Betrixaban compared with warfarin in patients with atrial fibrillation: results of a phase 2, randomized, dose-ranging study (Explore-Xa). *European Heart Journal* 2013; 34: 1498–1505.

Connolly (2009)
Connolly SJ, Ezekowitz MD, Yusuf S, et al. Dabigatran versus Warfarin in Patients with Atrial Fibrillation. *New England Journal of Medicine* 2009; 361: 1139–1151.

Cowles (2012)
Cowles T, Clark A, Mares K, et al. Observation-to-Imitate Plus Practice Could Add Little to Physical Therapy Benefits Within 31 Days of Stroke: Translational Randomized Controlled Trial. *http://dx.doi.org/101177/1545968312452470* 2012; 27: 173–182.

Cramer (2019)
Cramer SC, Dodakian L, Le V, et al. Efficacy of Home-Based Telerehabilitation vs In-Clinic Therapy for Adults after Stroke: A Randomized Clinical Trial. *JAMA Neurology* 2019; 76: 1079–1087.

da Cunha (2002)
da Cunha IT, Lim PA, Qureshy H, et al. Gait outcomes after acute stroke rehabilitation with supported treadmill ambulation training: A randomized controlled pilot study. *Archives of Physical Medicine and Rehabilitation* 2002; 83: 1258–1265.

Davis (2008)

Davis SM, Donnan GA, Parsons MW, et al. Effects of alteplase beyond 3 h after stroke in the Echoplanar Imaging Thrombolytic Evaluation Trial (EPITHET): a placebo-controlled randomised trial. *The Lancet Neurology* 2008; 7: 299–309.

Dennis (2006a)
Dennis M, Lewis S, Cranswick G, et al. FOOD: a multicentre randomised trial evaluating feeding policies in patients admitted to hospital with a recent stroke. *Health Technology Assessment* 2006; 10: 1–91.

Dennis (2006b)
Dennis M, Lewis S, Cranswick G, et al. FOOD: A multicentre randomized trial evaluating feeding policies in patients admitted to hospital with a recent stroke. *Health Technology Assessment* 2006; 10: 1–91.

Dennis (2009)
Dennis M, Cranswick G, Deary A, et al. Effectiveness of thigh-length graduated compression stockings to reduce the risk of deep vein thrombosis after stroke (CLOTS trial 1): a multicentre, randomised controlled trial. *The Lancet* 2009; 373: 1958–1965.

DePaul (2014)
DePaul VG, Wishart LR, Richardson J, et al. Varied Overground Walking Training Versus Body-Weight-Supported Treadmill Training in Adults Within 1 Year of Stroke: A Randomized Controlled Trial. *http://dx.doi.org/101177/1545968314546135* 2014; 29: 329–340.

Diener (2008)
HC D, RL S, S Y, et al. Effects of aspirin plus extended-release dipyridamole versus clopidogrel and telmisartan on disability and cognitive function after recurrent stroke in patients with ischaemic stroke in the Prevention Regimen for Effectively Avoiding Second Strokes (PRoFE. *The Lancet Neurology* 2008; 7: 875–884.

Dippel (2001)
Dippel DWJ, Van Breda EJ, Van Gemert HMA, et al. Effect of paracetamol (Acetaminophen) on body temperature in acute ischemic stroke: A double-blind, randomized phase II clinical trial. *Stroke* 2001; 32: 1607–1612.

Dohle (2009)
C D, J P, A N, et al. Mirror therapy promotes recovery from severe hemiparesis: a randomized controlled trial. *Neurorehabilitation and neural repair* 2009; 23: 209–217.

Dormandy (2005)
Dormandy JA, Charbonnel B, Eckland DJ, et al. Secondary prevention of macrovascular events in patients with type 2 diabetes in the PROactive Study (PROspective 193acrovascula Clinical Trial In 193acrovascular Events): a randomised controlled trial. *The Lancet* 2005; 366: 1279–1289.

Dregan (2014)
Dregan A, Van Staa TP, McDermott L, et al. Point-of-care cluster randomized trial in stroke secondary prevention using electronic health records. *Stroke* 2014; 45: 2066–2071.

Drummond (2013)
Drummond AER, Whitehead P, Fellows K, et al. Occupational therapy predischarge home visits for patients with a stroke (HOVIS): Results of a feasibility randomized controlled trial. *Clinical Rehabilitation* 2013; 27: 387–397.

Du (2016)

Du J, Yang F, Liu L, et al. Repetitive transcranial magnetic stimulation for rehabilitation of poststroke dysphagia: A randomized, double-blind clinical trial. *Clinical Neurophysiology* 2016; 127: 1907–1913.

Eames (2013)
Eames S, Hoffmann T, Worrall L, et al. Randomised controlled trial of an education and support package for stroke patients and their carers. *BMJ Open* 2013; 3: e002538.

England (2012)
England TJ, Abaei M, Auer DP, et al. Granulocyte-Colony Stimulating Factor for Mobilizing Bone Marrow Stem Cells in Subacute Stroke. *Stroke* 2012; 43: 405–411.

England (2017)
England TJ, Hedstrom A, O'Sullivan S, et al. RECAST (Remote Ischemic Conditioning after Stroke Trial): A Pilot Randomized Placebo Controlled Phase II Trial in Acute Ischemic Stroke. *Stroke* 2017; 48: 1412–1415.

Ginsberg (2013)
Ginsberg MD, Palesch YY, Hill MD, et al. High-dose albumin treatment for acute ischaemic stroke (ALIAS) part 2: A randomised, double-blind, phase 3, placebo-controlled trial. *The Lancet Neurology* 2013; 12: 1049–1058.

Glass (2004)
Glass TA, Berkman LF, Hiltunen EF, et al. The families in recovery from stroke trial (FIRST): Primary study results. *Psychosomatic Medicine* 2004; 66: 889–897.

Globas (2011)
Globas C, Becker C, Cerny J, et al. Chronic Stroke Survivors Benefit From High-Intensity Aerobic Treadmill Exercise: A Randomized Control Trial. *http://dx.doi.org/101177/1545968311418675* 2011; 26: 85–95.

Granger (2011)
Granger CB, Alexander JH, McMurray JJV, et al. Apixaban versus Warfarin in Patients with Atrial Fibrillation. *http://dx.doi.org/101056/NEJMoa1107039* 2011; 365: 981–992.

Gray (2007)
Gray CS, Hildreth AJ, Sandercock PA, et al. Glucose-potassium-insulin infusions in the management of post-stroke hyperglycaemia: the UK Glucose Insulin in Stroke Trial (GIST-UK). *Lancet Neurology* 2007; 6: 397–406.

Gurm (2008)
Gurm HS, Yadav JS, Fayad P, et al. Long-Term Results of Carotid Stenting versus Endarterectomy in High-Risk Patients. *New England Journal of Medicine* 2008; 358: 1572–1579.

Ha (2010)
Ha L, Hauge T, Spenning AB, et al. Individual, nutritional support prevents undernutrition, increases muscle strength and improves QoL among elderly at nutritional risk hospitalized for acute stroke: A randomized, controlled trial. *Clinical Nutrition* 2010; 29: 567–573.

Hacke (2009)
Hacke W, Furlan AJ, Al-Rawi Y, et al. Intravenous desmoteplase in patients with acute ischaemic stroke selected by MRI perfusion-diffusion weighted imaging or perfusion CT (DIAS-2): a prospective, randomised, double-blind, placebo-controlled study. *The Lancet Neurology* 2009; 8: 141–150.

Hacke (2006)
Hacke W, Ringleb PA, Kunze A, et al. 30 day results from the SPACE trial of stent-protected angioplasty versus carotid endarterectomy in symptomatic patients: a randomised non-inferiority trial. *The Lancet* 2006; 368: 1239–1247.

Harold (2008a)
Adams HP, Leira EC, Torner JC, et al. Treating patients with ‘wake-up’ stroke: The experience of the AbESTT-II trial. *Stroke* 2008; 39: 3277–3282.

Harold (2008b)
Harold P. Adams J, Effron MB, Torner J, et al. Emergency Administration of Abciximab for Treatment of Patients With Acute Ischemic Stroke: Results of an International Phase III Trial. *Stroke* 2008; 39: 87–99.

Harwood (2012)
Harwood M, Weatherall M, Talemaitoga A, et al. Taking charge after stroke: Promoting self-directed rehabilitation to improve quality of life-a randomized controlled trial. *Clinical Rehabilitation* 2012; 26: 493–501.

Hedegaard (2014)
Hedegaard U, Kjeldsen LJ, Pottegård A, et al. Multifaceted Intervention Including Motivational Interviewing to Support Medication Adherence after Stroke/Transient Ischemic Attack: A Randomized Trial. *Cerebrovascular Diseases Extra* 2014; 4: 221–234.

Herisson (2016)
Herisson F, Godard S, Volteau C, et al. Early Sitting in Ischemic Stroke Patients (SEVEL): A Randomized Controlled Trial. *PLOS ONE* 2016; 11: e0149466.

Hill (2011)
Hill MD, Martin RH, Palesch YY, et al. The Albumin in Acute Stroke Part 1 Trial. *Stroke* 2011; 42: 1621–1625.

Hofmeijer (2009)
Hofmeijer J, Kappelle LJ, Algra A, et al. Surgical decompression for space-occupying cerebral infarction (the Hemicraniectomy After Middle Cerebral Artery infarction with Life-threatening Edema Trial [HAMLET]): a multicentre, open, randomised trial. *The Lancet Neurology* 2009; 8: 326–333.

Hofstad (2013)
Hofstad H, Naess H, Moe-Nilssen R, et al. Early supported discharge after stroke in Bergen (ESD Stroke Bergen): A randomized controlled trial comparing rehabilitation in a day unit or in the patients’ homes with conventional treatment. *International Journal of Stroke* 2013; 8: 582–587.

Holmgren (2010)
Holmgren E, Gosman-Hedström G, Lindström B, et al. What is the benefit of a high-intensive exercise program on health-related quality of life and depression after stroke? A randomized controlled trial. *Advances in Physiotherapy* 2010; 12: 125–133.

Hornnes (2011)
Hornnes N, Larsen K, Boysen G. Blood Pressure 1 Year after Stroke: The Need to Optimize Secondary Prevention. *Journal of Stroke and Cerebrovascular Diseases* 2011; 20: 16–23.

Hougaard (2014)

Hougaard KD, Hjort N, Zeidler D, et al. Remote ischemic perconditioning as an adjunct therapy to thrombolysis in patients with acute ischemic stroke: a randomized trial. *Stroke* 2014; 45: 159–67.

Immink (2014)
Immink MA, Hillier S, Petkov J. Randomized Controlled Trial of Yoga for Chronic Poststroke Hemiparesis: Motor Function, Mental Health, and Quality of Life Outcomes. *https://doi.org/101310/tsr2103-256* 2014; 21: 256–271.

Jayasekeran (2010)
Jayasekeran V, Singh S, Tyrrell P, et al. Adjunctive Functional Pharyngeal Electrical Stimulation Reverses Swallowing Disability After Brain Lesions. *Gastroenterology* 2010; 138: 1737-1746.e2.

Jüttler (2007)
Jüttler E, Schwab S, Schmiedek P, et al. Decompressive surgery for the treatment of malignant infarction of the middle cerebral artery (DESTINY): A randomized, controlled trial. *Stroke* 2007; 38: 2518–2525.

Kennedy (2007)
Kennedy J, Hill MD, Ryckborst KJ, et al. Fast assessment of stroke and transient ischaemic attack to prevent early recurrence (FASTER): a randomised controlled pilot trial. *The Lancet Neurology* 2007; 6: 961–969.

Kernan (2016)
Kernan WN, Viscoli CM, Furie KL, et al. Pioglitazone after Ischemic Stroke or Transient Ischemic Attack. *https://doi.org/101056/NEJMoa1506930* 2016; 374: 1321–1331.

Kerry (2013)
Kerry SM, Markus HS, Khong TK, et al. Home blood pressure monitoring with nurse-led telephone support among patients with hypertension and a history of stroke: a community-based randomized controlled trial. *CMAJ* 2013; 185: 23–31.

Khedr (2013)
Khedr EM, Shawky OA, El-Hammady DH, et al. Effect of Anodal Versus Cathodal Transcranial Direct Current Stimulation on Stroke Rehabilitation: A Pilot Randomized Controlled Trial. *http://dx.doi.org/101177/1545968313484808* 2013; 27: 592–601.

Kronish (2014)
Kronish IM, Goldfinger JZ, Negron R, et al. Effect of Peer Education on Stroke Prevention. *Stroke* 2014; 45: 3330–3336.

Langhorne (2010)
Langhorne P, Stott D, Knight A, et al. Very Early Rehabilitation or Intensive Telemetry after Stroke: A Pilot Randomised Trial. *Cerebrovascular Diseases* 2010; 29: 352–360.

Lennon (2010)
Lennon OC, Carey A, Creed A, Durcan S, Blake C. Reliability and validity of COOP/WONCA functional health status charts for stroke patients in primary care. J Stroke Cerebrovasc Dis. 2010; 20: 465-73.

Levin (2012)
MF L, O S, DG L, et al. Virtual reality versus conventional treatment of reaching ability in chronic stroke: clinical feasibility study. *Neurology and therapy* 2012; 1: 1–15.

Levy (2009)
Levy DE, del Zoppo GJ, Demaerschalk BM, et al. Ancrod in Acute Ischemic Stroke. *Stroke* 2009; 40: 3796–3803.

Lowe (2006)
Lowe DB, Sharma AK, Leathley MJ. The CareFile Project: a feasibility study to examine the effects of an individualised information booklet on patients after stroke. *Age and Ageing* 2006; 36: 83–89.

Lund (2011)
Lund A, Michelet M, Sandvik L, et al. A lifestyle intervention as supplement to a physical activity programme in rehabilitation after stroke: a randomized controlled trial: *http://dx.doi.org/101177/0269215511429473* 2011; 26: 502–512.

MacMahon (2001)
MacMahon S, Neal B, Tzourio C, et al. Randomised trial of a perindopril-based blood-pressure-lowering regimen among 6105 individuals with previous stroke or transient ischaemic attack. *The Lancet* 2001; 358: 1033–1041.

Mansfield (2018)
Mansfield A, Aqui A, Danells CJ, et al. Does perturbation-based balance training prevent falls among individuals with chronic stroke? A randomised controlled trial. *BMJ Open* 2018; 8: e021510.

Mayer (2009a)
Mayer SA, Brun NC, Begtrup K, et al. Efficacy and Safety of Recombinant Activated Factor VII for Acute Intracerebral Hemorrhage. *http://dx.doi.org/101056/NEJMoa0707534* 2009; 358: 2127–2137.

Mayer (2009b)
Mayer SA, Brun NC, Begtrup K, et al. Recombinant Activated Factor VII for Acute Intracerebral Hemorrhage. *http://dx.doi.org/101056/NEJMoa042991* 2009; 4: 280.

McAlister (2014)
McAlister FA, Grover S, Padwal RS, et al. Case management reduces global vascular risk after stroke: Secondary results from the The preventing recurrent vascular events and neurological worsening through intensive organized case-management randomized controlled trial. *American Heart Journal* 2014; 168: 924–930.

McManus (2014)
McManus RJ, Mant J, Haque MS, et al. Effect of Self-monitoring and Medication Self-titration on Systolic Blood Pressure in Hypertensive Patients at High Risk of Cardiovascular Disease: The TASMIN-SR Randomized Clinical Trial. *JAMA* 2014; 312: 799–808.

Meier (2013)
Meier B, Kalesan B, Mattle HP, et al. Percutaneous Closure of Patent Foramen Ovale in Cryptogenic Embolism. *http://dx.doi.org/101056/NEJMoa1211716* 2013; 368: 1083–1091.

Meng (2012)
Meng R, Asmaro K, Meng L, et al. Upper limb ischemic preconditioning prevents recurrent stroke in intracranial arterial stenosis. *Neurology* 2012; 79: 1853–1861.

Mohr (2009)
Mohr JP, Thompson JLP, Lazar RM, et al. A Comparison of Warfarin and Aspirin for the Prevention of Recurrent Ischemic Stroke. *http://dx.doi.org/101056/NEJMoa011258* 2009;

345: 1444–1451.

Molina (2009)
Molina CA, Barreto AD, Tsivgoulis G, et al. Transcranial ultrasound in clinical sonothrombolysis (TUCSON) trial. *Annals of Neurology* 2009; 66: 28–38.

Nagapattinam (2015)
Nagapattinam S. EFFECT OF TASK SPECIFIC MIRROR THERAPY WITH FUNCTIONAL ELECTRICAL STIMULATION ON UPPER LIMB FUNCTION FOR SUBACUTE HEMIPLEGIA. *International Journal of Physiotherapy* 2015; 2: 840–849.

O'Carroll (2013)
O'Carroll RE, Chambers JA, Dennis M, et al. Improving Adherence to Medication in Stroke Survivors: A Pilot Randomised Controlled Trial. *Annals of Behavioral Medicine* 2013; 46: 358–368.

Palmer (2012)
Palmer R, Enderby P, Cooper C, et al. Computer therapy compared with usual care for people with long-standing aphasia poststroke: A pilot randomized controlled trial. *Stroke* 2012; 43: 1904–1911.

Park (2013)
Park J-W, Oh J-C, Lee J-W, et al. The effect of 5Hz high-frequency rTMS over contralesional pharyngeal motor cortex in post-stroke oropharyngeal dysphagia: a randomized controlled study. *Neurogastroenterology & Motility* 2013; 25: 324-e250.

Poletto (2015)
Poletto SR, Rebello LC, Valença MJM, et al. Early Mobilization in Ischemic Stroke: A Pilot Randomized Trial of Safety and Feasibility in a Public Hospital in Brazil. *Cerebrovascular Diseases Extra* 2015; 5: 31–40.
Poppitt (2009)
Poppitt SD, Howe CA, Lithander FE, et al. Effects of Moderate-Dose Omega-3 Fish Oil on Cardiovascular Risk Factors and Mood After Ischemic Stroke. *Stroke* 2009; 40: 3485–3492.

Prange (2014)
Prange GB, Kottink AIR, Buurke JH, et al. The Effect of Arm Support Combined With Rehabilitation Games on Upper-Extremity Function in Subacute Stroke: A Randomized Controlled Trial. *http://dx.doi.org/101177/1545968314535985* 2014; 29: 174–182.

Rabadi (2008)
Rabadi MH, Coar PL, Lukin M, et al. Intensive nutritional supplements can improve outcomes in stroke rehabilitation. *Neurology* 2008; 71: 1856–1861.

Rasmussen (2016)
Rasmussen RS, Østergaard A, Kjær P, et al. Stroke rehabilitation at home before and after discharge reduced disability and improved quality of life: A randomised controlled trial. *Clinical Rehabilitation* 2016; 30: 225–236.

Rehani (2015)
REHANI P, KUMARI R, MIDHA D. EFFECTIVENESS OF MOTOR RELEARNING PROGRAMME AND MIRROR THERAPY ON HAND FUNCTIONS IN PATIENTS WITH STROKE-A RANDOMIZED CLINICAL TRIAL. *International Journal of Therapies and Rehabilitation Research* 2015; 4: 20.

Ross (2009)

Ross LF, Harvey LA, Lannin NA. Do people with acquired brain impairment benefit from additional therapy specifically directed at the hand? A randomized controlled trial: *http://dx.doi.org/101177/0269215508101733* 2009; 23: 492–503.

Saal (2015)
Saal S, Becker C, Lorenz S, et al. Effect of a stroke support service in Germany: A randomized trial. *Topics in Stroke Rehabilitation* 2015; 22: 429–436.

Sandset (2011)
Sandset EC, Bath PM, Boysen G, et al. The angiotensin-receptor blocker candesartan for treatment of acute stroke (SCAST): a randomised, placebo-controlled, double-blind trial. *The Lancet* 2011; 377: 741–750.

Saposnik (2016)
Saposnik G, Cohen LG, Mamdani M, et al. Efficacy and safety of non-immersive virtual reality exercising in stroke rehabilitation (EVREST): a randomised, multicentre, single-blind, controlled trial. *The Lancet Neurology* 2016; 15: 1019–1027.

Saposnik (2010)
Saposnik G, Teasell R, Mamdani M, et al. Effectiveness of virtual reality using wii gaming technology in stroke rehabilitation: A pilot randomized clinical trial and proof of principle. *Stroke* 2010; 41: 1477–1484.

Schick (2017)
Schick T, Schlake HP, Kallusky J, et al. Synergy effects of combined multichannel EMG-triggered electrical stimulation and mirror therapy in subacute stroke patients with severe or very severe arm/hand paresis. *Restorative Neurology and Neuroscience* 2017; 35: 319–332.

Shaw (2014)
Shaw L, Price C, McLure S, et al. Paramedic Initiated Lisinopril For Acute Stroke Treatment (PIL-FAST): results from the pilot randomised controlled trial. *Emergency Medicine Journal* 2014; 31: 994–999.

Sheffler (2013)
Sheffler LR, Taylor PN, Gunzler DD, et al. Randomized Controlled Trial of Surface Peroneal Nerve Stimulation for Motor Relearning in Lower Limb Hemiparesis. *Archives of Physical Medicine and Rehabilitation* 2013; 94: 1007–1014.

Simpson (2009)
Simpson DM, Gracies JM, Yablon SA, et al. Botulinum neurotoxin versus tizanidine in upper limb spasticity: a placebo-controlled study. *Journal of Neurology, Neurosurgery & Psychiatry* 2009; 80: 380–385.

Spielmann (2016)
Spielmann K, van de Sandt-Koenderman WME, Heijenbrok-Kal MH, et al. Transcranial direct current stimulation in post-stroke sub-acute aphasia: Study protocol for a randomized controlled trial. *Trials* 2016; 17: 380.

Standen (2016)
Standen P, Threapleton K, Richardson A, et al. A low cost virtual reality system for home based rehabilitation of the arm following stroke: a randomised controlled feasibility trial: *http://dx.doi.org/101177/0269215516640320* 2016; 31: 340–350.

Stoykov (2009)
Stoykov ME, Lewis GN, Corcos DM. Comparison of bilateral and unilateral training for upper

extremity hemiparesis in stroke. *Neurorehabilitation and Neural Repair* 2009; 23: 945–953.

Subramanian (2013)
Subramanian SK, Lourenço CB, Chilingaryan G, et al. Arm motor recovery using a virtual reality intervention in chronic stroke: Randomized control trial. *Neurorehabilitation and Neural Repair* 2013; 27: 13–23.

Sullivan (2007)
Sullivan KJ, Brown DA, Klassen T, et al. Effects of Task-Specific Locomotor and Strength Training in Adults Who Were Ambulatory After Stroke: Results of the STEPS Randomized Clinical Trial. *Physical Therapy* 2007; 87: 1580–1602.

Sundseth (2012)
Sundseth A, Thommessen B, Rønning OM. Outcome After Mobilization Within 24 Hours of Acute Stroke. *Stroke* 2012; 43: 2389–2394.

Tavazzi (2008)
L T, AP M, R M, et al. Effect of n-3 polyunsaturated fatty acids in patients with chronic heart failure (the GISSI-HF trial): a randomised, double-blind, placebo-controlled trial. *Lancet (London, England)* 2008; 372: 1223–1230.

Thieme (2013)
H T, M B, M W, et al. Mirror therapy for patients with severe arm paresis after stroke–a randomized controlled trial. *Clinical rehabilitation* 2013; 27: 314–324.

Todd (2005)
Todd MM, Hindman BJ, Clarke WR, et al. Mild Intraoperative Hypothermia during Surgery for Intracranial Aneurysm. *New England Journal of Medicine* 2005; 352: 135–145.

Tomić (2017)
Tomić TJD, Savić AM, Vidaković AS, et al. ArmAssist robotic system versus matched conventional therapy for poststroke upper limb rehabilitation: A randomized clinical trial. *BioMed Research International*; 2017. Epub ahead of print 2017. DOI: 10.1155/2017/7659893.

Tyson (2015)
Tyson S, Wilkinson J, Thomas N, et al. Phase II Pragmatic Randomized Controlled Trial of Patient-Led Therapies (Mirror Therapy and Lower-Limb Exercises) During Inpatient Stroke Rehabilitation: *http://dx.doi.org/101177/1545968314565513* 2015; 29: 818–826.

Vanoglio (2017)
Vanoglio F, Bernocchi P, Mulè C, et al. Feasibility and efficacy of a robotic device for hand rehabilitation in hemiplegic stroke patients: A randomized pilot controlled study. *Clinical Rehabilitation* 2017; 31: 351–360.

Varley (2016)
Varley R, Cowell PE, Dyson L, et al. Self-Administered Computer Therapy for Apraxia of Speech. *Stroke* 2016; 47: 822–828.

Villafañe (2017)
Villafañe JH, Taveggia G, Galeri S, et al. Efficacy of Short-Term Robot-Assisted Rehabilitation in Patients With Hand Paralysis After Stroke: A Randomized Clinical Trial: *https://doi.org/101177/1558944717692096* 2017; 13: 95–102.

Weitz (2010)

JI W, SJ C, I P, et al. Randomised, parallel-group, multicentre, multinational phase 2 study comparing edoxaban, an oral factor Xa inhibitor, with warfarin for stroke prevention in patients with atrial fibrillation. *Thrombosis and haemostasis* 2010; 104: 633–641.

Wolf (2015)
Wolf SL, Sahu K, Bay RC, et al. The HAAPI (Home Arm Assistance Progression Initiative) Trial: A Novel Robotics Delivery Approach in Stroke Rehabilitation. *http://dx.doi.org/101177/1545968315575612* 2015; 29: 958–968.

Wolf (2006)
Wolf SL, Winstein CJ, Miller JP, et al. Effect of constraint-induced movement therapy on upper extremity function 3 to 9 months after stroke: The EXCITE randomized clinical trial. *Journal of the American Medical Association* 2006; 296: 2095–2104.

Wu (2012)
Wu C, Yang C, Chuang L, et al. Effect of Therapist-Based Versus Robot-Assisted Bilateral Arm Training on Motor Control, Functional Performance, and Quality of Life After Chronic Stroke: A Clinical Trial. *Physical Therapy* 2012; 92: 1006–1016.

Zedlitz (2012)
Zedlitz AMEE, Rietveld TCM, Geurts AC, et al. Cognitive and graded activity training can alleviate persistent fatigue after stroke: A randomized, controlled trial. *Stroke* 2012; 43: 1046–1051.

References

1. das Nair R, Cogger H, Worthington E, et al. Cognitive rehabilitation for memory deficits after stroke. *Cochrane Database Syst Rev*; 9. Epub ahead of print 1 September 2016. DOI: 10.1002/14651858.CD002293.pub3.

2. Schryver ELD, Algra A, Kappelle LJ, et al. Vitamin K antagonists versus antiplatelet therapy after transient ischaemic attack or minor ischaemic stroke of presumed arterial origin. *Cochrane Database Syst Rev*. Epub ahead of print 2012. DOI: 10.1002/14651858.cd001342.pub3.

3. Todhunter-Brown A, Baer G, Campbell P, et al. Physical rehabilitation approaches for the recovery of function and mobility following stroke. *Cochrane Database Syst Rev*. Epub ahead of print 2014. DOI: 10.1002/14651858.CD001920.pub3.

4. Bath PM, Krishnan K. Interventions for deliberately altering blood pressure in acute stroke. *Cochrane Database Syst Rev*. Epub ahead of print 2014. DOI: 10.1002/14651858.CD000039.pub3.

5. Denissen S, Staring W, Kunkel D, et al. Interventions for preventing falls in people after stroke. *Cochrane Database Syst Rev*. Epub ahead of print 2019. DOI: 10.1002/14651858.CD008728.pub3.

6. Mehrholz J, Pohl M, Platz T, et al. Electromechanical and robot-assisted arm training for improving activities of daily living, arm function, and arm muscle strength after stroke. *Cochrane Database Syst Rev*. Epub ahead of print 2018. DOI: 10.1002/14651858.CD006876.pub5.

7. Loetscher T, Potter K-J, Wong D, et al. Cognitive rehabilitation for attention deficits following stroke. *Cochrane Database Syst Rev*. Epub ahead of print 2019. DOI:

10.1002/14651858.CD002842.pub3.

8. Vloothuis JD, Mulder M, Veerbeek JM, et al. Caregiver-mediated exercises for improving outcomes after stroke. *Cochrane Database Syst Rev*. Epub ahead of print 2016. DOI: 10.1002/14651858.CD011058.pub2.

9. Bath PM, Krishnan K, Appleton JP. Nitric oxide donors (nitrates), L-arginine, or nitric oxide synthase inhibitors for acute stroke. *Cochrane Database Syst Rev*. Epub ahead of print 2017. DOI: 10.1002/14651858.CD000398.pub2.

10. Bridgwood B, Lager KE, Mistri AK, et al. Interventions for improving modifiable risk factor control in the secondary prevention of stroke. *Cochrane Database Syst Rev*; 2022. Epub ahead of print 2018. DOI: 10.1002/14651858.cd009103.pub3.

11. Mendes LA, Lima IN, Souza T, et al. Motor neuroprosthesis for promoting recovery of function after stroke. *Cochrane Database Syst Rev*. Epub ahead of print 2020. DOI: 10.1002/14651858.CD012991.pub2.

12. Brady MC, Kelly H, Godwin J, et al. Speech and language therapy for aphasia following stroke. *Cochrane Database Syst Rev*. Epub ahead of print 2016. DOI: 10.1002/14651858.CD000425.pub4.

13. Corbetta D, Sirtori V, Castellini G, et al. Constraint-induced movement therapy for upper extremities in people with stroke. *Cochrane Database Syst Rev*. Epub ahead of print 2015. DOI: 10.1002/14651858.CD004433.pub3.

14. Bath PM, Lee HS, Everton LF. Swallowing therapy for dysphagia in acute and subacute stroke. *Cochrane Database Syst Rev*; 2018. Epub ahead of print 2018. DOI: 10.1002/14651858.cd000323.pub3.

15. Boncoraglio GB, Ranieri M, Bersano A, et al. Stem cell transplantation for ischemic stroke. *Cochrane Database Syst Rev*. Epub ahead of print 2019. DOI: 10.1002/14651858.cd007231.pub3.

16. Laver KE, Adey-Wakeling Z, Crotty M, et al. Telerehabilitation services for stroke. *Cochrane Database Syst Rev*. Epub ahead of print 2020. DOI: 10.1002/14651858.CD010255.pub3.

17. Lindekleiv H, Berge E, Slot KMB, et al. Percutaneous vascular interventions versus intravenous thrombolytic treatment for acute ischaemic stroke. *Cochrane Database Syst Rev*. Epub ahead of print 2018. DOI: 10.1002/14651858.CD009292.pub2.

18. Mehrholz J, Thomas S, Elsner B. Treadmill training and body weight support for walking after stroke. *Cochrane Database Syst Rev*; 2017. Epub ahead of print 2017. DOI: 10.1002/14651858.cd002840.pub4.

19. Salazar CA, Aguila D del, Cordova EG. Direct thrombin inhibitors versus vitamin K antagonists for preventing cerebral or systemic embolism in people with non-valvular atrial fibrillation. *Cochrane Database Syst Rev*. Epub ahead of print 2014. DOI: 10.1002/14651858.CD009893.pub2.

20. Slot KMB, Berge E. Factor Xa inhibitors versus vitamin K antagonists for preventing cerebral or systemic embolism in patients with atrial fibrillation. *Cochrane Database Syst Rev*; 2018. Epub ahead of print 2018. DOI: 10.1002/14651858.cd008980.pub3.

21. Borges LR, Fernandes AB, Melo LP, et al. Action observation for upper limb rehabilitation after stroke. *Cochrane Database Syst Rev*. Epub ahead of print 2018. DOI: 10.1002/14651858.CD011887.pub2.

22. Wardlaw JM, Murray V, Berge E, et al. Thrombolysis for acute ischaemic stroke. *Cochrane Database Syst Rev*. Epub ahead of print 2014. DOI: 10.1002/14651858.CD000213.pub3.

23. Geeganage C, Beavan J, Ellender S, et al. Interventions for dysphagia and nutritional support in acute and subacute stroke. *Cochrane Database Syst Rev*. Epub ahead of print 2012. DOI: 10.1002/14651858.cd000323.pub2.

24. Naccarato M, Grandi FC, Dennis M, et al. Physical methods for preventing deep vein thrombosis in stroke. *Cochrane Database Syst Rev*. Epub ahead of print 2010. DOI: 10.1002/14651858.CD001922.pub3.

25. Hertog HMD, Worp HB van der, Tseng M-C, et al. Cooling therapy for acute stroke. *Cochrane Database Syst Rev*. Epub ahead of print 2009. DOI: 10.1002/14651858.cd001247.pub2.

26. Thieme H, Morkisch N, Mehrholz J, et al. Mirror therapy for improving motor function after stroke. *Cochrane Database Syst Rev*; 2018. Epub ahead of print 2018. DOI: 10.1002/14651858.CD008449.pub3.

27. Liu J, Wang L-N. Peroxisome proliferator-activated receptor gamma agonists for preventing recurrent stroke and other vascular events in people with stroke or transient ischaemic attack. *Cochrane Database Syst Rev*; 2019. Epub ahead of print 2019. DOI: 10.1002/14651858.cd010693.pub5.

28. Bath PM, Sprigg N, England T. Colony stimulating factors (including erythropoietin, granulocyte colony stimulating factor and analogues) for stroke. *Cochrane Database Syst Rev*. Epub ahead of print 2013. DOI: 10.1002/14651858.CD005207.pub4.

29. Zhao W, Zhang J, Sadowsky MG, et al. Remote ischaemic conditioning for preventing and treating ischaemic stroke. *Cochrane Database Syst Rev*. Epub ahead of print 2018. DOI: 10.1002/14651858.CD012503.pub2.

30. Chang TS, Jensen MB. Haemodilution for acute ischaemic stroke. *Cochrane Database Syst Rev*. Epub ahead of print 2014. DOI: 10.1002/14651858.cd000103.pub2.

31. Ellis G, Mant J, Langhorne P, et al. Stroke liaison workers for stroke patients and carers: an individual patient data meta-analysis. *Cochrane Database Syst Rev*. Epub ahead of print 2010. DOI: 10.1002/14651858.cd005066.pub2.

32. Geeganage C, Bath PM. Vasoactive drugs for acute stroke. *Cochrane Database Syst Rev*. Epub ahead of print 2010. DOI: 10.1002/14651858.cd002839.pub2.

33. Bonati LH, Lyrer P, Ederle J, et al. Percutaneous transluminal balloon angioplasty and stenting for carotid artery stenosis. *Cochrane Database Syst Rev*. Epub ahead of print 2012. DOI: 10.1002/14651858.cd000515.pub4.

34. Wardlaw JM, Koumellis P, Liu M. Thrombolysis (different doses, routes of administration and agents) for acute ischaemic stroke. *Cochrane Database Syst Rev*. Epub ahead of print 2013. DOI: 10.1002/14651858.CD000514.pub3.

35. Ciccone A, Motto C, Abraha I, et al. Glycoprotein IIb-IIIa inhibitors for acute ischaemic stroke. *Cochrane Database Syst Rev*. Epub ahead of print 2014. DOI: 10.1002/14651858.cd005208.pub3.

36. Sandercock PA, Counsell C, Gubitz GJ, et al. Antiplatelet therapy for acute ischaemic stroke. *Cochrane Database Syst Rev*. Epub ahead of print 2008. DOI: 10.1002/14651858.cd000029.pub2.

37. Fryer CE, Luker JA, Mcdonnell MN, et al. Self management programmes for quality of life in people with stroke. *Cochrane Database of Systematic Reviews*; 2016. Epub ahead of print August 2016. DOI: 10.1002/14651858.CD010442.pub2.

38. Langhorne P, Collier JM, Bate PJ, et al. Very early versus delayed mobilisation after stroke. *Cochrane Database Syst Rev*; 2018. Epub ahead of print 2018. DOI: 10.1002/14651858.cd006187.pub3.

39. Cruz-Flores S, Berge E, Whittle IR. Surgical decompression for cerebral oedema in acute ischaemic stroke. *Cochrane Database Syst Rev*. Epub ahead of print 2012. DOI: 10.1002/14651858.cd003435.pub2.

40. Langhorne P, Baylan S, Trialists ESD. Early supported discharge services for people with acute stroke. *Cochrane Database Syst Rev*; 2017. Epub ahead of print 2017. DOI: 10.1002/14651858.cd000443.pub4.

41. French B, Thomas LH, Coupe J, et al. Repetitive task training for improving functional ability after stroke. *Cochrane Database Syst Rev*; 2016. Epub ahead of print 2016. DOI: 10.1002/14651858.cd006073.pub3.

42. Lawrence M, Junior FTC, Matozinho HH, et al. Yoga for stroke rehabilitation. *Cochrane Database Syst Rev*; 2017. Epub ahead of print 2017. DOI: 10.1002/14651858.cd011483.pub2.

43. Manktelow BN, Potter JF. Interventions in the management of serum lipids for preventing stroke recurrence. *Cochrane Database Syst Rev*. Epub ahead of print 2009. DOI: 10.1002/14651858.cd002091.pub2.

44. Elsner B, Kugler J, Pohl M, et al. Transcranial direct current stimulation (tDCS) for improving function and activities of daily living in patients after stroke. *Cochrane Database Syst Rev*. Epub ahead of print 2013. DOI: 10.1002/14651858.CD009645.pub2.

45. MacKay-Lyons M, Thornton M, Ruggles T, et al. Non-pharmacological interventions for preventing secondary vascular events after stroke or transient ischemic attack. *Cochrane Database Syst Rev*. Epub ahead of print 2013. DOI: 10.1002/14651858.CD008656.pub2.

46. Laver KE, Lange B, George S, et al. Virtual reality for stroke rehabilitation. *Cochrane Database Syst Rev*. Epub ahead of print 2017. DOI: 10.1002/14651858.CD008349.pub4.

47. Hao Z, Liu M, Counsell C, et al. Fibrinogen depleting agents for acute ischaemic stroke. *Cochrane Database Syst Rev*. Epub ahead of print 2012. DOI: 10.1002/14651858.CD000091.pub2.

48. Forster A, Brown L, Smith J, et al. Information provision for stroke patients and their

caregivers. *Cochrane Database Syst Rev*. Epub ahead of print 2012. DOI: 10.1002/14651858.CD001919.pub3.

49. Zonneveld TP, Richard E, Vergouwen MD, et al. Blood pressure-lowering treatment for preventing recurrent stroke, major vascular events, and dementia in patients with a history of stroke or transient ischaemic attack. *Cochrane Database Syst Rev*. Epub ahead of print 2018. DOI: 10.1002/14651858.CD007858.pub2.

50. Salman RA-S, Law ZK, Bath PM, et al. Haemostatic therapies for acute spontaneous intracerebral haemorrhage. *Cochrane Database Syst Rev*. Epub ahead of print 2018. DOI: 10.1002/14651858.CD005951.pub4.

51. You H, Salman RA-S. Haemostatic drug therapies for acute primary intracerebral haemorrhage. *Cochrane Database Syst Rev*. Epub ahead of print 2006. DOI: 10.1002/14651858.CD005951.pub2.

52. Li J, Liu J, Liu M, et al. Closure versus medical therapy for preventing recurrent stroke in patients with patent foramen ovale and a history of cryptogenic stroke or transient ischemic attack. *Cochrane Database Syst Rev*. Epub ahead of print 2015. DOI: 10.1002/14651858.CD009938.pub2.

53. Algra A, Schryver ELD, Gijn J van, et al. Oral anticoagulants versus antiplatelet therapy for preventing further vascular events after transient ischaemic attack or minor stroke of presumed arterial origin. *Cochrane Database Syst Rev*. Epub ahead of print 2006. DOI: 10.1002/14651858.CD001342.pub2.

54. Ricci S, Dinia L, Sette MD, et al. Sonothrombolysis for acute ischaemic stroke. *Cochrane Database Syst Rev*. Epub ahead of print 2012. DOI: 10.1002/14651858.CD008348.pub3.

55. Campano CGA, Macleod MJ, Aucott L, et al. Marine-derived n-3 fatty acids therapy for stroke. *Cochrane Database Syst Rev*. Epub ahead of print 2019. DOI: 10.1002/14651858.CD012815.pub2.

56. Lindsay C, Kouzouna A, Simcox C, et al. Pharmacological interventions other than botulinum toxin for spasticity after stroke. *Cochrane Database Syst Rev*. Epub ahead of print 2016. DOI: 10.1002/14651858.CD010362.pub2.

57. Elsner B, Kugler J, Pohl M, et al. Transcranial direct current stimulation (tDCS) for improving aphasia in adults with aphasia after stroke. *Cochrane Database Syst Rev*. Epub ahead of print 2019. DOI: 10.1002/14651858.CD009760.pub4.

58. Coupar F, Pollock A, Wijck F van, et al. Simultaneous bilateral training for improving arm function after stroke. *Cochrane Database Syst Rev*. Epub ahead of print 2010. DOI: 10.1002/14651858.CD006432.pub2.

59. Li LR, You C, Chaudhary B. Intraoperative mild hypothermia for postoperative neurological deficits in people with intracranial aneurysm. *Cochrane Database Syst Rev*. Epub ahead of print 2016. DOI: 10.1002/14651858.CD008445.pub3.

60. Sirtori V, Corbetta D, Moja L, et al. Constraint-induced movement therapy for upper extremities in stroke patients. *Cochrane Database Syst Rev*. Epub ahead of print 2009. DOI: 10.1002/14651858.cd004433.pub2.

61. Wu S, Kutlubaev MA, Chun H-YY, et al. Interventions for post-stroke fatigue. *Cochrane*

Database Syst Rev. Epub ahead of print 2015. DOI: 10.1002/14651858.CD007030.pub3.
